# Enantioselective α,α-Chlorofluorination of Sulfoxonium Ylides

**DOI:** 10.1021/acs.orglett.5c05062

**Published:** 2026-01-28

**Authors:** Lucas G. Furniel, Kauê C. Capellaro, Viktor S. Câmara, Marcio Hayashi, Radell Echemendía, Camila B. Pinto, Ana B. A. M. Salata, Jackson A. L. Filho, Leandro W. Hantao, Javier Ellena, Antonio C. B. Burtoloso

**Affiliations:** † Chemistry Institute of São Carlos, University of São Paulo, CEP 13560-970, São Carlos, SP 05508-220, Brazil; ‡ São Carlos Institute of Physics, University of São Paulo, SP CEP 13560-970, São Carlos 05508-220, Brazil; § Chemistry Institute, University of Campinas, CEP 13083-862, Campinas 13083-970, Brazil

## Abstract

The first examples of enantioselective α,α-chlorofluorination of α-carbonylsulfoxonium ylides are described. Herein, two modes of reaction are explored, using chiral aminosulfoxonium ylides and a chiral fluorinating reagent, prepared *in situ* from commercially available cinchona alkaloids. Using these approaches, 25 examples of gem-dihalogenated compounds (including bromochlorination) were obtained in good yields (up to 90%) and enantioselectivity (up to 96:4 er).

## Graphical abstract

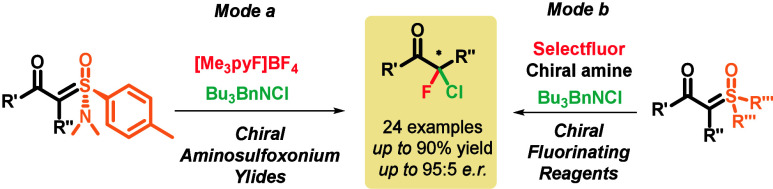

Fluorine substituents can significantly alter the p*K*_a_ of neighboring groups and affect properties such as dipole moments, metabolic stability, lipophilicity, and bioavailability.^1,2^ These characteristics have made fluorine ubiquitous in agrochemicals and pharmaceuticals.^3–5^ Due to these important characteristics and the rarity of naturally occurring fluorine-containing molecules,^6^ several methodologies for C–F bond formation have been developed over the past 30 years.^7–11^ Despite numerous advances in this field, fewer than 1% of pharmaceuticals containing fluorine atoms have a C–F bond at chiral nonracemic carbon centers, primarily due to the inherent challenges in constructing these centers.^12^ To address this limitation, various strategies have been employed to enable the asymmetric formation of C–F bonds with high enantioselectivity, with particular focus on α-fluorocarbonyl compounds.^7–16^

In contrast to these advances in asymmetric α-monofluorination, reports of enantioselective α,α-chlorofluorination are rare.^17–27^ To the best of our knowledge, only 10 studies have been published to date. In each of these cases, two separate steps are required: one for the installation of the first halogen in a racemic fashion followed by asymmetric monohalogenation of the previously halogenated substrate (Scheme 1a). These strategies are limited to aldehydes or dicarbonyl compounds and, in many cases, suffer from low yields in the first halogenation step due to the unwanted formation of difluorinated or dichlorinated side products. These limitations make the development of a one-step asymmetric α,α-chlorofluorination reaction a significant challenge in organic synthesis. Once formed, α,α-chlorofluorocarbonyl compounds are highly versatile synthetic intermediates. They are known to participate in carbonyl reductions, olefination reactions, organometallic additions, nucleophilic substitutions, and other reactions, allowing the preparation of various organofluorine skeletons without a loss of enantiopurity.^21,22,28^

**Scheme 1:**
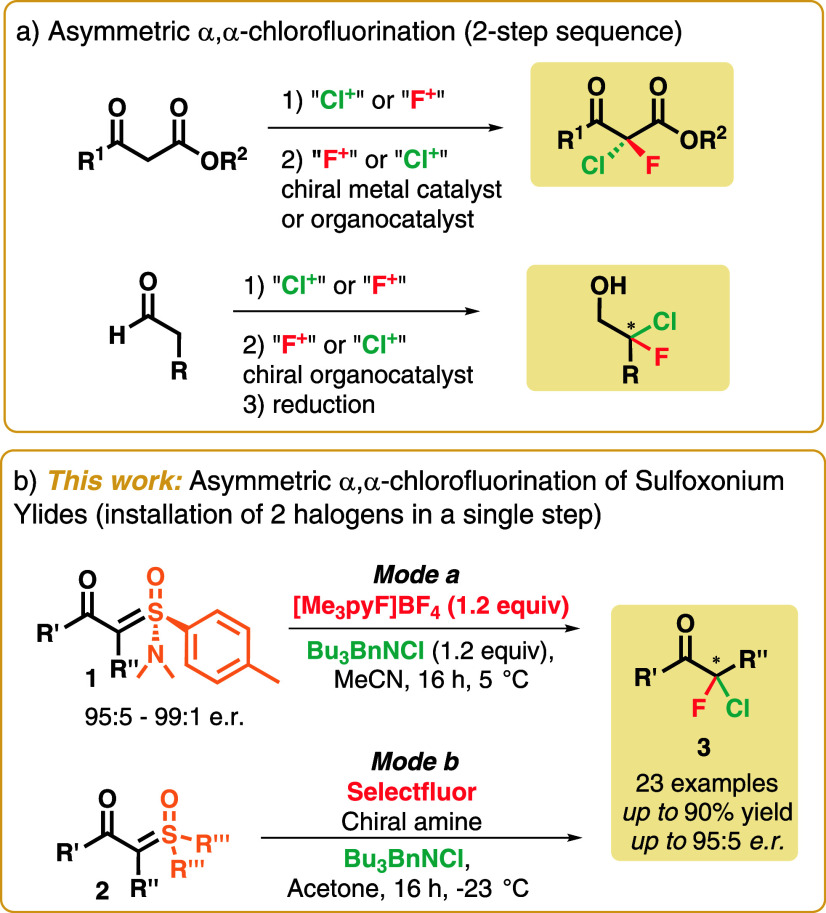
(a) Asymmetric α,α-Chlorofluorination of Carbonyl Compounds and (b) Proposed Asymmetric α,α-Chlorofluorination of α-Carbonyl Sulfoxonium Ylides by Two Different Modes

Sulfoxonium ylides are a class of compounds characterized by a carbanion directly attached to a positively charged sulfoxonium moiety.^29–32^ Due to this unique structure, they can act as both a nucleophile (through carbanion attack) and an electrophile (with neutral DMSO acting as a leaving group) within the same reaction.^29–32^ This dual reactivity makes them prime candidates for α,α-difunctionalization reactions with halogens. The first studies exploring this reactivity pattern date back to 1964,^33,34^ but a more comprehensive methodology has only been developed recently. In 2017 and 2021, our research group published consecutive studies detailing α,α-difunctionalization reactions with sulfoxonium ylides, leading to the preparation of a variety of highly functionalized products.^35,36^ This strategy eliminates ambiguity, allowing the use of one nucleophilic halogen and one electrophilic halogen within the same reaction vessel. However, despite these advances, all examples have been limited to the production of racemic products. As part of our group’s ongoing efforts to explore sulfoxonium ylide chemistry in asymmetric transformations,^37–39^ we describe herein the first asymmetric one-step α,α-chlorofluorination of α-carbonyl sulfoxonium ylides by two different modes: (a) the use of chiral Johnson‘s amino-sulfoxonium ylides **1** and (b) the use of Shibata’s chiral fluorinating agents, based on cinchona alkaloids (Scheme 1b). It is worth noting that in the case of Johnson’s amino-sulfoxonium ylides, this is the first demonstration of these important ylides being able to produce products with high enantiomeric excesses.^40,41^

We started our work employing the (+)-(*R*)-(dimethylamino)­ethyl-*p*-toIyloxosulfonium tetrafluoroborate salt, previously described by Johnson (see the Supporting Information for its preparation).^40,41^ Acylation with benzoyl chloride in the presence of NaH provided ylide **1** in 94% yield and 98:2 enantiomeric ratio. The absolute configuration of ylide **1h** was determined by single-crystal X-ray diffraction (SCXRD) analysis (Figure S1). Chiral ylide **1a** was then subjected to the chlorofluorination reaction under several reaction conditions and different sources of electrophilic/nucleophilic halogens to provide product **3a** (Table 1).

**Table 1:**
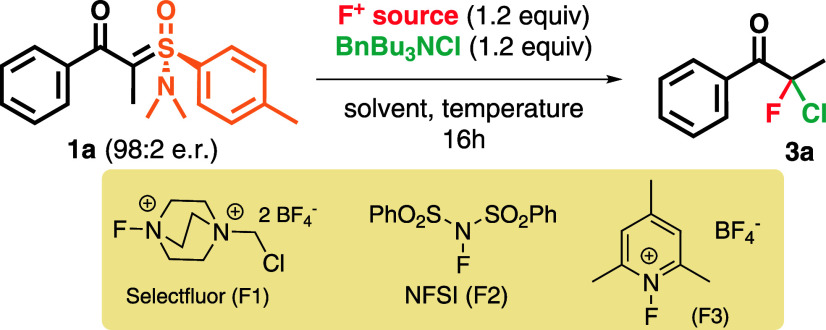
Optimization of the α,α-Chlorofluorination of Chiral Johnson’s Amino-sulfoxonium Ylides **1** ^a^

entry	F^+^ source	solvent	*T* (°C)	yield^b^ (%)	er
1	**F1**	MeCN	rt	36	71:29
2	**F1**	MeCN	5	41	74:26
3	**F1**	DMSO	5	20	75:25
4	**F1**	AcOEt	5	28	70:30
5	**F1**	DCM	5	61	69:31
6^c^	**F1**	MeCN	5	4	62:38
7^c^	**F1**	DCM	5	26	69:31
8	**F2**	MeCN	5	86	66:34
9	**F3**	MeCN	5	75	95:5
10	**F3**	MeCN	rt	38	94:6
11	**F3**	MeCN	–23	68	95:5
12	**F3**	MeCN	–40	70	91:9
13	**F3**	CHCl_3_	5	39	92:8
14	**F3**	CF_3_CH_2_OH	5	5	89:11
15	**F2**	DMF	5	58	95:5
16	**F3**	1,2-DCE	5	51	94:6

a Reaction conditions: **1** (0.15 mmol), “F^+^” (0.18 mmol), BnBu_3_NCl (0.18 mmol), MeCN (1.5 mL, 0.1 M), 16 h.

b Isolated yield.

c KCl (2.0 equiv) instead of BnBu_3_NCl.

We began our optimization studies using MeCN as the solvent for the reaction at room temperature and 5 °C, with entry 2 providing a superior result. Next, different solvents (entries 3–5) and KCl as a chloride source (entries 6 and 7) were screened, but no enhancement of the enantioselectivity was observed. However, the source of the electrophilic fluoride proved to have a profound impact on the enantioselectivity. First, using NFSI instead of Selectfluor (entry 8) did not result in improvement, but with pyridine-based “F^+^” reagent **F3**, the product was formed with 75% yield and 95:5 er (entry 9). Using this reagent at lower and higher temperatures (entries 10–12) and in combination with different solvents (entries 13–16) did not provide better selectivity of the product compared to entry 9 but did afford a reduced yield. With an efficient condition for the asymmetric chlorofluorination reaction (95:5 er) from chiral ylide **1a**, we next extended our investigation by preparing 14 new ylides (**1a**–**1n**). These ylides were prepared in high enantiomeric ratios and then subjected to the conditions depicted in entry 9 of Table 1 (Scheme 2).

**Scheme 2:**
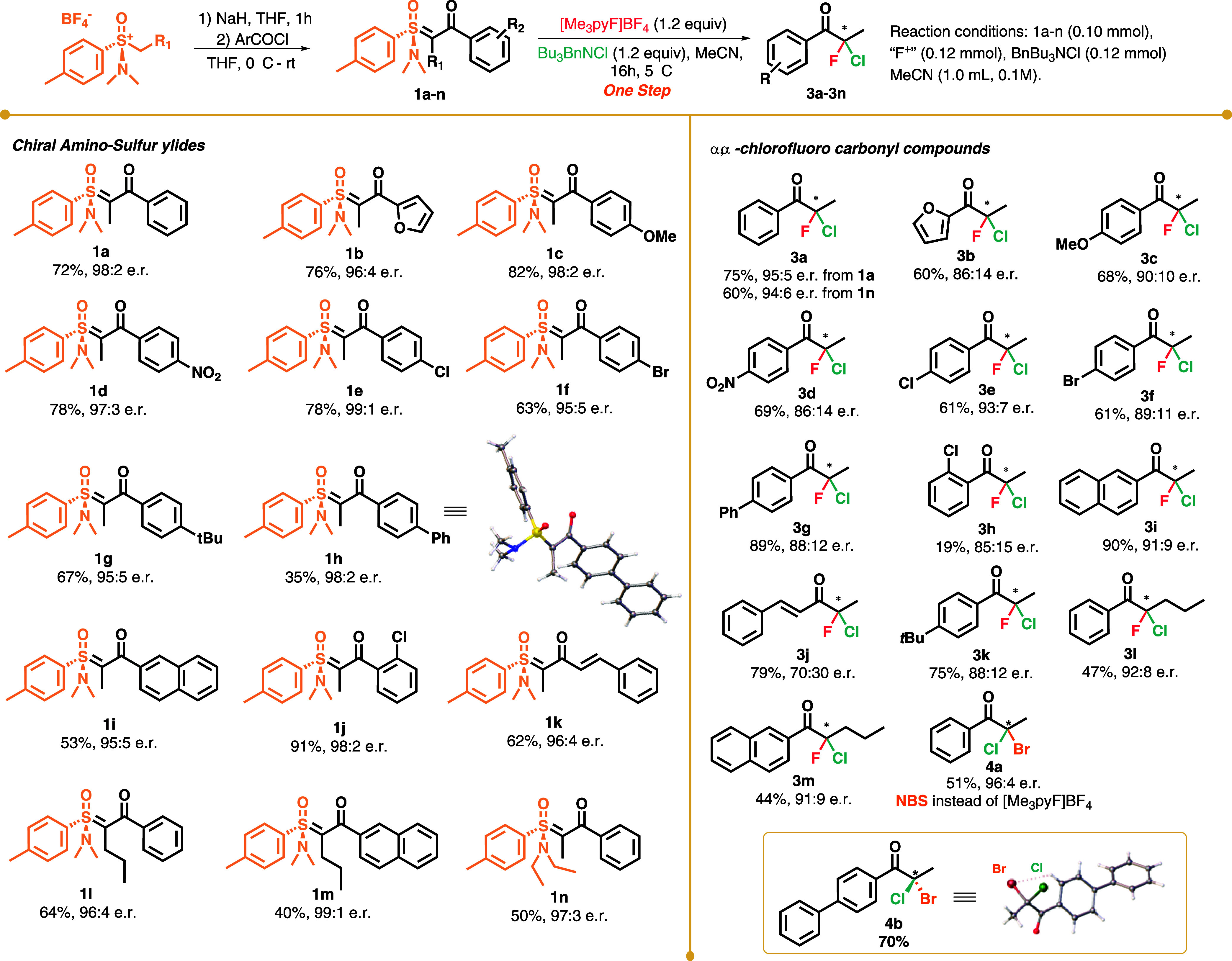
Substrate Scope for the Chlorofluorination of Chiral Johnson’s Amino-sulfoxonium Ylides

2-Furyl product **3b** was obtained with moderate enantioselectivity (86:14 er). *para*-substituted amino-sulfoxonium ylides furnished products **3c**–**3g** and **3k** with similar and >86:14 enantiomeric ratios, with the strong electron-withdrawing nitro group resulting in the lowest enantioselectivity (**3d**) and the unsubstituted benzene ring resulting in the highest observed enantioselectivity (**3a**, 95:5 er). These results imply that the electronics of the aromatic ring do not have a pronounced effect on the enantioselectivity of the reaction. The *o*-Cl-substituted ylide resulted in similar enantioselectivity, albeit in lower yield (**3h**, 19% yield, 85:15 er). Naphthyl-substituted ylide furnished product **3i** in 91% yield and 91:9 er. Styryl-substituted product **3j** was obtained in good yield but lower enantioselectivity (79%, 70:30 er). This result indicates that the steric hindrance or the π-system of the aromatic ring plays an important role in the interaction between the substrate and fluorinating agent in the enantio-determining step. Using ylides with α-propyl substitutions, products **3l** and **3m** were obtained with enantioselectivities of up to 92:8 er. To evaluate the effect of substituents on the sulfur chiral center of the ylide, we synthesized ylide **1n**, which contains ethyl substituents on the nitrogen atom of the sulfoximine. When ylide **1n** was subjected to the optimal conditions for α,α-dihalogenation, compound **3a** was obtained in a reduced yield (60% vs 75%) and with a slightly lower er (94:6). These results indicate that a bulkier substituent on the nitrogen atom of the sulfoximine does not strongly influence the reaction selectivity. Exchanging **F3** with NBS, product **4a** was obtained in 51% yield and 96:4 er, showing that α,α-bromochlorination is also possible using this method. This result is particularly interesting, since it opens up the possibility of employing other combinations of electrophiles and nucleophiles in this reaction, possibly not limited to halogens. Although this reaction system is not catalytic, it has an advantage compared with classic chiral auxiliary reactions, since there is no need for an extra removal step. The absolute configuration of compounds **3** could not be determined since none of the synthesized compounds were solid and a crystal structure could not be obtained. Nevertheless, during our attempts at α,α-bromochlorination, we synthesized compound **4b** in 70% yield, which was isolated as a solid. The enantiomeric ratio of this compound could not be determined, as we were unable to separate the two enantiomers. A crystal of this compound was obtained, and single-crystal X-ray diffraction (SCXRD) analysis was performed. The crystal structure of one of the enantiomers of **4b** is displayed on Scheme 2 and in detail in Figures S2 and S3.

Next, we were able to perform an asymmetric first-step formation of a C–F bond using an *in situ* preformed cinchona alkaloid fluorinating agent (Shibata’s classic procedure).^42–44^ The results obtained with this strategy are displayed in Table S4 (see discussion and the table in the Supporting Information). Several alkaloids in combination with the fluorine source were studied, with (QD)_2_PHAL **Q3** and Selectfluor providing the best results (61%, 89:11 er; acetone at −23 °C). With these best conditions in hand, we began to evaluate the behavior of substituted ylides under the reaction conditions (Scheme 3). Strong and mild electron-releasing groups -OMe and -Me in the *para* position furnished α,α-chlorofluoro products in good yields and good to moderate enantioselectivities (**3c** and **3n**, respectively). On the other hand, strong electron-withdrawing group *p*-NO_2_ resulted in lower yield and enantioselectivity (**3d**, 30%, 73:27 er). Halogen substitution on the aromatic ring furnished products with 84:16 and 81:19 er (**3e** and **3f**, respectively). The 4-phenyl-substituted ring provided good enantioselection and yield (**3g**, 65%, 90:10 er). The 2-furyl derivative and *ortho*-substituted ylides were not good substrates for this reaction, delivering products in lower selectivities (**3h** and **3b**, respectively). The naphthyl substituent provided product **3i** in 51% yield and 79:21 er. To evaluate the robustness of our procedure, we performed the reaction on a 1.5 mmol scale. The reaction proceeded smoothly, providing product **3a** in 65% yield (181.1 mg), albeit with a slightly lower enantioselectivity (85:15 er vs 89:11 er). **Q3** was also recovered in 82% yield for the scaled-up reaction. We also evaluated a more diverse array of ylide structures, such as cyclic and acyclic esters and aryl–aryl-substituted keto ylide. We began evaluating aryl-ester ylides and found that for this class of substrates chiral amine **C1** furnished better enantioselectivity than **Q3**. Phenyl ester derivative **3′a** was prepared with 61:39 er in 48% yield. Other substituents on the ester moiety did not provide an improvement in enantioselectivity (61:39 er for **3′b**, 56:44 er for **3′c**). Cyclic ester ylide resulted in product **3′d** with 70:30 er. Aryl–aryl-substituted ylides resulted in products with selectivities in the same range (69:31 er for **3′e**, 71:29 er for **3′f**). Mild electron-releasing and strong electron-withdrawing substituents at the *para* position of the aromatic ring for phenyl esters did not drastically alter the enantioselectivity (67:33 er for **3′g**, 75:25 er for **3′h**). Lastly, the reaction for the formation of product **3′i**, bearing an acidic α-carbonyl hydrogen, resulted in a complex mixture of products. We also investigated the catalytic version from ylide **2a**, employing 10–20 mol % catalyst. However, the enantiomeric ratios were reduced as well as the yields.

**Scheme 3:**
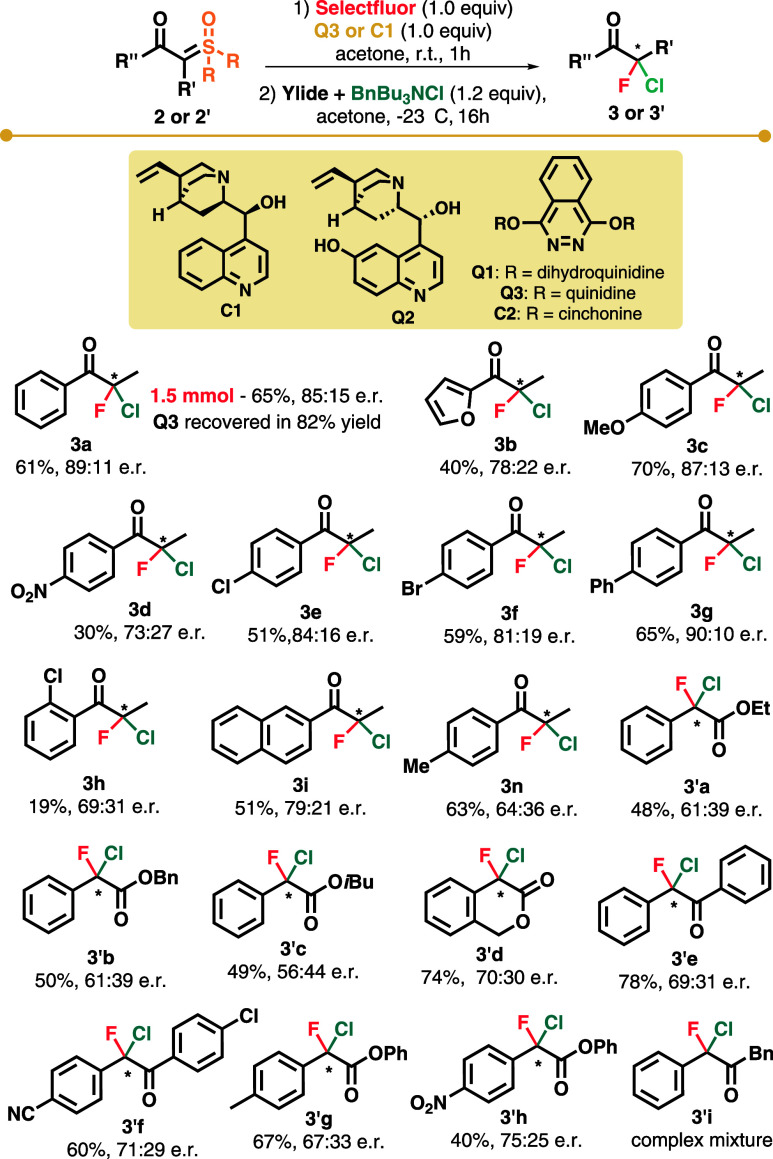
Substrate Scope for Asymmetric α,α-Chlorofluorination Promoted by **Q3** and **C1** ^a^

a Reaction conditions: **2** and **2′** (0.15 mmol), “F^+^” (0.15 mmol), chiral amine (0.15 mmol), BnBu_3_NCl (0.18 mmol), solvent (1.5 mL, 0.1 M), 16 h, −23 °C.

Lastly, additional tests regarding the interaction of the chiral aminosulfoxonium ylides with **Q3** were performed. The use of ylides **1a** and **Q3**, using Shibata’s chiral fluorinating agents, led to **3a** in 86% yield and 85:15 er. Nonetheless, the use of **ent-1a** led to **ent-3a** in 89% yield and 78:22 er, a significant decrease in enantioselectivity compared to the other conditions (Scheme 4A). Although the tests did not led to an improvement in enantioselectivity, they showcase the divergence in interactions of the chiral sulfoxonium ylides with **Q3**. Treatment of **3a** with an excess of Cl^–^ was performed to investigate if any stereoinversion would occur. After the elapsed time, no racemization was observed, indicating the formation of a stable stereocenter (Scheme 4B). It is worth noting that we evaluated several catalytic systems, including chiral organometallic complexes, chiral hydrogen bond donors, and chiral phase-transfer catalysts, for the α,α-dihalogenation of sulfur ylides. However, no enantioselectivity was observed, highlighting the challenging nature of this transformation. Further experiments can be performed to identify a suitable catalytic system for this reaction (see Tables S2 and S3 for full optimization conditions).

**Scheme 4:**
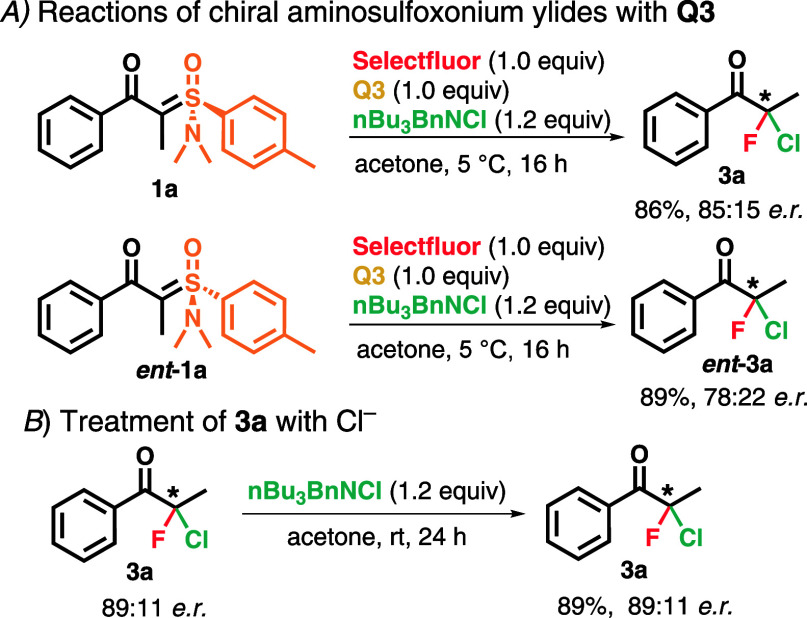
Additional Studies^a^

a (A) Chiral aminosulfoxonium ylides and Shibata’s chiral fluorinating reagents and (B) an excess of Cl^–^.

In summary, the first asymmetric one-step α,α-chlorofluorination of carbonyl compounds is presented. Combining two different strategies, 23 examples of α,α-chlorofluoro ketones or esters were prepared in an enantioenriched fashion. Two examples of α,α-bromochloroketones were also shown. Using the chiral amino-sulfoxonium strategy, 13 examples of α,α-chlorofluoro ketones were prepared with 19–90% yields and up to 95:5 er. Using the chiral fluorinating agent strategy, 19 examples of α,α-chlorofluoro carbonyl compounds were prepared in 19–80% yields and enantioselectivities of up to 90:10 er. Although the transformation is not catalytic, chiral promoter **Q3** could be recovered in 82% yield and reused without a loss of enantioselection in the next reaction. Moreover, several different skeletons were evaluated, providing a more diverse study of α,α-chlorofluorination of carbonyl compounds. Comparing the products that were obtained by both strategies, it is clear that chiral aminosulfoxonium ylides proved to be superior, providing products in higher enantiomeric ratios (and similar or higher yields in every case). Furthermore, this is the first example in which Johnson’s aminosulfoxonium ylides have been used to obtain products with high enantiomeric excesses, underscoring the limited studies on this important class of sulfur ylides. We believe that this strategy can serve as a starting point for the development of other asymmetric difunctionalizations of aminosulfoxonium ylides using different combinations of electrophilic and nucleophilic heteroatoms, not limited to halogens. We believe that for both strategies, the mechanism is similar to that proposed for the racemic reaction,^35^ with the initial attack of the sulfoxonium ylide on the electrophilic fluorine source generating the chiral carbon, which is then attacked by the chloride, with displacement of DMSO via an S_N_2 reaction. This hypothesis would explain the dramatic effect of the structure of the “F^+^” source on the observed er.

## Supplementary Material



## Data Availability

The data underlying this study are available in the published article and its Supporting Information.

## Notes

Detailed experimental procedures, characterization of new compounds, copies of NMR spectra, and SCXRD data for compounds **1h** and **4b** (PDF)
