# Supplementary material for: Enantioselective α,α-Chlorofluorination of Sulfoxonium Ylides
Source: Org Lett. 2026 Jan 28;28(5):1616–21. doi: 10.1021/acs.orglett.5c05062 (PMC12887995; doi:10.1021/acs.orglett.5c05062)
Supplement: Supplementary file 1 [file ol5c05062_si_001.pdf]

SUPPORTING INFORMATION

Enantioselective α,α-Chlorofluorination of Sulfoxonium Ylides

Lucas G. Furniel,[a] Kauê C. Capellaro,[a] Viktor S. Câmara,[a] Marcio Hayashi,[a] Radell Echemendía,[a] Camila B. Pinto,[b] Ana B. A. M. Salata,[c] Jackson A. L. Filho,[c] Leandro W. Hantao,[c] Javier Ellena,[b] Antonio C. B. Burtoloso*[a]

[a] Chemistry Institute of São Carlos, University of São Paulo, CEP 13560–970, São Carlos, SP, (Brazil) E–mail: antonio@iqsc.usp.br

[b] São Carlos Institute of Physics, University of São Paulo, São Carlos, SP CEP 13560-970, Brazil

[c] Chemistry Institute, University of Campinas, Campinas, CEP 13083-862, Brazil

TABLE OF CONTENTS

1. MATERIALS AND METHODS 3
2. PREPARATION OF YLIDES 4
2.1 PREPARATION OF AMINOSULFOXONIUM YLIDES 4
2.1.1. GENERAL SCHEME OF THE AMINOSULFOXONIUM YLIDES SYNTHESIS 4
2.1.2. SYNTHESIS OF RACEMIC AND CHIRAL AMINOSULFOXONIUM YLIDES 9
2.2 PREPARATION OF PRO-CHIRAL SULFOXONIUM YLIDES 18
2.2.1. SYNTHESIS OF SULFOXONIUM YLIDES VIA DIAZO COMPOUNDS: 24
3. OPTIMIZATION STUDIES 27
3.1. CHIRAL AMINOSULFOXONIUM YLIDES 27
3.2. CATALYTIC STUDIES 28
3.3. PRO-CHIRAL SULFOXONIUM YLIDES 30
4. DIHALOGENATION REACTIONS 32
4.1 DIHALOGENATION PROCEDURE: PRO-CHIRAL SULFOXONIUM YLIDES 32
4.2 DIHALOGENATION PROCEDURE: CHIRAL AMINOSULFOXONIUM YLIDES 33
5. CHARACTERIZATION OF PRODUCTS 33
5.1 ALFA-ALKYL CARBONYL DIHALOGENATION PRODUCTS 33
6. SINGLE CRYSTAL X-RAY DIFFRACTION (SCXRD) 50
7. CHROMATOGRAMS OF RACEMIC AND ENANTIOENRICHED COMPOUNDS 54
8. ^{1}H, $^{13}C\{H\}$ AND ^{19}F NMR SPECTRA 102
9. REFERENCES 176

1. MATERIALS AND METHODS

Reagents purchased commercially were used without further purification unless otherwise noted. Solvents were dried and distilled before use, following standard protocols. All reactions were stirred magnetically and performed in flame- or oven-dried glassware under argon, unless indicated otherwise. The cooling of reactions was performed by using an ice and water bath. Reactions were monitored using thin-layer chromatography (TLC) on Merck silica gel 60 F254 precoated plates (0.25 mm). Flash column chromatography was performed using silica gel 60 (particle size 0.063−0.210 mm). Reported yields refer to isolated products after column chromatography. ^{1}H NMR spectra were recorded on 400 (Agilent Technologies, 400/54 Premium Shielded) or 500 MHz (Agilent Technologies, 500/54 Premium Shielded) spectrometers, and the chemical shifts (δ) are reported in parts per million relative to tetramethylsilane as an internal standard or residual solvents from $CDCl_3$ (7.26 ppm). ^{13}C NMR spectra were recorded on 100 MHz or 125 MHz spectrometers, with chemical shifts (δ) referenced to $CDCl_3$ (77.0 ppm). ^{19}F NMR spectra were recorded on 376 MHz and 470 MHz spectrometers. Infrared spectra were obtained using Fourier-transform infrared spectroscopy (FT-IR) at a resolution of 4.0 cm^{-1} (Bruker, model ALPHA) and are reported in wavenumbers (cm-1). Melting points were determined using a digital melting point apparatus (Fisatom, model 430D), and the values were uncorrected. High-resolution electrospray ionization mass spectra (ESI-HRMS) were recorded using either a micrOTOF-Q II instrument (Bruker Daltonics, Karlsruhe, Germany) or a Waters Xevo G2-XS QToF instrument (Waters, Milford, USA), both operating in positive mode with a time-of-flight (TOF) analyzer. For some samples, Gas-Chromatography coupled to a Mass Spectrometry (GC-MS) was used. In this case, these analyses were performed with Shimadzu GC2010 Plus gas chromatography coupled to mass-selective detector Shimadzu MS2010 Plus in an electron ionization mode (70 eV). Enantiomeric excess was determined by: high-performance liquid chromatography (HPLC) - LC-20A Prominence Shimadzu and columns packed with a chiral stationary phase (CSP); Supercritical Fluid Chromatograph ACQUITY UPC2 Mass Spectrometer Waters Xevo TQD and columns packed with a chiral stationary phase (CSP)

2. PREPARATION OF YLIDES

2.1 PREPARATION OF AMINOSULFOXONIUM YLIDES

2.1.1. GENERAL SCHEME OF THE AMINOSULFOXONIUM YLIDES SYNTHESIS

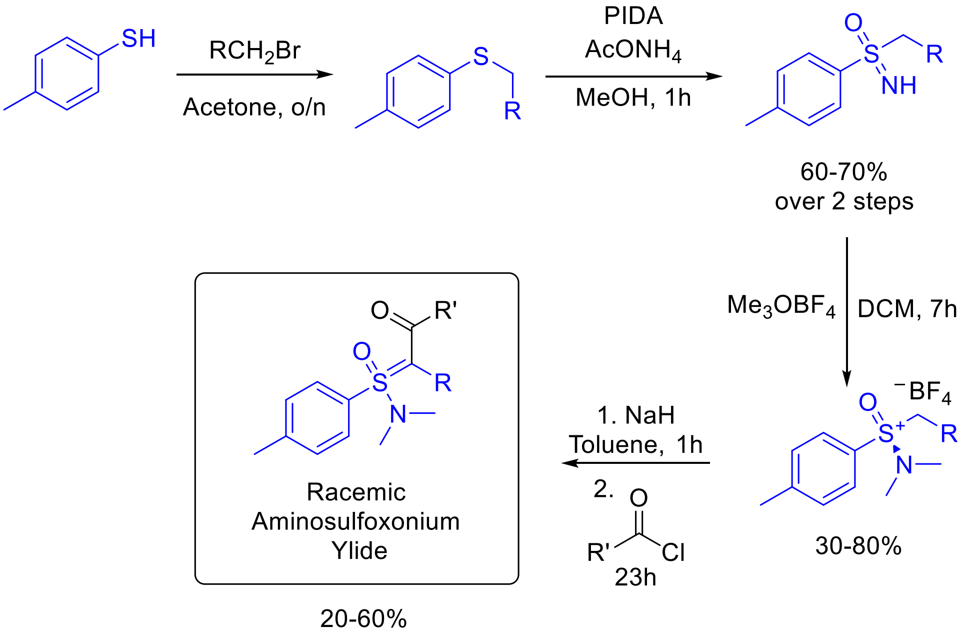

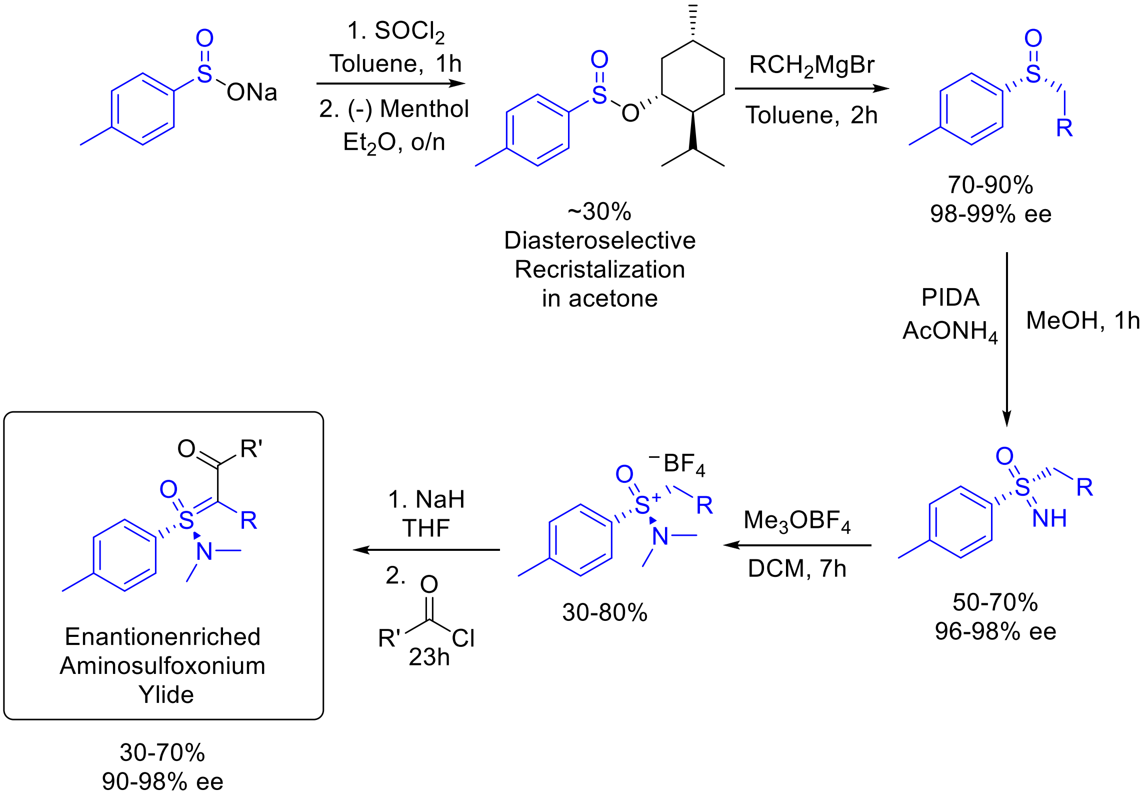

- *(R)*-1-(ethylsulfinyl)-4-methylbenzene (**SM-A1**)

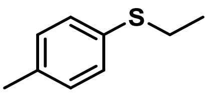

Prepared according to the procedure reported in the literature.[1]

Yellow oil. Yield: 82% (1.246 g).

^{1}H NMR (400 MHz, $CDCl_3$) δ 7.25 (d, J = 8.2 Hz, 2H), 7.13 – 7.05 (m, 2H), 2.90 (d, J = 7.3 Hz, 2H), 2.32 (s, 3H), 1.28 (t, J = 7.4 Hz, 3H).

^{13}C{H} NMR (100 MHz, $CDCl_3$) δ 135.9, 132.7, 130.0, 129.6, 28.4, 21.0, 14.5.

The spectroscopic data are in agreement with those reported in the literature.[1]

- (R)-1-(butylsulfinyl)-4-methylbenzene (**SM-A2**)

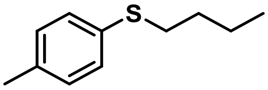

Prepared according to the procedure reported in the literature.[1]

Yellow oil. Yield: 78% (1.404 g).

^{1}H NMR (500 MHz, $CDCl_3$) δ 7.27 (d, J = 8.2 Hz, 2H), 7.11 (d, J = 7.9 Hz, 2H), 2.97 – 2.85 (m, 2H), 2.34 (s, 3H), 1.63 (p, J = 7.4 Hz, 2H), 1.46 (dq, J = 14.5, 7.3 Hz, 2H), 0.93 (t, J = 7.3 Hz, 3H).

^{13}C{H} NMR (125 MHz, $CDCl_3$) δ 135.8, 133.2, 129.8, 129.6, 128.6, 34.0, 31.3, 21.9, 21.0, 13.6.

The spectroscopic data are in agreement with those reported in the literature.[2]

- ((1R,2S,5R)-2-isopropyl-5-methylcyclohexyl 4-methylbenzenesulfinate (**SM-B0**)

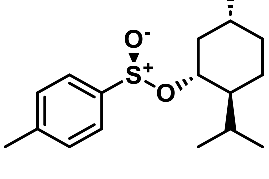

Prepared according to the procedure described in the literature.[3]

Yield: 36% (10.76 g, after 3 recrystallization steps). White crystalline solid.

^{1}H NMR (500 MHz, $CDCl_3$) δ 7.60 (d, J = 8.2 Hz, 2H), 7.32 (d, J = 7.9 Hz, 2H), 4.12 (td, J = 10.8, 4.5 Hz, 1H), 2.41 (s, 3H), 2.33 – 2.25 (m, 1H), 2.16 – 2.09 (m, 1H), 1.73– 1.62 (m, 2H), 1.55 – 1.44 (m, 1H), 1.35 (m, 1H), 1.22 (m, 1H), 1.09 – 0.99 (m,1H), 0.96 (d, J = 6.6 Hz, 3H), 0.92-0.80 (m, 4H), 0.72 (d, J = 6.9 Hz, 3H).

MP = 107-108°C.

[α]D[22] = -200 (c = 1.01, acetone).

The spectroscopic data are in agreement with those reported in the literature.[3]

- *(R)*-1-(ethylsulfinyl)-4-methylbenzene (**SM-B1**)

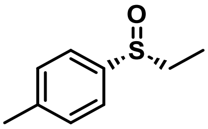

Prepared according to the procedure reported in the literature.[4]

Yellow oil. Yield: 93% (1.788 g).

^{1}H NMR (500 MHz, $CDCl_3$) δ 7.52 – 7.47 (m, 2H), 7.35 – 7.29 (m, 2H), 2.87 (dq, J = 13.2, 7.4 Hz, 1H), 2.76 (dq, J = 13.2, 7.4 Hz, 1H), 2.41 (s, 3H), 1.18 (t, J = 7.4 Hz, 3H).

^{13}C{H} NMR (125 MHz, $CDCl_3$) δ 141.4, 140.1, 129.9, 124.3, 50.4, 21.5, 6.11.

IR ν max (cm^{-1}): 1494, 1453, 1086, 1042, 1013, 811.

TLC: R_f = 0.52 (AcOEt).

$[\alpha]_D^{24}$ = +194 (c = 1.02, $CHCl_3$) for 98% *e.e.*

HPLC (AD-H, n-hexane/*i*-PrOH = 90/10, flow rate = 1.0 mL/min, l = 254 nm) t_R = 8.993 min (minor), t_R = 9.823 min (major), *e.r.* = 99:1.

The spectroscopic data are in agreement with those reported in the literature.[1]

- *(R)*-1-(butylsulfinyl)-4-methylbenzene (**SM-B2**)

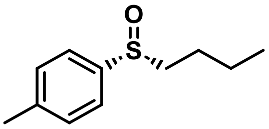

Prepared according to the procedure reported in the literature.[4]

Yellow oil. Yield: 78% (306 mg).

^{1}H NMR (500 MHz, $CDCl_3$) δ 7.55 – 7.47 (m, 2H), 7.36 – 7.28 (m, 2H), 2.85 – 2.69 (m, 2H), 2.42 (s, 3H), 1.79 – 1.32 (m, 5H), 0.92 (t, J = 7.3 Hz, 3H).

^{13}C{H} NMR (125 MHz, $CDCl_3$) δ 143.8, 139.0, 129.8, 128.4, 64.4, 57.4, 25.4, 25.1, 21.5, 21.5, 13.5.

- (R)-ethyl(imino)(p-tolyl)-λ^6-sulfanone **(SM-C1)**

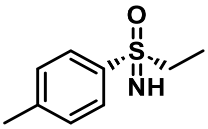

Prepared according to the procedure reported in the literature.[5]

Yellow oil. Yield: 80% (40.5 mg).

^{1}H NMR (500 MHz, $CDCl_3$) δ 7.88 – 7.80 (m, 2H), 7.38 – 7.32 (m, 2H), 3.22 – 3.11 (m, 2H), 2.45 (s, 3H), 1.25 (t, J = 7.4 Hz, 3H).

^{13}C{H} NMR (125 MHz, $CDCl_3$) δ 144.0, 138.3, 129.8, 128.6, 51.9, 21.6, 8.0.

IR ν max (cm^{-1}): 3269, 2977, 2936, 1596, 1453, 1204, 1094, 1044, 967, 816, 714, 692.

TLC: R_f = 0.34 (AcOEt);

$[\alpha]_D^{24}$ = -21,5 (c = 0.97, acetone) for 96% *e.e.*

HPLC (AD-H, n-hexane/*i*-PrOH = 90/10, flow rate = 1.0 mL/min, l = 254 nm) t_R = 11.674 min (minor), t_R = 12.601 min (major), *e.r.* = 2:98.

- R)-butyl(imino)(p-tolyl)-λ^6-sulfanone (**SM-C2**)

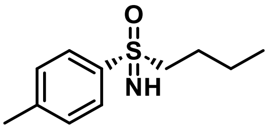

Prepared according to the procedure reported in the literature.[5]

Yellow oil. Yield: 66% (139 mg).

^{1}H NMR (400 MHz, $CDCl_3$) δ 7.87 – 7.78 (m, 2H), 7.37 – 7.29 (m, 2H), 3.12 (ddd, J = 9.7, 6.1, 3.1 Hz, 2H), 2.44 (s, 3H), 1.79 – 1.55 (m, 2H), 1.35 (h, J = 7.4 Hz, 2H), 0.87 (t, J = 7.3 Hz, 3H).

^{13}C{H} NMR (100 MHz, $CDCl_3$) δ 143.8, 139.0, 129.8, 128.4, 64.4, 57.4, 25.4, 25.1, 21.5, 21.5, 13.5.

IR ν max (cm^{-1}): 3550, 3530, 3509, 3473, 3455, 3425,3408, 3384, 3273, 3060, 2960, 2932, 2873, 1630, 1597, 1491, 1463, 1405, 1381, 1278, 1216,1103,1022, 981, 917, 817, 758, 731,711, 647, 624.

TLC: R_f = 0.4 (AcOEt);

$[\alpha]_D^{24}$ = +3 (c = 0.8, $CHCl_3$) for 98% *e.e.*

HPLC (AD-H, n-hexane/*i*-PrOH = 90/10, flow rate = 0.7 mL/min, l = 254 nm) t_R = 22.747 min (minor), t_R = 24.020 min (major), *e.r.* = 1:99.

HRMS (ESI) *m/z*: $[M+Na]^+$ Calcd. for $C_{11}H_{18}NOS+Na^+$ 212.1104 ; Found: 212.1103.

- (*R*)-tetrafluoroborate of ethyl *p*-tolyl(dimethylamino)oxosulfoxonium (**SM-D1**)

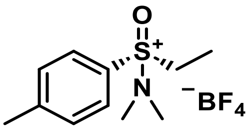

Prepared according to the procedure reported in the literature.[6]

Pale yellow solid (Enantioenriched), or off-white solid (Racemic). Yield: 79% (1.304 g).

^{1}H NMR (500 MHz, $CDCl_3$) δ 8.07 – 7.99 (m, 2H), 7.67 – 7.59 (m, 2H), 4.34 – 4.20 (m, 2H), 3.14 (s, 6H), 2.55 (s, 3H), 1.35 (t, J = 7.3 Hz, 3H).

^{13}C{H} NMR (125 MHz, $CDCl_3$) δ 149.6, 132.0, 129.8, 122.7, 47.4, 38.1, 22.0, 6.5.

IR ν max (cm^{-1}): 3635, 2990, 2945, 1592, 1455, 1408,1270, 1221, 1059, 951, 819, 787, 746, 716.

HRMS (ESI) *m/z*: $[M]^+$ Calcd. for $C_{11}H_{19}NOS^{2+}$ 212.1104; Found: 212.1104.

- (*R*)-tetrafluoroborate of butyl *p*-tolyl(dimethylamino) oxosulfoxonium (**SM-D2**)

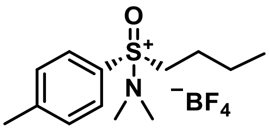

Prepared according to the procedure reported in the literature.[6]

Pale yellow solid (Enantioenriched), or off-white solid (Racemic). Yield: 50% (442 mg).

^{1}H NMR (400 MHz, $CDCl_3$) δ 8.05 – 7.91 (m, 2H), 7.63 – 7.55 (m, 2H), 4.25 – 4.07 (m, 2H), 3.09 (s, 6H), 2.51 (s, 3H), 1.78 – 1.62 (m, 1H), 1.57 – 1.23 (m, 3H), 0.84 (t, J = 7.2 Hz, 3H).

^{13}C{H} NMR (100 MHz, $CDCl_3$) δ 149.5, 132.0, 129.7, 123.3, 51.3, 38.1, 24.0, 21.9, 20.9.

IR ν max (cm^{-1}): 3633, 2964, 2935, 2877, 1592, 1459,1406, 1265, 1224, 1049, 948, 818, 734, 723.

HRMS (ESI) *m/z*: $[M]^+$ Calcd. for $C_{13}H_{22}NOS^+$ 240.1417 ; Found: 240.1414.

- (*R*)-tetrafluoroborate of ethyl *p*-tolyl(diethylamino)oxosulfoxonium (**SM-D3**)

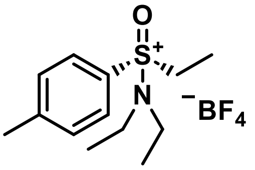

Prepared according to the procedure reported in the literature.[6]

Off white solid. Yield: 90% (1.001 g).

^{1}H NMR (500 MHz, $CDCl_3$) δ 8.08 – 7.99 (m, 2H), 7.66 – 7.58 (m, 2H), 4.58 – 4.09 (m, 2H), 3.76 – 3.36 (m, 4H), 2.55 (s, 3H), 1.32 (td, J = 7.2, 5.3 Hz, 9H).

^{13}C{H} NMR (126 MHz, $CDCl_3$) δ 149.4, 131.9, 131.9, 129.8, 123.7, 47.4, 43.4, 22.0, 13.2, 6.6.

IR ν max (cm^{-1}): 2989, 2945, 2883, 1592, 1454, 1392,1254, 1187, 1057, 944, 818, 790, 727, 696.

HRMS (ESI) *m/z*: $[M]^+$ Calcd. for $C_{13}H_{22}NOS^+$ 240.1417 ; Found: 240.1414.

2.1.2. SYNTHESIS OF RACEMIC AND CHIRAL AMINOSULFOXONIUM YLIDES

The procedure used was an adaptation of the method of Johnson et al. (1970).[7] First, NaH (80.2 mg, 2.0 mmol, 3.0 eq, 60% in mineral oil) was added to a previously dried 25 mL flask purged with argon. Then, 5.0 mL of anhydrous THF was added, and stirring was started using a magnetic stir bar. To this suspension of the hydride in THF was added (*R*)-ethyl *p*-toluyl(dimethylamino)oxosulfoxonium tetrafluoroborate (200.0 mg, 0.67 mmol, 1.0 eq), and stirring was continued for 1 h at room temperature. Afterwards, the system was cooled in an ice bath and an ice-cold solution of the respective benzoyl chloride (1.0 eq) in 5 mL of anhydrous THF was slowly added. After the addition, the reaction was maintained in an ice bath for 2 h, and then at rt for another 20 h. After this time, the reaction mixture was filtered, the precipitate washed with 30 mL of $CHCl_3$, and the residue concentrated by evaporation. The residue was then purified by a flash column on silica (70%-100% AcOEt/Hex), providing the respective pure aminosulfoxonium ylide.

- *N*,*N,*4-trimethyl-*S*-(1-oxo-1-phenylpropan-2-ylidene)benzenesulfinamide (**1a**)

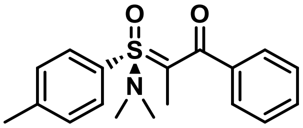

Yellow oil. Yield: 72% (159.5 mg).

^{1}H NMR (500 MHz, CDCl$_3$) δ 8.03 (d, J = 6.8 Hz, 2H), 7.51 – 7.39 (m, 2H), 7.31 (m, 5H), 2.89 (s, 6H), 2.41 (s, 3H), 2.04 (s, 3H).

^{13}C{H} NMR (125 MHz, CDCl$_3$) δ 185.1, 144.2, 142.7, 133.6, 129.7, 128.9, 128.8, 127.8, 127.5, 72.3, 36.9, 21.7, 13.4.

IR ν max (cm^{-1}): 3057, 2926, 1540, 1359, 1186, 988, 933, 813, 720, 701, 690, 639.

TLC: Rf = 0.21 (AcOEt).

$[\alpha]_D^{22}$ = - 56.20 (c = 2.79, CHCl$_3$) for 96% *e.e.*

HRMS (ESI) *m/z*: [M+H]$^+$ Calcd. for $C_{18}H_{22}NO_2S^+$ 316.13658; Found: 316.13837.

HPLC (AD-H, n-hexane/*i*-PrOH = 80/20, flow rate = 1.0 mL/min, l = 220 nm) t_R = 8.258 min (minor), t_R = 15.099 min (major), *e.r.* = 2:98.

- *S*-(1-(furan-2-yl)-1-oxopropan-2-ylidene)-*N,N*,4 trimethylbenzenesulfinamide (**1b**)

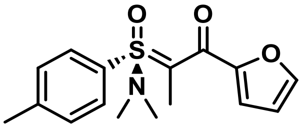

Brown oil. Yield: 76% (155.5 mg).

^{1}H NMR (500 MHz, CDCl$_3$) δ 8.06 – 8.00 (m, 2H), 7.43 (dd, J = 1.7, 0.8 Hz, 1H), 7.34 – 7.28 (m, 2H), 6.84 (dd, J = 3.5, 0.8 Hz, 1H), 6.41 (dd, J = 3.4, 1.7 Hz, 1H), 2.88 (s, 6H), 2.41 (s, 3H), 2.28 (s, 3H).

^{13}C{H} NMR (125 MHz, CDCl$_3$) δ 171.0, 154.3, 144.1, 144.1, 142.9, 142.9, 133.6, 129.5, 128.9, 113.0, 110.8, 73.0, 53.4, 36.8, 21.5, 11.2.

IR ν max (cm^{-1}): 3436, 2926, 2871, 1575, 1531, 1471, 1392, 1355, 1189, 1156, 1082, 1014, 996, 936, 913, 806, 776, 752, 712, 673.

TLC: Rf = 0.23 (AcOEt).

$[\alpha]_D^{22}$ = - 105.3 (c = 0.55, CHCl$_3$) for 92% *e.e.*

HRMS (ESI) *m/z*: [M+H]$^+$ Calcd. for $C_{16}H_{20}NO_3S^+$ 306.1158 ; Found: 306.1156.

HPLC (AD-H, n-hexane/*i*-PrOH = 80/20, flow rate = 1.0 mL/min, l = 220 nm) t_R = 6.970 min (minor), t_R = 10.212 min (major), *e.r.* = 4:96.

- *S*-(1-(4-methoxyphenyl)-1-oxopropan-2-ylidene)-*N,N*,4-trimethylbenzenesulfinamide (**1c**)

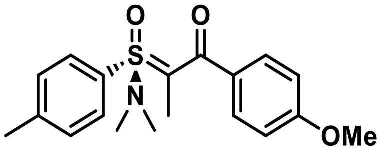

Yellow oil. Yield: 82% (190.68 mg).

^{1}H NMR (500 MHz, CDCl$_3$) δ 8.00 (d, J = 8.3 Hz, 2H), 7.47 (d, J = 8.8 Hz, 2H), 7.31 (d, J = 8.1 Hz, 2H), 6.82 (d, J = 8.8 Hz, 2H), 3.80 (s, 3H), 2.87 (s, 6H), 2.41 (s, 3H), 2.08 (s, 3H).

^{13}C{H} NMR (125 MHz, CDCl$_3$) δ 184.7, 160.3, 144.0, 135.0, 133.5, 129.6, 129.5, 128.8, 113.0, 71.7, 55.3, 36.9, 21.6, 13.6.

IR ν max (cm^{-1}): 2932, 1605, 1535, 1509, 1462, 1357, 1249, 1183, 1167, 988, 933, 777.

TLC: Rf = 0.13 (AcOEt).

$[\alpha]_D^{23}$ = - 40.8 (c = 0.63 CHCl$_3$) for 96% *e.e.*

HRMS (ESI) *m/z*: $[M+H]^+$ Calcd. for $C_{19}H_{24}NO_3S^+$ 346.1471; Found: 346.1479.

HPLC (PHENOMENEX AMYLOSE 2, n-hexane/*i*-PrOH = 70/30, flow rate = 1.0 mL/min, l = 220 nm) t_R = 17.826 min (minor), t_R = 26.217 min (major), *e.r.* = 2:98.

- *N,N*,4-trimethyl-*S*-(1-(4-nitrophenyl)-1-oxopropan-2-ylidene)benzenesulfinamide (**1d**)

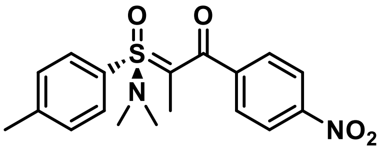

Yellow oil. Yield: 78% (188.4 mg).

Column chromatography eluent: AcOEt: MeOH 90:10.

^{1}H NMR (500 MHz, CDCl$_3$) δ 8.23 – 8.11 (m, 2H), 8.06 (s, 2H), 7.58 – 7.49 (m, 2H), 7.40 – 7.33 (m, 2H), 2.92 (s, 6H), 2.44 (s, 3H), 1.98 (s, 3H).

^{13}C{H} NMR (125 MHz, CDCl$_3$) δ 181.4, 144.8, 129.8, 128.6, 128.1, 123.2, 67.9, 53.4, 36.7, 25.6, 21.6, 12.9.

IR ν max (cm^{-1}): 2925, 1596, 1518, 1456, 1402, 1343,1315, 1291, 1270, 1186, 1104, 1081,1058, 991, 934, 858, 813, 725, 707,657.

TLC: Rf = 0.34 (AcOEt).

$[\alpha]_D^{22}$ = 25.9 (c = 0.29, $CHCl_3$) for 94% *e.e.*

HRMS (ESI) *m/z*: $[M+H]^+$ Calcd. for $C_{18}H_{21}N_2O_4S^+$ 361.1217; Found: 361.1222.

HPLC (AD-H, n-hexane/*i*-PrOH = 80/20, flow rate = 1.0 mL/min, l = 220 nm) t_R = 28.346 min (major), t_R = 34.354 min (minor), *e.r.* = 3:97.

- *S*-(1-(4-chlorophenyl)-1-oxopropan-2-ylidene)-*N,N*,4-trimethylbenzenesulfinamide (**1e**)

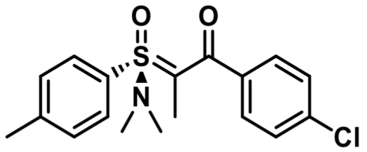

Yellow oil. Yield: 78% (183.8 mg).

^{1}H NMR (500 MHz, $CDCl_3$) δ 8.01 (m, 2H), 7.39 (d, J = 8.4 Hz, 2H), 7.33 (d, J = 8.1Hz, 2H), 7.29 – 7.25 (m, 2H), 2.88 (s, 6H), 2.42 (s, 3H), 2.02 (s, 3H).

^{13}C{H} NMR (125 MHz, $CDCl_3$) δ 183.6, 144.4, 141.1, 134.7, 133.3, 129.7, 129.0, 128.7,128.0, 72.8, 36.8, 21.7, 13.4.

IR ν max (cm^{-1}): 2926 1591, 1574, 1534, 1360, 1184, 1085, 988, 932, 842, 812, 751,734, 705, 666, 656.

TLC: Rf = 0.50 (AcOEt).

$[\alpha]_D^{23}$ = - 48.5 (c = 3.35, $CHCl_3$) for 98% *e.e.*

HRMS (ESI) *m/z*: $[M+H]^+$ Calcd. for $C_{18}H_{21}ClNO_2S^+$ 350.0976; Found: 350.0979.

HPLC (AD-H, n-hexane/*i*-PrOH = 70/30, flow rate = 1.0 mL/min, l = 220 nm) t_R = 8.133 min (minor), t_R = 18.298 min (major), *e.r.* = 1:99.

S-(1-(4-bromophenyl)-1-oxopropan-2-ylidene)-*N,N*,4-trimethylbenzenesulfinamide (**1f**)

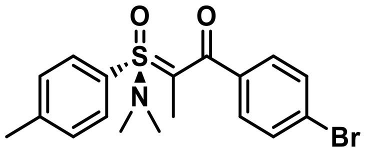

Yellow oil. Yield: 63% (166.4 mg).

Column chromatography eluent: AcOEt: MeOH 90:10.

^{1}H NMR (500 MHz, CDCl$_3$) δ 8.02 – 7.92 (m, 2H), 7.43 – 7.37 (m, 2H), 7.34 – 7.27 (m, 4H), 2.85 (s, 6H), 2.39 (s, 3H), 1.98 (s, 3H).

^{13}C{H} NMR (125 MHz, CDCl$_3$) δ 183.5 ,144.3, 141.5, 133.2, 130.8, 129.6, 129.2, 128.6, 122.8, 72.7, 36.7, 21.5, 13.2.

IR ν max (cm^{-1}): 2954, 2924, 2853, 1735, 1573, 1527, 1461, 1365, 1192, 1083, 1068, 990, 938, 841, 813, 748, 726, 657.

TLC: Rf = 0.45 (AcOEt).

$[\alpha]_D^{22}$ = - 15.8 (c = 1.65, CHCl$_3$) for 90% *e.e.*

HRMS (ESI) *m/z*: $[M+H]^+$ Calcd. for $C_{18}H_{21}BrNO_2S^+$ 394.0471; Found: 394.0464.

HPLC (AD-H, n-hexane/*i*-PrOH = 80/20, flow rate = 1.0 mL/min, l = 220 nm) t_R = 13.406 min (minor), t_R = 38.653 min (major), *e.r.* = 5:95.

- *(R)*- S-(1-(4-(tert-butyl)phenyl)-1-oxopropan-2-ylidene)-*N,N*,4-trimethylbenzenesulfinamide (**1g**)

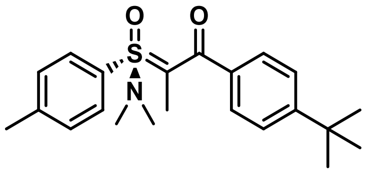

Yellow oil. Yield: 67% (166.8 mg).

^{1}H NMR (500 MHz, CDCl$_3$) δ 8.01 (d, J = 8.0 Hz, 2H), 7.42 (d, J = 8.4 Hz, 2H), 7.34 – 7.28 (m, 4H), 2.88 (s, 6H), 2.40 (s, 3H), 1.29 (s, 9H).

^{13}C{H} NMR (125 MHz, CDCl$_3$) δ 183.5, 152.0, 144.0, 139.5, 133.5, 129.5, 128.7, 127.4, 124.6, 72.0, 36.8, 34.7, 31.3, 21.5, 13.4.

IR ν max (cm^{-1}): 2959, 2927, 2869, 1646, 1572, 1537,1461, 1401, 1385, 1362, 1191, 1183,1118, 1106, 1082, 989, 935, 847,812, 735, 707,669.

TLC: Rf = 0.48 (AcOEt).

$[\alpha]_D^{22}$ = - 36.9 (c = 1.1, CHCl$_3$) for 90% *e.e.*

HRMS (ESI) *m/z*: $[M+H]^+$ Calcd. for $C_{22}H_{30}NO_2S^+$ 372.1992; Found: 372.1986.

HPLC (AD-H, n-hexane/*i*-PrOH = 80/20, flow rate = 1.0 mL/min, l = 220 nm) t_R = 8.035 min (minor), t_R = 18.019 min (major), *e.r.* = 5:95.

- *(R)*-(1-([1,1'-biphenyl]-4-yl)-1-oxopropan-2-ylidene)-*N,N*,4-trimethylbenzenesulfinamide (**1h**)

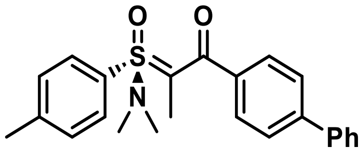

White wax. Yield: 35% (91.8 mg).

^{1}H NMR (500 MHz, $CDCl_3$) δ 8.04 (d, J = 7.7 Hz, 2H), 7.61 – 7.56 (m, 2H), 7.54 (s, 4H), 7.42 (t, J = 7.5 Hz, 2H), 7.38 – 7.30 (m, 3H), 2.90 (s, 6H), 2.42 (s, 3H), 2.10 (s, 3H).

^{13}C{H} NMR (125 MHz, $CDCl_3$) δ 184.6, 144.1, 141.6, 140.8, 129.5, 128.7, 128.0, 127.3, 127.1, 126.5, 72.4, 36.8, 21.5, 13.4.

IR ν max (cm^{-1}): 3055, 3029, 2927, 1564, 1529, 1448, 1355, 1179, 1119, 1080, 1046, 987, 932, 851, 812, 776, 746, 699, 679, 633.

TLC: Rf = 0.45 (AcOEt).

$[\alpha]_D^{22}$ = - 9 (c = 0.7, $CHCl_3$) for 96% *e.e.*

HRMS (ESI) *m/z*: $[M+H]^+$ Calcd. for $C_{24}H_{26}NO_2S^+$ 392.1679; Found: 392.1671.

HPLC (AD-H, n-hexane/*i*-PrOH = 90/10, flow rate = 0.7 mL/min, l = 220 nm) t_R = 8.490 min (minor), t_R = 9.234 min (major), *e.r.* = 2:98.

- *(R)*- *N,N*,4-trimethyl-S-(1-(naphthalen-2-yl)-1-oxopropan-2-ylidene)benzenesulfinamide (**1i**)

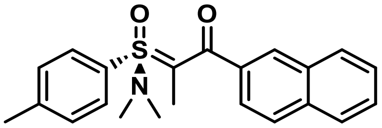

Colorless oil. Yield: 53% (130.0 mg).

^{1}H NMR (500 MHz, $CDCl_3$) δ 8.06 (d, J = 7.5 Hz, 2H), 7.93 (s, 1H), 7.85 – 7.76 (m, 3H), 7.58 (dd, J = 8.4, 1.6 Hz, 1H), 7.50 – 7.41 (m, 2H), 7.33 (d, J = 8.2 Hz, 2H), 2.92 (s, 6H), 2.41 (s, 3H), 2.10 (s, 3H).

^{13}C{H} NMR (125 MHz, $CDCl_3$) δ 184.9, 144.2, 140.0, 133.5, 132.8, 129.6, 129.6, 128.7, 128.5, 127.6, 127.4, 126.9, 126.3, 126.0, 125.4, 72.6, 36.8, 21.5, 13.4.

IR ν max (cm^{-1}): 3443, 3054, 2926, 2891, 1576, 1533, 1469, 1388, 1354, 1191, 1165, 1082, 1019, 997, 938, 802, 778, 757, 710, 680.

TLC: Rf = 0.45 (AcOEt).

[α]$_D^{22}$ = - 11.3 (c = 0.9, $CHCl_3$) for 90% *e.e.*

HRMS (ESI) *m/z*: $[M+H]^+$ Calcd. for $C_{22}H_{24}NO_2S^+$ 366.1522; Found: 366.1519.

HPLC (AD-H, n-hexane/*i*-PrOH = 90/10, flow rate = 0.7 mL/min, l = 220 nm) t_R = 8.478 min (minor), t_R = 9.233 min (major), *e.r.* = 5:95.

- *S*-(1-(2-chlorophenyl)-1-oxopropan-2-ylidene)-*N,N*,4-trimethylbenzenesulfinamide (**1j**)

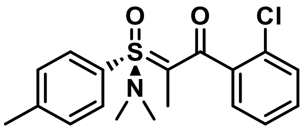

Yellow oil. Yield: 91% (212.8 mg)

^{1}H NMR (500 MHz, $CDCl_3$) δ 8.11 (d, J = 8.4 Hz, 1H), 7.35 (d, J = 8.1 Hz, 1H), 7.33 –7.30 (m, 1H), 7.23 – 7.10 (m, 2H), 2.97 (s, 3H), 2.44 (s, 2H), 1.81 (s, 2H).

^{13}C{H} NMR (125 MHz, $CDCl_3$) δ 182.0, 144.5, 142.5, 133.6, 130.3, 129.7, 129.4, 128.8, 128.7, 128.1, 126.8, 73.7, 36.9, 21.7, 12.1.

IR ν max (cm-1): 2925, 1591, 1546, 1456, 1432, 1383, 1364, 1191, 1083, 1056, 988, 935, 765, 744, 722, 706, 678.

TLC: Rf = 0.43 (AcOEt).

[α]$_D^{23}$ = - 55.4 (c = 3.27 $CHCl_3$) for 96% *e.e.*

HRMS (ESI) *m/z*: $[M+H]^+$ Calcd. for $C_{18}H_{21}ClNO_2S^+$ 350.0976; Found: 350.0972.

HPLC (PHENOMENEX AMYLOSE-2, n-hexane/*i*-PrOH = 70/30, flow rate = 1.0 mL/min, l = 220 nm) t_R = 8.415 min (minor), t_R = 11.636 min (major), *e.r.* = 2:98.

- *(R)*- (*E*)-*N,N*,4-trimethyl-S-(3-oxo-5-phenylpent-4-en-2-ylidene)benzenesulfinamide (**1k**)

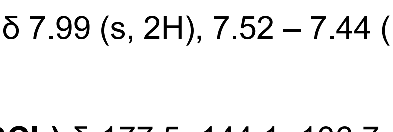

Yellow oil. Yield: 62% (141.8 mg).

^{1}H NMR (400 MHz, $CDCl_3$) δ 7.99 (s, 2H), 7.52 – 7.44 (m, 2H), 7.35 – 7.19 (m, 5H), 2.83 (s, 6H), 2.38 (s, 3H).

^{13}C{H} NMR (100 MHz, $CDCl_3$) δ 177.5, 144.1, 136.7, 136.4, 133.8, 130.0, 129.5,

128.6, 128.5, 127.6, 125.3, 73.6, 50.8, 36.6, 21.5.

IR ν max (cm^{-1}): 2923, 1633, 1527, 1494, 1448, 1360,1253, 1185, 1157, 1144, 1082, 1039,976, 928, 921,859, 847, 814, 762,748, 698, 661,649.

TLC: Rf = 0.26 (AcOEt).

[α]$_D^{22}$ = - 65.8 (c = 0.4, $CHCl_3$) for 92% *e.e.*

HRMS (ESI) *m/z*: [M+H]$^+$ Calcd. for $C_{20}H_{24}NO_2S^+$ 342.1522; Found: 342.1519.

HPLC (AD-H, n-hexane/*i*-PrOH = 80/20, flow rate = 1.0 mL/min, l = 220 nm) t_R = 9.380 min (minor), t_R = 26.361 min (major), *e.r.* = 4:96.

- *(R)-N,N*,4-trimethyl-S- (1-oxo-1-phenylpentan-2-ylidene) benzenesulfinamide (**1l**)

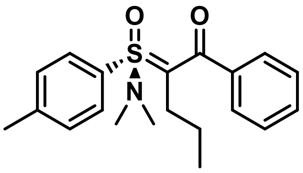

Pale yellow oil. Yield: 64% (147.2 mg).

^{1}H NMR (400 MHz, $CDCl_3$) δ 8.06 (d, J = 8.3 Hz, 2H), 7.40 – 7.35 (m, 2H), 7.33 – 7.28 (m, 5H), 2.92 (s, 6H), 2.45 – 2.40 (m, 5H), 1.48 – 1.34 (m, 2H), 0.72 (t, J = 7.3 Hz, 3H).

^{13}C{H} NMR (125 MHz, $CDCl_3$) δ 185.4, 144.0, 143.1, 134.7, 129.5, 128.7, 128.4, 127.8, 126.8, 36.9, 29.7, 26.0, 21.5, 13.6.

IR ν max (cm^{-1}): 2957, 2927, 2869, 1593, 1580, 1537,1489, 1455, 1443, 1366, 1303, 1282,1265, 1243, 1183, 1129, 1081, 1047, 1018, 935, 888, 812, 784, 722, 699, 638, 623.

TLC: Rf = 0.4 (AcOEt).

[α]$_D^{22}$ = - 75.4 (c = 1.3, $CHCl_3$) for 92% *e.e.*

HRMS (ESI) *m/z*: [M+H]$^+$ Calcd. for $C_{20}H_{26}NO_2S^+$ 344.1679; Found: 344.1673.

HPLC (AD-H, n-hexane/*i*-PrOH = 80/20, flow rate = 1.0 mL/min, l = 220 nm) t_R = 6.236 min (minor), t_R = 12.340 min (major), *e.r.* = 4:96.

- *(R)- N,N*,4-trimethyl-S-(1-(naphthalen-2-yl)-1-oxopentan-2-

ylidene)benzenesulfinamide (**1m**)

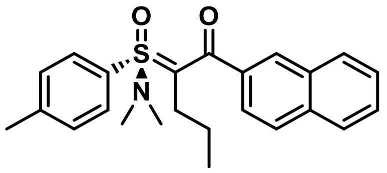

Pale yellow oil/wax. Yield: 40% (105.5 mg).

^{1}H NMR (400 MHz, $CDCl_3$) δ 8.08 (d, J = 8.1 Hz, 2H), 7.92 – 7.72 (m, 4H), 7.54 – 7.39 (m, 3H), 7.35 – 7.28 (m, 2H), 2.94 (s, 6H), 2.52 – 2.43 (m, 2H), 2.41 (s, 3H), 1.43 (dq, J = 14.8, 7.4 Hz, 2H), 0.69 (t, J = 7.3 Hz, 3H).

^{13}C{H} NMR (100 MHz, $CDCl_3$) δ 185.1, 144.0, 140.5, 134.6, 133.3, 132.8, 129.5, 128.7, 128.4, 127.6, 127.4, 126.1, 126.0, 125.9, 125.1, 80.0, 37.0, 29.8, 26.0, 21.5, 13.6.

IR ν max (cm^{-1}): 3054, 2957, 2926, 2869, 1595, 1539,1463, 1372, 1269, 1190, 1128, 1082,1049, 938, 821, 729.

TLC: Rf = 0.48 (AcOEt).

$[\alpha]_D^{22}$ = - 16.5 (c = 0.4, $CHCl_3$) for 98% *e.e.*

HRMS (ESI) *m/z*: $[M+H]^+$ Calcd. for $C_{24}H_{28}NO_2S^+$ 394.1835; Found: 394.1833.

HPLC (PHENOMENEX AMYLOSE 2, n-hexane/*i*-PrOH = 80/20, flow rate = 1.0 mL/min, l = 220 nm) t_R = 11.802 min (minor), t_R = 20.284 min (major), *e.r.* = 1:99.

- *N,N*-diethyl-4-methyl-*S*-(1-oxo-1-phenylpropan-2-ylidene)benzenesulfinamide (**1n**)

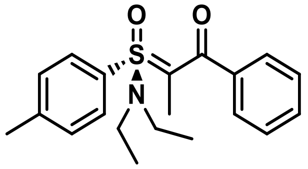

White solid. Yield: 50% (115.0 mg).

^{1}H NMR (400 MHz, $CDCl_3$) δ 8.05 (d, J = 8.0 Hz, 2H), 7.45 – 7.36 (m, 2H), 7.32 – 7.22 (m, 5H), 3.45 – 3.23 (m, 4H), 2.38 (s, 3H), 2.03 (s, 3H), 1.21 (t, J = 7.1 Hz, 6H).

^{13}C{H} NMR (100 MHz, $CDCl_3$) δ 184.0, 143.8, 142.7, 134.7, 129.2, 128.8, 128.6, 127.6, 127.4, 73.2, 40.4, 21.5, 13.6, 13.5.

IR ν max (cm^{-1}): 3057, 2976, 2934, 2875, 1581, 1540,1462, 1383, 1259, 1177, 1080, 914, 812, 786, 711, 689.

TLC: Rf = 0.53 (AcOEt).

[α]D[22] = - 24.1 (c = 0.7, $CHCl_3$) for 94% *e.e.*

HRMS (ESI) *m/z*: $[M+H]^+$ Calcd. for $C_{20}H_{26}NO_2S^+$ 344.1679; Found: 344.1675.

HPLC (AD-H, n-hexane/*i*-PrOH = 80/20, flow rate = 1.0 mL/min, l = 220 nm) t_R = 5.795 min (minor), t_R = 7.485 min (major), *e.r.* = 3:97.

2.2 PREPARATION OF PRO-CHIRAL SULFOXONIUM YLIDES

- Triethylsulfoxonium chloride (**SM-E**)

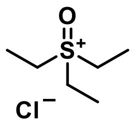

Prepared according to literature.[8]

Hygroscopic white solid. Yield: 75% (2.905 g, 2 steps starting from diethyl sulfide).

^{1}H NMR (400 MHz, DMSO-d6): δ 4.23 (q, J = 7.4 Hz, 4H), 1.42 (t, J = 7.4 Hz, 5H).

^{13}C{H} NMR (126 MHz, DMSO-d6) δ 42.4, 4.7.

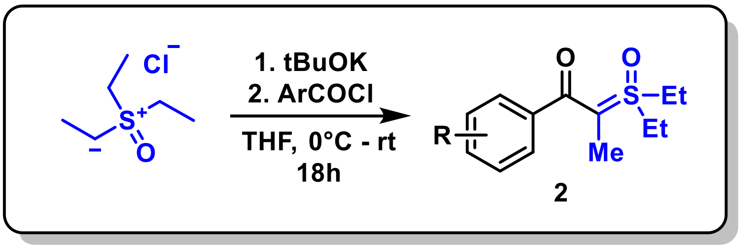

To a 25 mL flask were added *t*-BuOK (505.0 mg, 4.5 mmol, 2 eq) and Et_3SOCl (256 mg, 1.5 mmol, 1.0 eq). The vial was purged with Argon, and then 10 mL of dry THF was added. This suspension was stirred under reflux for 2 h. After this time, the reaction suspension was cooled to 0 °C, and to it was added a solution of the respective benzoyl chloride (1.5 mmol, 1.0 eq) in 2 mL of THF. The reaction was naturally warmed to room temperature and stirred for 16 h. After this time, the reaction was terminated by the addition of 5 mL of H_2O (stirring for 5 min). Then, the biphasic mixture was rotoevaporated to eliminate THF, and 10 mL of water was added to the aqueous suspension and extracted with AcOEt (3 x 20 mL), monitoring by TLC. The combined organic phases were dried ($MgSO_4$), filtered and concentrated in a rotovap. This residue was then purified by flash column (SiO_2,

$MeOH/CHCl_3$ 0.5%-> 5%).

- 2-(diethyl(oxo)-λ^6-sulfaneylidene)-1-phenylpropan-1-one (**2a**)

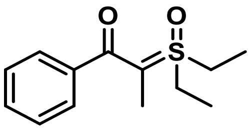

White solid. Yield: 51% (723.0 mg – 4.5 mmol scale).

^{1}H NMR (500 MHz, $CDCl_3$) δ 7.58 – 7.48 (m, 2H), 7.42 – 7.31 (m, 3H), 4.33 (m, 2H), 3.32 (m, 2H), 1.99 (s, 3H), 1.39 (t, J = 7.4 Hz, 6H).

^{13}C{H} NMR (125 MHz, $CDCl_3$) δ 182.7, 141.6, 129.1, 128.0, 127.5, 67.9, 46.4, 11.5, 5.1.

MP = 78-81°C.

TLC: Rf = 0.31 (5% MeOH/AcOEt);

IR ν max (cm^{-1}): 3446, 2976, 2935, 2875, 1672, 1595, 1580, 1514, 1449, 1375, 1330, 1308, 1232, 1202, 1182, 1051, 1020, 1182, 1051, 1020, 1000, 988, 957, 945, 782, 758, 721, 688.

HRMS (ESI) *m/z*: $[M+H]^+$ Calcd. for $C_{13}H_{19}O_2S^+$ 239.1100; Found: 239.1099.

- 2-(diethyl(oxo)-λ^6-sulfaneylidene)-1-(furan-2-yl) propan-1-one (**2b**)

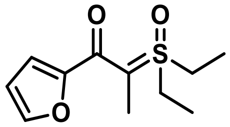

White solid. Yield: 78% (267.3 mg).

^{1}H NMR (500 MHz, $CDCl_3$) δ 7.49 (m, 1H), 6.86 (m, 1H), 6.47 (m, 1H), 4.33 (dq, J = 13.3, 7.6 Hz, 2H), 3.32 (dq, J = 14.4, 7.2 Hz, 2H), 2.17 (s, 3H), 1.34 (t, J = 7.4 Hz, 6H).

^{13}C{H} NMR (125 MHz, $CDCl_3$) δ 169.2, 153.8, 143.2, 112.7, 111.1, 68.5, 46.6, 9.3, 5.1.

MP = 103-105°C.

TLC: Rf = 0.14 (2% MeOH/AcOEt);

IR ν max (cm^{-1}): 2979, 2936, 2875, 1577, 1515, 1469, 1389, 1370, 1174, 1012, 994,910, 797, 748, 691.

HRMS (ESI) *m/z*: $[M+H]^+$ Calcd. for $C_{11}H_{17}O_3S^+$ 229.0893; Found: 229.0894.

- 2-(diethyl(oxo)-λ^6-sulfaneylidene)-1-(4-methoxyphenyl) propan-1-one (**2c**)

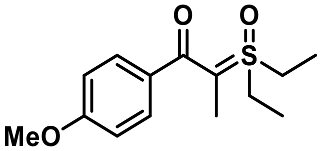

White solid. Yield: 66% (265.3 mg).

^{1}H NMR (500 MHz, CDCl$_3$) δ 7.61 – 7.49 (m, 2H), 6.94 – 6.84 (m, 2H), 4.37 – 4.26 (m, 2H), 3.83 (s, 3H), 3.41 – 3.26 (m, 2H), 2.02 (d, J = 1.5 Hz, 3H), 1.41 – 1.33 (m, 6H).

^{13}C{H} NMR (125 MHz, CDCl$_3$) δ 181.9, 160.4, 133.9, 129.3, 113.2, 67.8, 55.4, 46.6, 11.7,5.2.

MP = 92-94°C.

TLC: Rf = 0.20 (5% MeOH/AcOEt);

IR ν max (cm^{-1}): 2997, 2936, 1604, 1499, 1455, 1375, 1246, 1167, 1030, 987, 841, 790, 754, 700.

The spectroscopic data are in agreement with those reported in the literature.[8]

- 2-(dietil(oxo)-λ^6-sulfanolideno)-1-(4-nitrofenil)propan-1-one (**2d**)

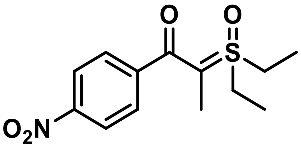

Yellow solid.Yield: 58% (246.6 mg).

^{1}H NMR (500 MHz, CDCl$_3$) δ 8.33 – 8.12 (m, 2H), 7.88 – 7.49 (m, 2H), 4.31 (dq, J =13.4, 7.6 Hz, 2H), 3.39 (dq, J = 14.4, 7.2 Hz, 2H), 1.95 (s, 3H), 1.42 (t, J = 7.4 Hz, 6H).

^{13}C{H} NMR (125 MHz, CDCl$_3$) δ 179.7, 147.9, 147.8, 128.4, 123.5, 70.0, 46.3, 11.2, 5.1.

MP = 130-132°C.

TLC: Rf = 0.55 (5% MeOH/AcOEt);

IR ν max (cm^{-1}): 2967, 2920, 1603, 1524, 1378, 1345, 1315, 1172, 990, 856, 809, 714.

HRMS (ESI) *m/z*: $[M+H]^+$ Calcd. for $C_{13}H_{18}NO_4S^+$ 284.0951; Found: 284.0951.

- 2-(diethyl(oxo)-λ[6]-sulfaneylidene)-1-(p-tolyl) propan-1-one (**2e**)

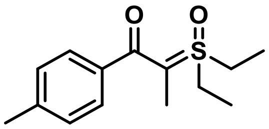

Pale yellow solid. Yield: 49% (185 mg). Purification by column chromatography in 5% MeOH/EtOAc.

^{1}H NMR (500 MHz, $CDCl_3$) δ 7.46– 7.44 (m, 2H), 7.19 – 7.16 (m, 2H), 4.33 (dq, *J*= 13.2, 7.5 Hz, 2H), 3.33 (dq, *J*= 13.1, 7.0 Hz, 2H), 2.36 (s, 3H), 1.99 (s, 3H), 1.38 (t, *J*= 7.4 Hz, 6H) ppm

^{13}C{H} NMR (125 MHz, $CDCl_3$) δ 182.5, 139.2, 138.5, 129.0, 128.7, 127.6, 68.4, 46.7, 21.5, 11.6, 5.2 ppm

MP = 97-98°C.

TLC: R_f = 0.26 (100% AcOEt);

IR ν max (cm^{-1}): 2970, 2962, 2224, 1675, 1605, 1532, 1441, 1315, 1198.

HRMS (ESI) *m/z*: $[M+H]^+$ Calcd. for $C_{14}H_{21}O_2S^+$ 253.1262; Found: 253.1266.

- 1-(4-chlorophenyl)-2-(diethyl(oxo)-λ[6]-sulfaneylidene) propan-1-one (**2f**)

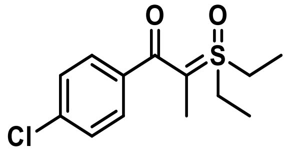

White solid. Yield: 64% (263.5 mg).

^{1}H NMR (500 MHz, $CDCl_3$) δ 7.52 – 7.45 (m, 2H), 7.39 – 7.32 (m, 2H), 4.32 (dq, J = 13.5, 7.6 Hz, 2H), 3.35 (dq, J = 14.4, 7.2 Hz, 2H), 1.97 (s, 3H), 1.39 (t, J = 7.4 Hz, 6H).

^{13}C{H} NMR (125 MHz, $CDCl_3$) δ 180.9, 139.7, 135.0, 129.0, 128.3, 69.4, 46.5, 11.5, 5.1.

MP = 82-84°C.

TLC: Rf = 0.27 (2% MeOH/AcOEt);

IR ν max (cm-1): 2979, 2936, 1591, 1573, 1510, 1484, 1375, 1171, 1080, 988, 840, 807, 747, 721.

HRMS (ESI) *m/z*: $[M+H]^+$ Calcd. for $C_{13}H_{18}ClO_2S^+$ 273.0711; Found: 273.0712.

- 1-(4-bromophenyl)-2-(diethyl(oxo)-λ^6-sulfaneylidene) propan-1-one (**2g**)

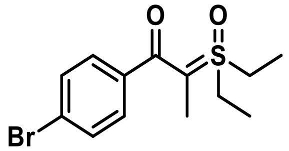

Purification by column chromatography in 5% MeOH/EtOAc.

White solid. Yield: 51% (242 mg).

^{1}H NMR (500 MHz, CDCl$_3$) δ 7.52– 7.49 (m, 2H), 7.43 – 7.40 (m, 2H), 4.28 (dq, *J*= 13.1, 7.5 Hz, 2H), 3.33 (dq, *J*= 13.2, 7.3 Hz, 2H), 1.96 (s, 3H), 1.38 (t, *J*= 7.4Hz, 6H) ppm.

^{13}C{H} NMR (125 MHz, CDCl$_3$) δ 181.3, 140.5, 131.2, 129.3, 123.2, 68.5, 46.4, 11.5, 5.1 ppm.

MP = 102-103°C.

TLC: R_f = 0.27 (100% AcOEt);

IR ν max (cm^{-1}): 2985, 2962, 2124, 1615, 1585, 1462, 1371, 1310, 1190, 1171.

HRMS (ESI) *m/z*: [M+H]$^+$ Calcd. for $C_{13}H_{18}BrO_2S^+$ 317.0211; Found: 317.0202.

- 1-([1,1'-biphenyl]-4-yl)-2-(diethyl(oxo)-λ^6-sulfaneylidene) propan-1-one (**2h**)

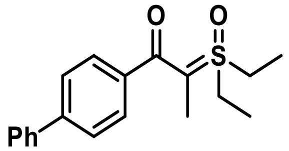

Purification by column chromatography in 5% MeOH/EtOAc.

Yellow solid. Yield: 62% (292 mg).

^{1}H NMR (500 MHz, CDCl$_3$) δ 7.64-7.60 (m, 6H), 7.44 (t, *J* = 7.7 Hz, 2H), 7.35 (t, *J* = 7.7 Hz, 1H), 4.33 (dq, *J*= 13.1, 7.4 Hz, 2H), 3.33 (dq, *J*= 13.1, 7.3 Hz, 2H), 2.05 (s, 3H), 1.40 (t, *J*= 7.3 Hz, 6H) ppm.

^{13}C{H} NMR (125 MHz, CDCl$_3$) δ 182.3, 141.9, 140.9, 140.4, 128.9, 128.1, 127.6, 127.3, 126.8, 68.1, 46.5, 11.6, 5.2 ppm.

MP = 105-107°C.

TLC: R_f = 0.43 (5% MeOH/AcOEt);

IR ν max (cm^{-1}): 2980, 2952, 2264, 1670, 1650, 1458, 1430, 1321, 1195, 1151, 731, 697.

HRMS (ESI) *m/z*: [M+H]$^+$ Calcd. for $C_{19}H_{23}O_2S^+$ 315.1418; Found: 315.1404.

- 1-(2-chlorophenyl)-2-(diethyl(oxo)-λ^6-sulfaneylidene) propan-1-one (**2i**)

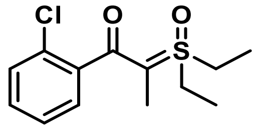

White solid. Yield: 58% (237.9 mg).

^{1}H NMR (500 MHz, CDCl$_3$) δ 7.39 – 7.33 (m, 1H), 7.31 – 7.21 (m, 3H), 4.39 (dq, J = 13.5, 7.6 Hz, 2H), 3.32 (dq, J = 14.4, 7.2 Hz, 2H), 1.73 (s, 3H), 1.45 (t, J = 7.4 Hz, 6H).

^{13}C{H} NMR (125 MHz, CDCl$_3$) δ 180.5, 141.5, 130.4, 129.6, 129.1, 128.4, 126.9, 69.2, 46.1, 10.2, 5.0.

MP = 95-98°C.

TLC: R_f = 0.43 (5% MeOH/AcOEt);

IR ν max (cm^{-1}): 2977, 2934, 1591, 1522, 1453, 1432, 1377, 1179, 1053, 1032, 987, 815, 765, 743, 707, 691.

HRMS (ESI) *m/z*: [M+H]$^+$ Calcd. for $C_{13}H_{18}ClO_2S^+$ 273.0712; Found: 273.0714.

- 2-(diethyl(oxo)-λ^6-sulfaneylidene)-1-(naphthalen-2-yl) propan-1-one (**2j**)

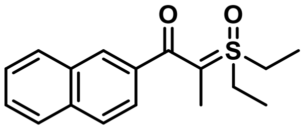

Purification by column chromatography in 5% MeOH/EtOAc.

Off White solid. Yield: 52% (225 mg).

^{1}H NMR (500 MHz, CDCl$_3$) δ 7.99 (s, 1H), 7.86 – 7.80 (m, 3H), 7.64 – 7.61 (m, 1H), 7.48 – 7.44 (m, 2H), 4.33 (dq, *J*= 13.2, 7.5 Hz, 2H), 3.31 (dq, *J*= 13.1, 7.0 Hz, 2H), 2.01 (s, 3H), 1.38 (t, *J*= 7.4 Hz, 6H) ppm.

^{13}C{H} NMR (125 MHz, CDCl$_3$) δ 182.4, 138.9, 133.6, 132.8, 128.5, 127.7, 126.9, 126.4, 126.1, 125.2, 68.4, 46.4, 44.8, 11.5, 6.7 ppm.

MP = 115-117°C.

TLC: R_f = 0.29 (100% AcOEt);

IR ν max (cm^{-1}): 2950, 2932, 2324, 1695, 1650, 1456, 1432, 1321, 1198, 1151, 731, 695.

HRMS (ESI) *m/z*: [M+H]$^+$ Calcd. for $C_{17}H_{21}O_2S^+$ 289.1262; Found: 289.1248.

2.2.1. SYNTHESIS OF SULFOXONIUM YLIDES VIA DIAZO COMPOUNDS:

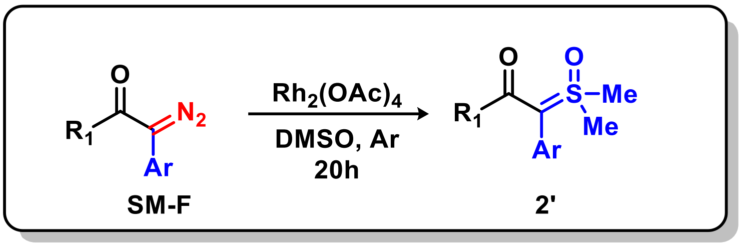

In a round bottom flask under argon atmosphere, a reddish solution of dry DMSO (23 mL) and Rhodium (II) acetate (1 mol %) was prepared at 70 °C. Then, a solution of diazoesters (**SM-F**) in dry DMSO (23 mL) was added dropwise for 18 h using a syringe pump. After the addition, the reaction was stirred for an additional 2 h. The reaction was then cooled to room temperature, diluted in ethyl acetate (150 mL), and washed with water (5 x 20 mL) until the ethyl acetate solution no longer contained DMSO (the extraction was monitored by TLC). Then, the organic phase was dried over magnesium sulfate, filtered, and concentrated. The residue was then suspended in a 10% EtOAc/Hexanes solution and filtered. The solid retained on the filter is then dried under high vacuum affording ylides (**2’**) as a white or yellow solid.

Ylides **2’a**[9], **2’b**[9], **2’c**[10], **2’e**[11], **2’f**[12], **2’g**[13], **2’h**[10], **2’k**[14] had already been reported, and their spectroscopic data were in agreement with those reported in the literature.

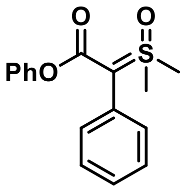

2'a

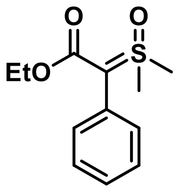

2'b

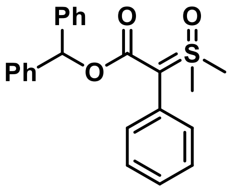

2'c

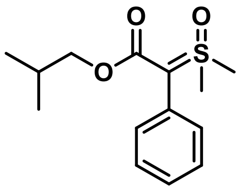

2'e

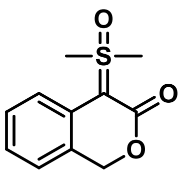

2'f

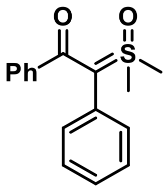

2'g

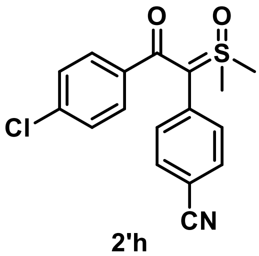

2'h

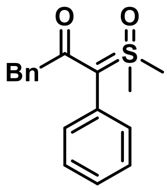

2'k

- benzyl 2-(dimethyl(oxo)-λ^6-sulfaneylidene)-2-phenylacetate (**2’d**).

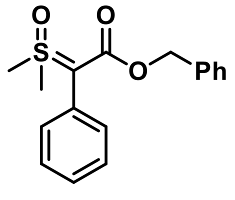

Yield: 34% (62.4 mg). Brown oil**.**

TLC: *R*f = 0.72 (AcOEt).

^{1}H NMR (500 MHz, CDCl$_3$): δ 7.39-7.18 (m, 10H), 5.13 (s, 2H), 3.39 (s, 5H).

^{13}C{H} NMR (100 MHz, CDCl$_3$) δ 165.9, 137.9, 133.8, 132.5, 128.5, 128.4, 127.4, 127.3, 127.2, 70.7, 64.5, 43.3.

IR (ATR, ν max, cm^{-1}) = 3028, 2927, 1628, 1593, 1322, 1216, 1171, 1087, 1016, 754,700.

HRMS (ESI) *m/z*: [M+H]$^+$ Calcd. for $C_{17}H_{19}O_3S^+$ 303.1049; Found: 303.1043.

- phenyl 2-(dimethyl(oxo)-λ^6-sulfaneylidene)-2-(p-tolyl) acetate (**2’i**)

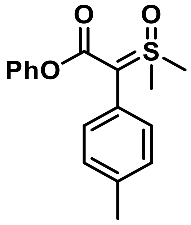

Purification by column chromatography in 5% MeOH/EtOAc.

White solid. Yield: 60% (181 mg).

TLC: Rf = 0.32 (100% AcOEt).

^{1}H NMR (500 MHz, CDCl3): δ 7.33-7.28 (m, 4H), 7.19-7.15 (m, 2H), 7.13-7.10 (m, 1H), 7.07-7.05 (m, 2H), 3.44 (s, 6H), 2.35 (s, 3H) ppm.

^{13}C{H} NMR (125 MHz, CDCl3): δ 164.8, 151.7, 137.4, 133.9, 129.4, 129.1, 124.8, 122.5, 70.5, 43.1, 21.4 ppm.

IR (ATR, ν max, cm^{-1}) = 2985, 2952, 2264, 1660, 1630, 1458, 1432, 1321, 1195, 1151, 740, 690.

MP = 145-147 °C

HRMS (ESI) *m/z*: [M+H]$^+$ Calcd. for $C_{17}H_{19}O_3S^+$ 303.1049; Found: 303.1057.

- phenyl 2-(dimethyl(oxo)-λ^6-sulfaneylidene)-2-(4-nitrophenyl) acetate (**2'j**)

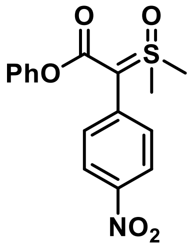

Prepared according to the general procedure B. Purification by column chromatography in 5-6% MeOH/EtOAc.

Yellow solid. Yield: 78% (260 mg).

TLC: Rf = 0.33 (100% AcOEt).

^{1}H NMR (500 MHz, CDCl3): δ 8.19-8.15 (m, 2H), 7.61-7.59 (m, 2H), 7.38-7.33 (m, 2H), 7.21-7.08 (m, 3H), 3.58(s, 6H) ppm.

^{13}C{H} NMR (125 MHz, CDCl3) δ 163.9, 151.0, 145.6, 139.3, 132.4, 129.4, 125.4, 123.3, 122.2, 120.4, 69.2, 44.0 ppm.

IR (ATR, ν max, cm^{-1}) = 3020, 2982, 2254, 1680, 1620, 1508, 1428, 1321, 1095.

MP = 150-151°C

HRMS (ESI) *m/z*: $[M+H]^+$ Calcd. for $C_{16}H_{16}NO_5S^+$ 334.0744; Found: 334.0751.

3. OPTIMIZATION STUDIES

3.1. CHIRAL AMINOSULFOXONIUM YLIDES

Table S1. Optimization of asymmetric α,α-chlorofluorination of chiral aminosulfoxonium ylides.

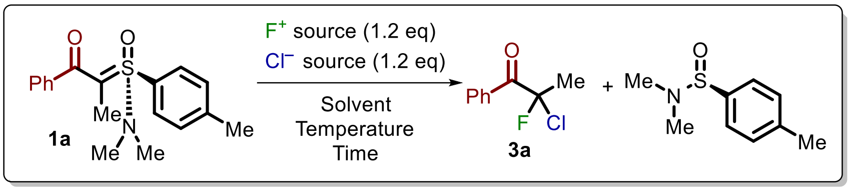

Entry	F^+ source	Cl^- source	Solvent	Temperature	Yield	e.r.[a]
1	Selectfluor	$BnBu_3NCl$	MeCN	r.t.	36%	70.5:29:5
2	Selectfluor	$BnBu_3NCl$	THF	r.t.	45%	66:34
3	Selectfluor	$BnBu_3NCl$	MeCN	5°C	41%	74:26
4[c]	Selectfluor	$BnBu_3NCl$	MeCN	-23°C	69%	75:25
5	Selectfluor	$BnBu_3NCl$	MeCN	-40°C	70%	91:9
6	Selectfluor	$BnBu_3NCl$	Hex/MeCN (2:1)	5°C	54%	74.5:25.5
7	Selectfluor	$BnBu_3NCl$	DMSO	5°C	20%	75:25
8	Selectfluor	$BnBu_3NCl$	AcOEt	5°C	28%	70:30
9	Selectfluor	$BnBu_3NCl$	DCM	5°C	61%	68.5:32.5
10	Selectfluor	KCl[b]	MeCN	5°C	4%	61.5:38.5
11	Selectfluor	KCl[b]	DCM	5°C	26%	68.5:32.5
**12**	**$[Me_3pyF]BF_4$**	**$BnBu_3NCl$**	**MeCN**	5°C	**75%**	**95:5**
13	NFSI	$BnBu_3NCl$	MeCN	5°C	86%	65.5:34.5
14	$[Me_3pyF]BF_4$	$BnBu_3NCl$	MeCN	r.t.	38%	94:6
15	$[Me_3pyF]BF_4$	$BnBu_3NCl$	MeCN	-23°C	68%	95:5
16	$[Me_3pyF]BF_4$	$BnBu_3NCl$	MeCN	-40ºC	70%	91:9
17	$[Me_3pyF]BF_4$	$BnBu_3NCl$	$CHCl_3$	5°C	39%	92:8
18	$[Me_3pyF]BF_4$	$BnBu_3NCl$	TFE	5°C	5%	89:11
19	$[Me_3pyF]BF_4$	$BnBu_3NCl$	DMF	5°C	58%	95:5
20	$[Me_3pyF]BF_4$	$BnBu_3NCl$	1,2-DCE	5°C	51%	94:6

[a]e.r. determined by HPLC – Phenomenex Amylose 2 Chiral Column, 100% hexanes, 0.7ml.min^{-1}, 254 nm; [b]2.0 eq of KCl; [c]Reaction run for 20h

3.2. CATALYTIC STUDIES

Table S2. Initial tests involving asymmetric α,α-chlorofluorination of α-carbonyl sulfoxonium ylides.

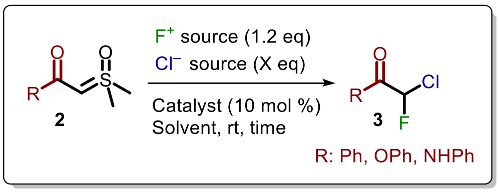

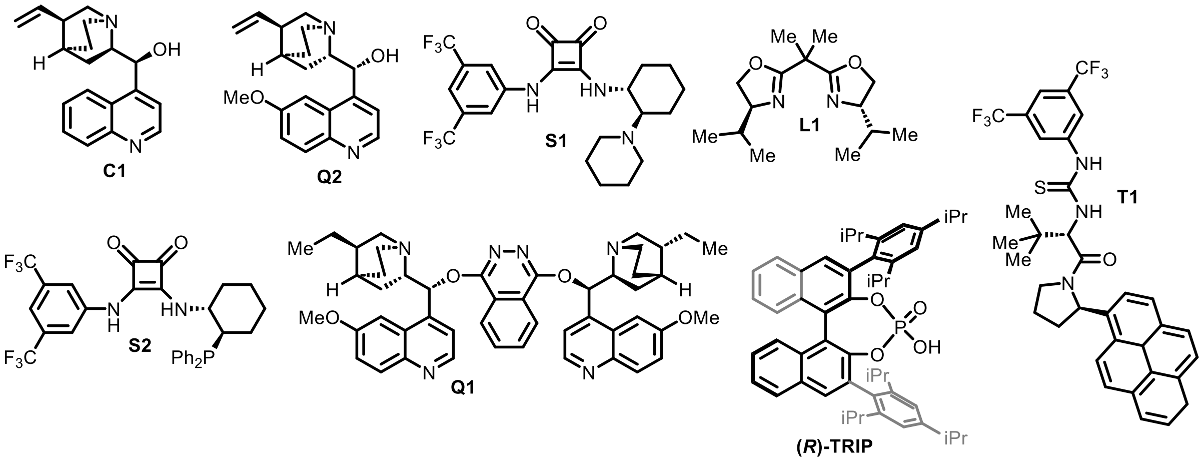

Entry	R	F+ source	Cl− source	Solvent	Catalyst	Yield	e.r.
1	Ph	Selectfluor	$BnBu_3NCl$ (1.1 eq)	THF	C1	73%	0%
2	Ph	Selectfluor	$BnBu_3NCl$ (1.1 eq)	THF	Q2	76%	0%
3	Ph	Selectfluor	$BnBu_3NCl$ (1.1 eq)	THF	Q1	29%	0%
4	Ph	Selectfluor	$BnBu_3NCl$ (1.1 eq)	THF	S1	50%	0%
5[a]	Ph	Selectfluor	$BnBu_3NCl$ (1.1 eq)	$CF_3C_6H_5$	(*R*)-TRIP[b]	35%	0%
6	Ph	Selectfluor	$BnBu_3NCl$ (1.1 eq)	DCM	$Cu(OTf)_2$-L1	36%	0%
7	Ph	Selectfluor	KCl (2.0 eq)	THF	$CuCl_2$-S2	23%	0%
8	Ph	Selectfluor	KCl (2.0 eq)	THF	$NiCl_2$-S2	63%	0%
9	Ph	Selectfluor	KCl (2.0 eq)	THF	T1	20%	0%
10	Ph	NFSI	KCl (2.0 eq)	THF	S1	traces	-
11	Ph	$[Me_3pyF]BF_4$	KCl (2.0 eq)	THF	S1	13%	0%
12	Ph	$[Me_3pyF]BF_4$	$BnBu_3NCl$ (1.1 eq)	THF	S1	69%	0%
13	Ph	NFSI	$BnBu_3NCl$ (1.1 eq)	THF	S1	80%	0%
14	OPh	Selectfluor	KCl (2.0 eq)	THF	S1	CM	-
15	NHPh	Selectfluor	KCl (2.0 eq)	THF	S1	41%	0%

[a]1.5 eq of Selectfluor and Na_2CO_3 (1.5 eq) as additive; [b]5 mol%; CM:complex mixture

Table S3. Initial tests involving asymmetric α,α-chlorofluorination of pro-chiral α-carbonyl sulfoxonium ylides.

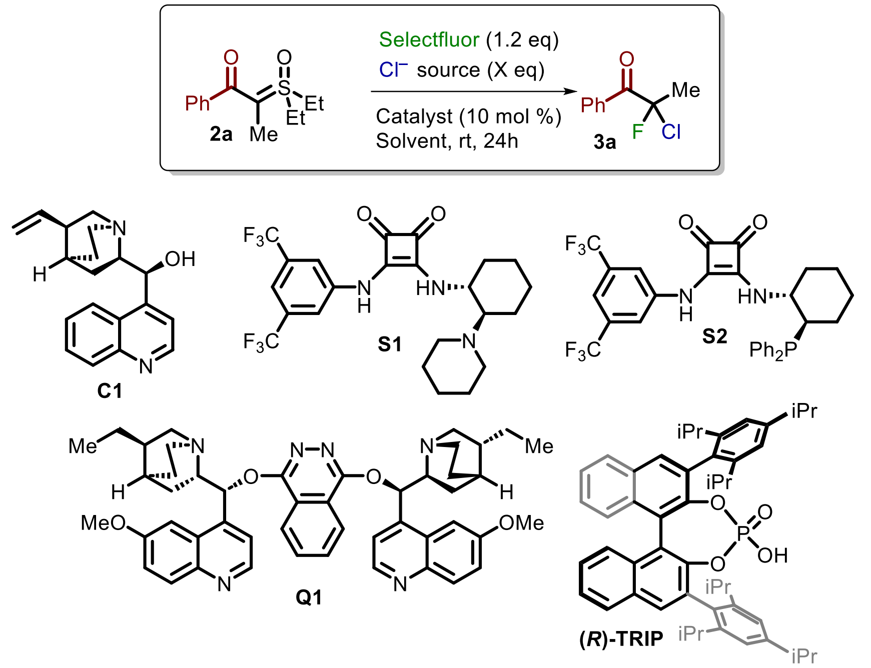

Entry	Cl⁻ source	Solvent	Catalyst	Yield	e.r.
1	BnBu3NCl (1.2 eq)	THF	-	77%	-
2	BnBu3NCl (1.2 eq)	THF	C1	33%	0%
3	KCl (2.0 eq)	THF	C1	18%	0%
4	BnBu3NCl (1.2 eq)	THF	Q1	46%	0%
5[a]	BnBu3NCl (1.2 eq)	THF	S2	68%	0%
6	BnBu3NCl (1.2 eq)	DCM	(*R*)-TRIP	69%	0%
7	BnBu3NCl (1.2 eq)	THF	NiCl2-S2	61%	0%

3.3. PRO-CHIRAL SULFOXONIUM YLIDES

Table S4. Optimization conditions for asymmetric α,α–chlorofluorination of sulfoxonium ylides using Shibata's chiral fluorinating agents.

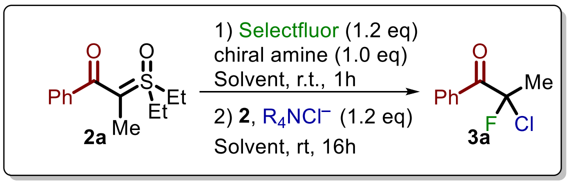

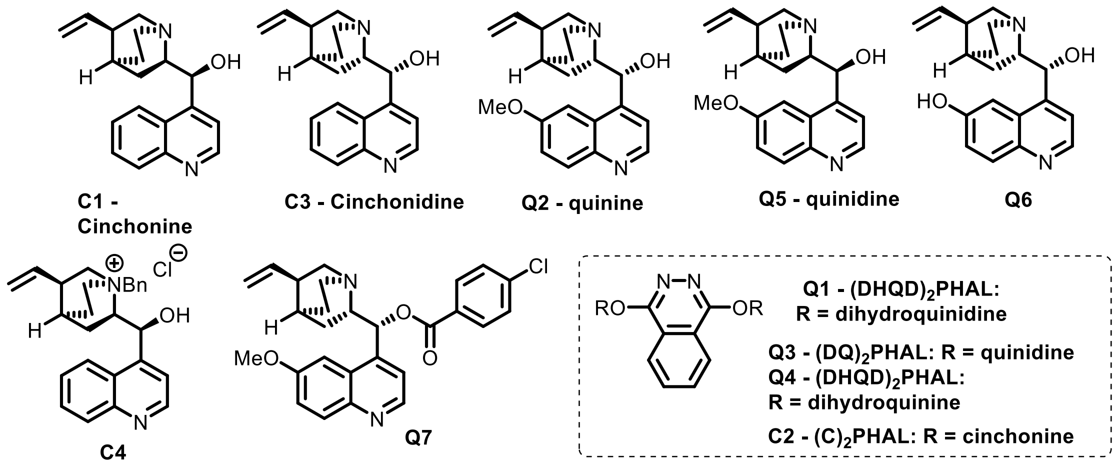

Entry	2a (eq)	R_4NCl	Chiral amine	Solvent	Temperature	Yield	e.r.[a]
1	1.0	$BnBu_3NCl$	Q1	MeCN	r.t.	44%	84:16
2	1.0	$BnBu_3NCl$	Q3	MeCN	r.t.	45%	70:30
3	1.0	$BnBu_3NCl$	C1	MeCN	r.t.	51%	71.5:28.5
4	1.0	$BnBu_3NCl$	C3	MeCN	r.t.	57%	58:42
5	1.0	$BnBu_3NCl$	Q2	MeCN	r.t.	38%	56:44
6	1.0	$BnBu_3NCl$	Q5	MeCN	r.t.	35%	53:47
7	1.0	$BnBu_3NCl$	Q1	MeCN	-23°C	41%	85:15
8	1.0	$BnBu_3NCl$	C1	MeCN	-23°C	65%	77:23
9	1.2	$BnBu_3NCl$	Q1[b]	MeCN	-23°C	27%	85:15
10	1.2	$BnBu_3NCl$	C1[b]	MeCN	-23°C	69%	79:21
11	1.2	$BnBu_3NCl$	Q7	MeCN	-23°C	53%	61:39
12	1.0	Q5	Q1	MeCN	-23°C	35%	85:15
13	1.0	Q5	-	MeCN	-23°C	83%	51:49
14	1.0	$BnBu_3NCl$	Q6	MeCN	-23°C	48%	54.5:45.5
15	1.0	$BnBu_3NCl$	Q3	MeCN	-23°C	55%	85:15
16	1.0	$BnBu_3NCl$	Q3[c]	MeCN	-23°C	45%	85:15
17	1.0	$BnBu_3NCl$	C2	MeCN	-23°C	63%	70.5:29.5
18	1.0	$BnBu_3NCl$	Q3	MeCN+DCM	-23°C	47%	81.5:18.5

19	1.0	$BnBu_3NCl$	Q3	MeCN+acetone	-23°C	50%	85.5:14.5
20	1.0	$BnBu_3NCl$	Q3	DCM	-23°C	23%	60.5:39.5
21	1.0	$BnBu_3NCl$	Q1	Acetone+MeCN	-23°C	50%	77:23
**22**	**1.0**	**$BnBu_3NCl$**	**Q3**	**Acetone**	**-23°C**	**61%**	**89:11**
23	1.0	$BnBu_3NCl$	Q3	AcOEt	-23°C	61%	60.5:39.5
24	1.0	$BnBu_3NCl$	Q3	DMF	-23°C	68%	85.5:14.5
25	1.0	$BnBu_3NCl$	Q3[d]	Acetone	-23°C	61%	86.5:13.5
26	1.0	$BnBu_3NCl$	Q3[e]	Acetone	-23°C	57%	89:11
27	1.0	$BnBu_3NCl$	Q3[f]	Acetone	-23°C	0%	-
28	1.0	$BnBu_3NCl$	Q3[g]	Acetone	-23°C	41%	89:11
29	1.0	$BnBu_3NCl$	Q3[h]	Acetone	-23°C	36%	58:42
30	1.0	$BnBu_3NCl$	(-)-Sparteine	Acetone	-23°C	0%	-
31	1.0	$BnBu_3NCl$	Q3	Acetone	-40°C	16%	82:18

Reaction conditions: 2 (0.05 mmol), "F^+" (0.05 mmol), chiral amine (0.05 mmol), $BnBu_3NCl$ (0.05 mmol) solvent (0.5 mL, 0.1M), 16 h, -23 °C. [a]e.r. determined by HPLC – Phenomenex Amylose 2 Chiral Column, 100% hexanes, 0.7ml.min^{-1}, 254 nm; [b]1.3 eq; [c]Recovered catalyst from entry 15, [d]1.2 eq; [e]1.5 eq, [f]F3 instead of selectfluor, [g]3eq. of $BnBu_3NCl$, [h]10 mol % of Q3

Brief discussion: To our delight, in our first entry using Q1 as a pre–formed chiral "F^+" source, we were able to isolate the desired product **3a** in 44% yield and 84:16 e.r. Using other chiral amines instead of Q1 resulted in lower enantioselectivities (entries 2–6). Lower temperatures were beneficial (entries 7-17), and Q3 furnished the product with the same selectivity and better yield (entry 15), and ever after one cycle of reuse (entry 16), after 82% yield of recovery. Other solvents were screened (entries 18–24), and acetone provided improved yield and enantioselectivity compared to acetonitrile (61%, 89:11 e.r., entry 22). Using higher amounts of Q3 (entries 25-26) or $BnBu_3NCl$ (entry 28) didn't improve the results. Otherwise, catalytic amounts of Q3 (10 mol %) led to a decrease in yields and enantioselectivity (entry 29). The use of F3 as fluorinating reagent, as well as lowering the temperature to -40°C didn't led to any improvement of the reaction (entries 27 and 31).

4. DIHALOGENATION REACTIONS

4.1 DIHALOGENATION PROCEDURE: PRO-CHIRAL SULFOXONIUM YLIDES

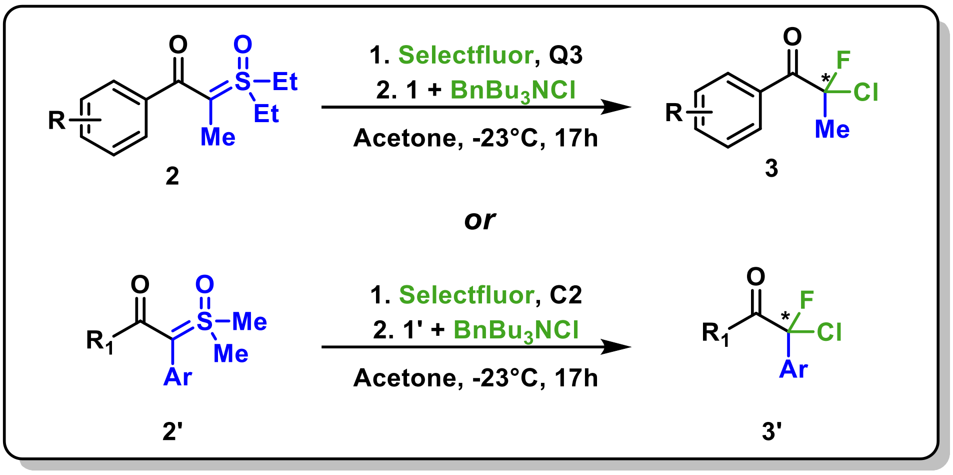

In a 4.0 mL vial, Selectfluor (54.2 mg, 0.153 mmol, 98%, 1.0 eq) and QD2PHAL-Q3 (116.2 mg, 0.15 mmol, 1.0 eq) were added for ethyl sulfoxonium ylide (**2**), or Cinchonine-C2 (29,1 mg, 0,15 mmol, 1,0 eq) for aryl sulfoxonium ylide (**2’**). The vial was closed, the vial was purged with argon atmosphere, 1.5 mL of acetone was added, and this solution was stirred at rt for 1 h. In another 4.0 mL vial, the respective ylide sulfoxonium (0.15 mmol, 1.0 eq), $BnBu_3NCl$ (56.1 mg, 0.18 mmol, 1.2 eq) and acetone (1.0 mL) were added, under argon atmosphere. The Selectfluor + catalyst solution was then stirred in the freezer for 5 minutes, and then the ylide solution was added to this solution. The reaction was then kept stirring in the freezer (-23°C) for 16 h. After this time, the reaction was concentrated (not evaporated to dryness) and then directly purified on a short SiO_2 column (1% EtOAc/Hexanes). The catalyst was recovered from the column using a 2% TEA solution in 1:1 EtOH/EtOAc. The column fractions were concentrated (not evaporated to dryness) in the rotovap, and the concentrate was transferred to a previously weighed vial and allowed to evaporate naturally over 24 h. This procedure is necessary because most dihalogenated products are volatile and attempts to evaporate to dryness in the rotovap resulted in large losses of product mass.

Note: for the optimization tables, the ylide scale used was 0.05 mmol, using the same proportions.

4.2 DIHALOGENATION PROCEDURE: CHIRAL AMINOSULFOXONIUM YLIDES

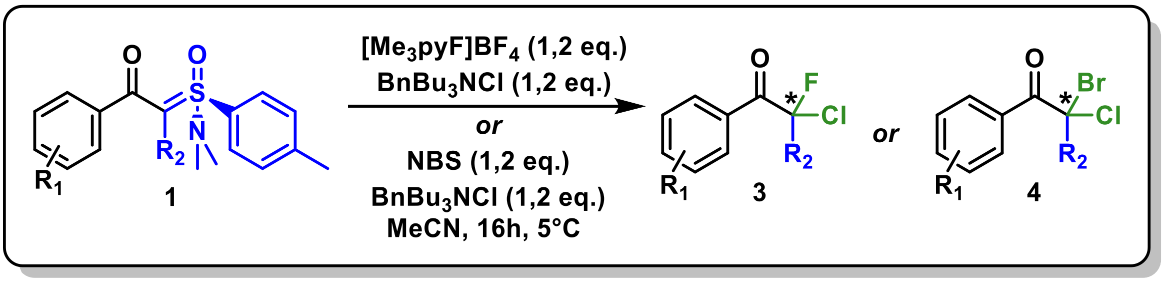

In a 4.0 mL vial were added the respective chiral aminosulfoxonium ylide (0.10 mmol, 1.0 eq), electrophilic fluorine reagent ([Me_3pyF]BF_4) (0.12 mmol, 1.2 eq) or electrophilic bromine reagent (NBS) (0.12 mmol, 1.2 eq), $BnBu_3NCl$ (0.12 mmol, 1.2 eq), and MeCN (1.0 mL). The reaction was then stirred at 5°C for 16h. Then, the solvent evaporated, and the crude reaction mixture was directly purified by flash column chromatography (SiO_2, EtOAc/Hexanes). The column fractions were concentrated (not evaporated to dryness) in a rotovap in a cold bath, and the concentrate was transferred to a previously weighed flask and allowed to evaporate naturally over 24 hours, providing the chlorofluorinated compound (**3**) or chlorobrominated compound (**4**). This procedure is necessary because most dihalogenated products are extremely volatile and attempts to evaporate to dryness in the rotovap resulted in large losses of product mass.

Note: a 0.10 mmol ylide scale was used for the scope, and a 0.05 mmol ylide scale was used for the optimization tables.

5. CHARACTERIZATION OF PRODUCTS

5.1 ALFA-ALKYL CARBONYL DIHALOGENATION PRODUCTS

- 2-chloro-2-fluoro-1-phenylpropan-1-one (**3a**)

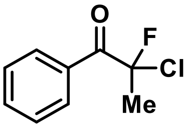

Colorless oil. Yield: 75% (14.0 mg – from **1a**), 65% (181.1 mg – from 1.5 mmol scale)
^{1}H NMR (400 MHz, $CDCl_3$) δ 8.23 – 8.14 (m, 2H), 7.64 – 7.56 (m, 1H), 7.54 – 7.42 (m, 2H), 2.18 (d, J = 20.2 Hz, 3H).

$^{13}C\{H\}$ NMR (100 MHz, $CDCl_3$) δ 189.4 (d, J = 29.4 Hz), 134.1, 131.5 (d, J = 3.8 Hz), 130.8 (d, J = 5.0 Hz), 128.6 (d, J = 0.7 Hz), 108.5 (d, J = 256.6 Hz), 27.5 (d, J = 23.4 Hz).

^{19}F NMR (470 MHz, $CDCl_3$) δ -106.19 (q, J = 20.1 Hz).

TLC: Rf = 0.58 (2% AcOEt/Hex);

$[\alpha]_D^{24}$ = +4.24 (c = 0.49, $CHCl_3$) for 78% *e.e.*

$[\alpha]_D^{24}$ = +5.96 (c = 0.21, $CHCl_3$) for 90% *e.e.*

IR ν max (cm^{-1}): 2976, 2935, 2875, 1672, 1595, 1580, 1514, 1449, 1375, 1330, 1308, 1232, 1202, 1182, 1051, 1020 1000, 988, 957, 945, 782, 758, 721, 688.

HPLC (Phenomenex Amylose 2, n-hexane, flow rate = 0.7 mL/min, l = 254 nm) t_R = 9.375 min (major), t_R = 9.723 min (minor), *e.r.* = 95:5. From the reaction using chiral ylide **1a**.

HPLC (Phenomenex Amylose 2, n-hexane, flow rate = 0.7 mL/min, l = 254 nm) t_R = 8.616 min (major), t_R = 8.974 min (minor), *e.r.* = 89:11. From the reaction using achiral ylide **2a**, the best condition from the optmization.

HPLC (Phenomenex Amylose 2, n-hexane, flow rate = 0.7 mL/min, l = 254 nm) t_R = 9.359 min (major), t_R = 9.799 min (minor), *e.r.* = 85:15. From the reaction using achiral ylide **2a**, the best condition from the optmization. From 1.5 mmol scale.

HPLC (Phenomenex Amylose 2, n-hexane, flow rate = 0.7 mL/min, l = 254 nm) t_R = 10.765 min (major), t_R = 11.300 min (minor), *e.r.* = 94:06. From the reaction using achiral ylide **2a**, the best condition from the optmization. From the reaction using chiral ylide **1n**.

This compound proved to be extremely apolar. We believe that the presence of any trace amounts of water or of the reaction solvents (acetone or CH_3CN) in the sample can slightly change the retention times during the HPLC analysis in some cases. However, in every chromatographic run, the compound was pure and had its identity confirmed by ^{1}H-NMR.

The spectroscopic data are in agreement with those reported in the literature.[15]

- 2-chloro-2-fluoro-1-(furan-2-yl) propan-1-one (**3b**)

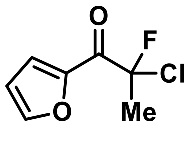

Colorless oil. Yield: 60% (10.6 mg) from **1b** and 40% (10.55 mg) from **2b**.

^{1}H NMR (500 MHz, CDCl$_3$) δ 7.74 (dd, J = 1.7, 0.7 Hz, 1H), 7.52 (td, J = 3.6, 0.7 Hz, 1H), 6.62 (dd, J = 3.7, 1.7 Hz, 1H), 2.16 (d, J = 20.1 Hz, 3H).

^{13}C{H}NMR (126 MHz, CDCl$_3$) δ 178.4 (d, J = 29.7 Hz), 148.7, 147.3 (d, J = 4.1 Hz), 123.1 (d, J = 9.8 Hz), 112.7 (d, J = 1.8 Hz), 107.8 (d, J = 254.2 Hz), 27.0 (d, J = 22.9 Hz).

^{19}F NMR (376 MHz, CDCl$_3$) δ -107.52(m).

TLC: Rf = 0.37 (2% AcOEt/Hex);

$[\alpha]_D^{24}$ = +13.4 (c = 0.80, CHCl$_3$) for 56% *e.e.*

$[\alpha]_D^{24}$ = +28.1° (c = 0.50, CHCl$_3$) for 72% *e.e.*

IR ν max (cm^{-1}): 3151, 2925, 2854, 1683, 1461, 1394, 1380, 1154, 1083, 1035, 921, 875, 768, 698.

HRMS (ESI) *m/z*: [M+H]$^+$ Calcd. for $C_7H_7ClFO_2^+$ 177.0113; Found: 177.0110.

HPLC (Phenomenex Amylose 2, n-hexane, flow rate = 0.7 mL/min, l = 254 nm) t_R = 31.107 min (major), t_R = 33.515 min (minor), *e.r.* = 78:22.

HPLC (Phenomenex Amylose 2, n-hexane/*i*-PrOH = 99.5/0.5, flow rate = 0.6 mL/min, l = 254 nm) t_R = 11.106 min (major), t_R = 11.761 min (minor), *e.r.* = 86:14.

- 2-chloro-2-fluoro-1-(4-methoxyphenyl) propan-1-one (**3c**)

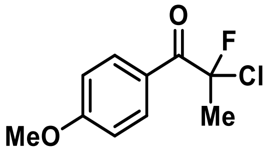

Colorless oil. Yield: 68% (14.8 mg) from **1c** and 70% (22.85 mg) from **2c**.

^{1}H NMR (500 MHz, CDCl$_3$) δ 8.22 – 8.16 (m, 2H), 6.99 – 6.92 (m, 2H), 3.89 (s, 3H), 2.16 (d, J = 20.3 Hz, 3H).

^{13}C{H} NMR (126 MHz, CDCl$_3$) δ 188.0 (d, J = 28.7 Hz), 164.3, 133.4 (d, J = 5.3 Hz), 124.2 (d, J = 3.8 Hz), 113.9 (d, J = 0.7 Hz), 108.8 (d, J = 257.0 Hz), 55.7, 27.6 (d, J = 23.4 Hz).

^{19}F NMR (470 MHz, CDCl$_3$) δ -105.03 (q, J = 20.0 Hz).

TLC: Rf = 0.26 (2% AcOEt/Hex);

$[\alpha]_D^{24}$ = -25.94 (c = 2.02, CHCl$_3$) for 74% *e.e.*

$[\alpha]_D^{24}$ = -28.87 (c = 2.00, CHCl$_3$) for 79% *e.e.*

IR ν max (cm^{-1}): 1686, 1599, 1573, 1510, 1314, 1289, 1264, 1150, 1079, 1030, 984, 892, 844, 685, 613.

HRMS (ESI) *m/z*: $[M+H]^+$ Calcd. for $C_{10}H_{10}ClFO_2^+$ 217.0426; Found: 217.0420.
HPLC (Phenomenex Cellulose 3, MeOH/H_2O = 75/25, flow rate = 0.7 mL/min, l = 220 nm) t_R = 34.271min (minor), t_R = 39.549 min (major), *e.r.* = 10:90.
HPLC (Phenomenex Cellulose 3, MeOH/H_2O = 75/25, flow rate = 0.7 mL/min, l = 220 nm) t_R = 34.545 min (minor), t_R = 39.949 min (major), *e.r.* = 13:87.

- 2-chloro-2-fluoro-1-(4-nitrophenyl) propan-1-one (**3d**)

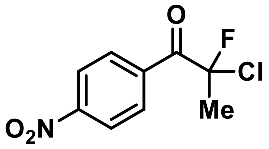

Yellow oil. Yield: 69% (24.0 mg) from **1d** and 30% (10.6 mg) from **2d**.
^{1}H NMR (500 MHz, $CDCl_3$) δ 8.37 – 8.31 (m, 4H), 2.21 (d, J = 20.3 Hz, 3H).
^{13}C{H} NMR (125 MHz, $CDCl_3$) δ 188.0 (d, J = 30.8 Hz), 150.8, 136.3 (d, J = 3.9 Hz), 131.9 (d, J = 5.2 Hz), 123.7 (d, J = 0.6 Hz), 108.3 (d, J = 255.4 Hz), 27.2 (d, J = 23.1 Hz).
^{19}F NMR (376 MHz, $CDCl_3$) δ -107.61 (q, J = 20.3 Hz).
TLC: Rf = 0.39 (2% AcOEt/Hex);
$[\alpha]_D^{24}$ = +7.22 (c = 0.41, $CHCl_3$) for 46% *e.e.*
$[\alpha]_D^{24}$ = +12.6 (c =1.11, $CHCl_3$) for 72% *e.e.*
IR ν max (cm^{-1}): 1710, 1530, 1351, 1279, 1153, 1082, 988, 896, 853, 718.
HRMS (ESI) *m/z*: $[M+H]^+$ Calcd. for $C_9H_8ClFNO_3^+$ 232.0173; Found: 232.0170.
HPLC (AD-H, n-hexane/*i*-PrOH = 99.9/0.1, flow rate = 1.0 mL/min, l = 254 nm) t_R = 7.043 min (major), t_R = 7.738 min (minor), *e.r.* = 73:27.
HPLC (AD-H, n-hexane/*i*-PrOH = 99.9/0.1, flow rate = 0.7 mL/min, l = 254 nm) t_R = 7.211 min (major), t_R = 7.971 min (minor), *e.r.* = 86:14.

- 2-chloro-1-(4-chlorophenyl)-2-fluoropropan-1-one (**3e**)

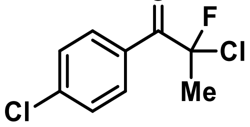

Colorless oil. Yield: 61% (13.5 mg) from **1d** and 51% (16.28 mg) from **2f**.
^{1}H NMR (500 MHz, $CDCl_3$) δ 8.37 – 8.04 (m, 2H), 7.60 – 7.40 (m, 2H), 2.17 (d, J = 20.3 Hz, 2H).

^{13}C{H} NMR (125 MHz, $CDCl_3$) δ 188.3 (d, J = 29.7 Hz), 140.8, 132.2 (d, J = 5.1 Hz), 129.8 (d, J = 3.7 Hz), 129.0, 108.5 (d, J = 256.2 Hz), 27.4 (d, J = 23.3 Hz).

^{19}F NMR (376 MHz, $CDCl_3$) δ -106.42 (q, J = 20.4 Hz).

TLC: Rf = 0.55 (2% AcOEt/Hex);

$[\alpha]_D^{24}$ = -5.52 (c = 1.59, $CHCl_3$) for 86% *e.e.*

IR ν max (cm^{-1}): 1700, 1588, 1403, 1308, 1284, 1150, 1094, 1080, 986, 892, 845, 746,670.

HRMS (ESI) *m/z*: $[M+H]^+$ Calcd. for $C_9H_8Cl_2FO^+$ 220.9931; Found: 220.9930.

HPLC (Phenomenex Cellulose 3, $MeCN/H_2O$ = 48/52, flow rate = 0.9 mL/min, l = 254 nm) t_R = 16.461 min (minor), t_R = 17.268 min (major), *e.r.* = 7:93.

HPLC (Phenomenex Cellulose 3, $MeCN/H_2O$ = 48/52, flow rate = 0.9 mL/min, l = 254 nm) t_R = 16.249 min (minor), t_R = 17.086 min (major), *e.r.* = 16:84.

- 1-(4-bromophenyl)-2-chloro-2-fluoropropan-1-one (**3f**)

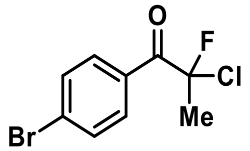

Colorless oil. Yield: 61% (16.2 mg) from **1f** and 59% (23.5 mg) from **2f**.

Purification by column chromatography in 0.5-1% EtOAc/*n*-Hex.

^{1}H NMR (500 MHz, $CDCl_3$) δ 8.06-8.03 (m, 2H), 7.65-7.62 (m, 2H), 2.17 (d, *J*= 20.3 Hz, 3H) ppm.

^{13}C{H} NMR (125 MHz, $CDCl_3$) δ 188.5 (d, *J*= 29.7 Hz), 132.4, 132.3, 132.3, 132.0, 130.2, 129.7, 108.5 (d, *J*= 256.3 Hz), 107.4, 27.4 (d, J= 23.3 Hz) ppm.

^{19}F NMR (376 MHz, $CDCl_3$) δ -106.5 (q, *J* = 20.1 Hz).

TLC: Rf = 0.88 (1% AcOEt/Hex);

$[\alpha]_D^{23}$ = -4.67 (*c* 0.5, $CHCl_3$) for 61% *e.e.*

$[\alpha]_D^{23}$ = -17.4 (*c* 0.35, $CHCl_3$) for 78% *e.e.*

IR ν max (cm^{-1}): 2955, 2924, 2854, 702, 1585, 1461, 1379, 1281, 1152, 1076, 985, 893, 738.

HRMS (ESI) *m/z*: $[M+K]^+$ Calcd. for $C_9H_7BrClFKO^+$ 302.8990; Found: 302.8981.

HPLC (Phenomenex Cellulose 3, $MeCN/H_2O$ = 50/50, flow rate = 1.0 mL/min, l = 254 nm) t_R = 14.29 min (minor), t_R = 15.34 min (major), *e.r.* = 19.5:80.5.

HPLC (Phenomenex Cellulose 3, $MeCN/H_2O$ = 50/50, flow rate = 1.0 mL/min, l =

254 nm) t_R = 13.974 min (minor), t_R = 14.865 min (major), *e.r.* = 11:89.

- 1-([1,1'-biphenyl]-4-yl)-2-chloro-2-fluoropropan-1-one (**3g**)

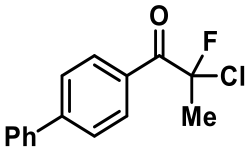

Pale yellow oil. Yield: 89% (23.4 mg) from **1h** and 65% (25.6 mg) from **2h**.
Purification by column chromatography in 2% EtOAc/*n*-Hex.

^{1}H NMR (500 MHz, $CDCl_3$) δ 8.29-8.26 (m, 2H), 7.73-7.70 (m, 2H) 7.66-7.63 (m, 2H), 7.50-7.47 (m, 2H), 7.44-7.40 (m, 1H), 2.20 (d, *J* = 20.3 Hz, 3H) ppm

^{13}C{H} NMR (125 MHz, $CDCl_3$) δ 189.0 (d, *J*= 29.2 Hz), 146.8, 139.8, 131.4 (d *J*= 6.9 Hz), 130.2, 129.8, 129.2, 128.6, 127.5, 127.2, 108.5 (d, *J*= 256.9 Hz), 27.6 (d, *J* =23.4 Hz) ppm.

^{19}F NMR (376 MHz, $CDCl_3$) δ -105.9 (q, *J* = 20.1 Hz)

TLC: Rf = 0.59 (2% AcOEt/Hex);

$[\alpha]_D^{24}$ = -17.3 (*c* = 0.4. $CHCl_3$) for 80% *e.e.*

$[\alpha]_D^{24}$ = -18.5 (c = 1.7, $CHCl_3$) for 76% *e.e.*

IR ν max (cm^{-1}): 2955, 2924, 2852, 1694, 1604, 1379, 1284, 1153, 1080, 985, 894, 854, 748, 721, 694.

HRMS (ESI) *m/z*: $[M+K]^+$ Calcd. for $C_{15}H_{12}ClFKO^+$ 301.0198; Found: 301.0189.

HPLC (Phenomenex Cellulose 3, MeCN/H_2O = 50/50, flow rate = 1.0 mL/min, l = 220 nm) t_R = 40.15 min (minor), t_R = 48.98 min (major), *e.r.* = 10:90.

HPLC (Phenomenex Amylose 2, n-hexane, flow rate = 0.7 mL/min, l = 220 nm) t_R = 15.194 min (minor), t_R = 24.266 min (major), *e.r.* = 12:88.

- 2-chloro-1-(2-chlorophenyl)-2-fluoropropan-1-one (**3h**)

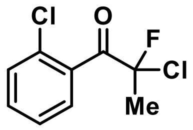

Colorless oil. Yield: 19% (4.2 mg) from **1j** and 19% (6.28 mg) from **2i**.

^{1}H NMR (500 MHz, $CDCl_3$) δ 7.65 (m, 1H), 7.50 – 7.40 (m, 2H), 7.39 – 7.33 (m, 1H), 2.20 (d, J = 19.4 Hz, 3H).

^{13}C{H} NMR (125 MHz, $CDCl_3$) δ 193.4 (d, J = 33.0 Hz), 134.5, 132.4, 132.1, 130.4,

129.2 (d, J = 4.3 Hz), 126.5, 107.9 (d, J = 256.0 Hz), 26.9 (d, J = 22.9 Hz).
^{19}F NMR (376 MHz, $CDCl_3$) δ -108.70 (q, J = 19.4 Hz).
TLC: Rf = 0.42 (2% AcOEt/Hex);
$[\alpha]_D^{24}$ = +23.7 (c = 0.38, $CHCl_3$) for 70% *e.e.*
IR ν max (cm^{-1}): 1733, 1436, 1378, 1285, 1163, 1078, 1036, 983, 762, 733, 732, 642.
HRMS (ESI) *m/z*: $[M+H]^+$ Calcd. for $C_9H_8Cl_2FO^+$ 220.9931; Found: 220.9932.
HPLC (AD-H, n-hexane, flow rate = 0.5 mL/min, l = 220 nm) t_R = 13.439 min (major), t_R = 14.728 min (minor), *e.r.* = 85:15.
HPLC (AD-H, n-hexane, flow rate = 0.5 mL/min, l = 220 nm) t_R = 13.069 min (major), t_R = 15.124 min (minor), *e.r.* = 69.5:30.5.

- 2-chloro-2-fluoro-1-(naphthalen-2-yl) propan-1-one (**3i**)

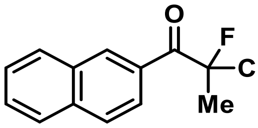

Pale yellow oil/white solid. Yield: 90% (21.3 mg) from **1i** and 51% (18.1 mg) from **2i**. Purification by column chromatography in 2% EtOAc/hexanes
^{1}H NMR (500 MHz, $CDCl_3$) δ 8.81 (s, 1H), 8.16 (dt, *J* = 8.7, 1.5 Hz, 1H) 8.01-7.99 (m, 1H), 7.92-7.88 (m, 2H), 7.65-7.62 (m, 1H), 7.59-7.56 (m, 1H), 2.24 (d, *J* = 20.3 Hz, 3H) ppm.
^{13}C{H} NMR (125 MHz, $CDCl_3$) δ 189.4 (d, *J*= 29.2 Hz), 136.0, 133.4 (d, *J*= 6.9 Hz), 130.2, 129.3, 128.4, 127.9, 127.0, 125.7 (d, *J*= 3.4 Hz), 124.6, 108.8 (d, *J*= 256.8 Hz), 27.7 (d, *J*=23.1 Hz) ppm.
^{19}F NMR (376 MHz, $CDCl_3$) δ -105.4 (q, *J*=20.1 Hz).
TLC: Rf = 0.85 (2% AcOEt/Hex);
MP = 111-112 °C
$[\alpha]_D^{24}$ = -9.5 (*c* 0.4, $CHCl_3$) for 58% *e.e.*
$[\alpha]_D^{24}$ = -31.9 (c = 1.1, $CHCl_3$) for 82% *e.e.*
IR ν max (cm^{-1}): 2955, 2922, 2852, 1737, 1696, 1461, 1377, 1151, 1122, 1082, 1023.
HRMS (ESI) *m/z*: $[M+K]^+$ Calcd. for $C_{13}H_{10}ClFKO^+$ 275.0036; Found: 275.0039.
HPLC (Phenomenex Cellulose 3, MeCN/H_2O = 50/50, flow rate = 1.0 mL/min, l = 220 nm) t_R = 21.69 min (major), t_R = 22.64 min (minor), *e.r.* = 79:21.

HPLC (Phenomenex Cellulose 3, MeCN/H_2O = 50/50, flow rate = 1.0 mL/min, l = 220 nm) t_R = 79.253 min (major), t_R = 81.192 min (minor), *e.r.* = 91:9.

- (E)-4-chloro-4-fluoro-1-phenylpent-1-en-3-one (**3j**)

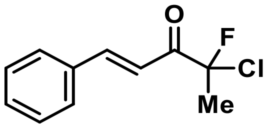

Colorless oil. Yield: 79% (16.8 mg) from **1k**.

^{1}H NMR (500 MHz, $CDCl_3$) δ 7.89 (d, J = 15.9 Hz, 1H), 7.66 – 7.61 (m, 2H), 7.46 – 7.41 (m, 3H), 7.24 (dd, J = 15.9, 2.7 Hz, 1H), 2.08 (d, J = 19.8 Hz, 3H).

^{13}C{H} NMR (125 MHz, $CDCl_3$) δ 188.5 (d, J = 29.0 Hz), 147.6 (d, J = 1.8 Hz), 134.1, 131.3, 128.9 (d, J = 16.0 Hz), 117.3, 108.1 (d, J = 254.2 Hz), 25.9 (d, J = 22.9 Hz).

^{19}F NMR (376 MHz, $CDCl_3$) δ -111.26 – -111.54 (m, 1F).

TLC: Rf = 0.6 (2% AcOEt/Hex);

$[\alpha]_D^{24}$ = +9.7 (c = 1.94, $CHCl_3$) for 40% *e.e.*

IR ν max (cm^{-1}): 2923, 1633, 1527, 1494, 1448, 1360,1253, 1185, 1157, 1144, 1082, 1039,976, 928, 921,859, 847, 814, 762,748, 698, 661,649.

HRMS (ESI) *m/z*: $[M+H]^+$ Calcd. for $C_{11}H_{11}ClFO^+$ 213.0477; Found: 213.0474.

UPLC (Trefoil Celullose 1, i-PrOH/CO_2 = 0.5/99.5, flow rate = 1.0 mL/min, l = 254 nm) t_R = 1.89 min (minor), t_R = 2.01 min (major), *e.r.* = 70:30.

- 1-(4-(tert-butyl) phenyl)-2-chloro-2-fluoropropan-1-one (**3k**)

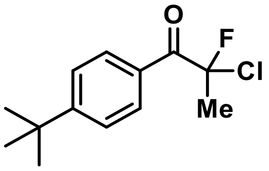

Colorless oil. Yield: 75% (18.2 mg) from **1g**.

^{1}H NMR (500 MHz, $CDCl_3$) δ 8.17 – 8.09 (m, 2H), 7.50 (d, J = 8.8 Hz, 2H), 2.17 (d, J = 20.2 Hz, 3H), 1.35 (s, 3H).

^{13}C{H} NMR (125 MHz, $CDCl_3$) δ 188.9 (d, J = 29.0 Hz), 157.9, 130.7 (d, J = 5.0 Hz), 125.5, 108.5 (d, J = 256.7 Hz), 35.2, 30.9, 27.4 (d, J = 23.4 Hz).

^{19}F NMR (376 MHz, $CDCl_3$) δ -105.49 – -105.98 (m).

TLC: Rf = 0.6 (2% AcOEt/Hex);

$[\alpha]_D^{24}$ = +15.7 (c = 0.35, $CHCl_3$) for 76% ee.

IR ν max (cm^{-1}): 2965, 2906, 2871, 1693, 1605, 1411,1379, 1366, 1286, 1272, 1164, 1151,1109, 1081, 986, 894, 851, 754, 679.

HRMS (ESI) *m/z*: $[M+H]^+$ Calcd. for $C_{13}H_{17}ClFO^+$ 243.0946; Found: 243.0946.

UPLC (Trefoil Celullose 1, i-PrOH/CO_2 = 0.1/99.9, flow rate = 1.0 mL/min, l = 254 nm) t_R = 2.34 min (major), t_R = 2.50 min (minor), *e.r.* = 88:12.

- 2-chloro-2-fluoro-1-phenylpentan-1-one (**3l**)

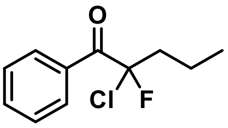

Pale yellow oil. Yield: 47% (10,0 mg) from **1l**.

^1H NMR (400 MHz, $CDCl_3$) δ 8.14 (dt, J = 8.6, 1.5 Hz, 2H), 7.66 – 7.57 (m, 1H), 7.48 (dd, J = 8.4, 7.2 Hz, 2H), 2.57 – 2.30 (m, 2H), 1.82 – 1.39 (m, 2H), 1.01 (t, J = 7.4 Hz, 3H).

$^{13}C\{H\}$ NMR (100 MHz, $CDCl_3$) δ 190.1, 189.8, 133.8, 132.1, 130.5, 130.4, 128.4, 111.7, 109.1, 41.6, 41.4, 16.6, 16.6, 13.7.

^{19}F NMR (376 MHz, $CDCl_3$) δ -114.74 (t, J = 21.1 Hz).

TLC: Rf = 0.81 (2% AcOEt/Hex);

$[\alpha]_D^{24}$ = +13.3 (c = 0.6, $CHCl_3$) for 84% *e.e.*

IR ν max (cm^{-1}): 3417, 2957, 2925, 2854, 1739, 1698, 1461, 1379, 1260, 1096, 1028, 804.

HRMS (ESI) *m/z*: $[M+H]^+$ Calcd. for $C_{11}H_{13}ClFO^+$ 215.0633; Found: 215.0634.

UPLC (Trefoil Celullose 1, i-PrOH/CO_2 = 0.5/99.5, flow rate = 1.0 mL/min, l = 254 nm) t_R = 1.49 min (major), t_R = 2.50 min (minor), *e.r.* = 92:8.

- 2-chloro-2-fluoro-1-(naphthalen-2-yl) pentan-1-one (**3m**)

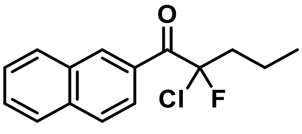

Pale yellow oil. Yield: 44% (12 mg) from **1m**.

^1H NMR (400 MHz, $CDCl_3$) δ 8.76 (d, J = 2.0 Hz, 1H), 8.13 (dt, J = 8.7, 1.6 Hz, 1H), 7.99 (dd, J = 8.2, 1.3 Hz, 1H), 7.89 (dd, J = 10.1, 8.3 Hz, 2H), 7.67 – 7.48 (m, 2H), 2.59 – 2.35 (m, 2H), 1.82 – 1.60 (m, 2H), 1.03 (t, J = 7.4 Hz, 3H).

$^{13}C\{H\}$ NMR (100 MHz, $CDCl_3$) δ 190.0, 189.7, 135.8, 132.9, 132.8, 132.2, 130.0,

129.3, 129.2, 129.1, 128.2, 127.7, 126.8, 125.4, 125.4, 111.9, 109.4, 41.7, 41.5, 31.9, 29.3, 22.7, 16.69, 16.66, 14.1, 13.8.

^{19}F NMR (376 MHz, $CDCl_3$) δ -114.00 (t, J = 21.3 Hz).

TLC: Rf = 0.85 (2% AcOEt/Hex);

$[\alpha]_D^{24}$ = +7.3 (c = 0.4, $CHCl_3$) for 84% *e.e.*

IR ν max (cm^{-1}): 3061, 2963, 2928, 2875, 1692, 1628, 1597, 1541, 1465, 1357, 1278, 1199, 1119, 1032, 933, 824, 750.

HRMS (ESI) *m/z*: $[M+Na]^+$ Calcd. for $C_{15}H_{14}ClFNaO^+$ 287.0609; Found: 287.0604.

UPLC (Trefoil Celullose 1, i-PrOH/CO_2 = 0.5/99.5, flow rate = 1.0 mL/min, l = 254 nm) t_R = 5.02 min (minor), t_R = 5.20 min (major), *e.r.* = 92:8.

- 2-chloro-2-fluoro-1-(p-tolyl) propan-1-one (**3n**)

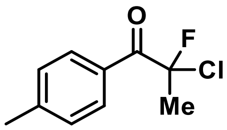

Colorless oil. Yield: 63% (19 mg) from **2e**.

Purification by column chromatography in 0.2-1% EtOAc/hexanes.

^{1}H NMR (500 MHz, $CDCl_3$) δ 8.10-8.07 (m, 2H), 7.30-7.27 (m, 2H), 2.43 (s, 3H), 2.16 (d, *J* = 20.2 Hz, 3H) ppm.

^{13}C{H} NMR (125 MHz, $CDCl_3$) δ 189.1 (d, *J*= 29.1 Hz), 145.3, 131.0, 131.0, 129.4, 129.4, 129.0, 128.9, 108.7 (d, *J* = 256.8 Hz), 27.6 (d, *J* =23.4 Hz) 21.9 ppm.

^{19}F NMR (376 MHz, $CDCl_3$) δ -105.8 (q, *J* =20.2 Hz) ppm.

TLC: Rf = 0.83 (1% AcOEt/hexanes).

$[\alpha]_D^{24}$ = 4.92 (*c* 0.1, $CHCl_3$) for 76% *e.e.*

IR ν max (cm^{-1}): 2955, 2922, 2869, 2852, 1461, 1377.

HRMS (ESI) *m/z*: $[M+K]^+$ Calcd. for $C_{10}H_{10}ClFKO^+$ 239.0041; Found: 239.0050.

HPLC (Phenomenex Cellulose 4, n-hexane/*i*-PrOH = 99.8/0.2, flow rate = 1.0 mL/min, l = 254 nm) t_R = 5.771 min (major), t_R = 6.270 min (minor), *e.r.* = 64:36.

- 2-bromo-2-chloro-1-phenylpropan-1-one (**4a**)

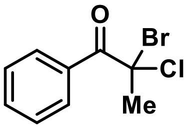

Pale yellow oil. Yield: 51% (13.7 mg) from **1a**.

^{1}H NMR (500 MHz, $CDCl_3$) δ 8.39 – 8.32 (m, 2H), 7.60 (d, J = 7.4 Hz, 1H), 7.54 – 7.45 (m, 2H), 2.56 (s, 3H).

^{13}C{H} NMR (125 MHz, $CDCl_3$) δ 188.4, 133.5, 131.5, 131.3, 128.0, 71.2, 36.0.

TLC: Rf = 0.6 (2% AcOEt/Hex);

$[\alpha]_D^{24}$ = +63.4 (c = 0.16, $CHCl_3$) for 92% *e.e.*

IR ν max (cm^{-1}): 2955, 2925, 2854, 1691, 1614, 1598,1528, 1461, 1378, 1255, 1187, 1131,1033, 934, 806, 764.

HRMS: We exhaustively tried different conditions and instruments, but we could not observe the molecular ion for this compound.

UPLC (Trefoil Amylose 1, *i*-PrOH/CO_2 = 0.5/99.5, flow rate = 1.0 mL/min, l = 254 nm) t_R = 2.21 min (minor), t_R = 2.89 min (major), *e.r.* = 04:96.

- 1-([1,1'-biphenyl]-4-yl)-2-bromo-2-chloropropan-1-one (**4b**)

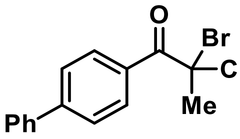

Colorless oil. Yield: 70% (22.6 mg) from **1h**.

Purification by preparative TLC in 5-10% ACOEt/*n*-Hex.

^{1}H NMR (400 MHz, $CDCl_3$) δ 8.46 (d, J = 8.6 Hz, 2H), 7.73 – 7.69 (m, 2H), 7.68 – 7.65 (m, 2H), 7.50 (t, J = 7.5 Hz, 2H), 7.44 (d, J = 7.3 Hz, 1H), 2.58 (s, 3H) ppm.

^{13}C NMR (101 MHz, $CDCl_3$) δ 187.89, 146.22, 139.68, 131.95, 130.08, 128.99, 128.42, 127.31, 126.64, 71.29, 36.00 ppm.

IR ν max (cm^{-1}): 2955, 2923, 2866, 2854, 1682, 1604,1405, 1379, 1258, 1072, 852, 748, 684.

(GCMS+EI) m/z: calc. for $C_{15}H_{13}BrClO$ $[M+H]^+$: 322; found: 322.

HRMS: We exhaustively tried different conditions and instruments, but we could not observe the molecular ion for this compound.

5.2 ALFA-ARYL CARBONYL PRODUCTS (3')

The molecules described below are non-volatile and can be dried in high vacuum.

- Ethyl 2-chloro-2-fluoro-2-phenylacetate (**3'a**)

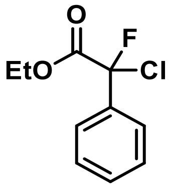

Colorless oil. Yield: 48% (14.9 mg) from **2'b**.

Purification by column chromatography in 1% EtOAc/*n*-Hex.

^{1}H NMR (400 MHz, $CDCl_3$) δ 7.71 – 7.62 (m, 2H), 7.47 – 7.39 (m, 3H), 4.37 – 4.22 (m, 2H), 1.29 (t, J = 7.1 Hz, 3H).

^{13}C{H} NMR (100 MHz, $CDCl_3$) δ 165.4 (d, J = 30.5 Hz), 136.2 (d, J = 22.7 Hz), 130.5, 128.6, 125.9 (d, J = 7.5 Hz), 104.5 (d, J = 254.1 Hz), 63.7, 13.9.

^{19}F NMR (470 MHz, $CDCl_3$) δ -112.2.

TLC: Rf = 0.33 (2% AcOEt/Hex);

$[\alpha]_D^{24}$ = +16.8 (c = 0.50, $CHCl_3$) for 22% *e.e.*

IR ν max (cm^{-1}): 2925, 1760, 1450, 1259, 1224, 1115, 1094, 1071, 1020, 903, 744, 693, 641.

HPLC (Phenomenex Amylose 2, n-hexane, flow rate = 1.0 mL/min, l = 220 nm) t_R = 12.890 min (major), t_R = 15.714 min (minor), *e.r.* = 61:39.

The spectroscopic data are in agreement with those reported in the literature.[16]

- Benzyl 2-chloro-2-fluoro-2-phenylacetate (**3'b**)

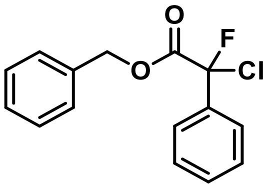

Colorless oil. Yield: 50% (21 mg) from **2'd**.

Purification by column chromatography in 1% EtOAc/*n*-Hex.

^{1}H NMR (500 MHz, $CDCl_3$) δ 7.67-7.64 (m, 2H), 7.44-7.39 (m, 3H), 7.35-7.32 (m, 3H), 7.29-7.26 (m, 2H), 5.28 (d, J=12.3Hz, 1H), 5.24(d, J= 12.3 Hz, 1H) ppm.

^{13}C{H} NMR (125 MHz, $CDCl_3$) δ 165.3 (d, J=30.9 Hz), 136.0 (d, J = 22.0 Hz), 130.6 (d, J = 1.4 Hz), 128.8, 128.8, 128.6, 128.6, 128.1, 126.0, 125.9, 104.6 (d, J = 254.3 Hz), 69.0 ppm.

^{19}F NMR (376 MHz, $CDCl_3$) δ -112.1 ppm.

TLC: Rf = 0.53 (2% AcOEt/Hex);

$[\alpha]_D^{24}$ = +11.1 (c 0.3, $CHCl_3$) for 42% *e.e.*

IR ν max (cm^{-1}): 2921, 1759, 1450, 1256, 1224, 1111, 1092, 1071, 1030, 988, 799, 742, 692.

HRMS (ESI) *m/z*: $[M+K]^+$ Calcd. for $C_{15}H_{12}ClFKO_2^+$ 317.0147; Found: 317.0151.

HPLC (AD-H, *n*-Hexanes/i-PrOH = 99/1, flow rate = 1.0 mL/min, l = 220 nm) t_R = 11.415 min (minor), t_R = 12.137 min (major), *e.r.* = 29:71.

- isobutyl 2-chloro-2-fluoro-2-phenylacetate (**3'c**)

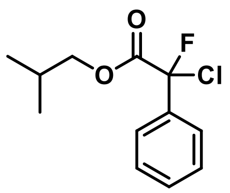

Colorless oil. Yield: 49% (18.3 mg) from **2'e**.

Purification by column chromatography in 1% EtOAc/*n*-Hex.

^{1}H NMR (500 MHz, $CDCl_3$) δ 7.68-7.66 (m, 2H), 7.45-7.42 (m, 3H), 4.07-3.98 (m, 2H), 1.97 (dt, *J* = 13.4, 6.7 Hz, 1H), 0.89 (d, *J* = 1.7 Hz, 3H), 0.88 (d, *J* = 1.7 Hz, 3H) ppm.

^{13}C{H} NMR (125 MHz, $CDCl_3$) δ 165.5 (d, *J*= 30.8 Hz), 136.3 (d, *J*= 23.2 Hz), 131.0, 130.5, 129.1, 129.0, 128.6, 128.6, 126.0, 125.9, 104.6 (d, *J*=253.4 Hz), 73.3, 68.3, 27.8, 18.9 ppm.

^{19}F NMR (376 MHz, $CDCl_3$)) δ -112.2.

TLC: R_f = 0.88 (2% AcOEt/Hex);

$[\alpha]_D^{24}$ = +11.06 (c = 0.94, $CHCl_3$) for 12% *e.e.*

IR ν max (cm^{-1}): 2959, 2926, 2873, 2854, 1764, 1466, 1453, 1377, 1263, 1225, 1116, 1094, 1072, 1008, 747, 693.

HRMS (ESI) *m/z*: $[M+K]^+$ Calcd. for $C_{12}H_{14}ClFKO_2^+$ 283.0303; Found: 283.0310.

HPLC (Phenomenex Cellulose 4, *n*-Hexanes/i-PrOH = 99/1, flow rate = 0.7 mL/min, l = 254 nm) t_R = 5.36 min (major), t_R = 7.78 min (minor), *e.r.* = 56:44.

- 4-chloro-4-fluoroisochroman-3-one (**3’d**)

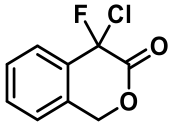

Colorless oil. Yield: 74% (22.1 mg) from **2’f**.

Purification by column chromatography in 1% EtOAc/*n*-Hex.

^{1}H NMR (500 MHz, $CDCl_3$) δ 7.81 – 7.76 (m, 1H), 7.58 – 7.50 (m, 2H), 7.33 (dd, J = 8.4, 3.7 Hz, 1H), 5.65 (d, J = 14.4 Hz, 1H), 5.35 (d, J = 14.4 Hz, 1H).

^{13}C{H}NMR (125 MHz, $CDCl_3$) δ 162.4 (d, J = 24.4 Hz), 132.4 (d, J = 22.6 Hz), 131.2, 131.1 (d, J = 5.8 Hz), 129.9, 125.2 (d, J = 1.8 Hz), 124.1 (d, J = 6.4 Hz), 94.8 (d, J = 257.9 Hz), 69.8.

^{19}F NMR (470 MHz, $CDCl_3$) δ -122,22.

TLC: Rf = 0.32 (2% AcOEt/Hex);

$[\alpha]_D^{24}$ = -3.33 (c = 0.42, $CHCl_3$) for 40% *e.e.*

IR ν max (cm^{-1}): 1771, 1458, 1392, 1261, 1245, 1196, 1140, 1084, 1028, 942, 888, 862, 754, 709, 633.

HRMS (ESI) *m/z*: $[M+H]^+$ Calcd. for $C_9H_7ClFO_2^+$ 201.0113; Found: 201.0113.

HPLC (Phenomenex Amylose 2, *n*-Hexanes/i-PrOH = 90/10, flow rate = 1.0 mL/min, l = 220 nm) t_R = 10.179 min (minor), t_R = 12.234 min (major), *e.r.* = 30:70.

- 2-chloro-2-fluoro-1,2-diphenylethan-1-one (**3’e**)

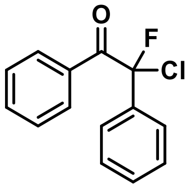

Colorless oil. Yield: 78% (29.0 mg) from **2’g**.

Purification by column chromatography in 1% EtOAc/*n*-Hex.

^{1}H NMR (500 MHz, $CDCl_3$) δ 7.98 (m, 2H), 7.68 – 7.61 (m, 2H), 7.59 – 7.53 (m, 1H),7.49 – 7.38 (m, 5H).

^{13}C{H} NMR (125 MHz, $CDCl_3$) δ 189.0 (d, J = 29.2 Hz), 136.6 (d, J = 22.8 Hz), 134.0, 132.3 (d, J = 2.9 Hz), 130.8 (d, J = 4.4 Hz), 130.5 (d, J = 1.5 Hz), 128.8, 128.6, 126.0 (d, J = 6.7 Hz), 110.0 (d, J = 255.9 Hz).

^{19}F NMR (376 MHz, $CDCl_3$) δ -106.70.

TLC: Rf = 0.48 (2% AcOEt/Hex);

$[\alpha]_D^{24}$ = -2.17 (c = 0.96, $CHCl_3$) for 38% *e.e.*

IR ν max (cm^{-1}): 3063, 2925, 2853, 1701, 1596, 1448, 1237, 1215, 1185, 876, 835, 817, 743, 693, 642, 631.

HPLC (AD-H, *n*-Hexanes/i-PrOH = 99.5/0.5, flow rate = 1.0 mL/min, l = 254 nm) t_R = 6.120 min (minor), t_R = 6.624 min (major), *e.r.* = 31:69.

The spectroscopic data are in agreement with those reported in the literature.[17]

- 4-(1-chloro-2-(4-chlorophenyl)-1-fluoro-2-oxoethyl)benzonitrile (**3'f**)

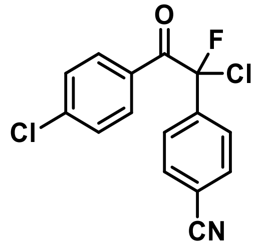

Yellow solid. Yield: 60% (27.6 mg) from **2'h**.

Purification by column chromatography in 1% EtOAc/*n*-Hex.

^{1}H NMR (500 MHz, $CDCl_3$) δ 8.08 – 7.99 (m, 1H), 7.82 – 7.67 (m, 2H), 7.50 – 7.44 (m, 1H).

^{13}C{H} NMR (125 MHz, $CDCl_3$) δ 186.9 (d, J = 30.2 Hz), 141.4, 140.8 (d, J = 23.1 Hz),132.4, 132.2 (d, J = 5.2 Hz), 129.8 (d, J = 3.8 Hz), 129.3, 127.2 (d, J = 7.7 Hz), 118.0,114.6 (d, J = 1.5 Hz), 108.2 (d, J = 259.0 Hz).

^{19}F NMR (470 MHz, $CDCl_3$) δ -113.03.

MP = 85-88°C.

TLC: Rf = 0.34 (2% AcOEt/Hex);

$[\alpha]_D^{24}$ = +15.86 (c = 0.66 $CHCl_3$) for 42% *e.e.*

IR ν max (cm^{-1}): 2923, 2232, 1701, 1587, 1402, 1216, 1097, 1014, 892, 861, 838, 802, 734.

HRMS (ESI) *m/z*: $[M+H]^+$ Calcd. for $C_{15}H_9Cl_2FNO^+$ 308.0040; Found: 308.0038.

HPLC (AD-H, *n*-Hexanes/i-PrOH = 90/10, flow rate = 1.0 mL/min, l = 254 nm) t_R = 10.380 min (major), t_R = 13.970 min (minor), *e.r.* = 71:29.

- phenyl 2-chloro-2-fluoro-2-(p-tolyl) acetate (**3’g**)

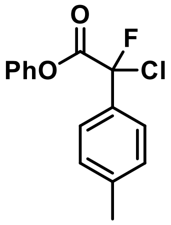

Colorless oil. Yield: 67% (28 mg) from 2’i.

Purification by column chromatography in 1% EtOAc/*n*-Hex.

^{1}H NMR (500 MHz, $CDCl_3$) δ 7.67-7.64 (m, 2H), 7.40-7.34(m, 2H) 7.29-7.25 (m, 3H), 7.10-7.08 (m, 2H), 2.41 (s, 3H) ppm.

^{13}C{H} NMR (125 MHz, $CDCl_3$) δ 164.0 (d, *J* = 32.0 Hz), 150.4, 133.0, 132.7, 130.4, 129.9 (d, *J* = 9.2 Hz), 129.8, 126.8, 126.0 (d, *J* = 6.9 Hz), 121.4, 120.9, 104.6 (d, *J* = 254.1 Hz), 21.4 ppm **.**

^{19}F NMR (376 MHz, CDCl3) δ -111.2.

TLC: R_f = 0.50 (2% AcOEt/Hex).

$[\alpha]_D^{24}$ = 5.96 (*c* 0.2, $CHCl_3$) for 34% *e.e.*

IR ν max (cm^{-1}): 2956, 2924, 2853, 1777, 1606, 1491, 1460, 1377, 1233, 1187, 1164, 1088, 1025, 971, 909, 813, 740, 689.

HRMS (ESI) *m/z*: $[M+K]^+$ Calcd. for $C_{15}H_{12}ClFKO_2^+$ 317.0147; Found: 317.0151.

HPLC (Phenomenex Cellulose 3, MeCN/H_2O = 48/52, flow rate = 0.9 mL/min, l = 254 nm) t_R = 40.94 min (major), t_R = 55.91 min (minor), *e.r.* = 67:33.

- phenyl 2-chloro-2-fluoro-2-(4-nitrophenyl) acetate (**3’h**)

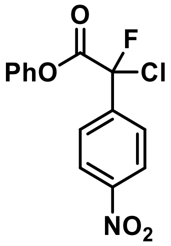

Yellow solid. Yield: 40% (18.6 mg) from **2’j**.

Purification by column chromatography in 2-3% EtOAc/*n*-Hex.

^{1}H NMR (500 MHz, $CDCl_3$) δ 8.38-8.35 (m, 2H), 8.01-7.98 (m, 2H) 7.44-7.40 (m, 2H), 7.32-7.29 (m, 1H), 7.13-7.09 (m, 2H) ppm.

^{13}C{H} NMR (126 MHz, $CDCl_3$) δ 162.9 (d, *J* = 30.8 Hz), 150.1, 141.9 (d, *J* = 23.6 Hz), 129.9, 127.55 (d, *J* = 7.5 Hz), 127.2, 124.0, 124.0, 120.7, 103.1 (d, *J* = 256.2

Hz) ppm.

^{19}F NMR (376 MHz, $CDCl_3$) δ -114.8.

TLC: R_f = 0.50 (2% AcOEt/Hex);

$[\alpha]_D^{24}$ = -9.9 (*c* 0.3, $CHCl_3$) for 50% *e.e.*

IR ν max (cm^{-1}): 2956, 2925, 2853, 1758, 1529, 1463, 1377, 1351, 1297, 1268, 1222, 1125, 1092, 1016, 988, 981, 915, 851, 738, 721, 697.

HRMS (ESI) *m/z*: $[M+K]^+$ Calcd. for $C_{14}H_9ClFKNO_4^+$ 347.9841; Found: 347.9839.

HPLC (Phenomenex Cellulose 3, MeCN/H_2O = 48/52, flow rate = 0.9 mL/min, l = 254 nm) t_R = 30.95 min (major), t_R = 37.69 min (minor), *e.r.* = 75:25.

6. SINGLE CRYSTAL X-RAY DIFFRACTION (SCXRD)

Compounds **1h** and **4b** were crystallized by the same method: they were dissolved in a minimal amount of dichloromethane (DCM) in a small vial. The vial was placed inside a larger sealed container, into which hexane was added without contacting the solution. The system was sealed and left undisturbed at room temperature, allowing slow vapor diffusion of hexane into the DCM solution until crystal growth was observed.

Experimental and refinement details are presented in Table S5 for both compounds (**1h** and **4b**).

Table S5. Experimental details for single-crystal X-ray diffraction experiment.

	**1h**	**4b**
Crystal data		
Chemical formula	$C_{24}H_{25}NO_2S$	$C_{15}H_{12}BrClO$
M_r	391.51	323.61
Crystal system, space group	Monoclinic, $P2_1$	Monoclinic, $P2_1/n$
Temperature (K)	100	210
a, *b*, *c* (Å)	7.3271 (3), 6.9571 (4), 20.1400 (12)	5.9979 (1), 30.1680 (4), 7.2583 (1)
β (°)	90.385 (5)	91.721 (1)
V (Å^3)	1026.62 (9)	1312.76 (3)
Z	2	4
Radiation type	Cu *K*α	
μ (mm^{-1})	1.54	6.00
Crystal size (mm)	0.18 × 0.11 × 0.03	0.16 × 0.08 × 0.05
Data collection		
Diffractometer	XtaLAB Synergy, Dualflex, HyPix	

Absorption correction	Gaussian<br>*CrysAlis PRO* 1.171.43.118a (Rigaku Oxford Diffraction, 2024) Numerical absorption correction based on gaussian integration over a multifaceted crystal model Empirical absorption correction using spherical harmonics, implemented in SCALE3 ABSPACK scaling algorithm.	
T_{min}, T_{max}	0.768, 1.000	0.428, 1.000
No. of measured, independent and observed [$I > 2\sigma(I)$] reflections	10876, 4054, 3881	14973, 2837, 2555
R_{int}	0.044	0.042
$(\sin \theta/\lambda)_{max}$ (Å^{-1})	0.638	0.638
Refinement		
$R[F^2 > 2\sigma(F^2)]$, $wR(F^2)$, S	0.063, 0.162, 1.07	0.033, 0.097, 1.11
No. of reflections	4054	2837
No. of parameters	259	183
No. of restraints	1	2
H-atom treatment	H-atom parameters constrained	H-atom parameters constrained
$\Delta\rho_{max}$, $\Delta\rho_{min}$ (e Å^{-3})	0.46, −0.65	0.34, −0.50
Absolute structure	Flack x determined using 1472 quotients [(I+)-(I-)]/[(I+)+(I-)] (Parsons, Flack and Wagner, Acta Cryst. B69 (2013) 249-259).	not applicable
Absolute structure parameter	−0.006 (15)	not applicable

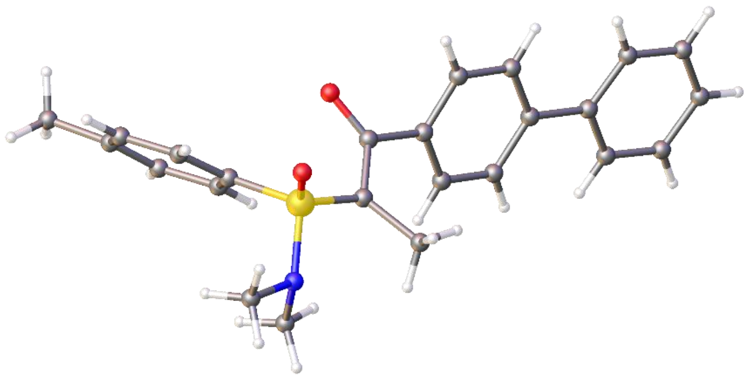

Figure S1. Asymmetric unit for **1h**. Ellipsoids drawn at 50% probability level.

The crystal structure for **1h** indicates the presence of the pure R-enantiomer, crystallizing in space group P2_1. The structure is twinned, with the second component accounting for 8.5% of the crystal structure. However, given that the only operation for the twinned component is a rotation around the *a* axis (x,$\bar{y},\bar{z}$), the molecule crystallized in an enantiomorphic pure manner.

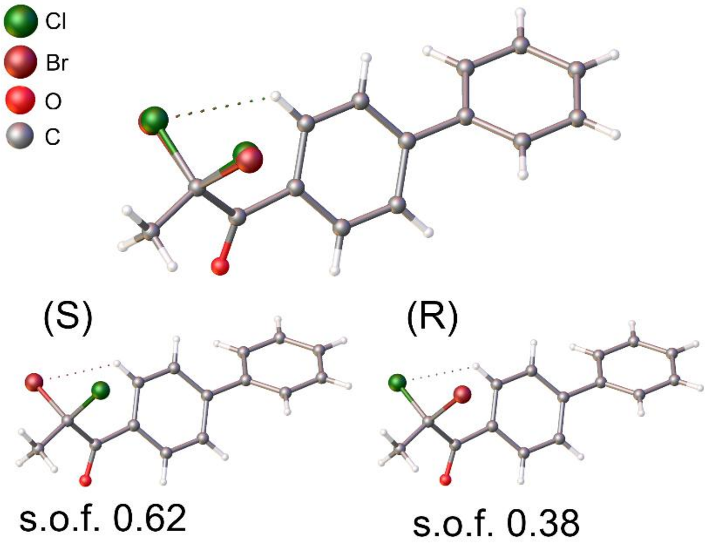

Figure S2. Asymmetric unit for **4b** and the representation of the unique molecules composing the disordered molecule. Ellipsoids drawn at 50% probability level.

The structure for **4b** is composed of both enantiomers, *R* and *S*, due to a disorder on the halogen atoms. The disorder causes the percentage of each enantiomer to be around 62% for the *S*-enantiomer and around 38% for the *R*-enantiomer. However, given the structural similarity between both enantiomers, they crystallize in the centrosymmetric space group P2_1/*n*. Chiral structures usually

crystallize in non-centrosymmetric space groups, however, in this case, the crystallization in a centrosymmetric space group (Figure S3), along with the enantiomeric disorder, indicates that it is a solid solution of enantiomers, with a random distribution of enantiomers.[18] About only 2% of racemic mixtures crystallize as enantiomeric solid solutions, so there is much yet to understand about the processes that cause this type of crystallization.[19]

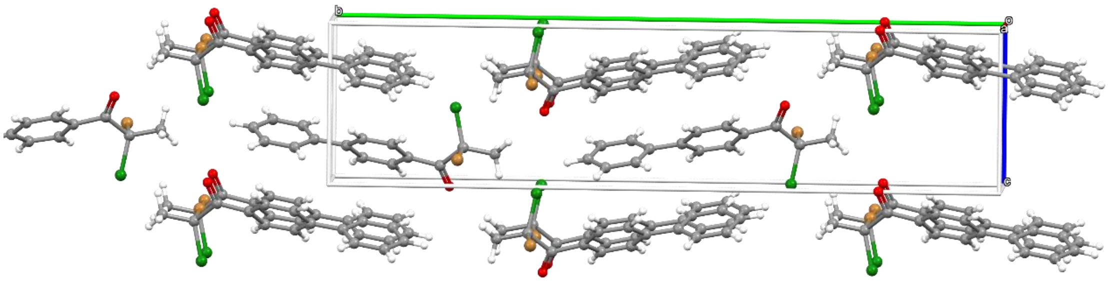

Figure S3. Packing of **3g**, showing only the molecule with higher occupancy in the asymmetric unit (*S*). The centrosymmetric space group presents a glide plane, so in the packing, the molecule and its mirror image are packed together.

7. CHROMATOGRAMS OF RACEMIC AND ENANTIOENRICHED COMPOUNDS

Chromatogram of racemic molecule **SM-B1**:

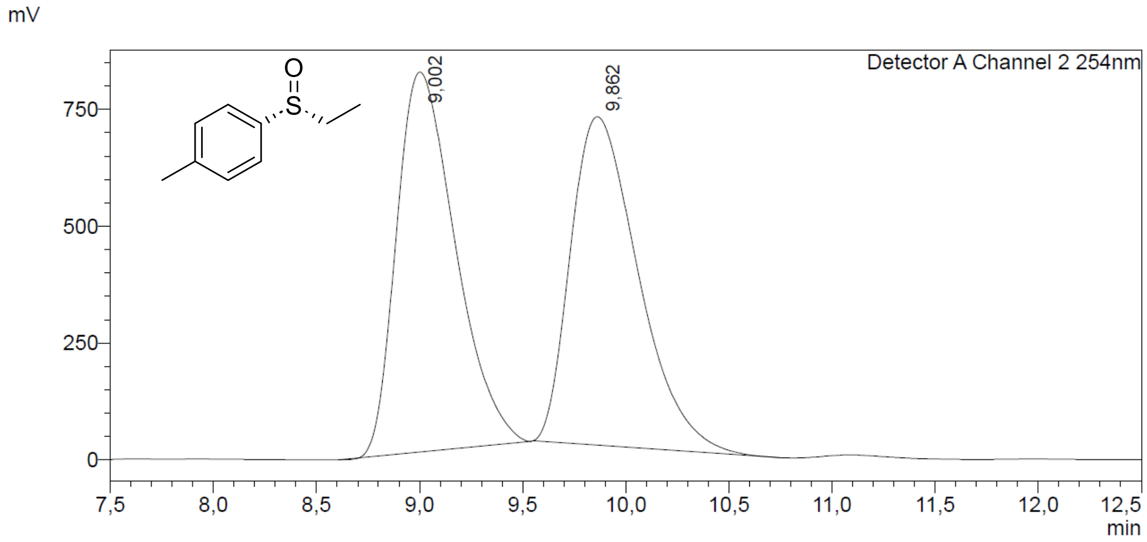

Detector A Channel 2 254nm

Peak#	Ret. Time	Area	Height	Conc.	Unit	Mark	Name
1	9,002	16142039	813322	50,088		M	
2	9,862	16085425	703335	49,912		M	
Total		32227464	1516657				

Chromatogram of enantioenriched molecule **SM-B1**:

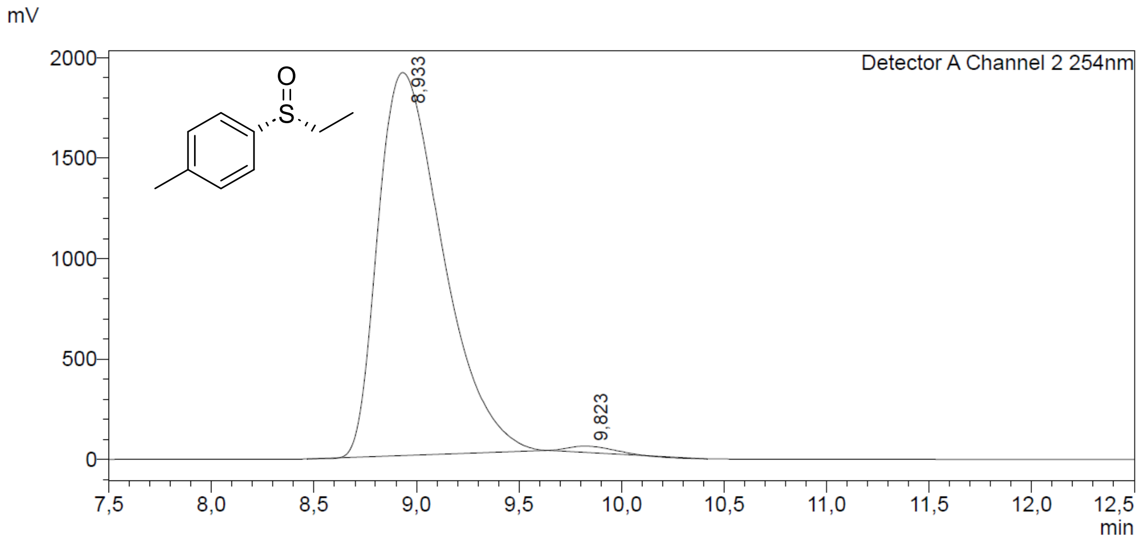

Detector A Channel 2 254nm

Peak#	Ret. Time	Area	Height	Conc.	Unit	Mark	Name
1	8,933	41997693	1907220	99,005		M	
2	9,823	422274	31009	0,995		M	
Total		42419967	1938229				

Chromatogram of racemic molecule **SM-B2**:

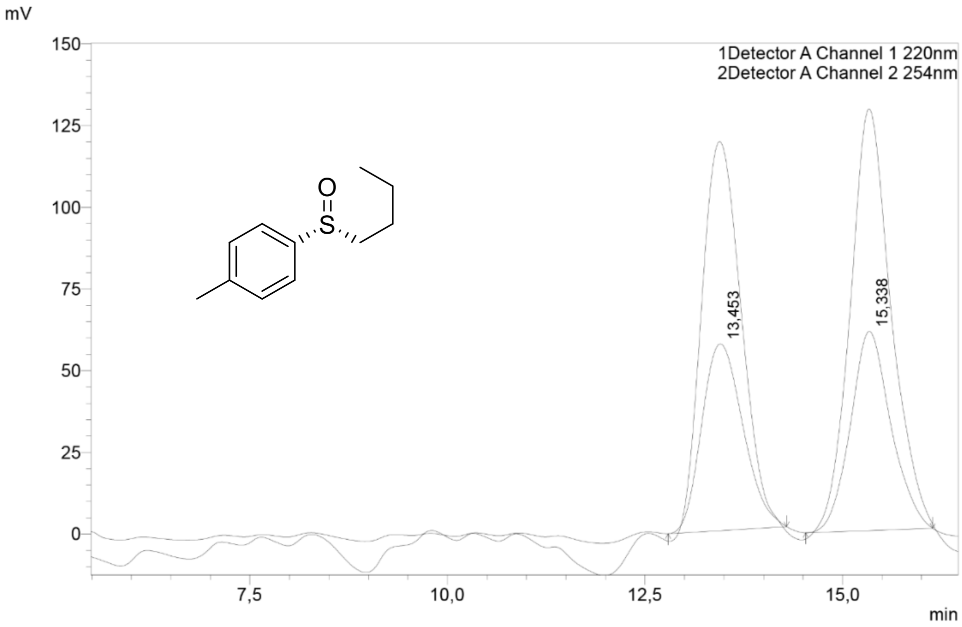

Detector A Channel 2 254nm

Peak#	Ret. Time	Area	Height	Conc.	Unit	Mark	Name
1	13,453	2077613	57146	48,836		M	
2	15,338	2176630	60941	51,164		M	
Total		4254242	118087				

Chromatogram of enantioenriched molecule **SM-B2**:

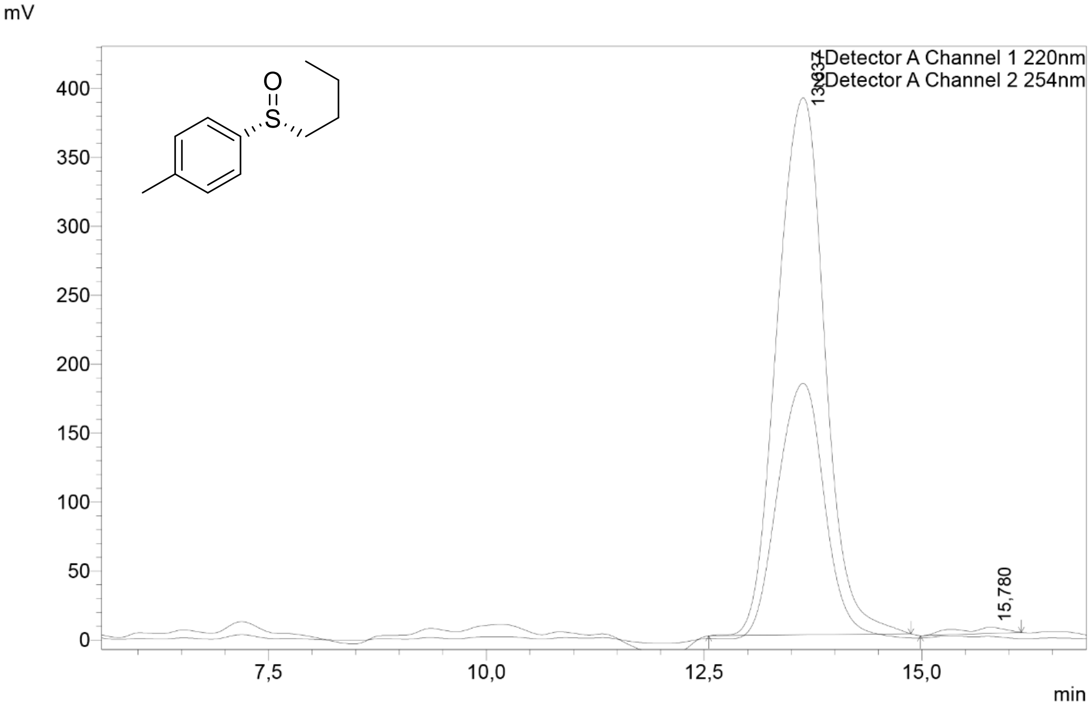

Detector A Channel 1 220nm

Peak#	Ret. Time	Area	Height	Conc.	Unit	Mark	Name
1	13,637	14910855	389675	98,845		M	
2	15,780	174168	4473	1,155		M	
Total		15085023	394149				

Chromatogram of racemic molecule **SM-C1**:

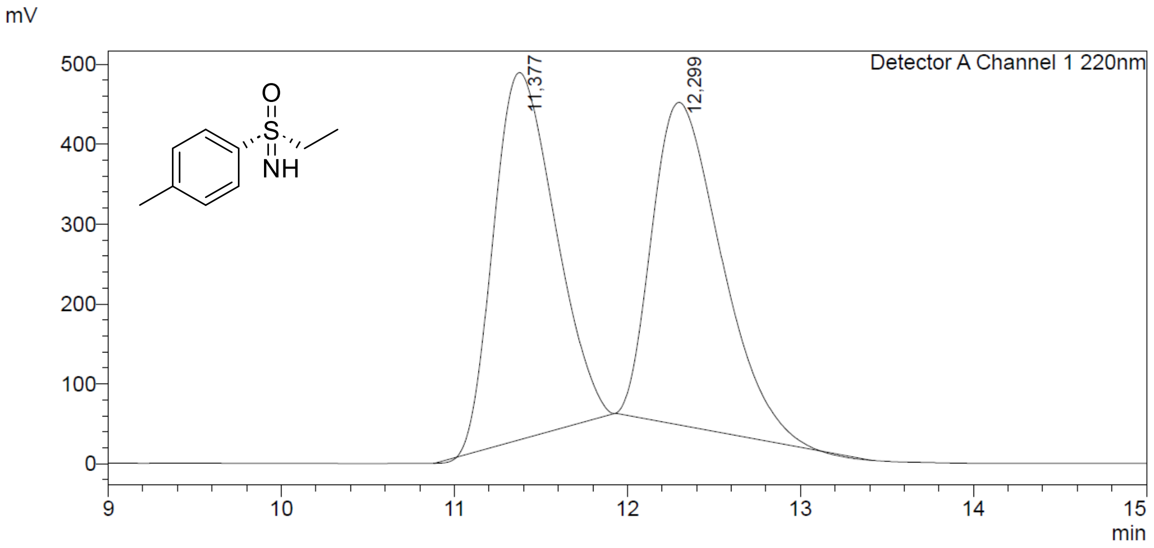

Peak#	Ret. Time	Area	Height	Conc.	Unit	Mark	Name
1	11,377	11358697	459620	50,151		M	
2	12,299	11290445	403581	49,849		M	
Total		22649142	863201				

Chromatogram of enantioenriched molecule **SM-C1**:

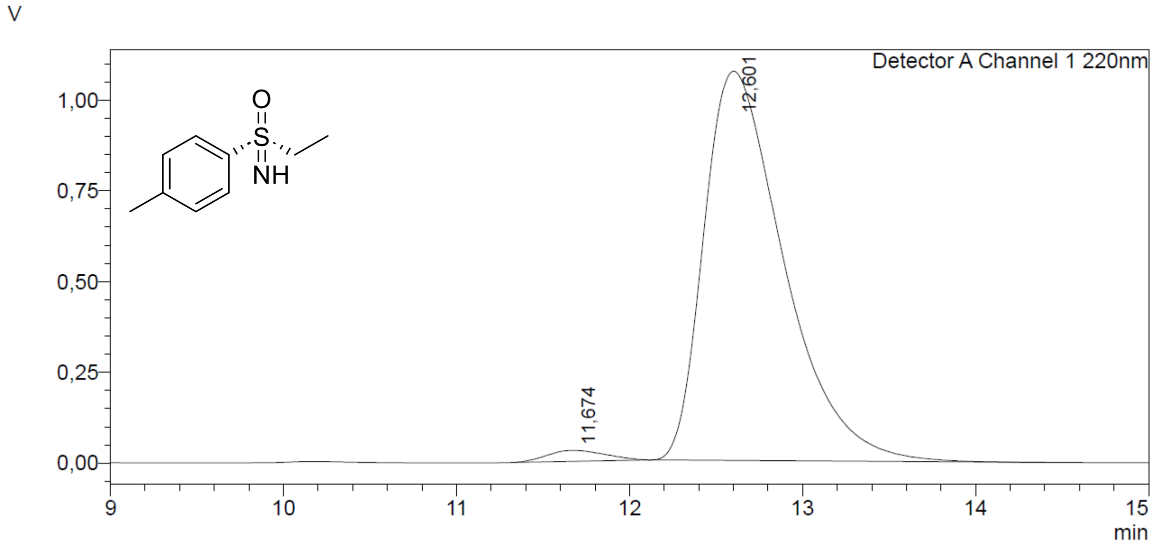

Peak#	Ret. Time	Area	Height	Conc.	Unit	Mark	Name
1	11,674	718402	30695	2,038		M	
2	12,601	34537023	1072733	97,962		M	
Total		35255426	1103428				

Chromatogram of racemic molecule **SM-C2**:

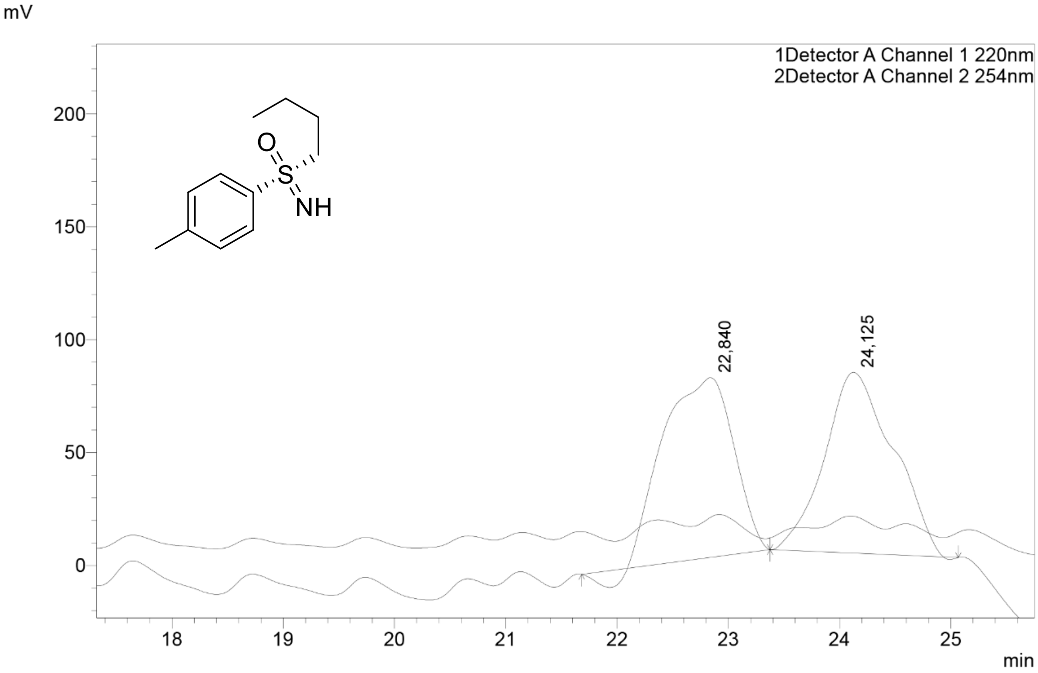

Detector A Channel 1 220nm

Peak#	Ret. Time	Area	Height	Conc.	Unit	Mark	Name
1	22,840	3484098	79625	50,233		M	
2	24,125	3451722	80091	49,767		M	
Total		6935820	159716				

Chromatogram of enantioenriched molecule **SM-C2**:

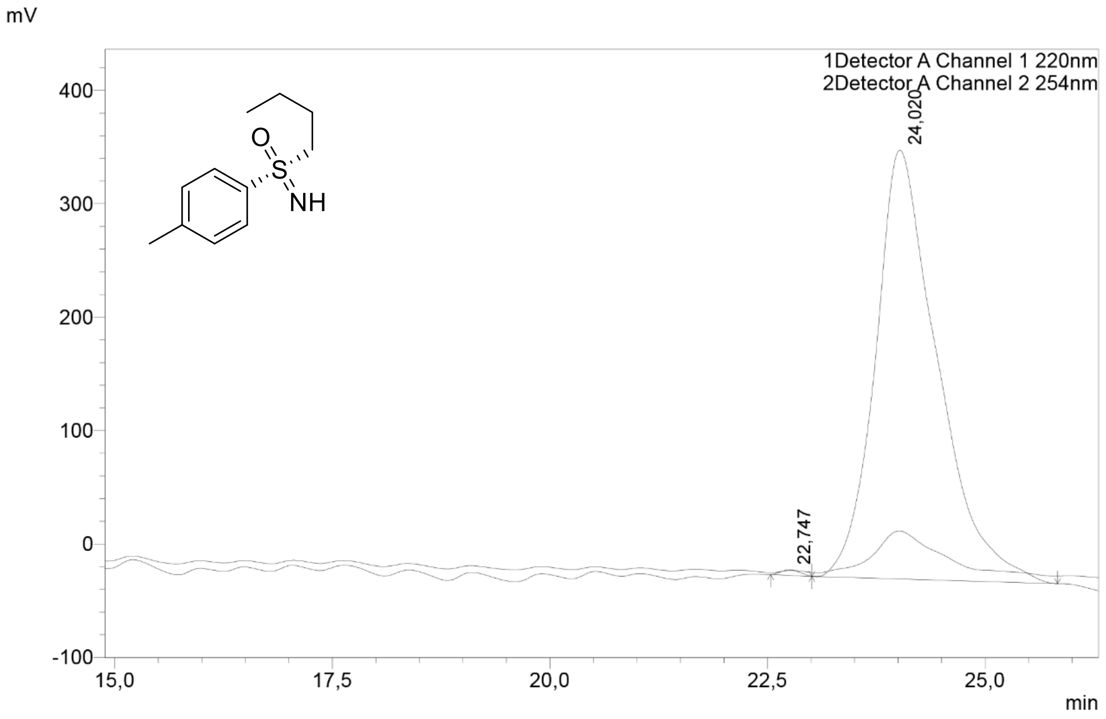

Detector A Channel 1 220nm

Peak#	Ret. Time	Area	Height	Conc.	Unit	Mark	Name
1	22,747	77700	4955	0,412		M	
2	24,020	18762039	378043	99,588		M	
Total		18839738	382998				

Chromatogram of racemic molecule **1a**:

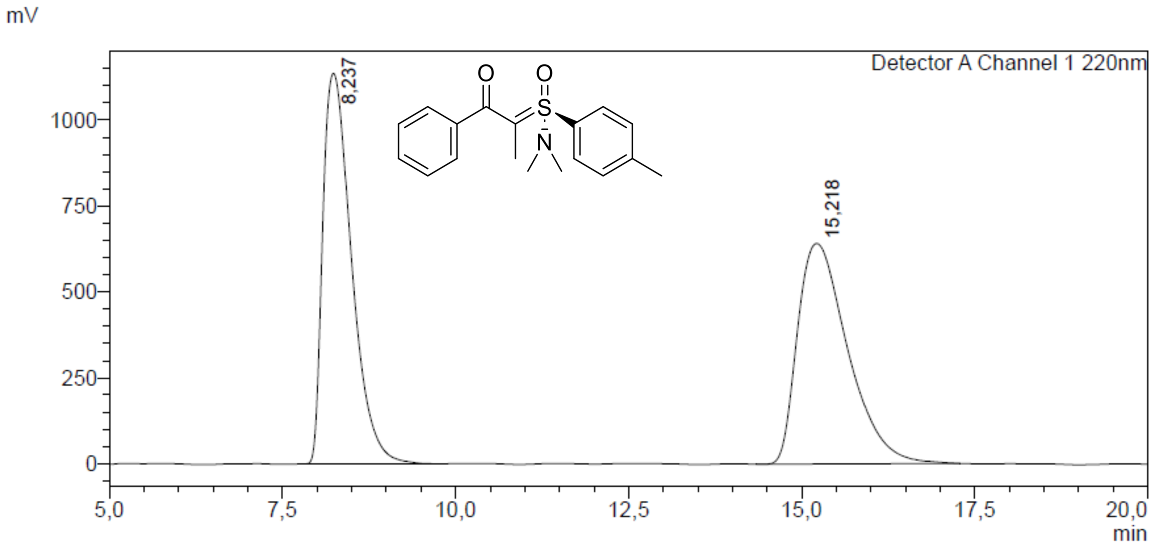

Peak#	Ret. Time	Area	Height	Conc.	Unit	Mark	Name
1	8,237	32874417	1137188	50,325		M	
2	15,218	32449895	641779	49,675		M	
Total		65324312	1778967				

Chromatogram of enantioenriched molecule **1a**:

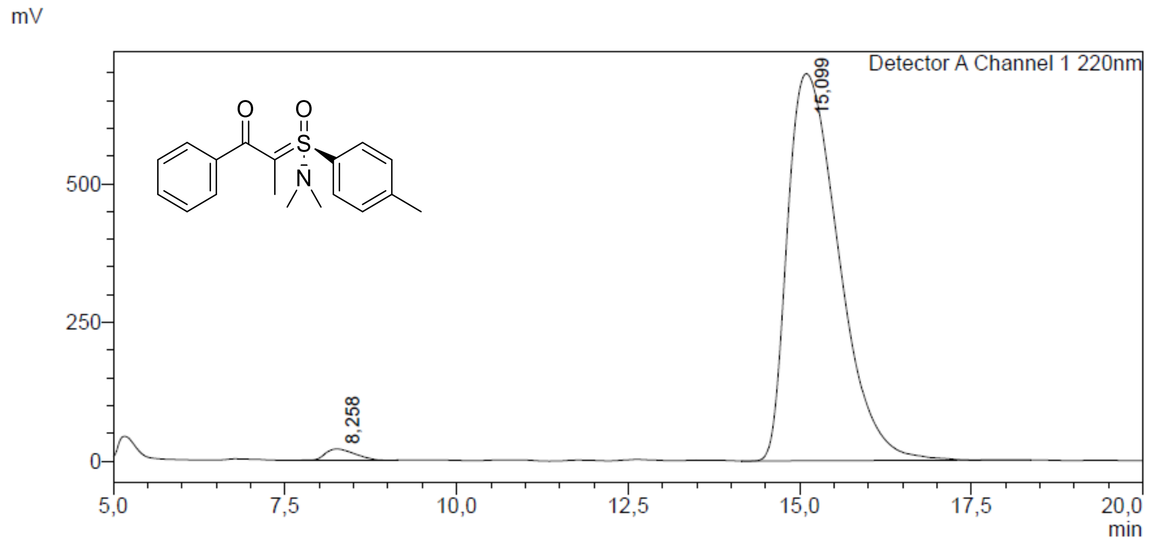

Peak#	Ret. Time	Area	Height	Conc.	Unit	Mark	Name
1	8,258	604125	20258	1,636		M	
2	15,099	36314165	697913	98,364		M	
Total		36918291	718171				

Chromatogram of racemic molecule **1b**:

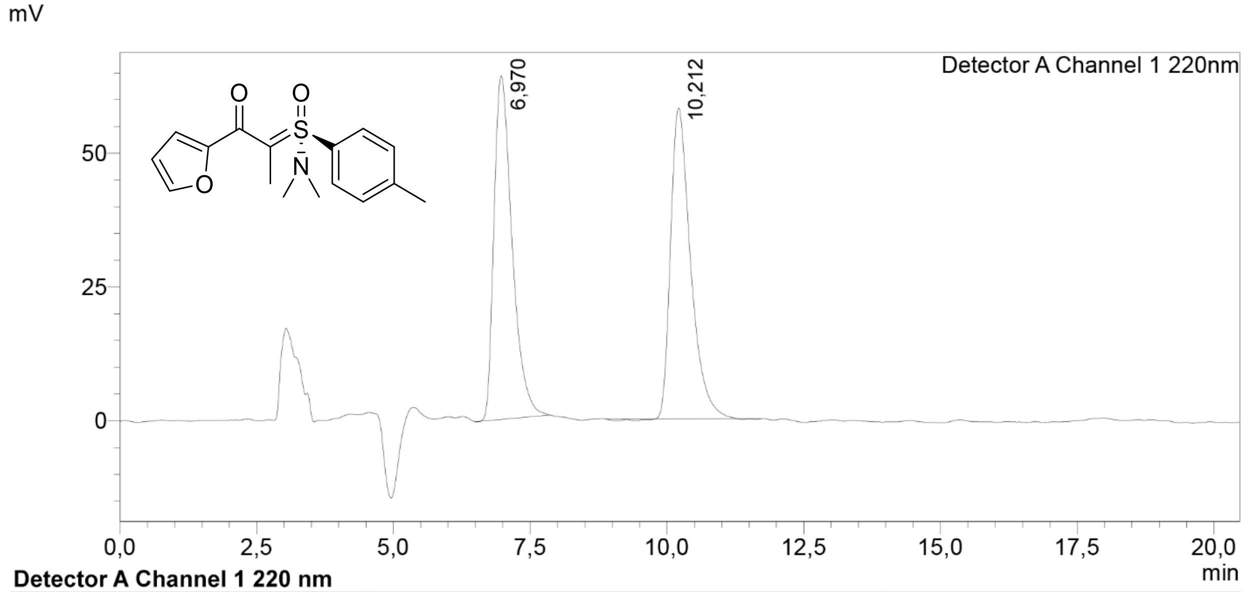

Detector A Channel 1 220 nm

Peak#	Ret. Time	Area	Height	Conc.	Unit	Mark	Name
1	6,970	1483037	64172	50,175		M	
2	10,212	1472696	58093	49,825		M	
Total		2955734	122265				

Chromatogram of enantioenriched molecule **1b**:

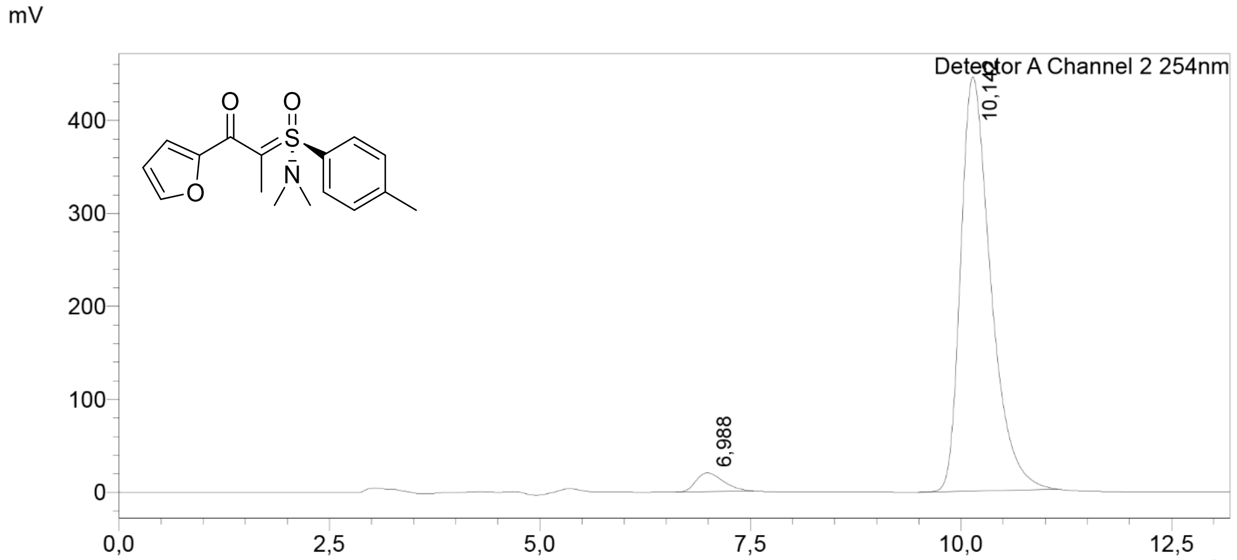

Detector A Channel 2 254nm

Peak#	Ret. Time	Area	Height	Conc.	Unit	Mark	Name
1	6,988	452922	20093	3,930		M	
2	10,142	11071625	445407	96,070		M	
Total		11524547	465499				

Chromatogram of racemic molecule **1c**:

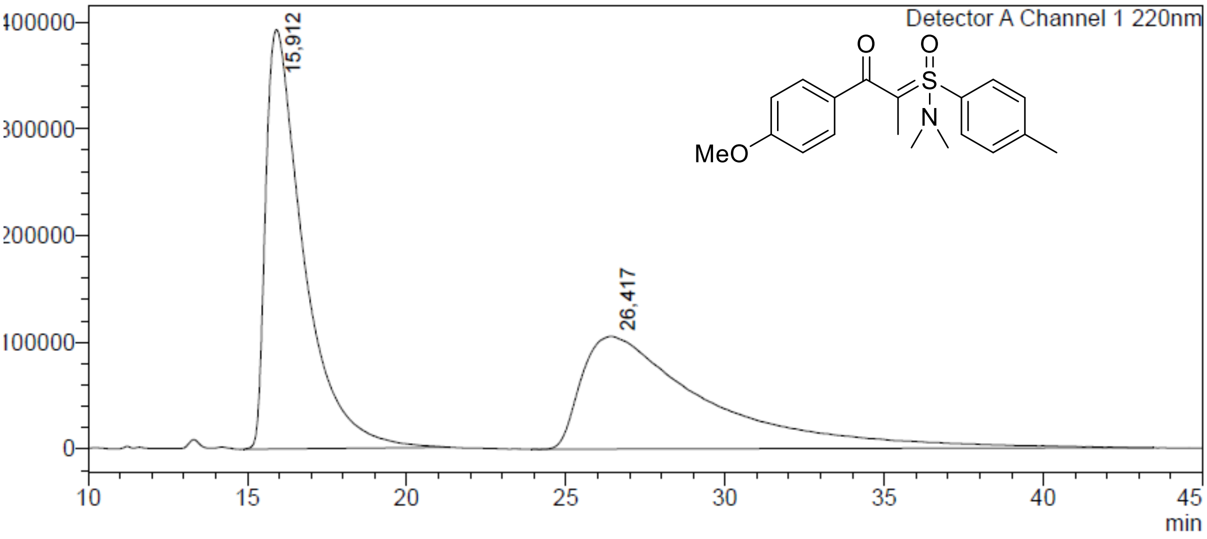

Peak#	Ret. Time	Area	Height	Conc.	Unit	Mark	Name
1	15,912	32477115	392882	52,821		M	
2	26,417	29008133	105788	47,179		M	
Total		61485248	498670				

Chromatogram of enantioenriched molecule **1c**:

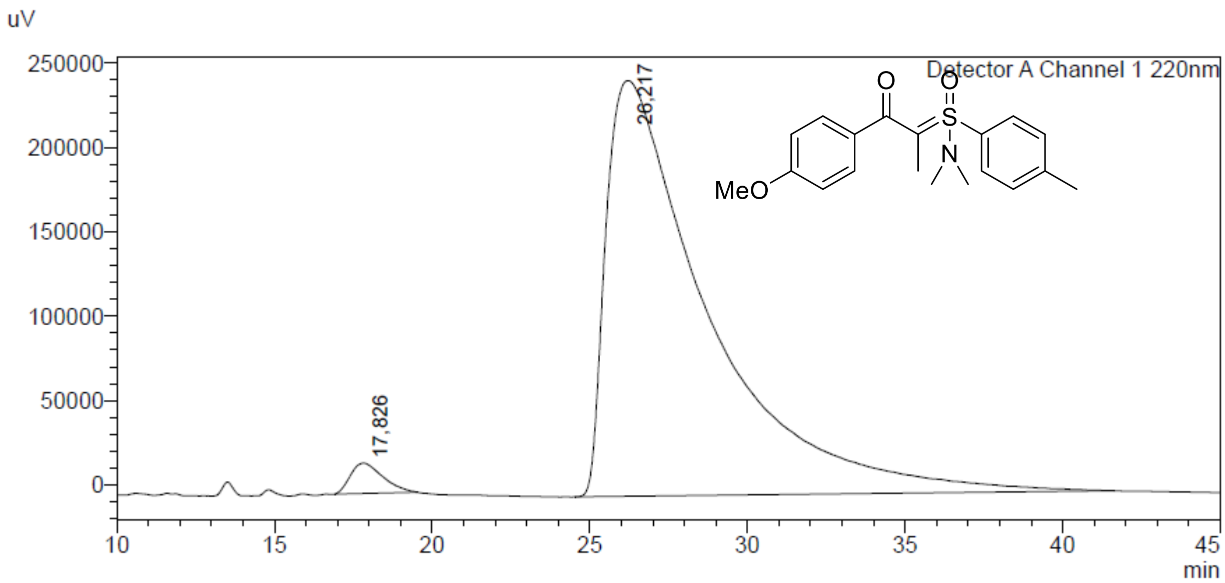

Peak#	Ret. Time	Area	Height	Conc.	Unit	Mark	Name
1	17,826	1283075	17967	2,257		M	
2	26,217	55574760	246358	97,743		M	
Total		56857835	264325				

Chromatogram of racemic molecule **1d**:

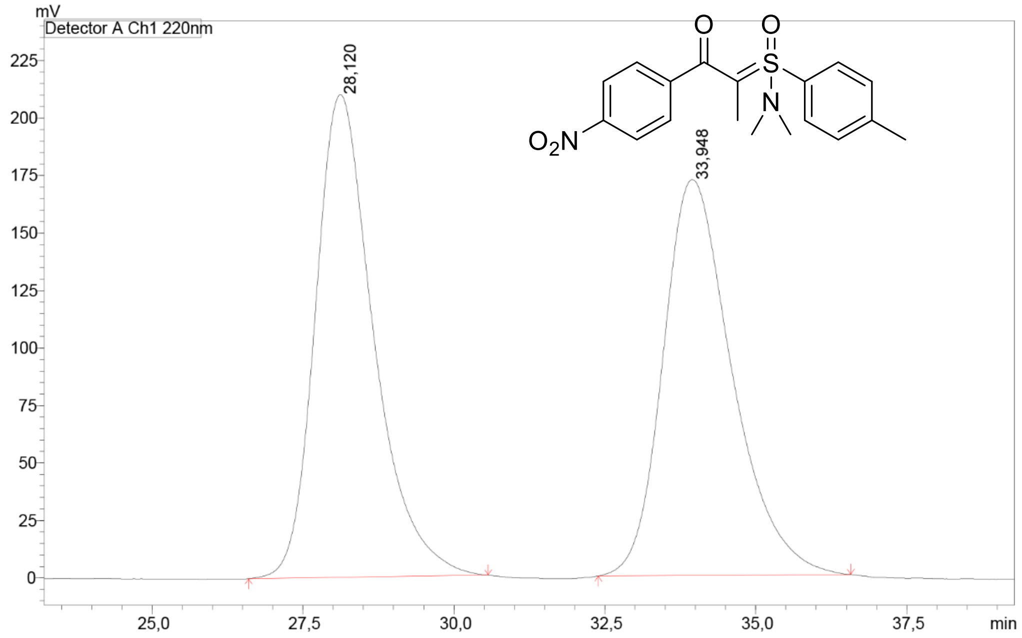

Peak Table

Detector A Channel 1 220nm

Peak#	Ret. Time	Area	Height	Area%	Height%
1	28,120	14112016	209933	50,704	54,941
2	33,948	13720389	172171	49,296	45,059
Total		27832405	382104	100,000	100,000

Chromatogram of enantioenriched molecule **1d**:

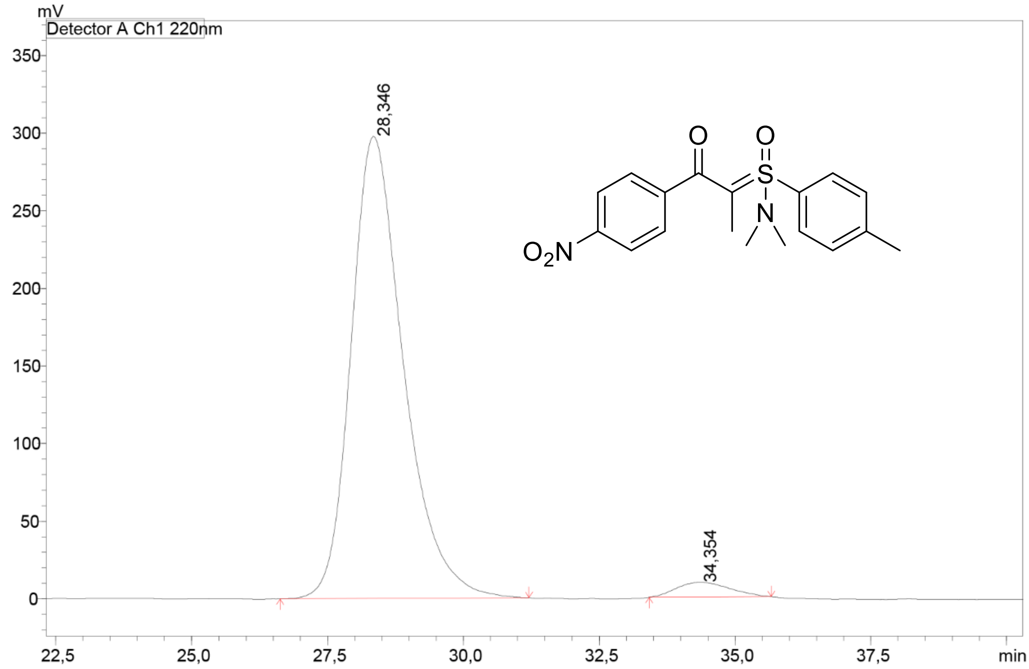

Peak Table

Detector A Channel 1 220nm

Peak#	Ret. Time	Area	Height	Area%	Height%
1	28,346	20042177	297680	96,850	96,880
2	34,354	651813	9587	3,150	3,120
Total		20693990	307267	100,000	100,000

Chromatogram of racemic molecule **1e**:

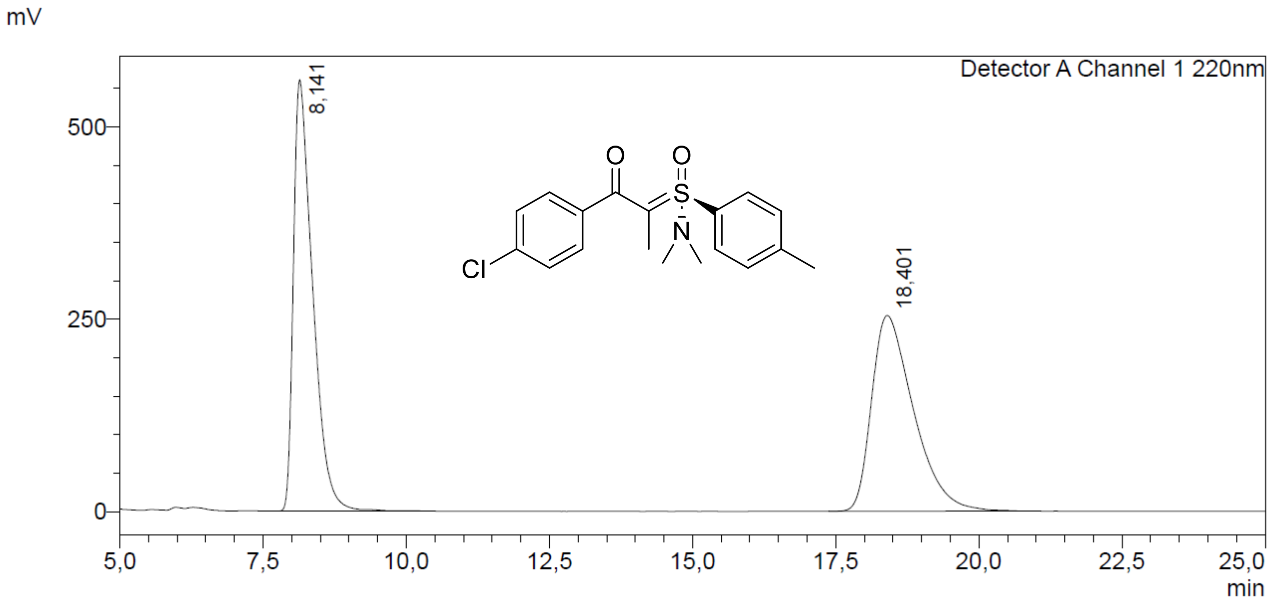

Peak#	Ret. Time	Area	Height	Conc.	Unit	Mark	Name
1	8,141	13053686	560094	50,126		M	
2	18,401	12988159	254325	49,874		M	
Total		26041844	814420				

Chromatogram of enantioenriched molecule **1e**:

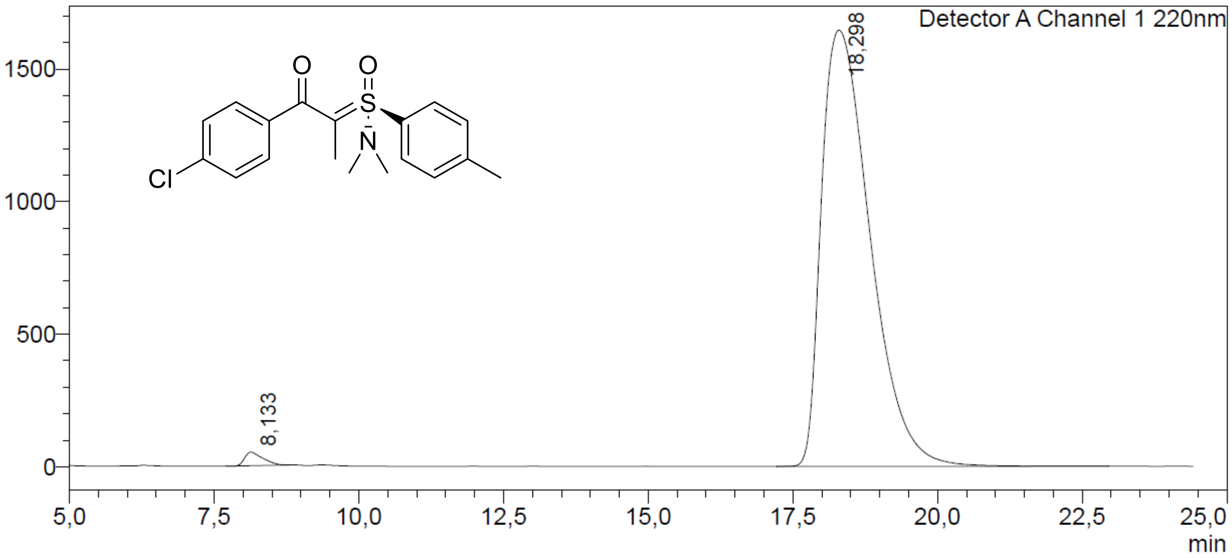

Peak#	Ret. Time	Area	Height	Conc.	Unit	Mark	Name
1	8,133	1122224	51847	1,152		M	
2	18,298	96252757	1645390	98,848		M	
Total		97374981	1697237				

Chromatogram of racemic molecule **1f**:

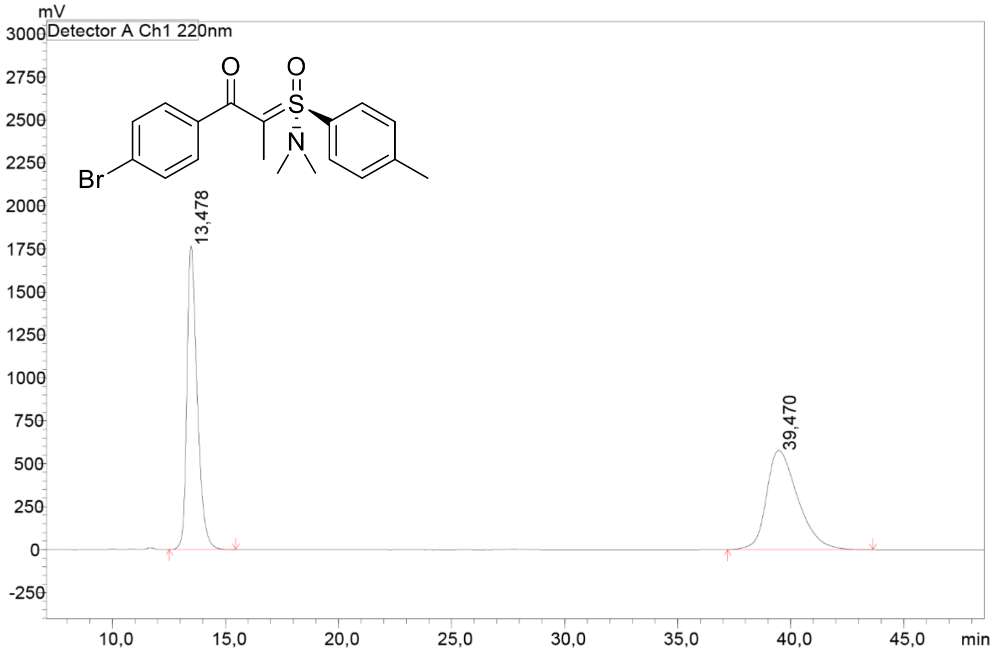

Peak Table

Detector A Channel 1 220nm

Peak#	Ret. Time	Area	Height	Area%	Height%
1	13,478	56098048	1769093	50,133	75,388
2	39,470	55799944	577555	49,867	24,612
Total		111897992	2346647	100,000	100,000

Chromatogram of enantioenriched molecule **1f**:

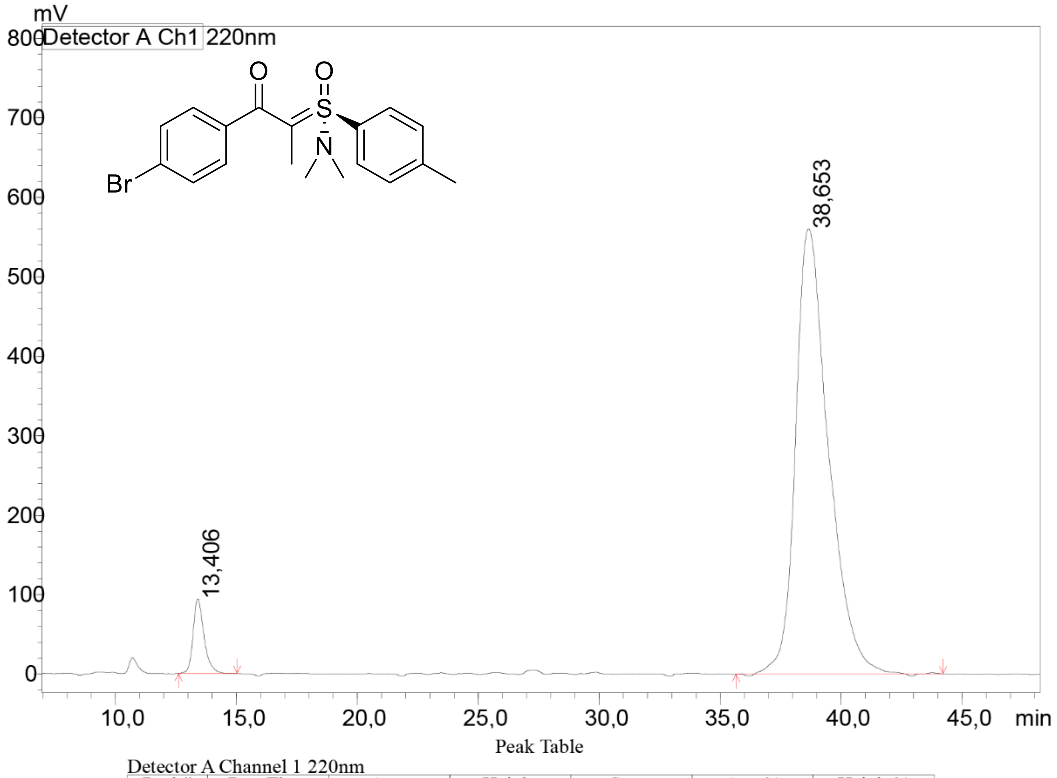

Peak Table

Detector A Channel 1 220nm

Peak#	Ret. Time	Area	Height	Conc.	Area%	Height%
1	13,406	2950217	93519	5,269	5,269	14,300
2	38,653	53043889	560468	94,731	94,731	85,700
Total		55994107	653987		100,000	100,000

Chromatogram of racemic molecule **1g**:

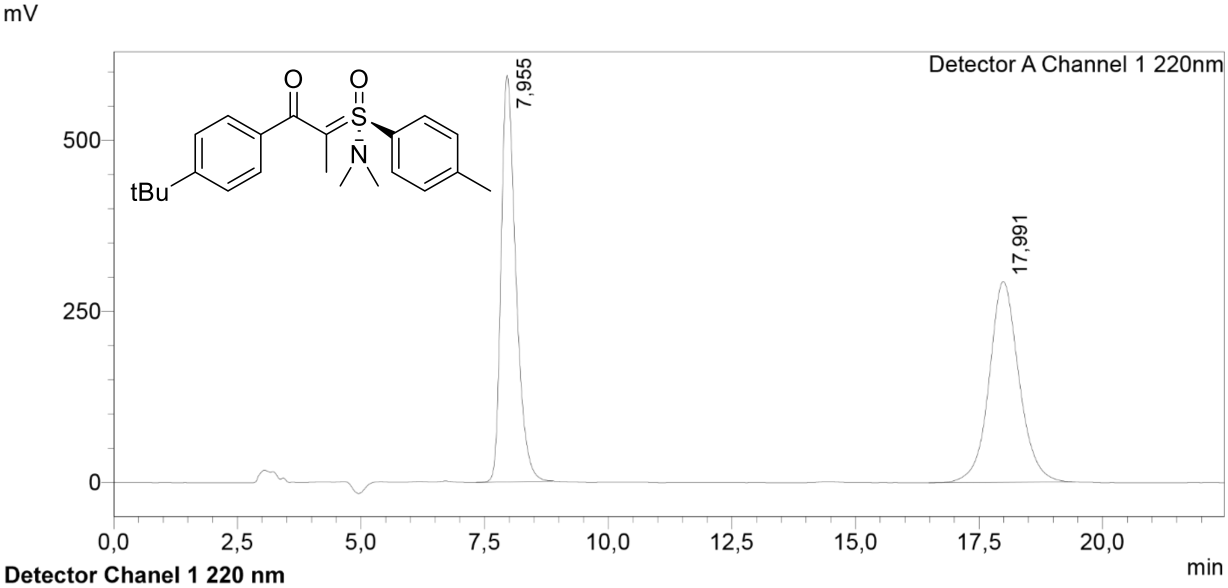

Detector Chanel 1 220 nm

Peak#	Ret. Time	Area	Height	Conc.	Unit	Mark	Name
1	7,955	12317560	594314	49,943		M	
2	17,991	12345746	293500	50,057		M	
Total		24663306	887813				

Chromatogram of enantioenriched molecule **1g**:

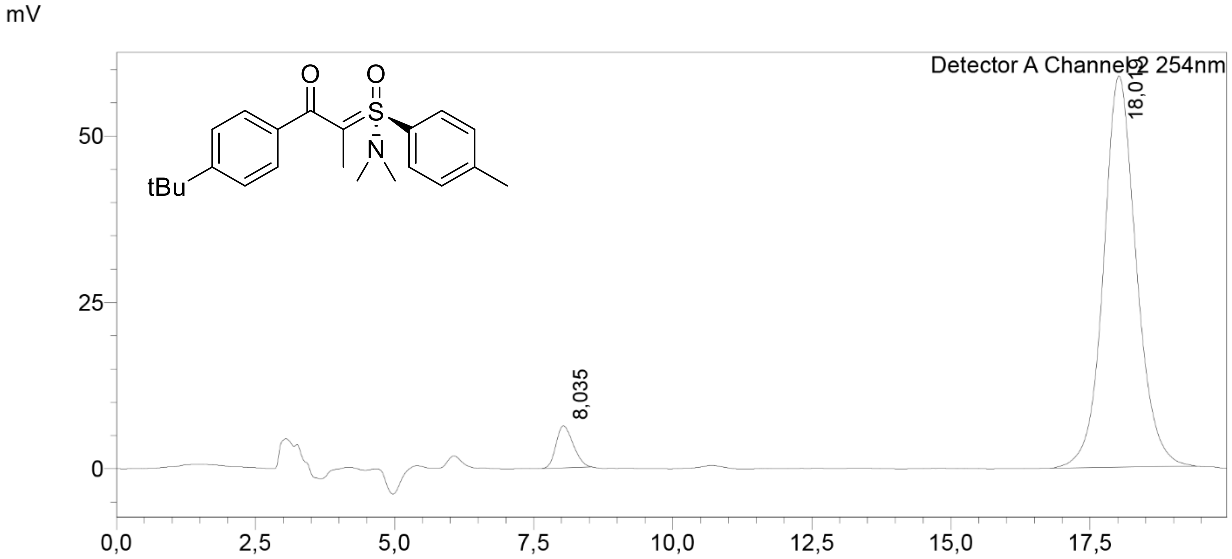

Detector A Channel 2 254nm

Peak#	Ret. Time	Area	Height	Conc.	Unit	Mark	Name
1	8,035	135357	6314	5,299		M	
2	18,019	2418840	58832	94,701		M	
Total		2554197	65146				

Chromatogram of racemic molecule **1h**:

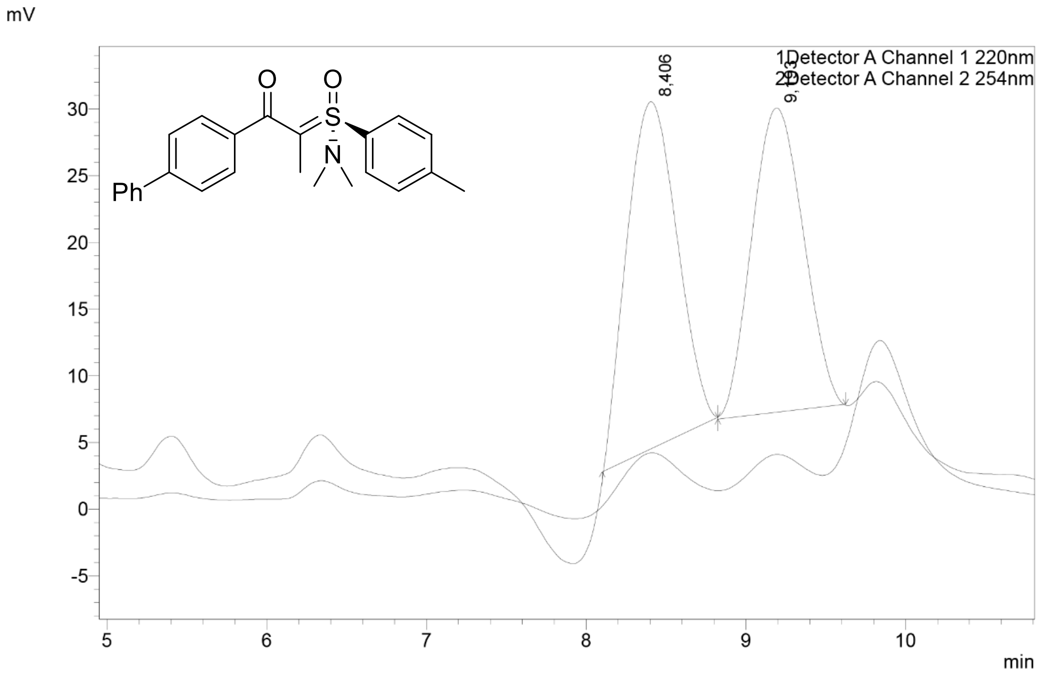

Detector A Channel 1 220nm

Peak#	Ret. Time	Area	Height	Conc.	Unit	Mark	Name
1	8,406	642069	27737	55,616		M	
2	9,193	512391	22884	44,384		M	
Total		1154460	50621				

Chromatogram of enantioenriched molecule **1h**:

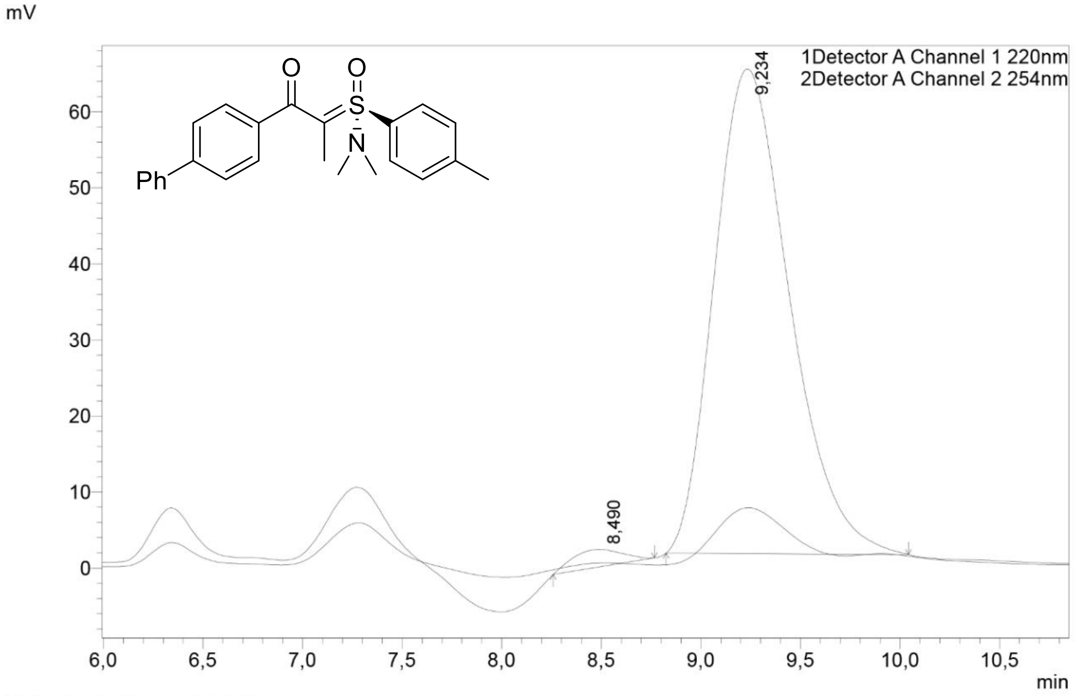

Detector A Channel 1 220nm

Peak#	Ret. Time	Area	Height	Conc.	Unit	Mark	Name
1	8,490	41425	2290	2,408		M	
2	9,234	1679101	63740	97,592		M	
Total		1720526	66031				

Chromatogram of racemic molecule **1i**:

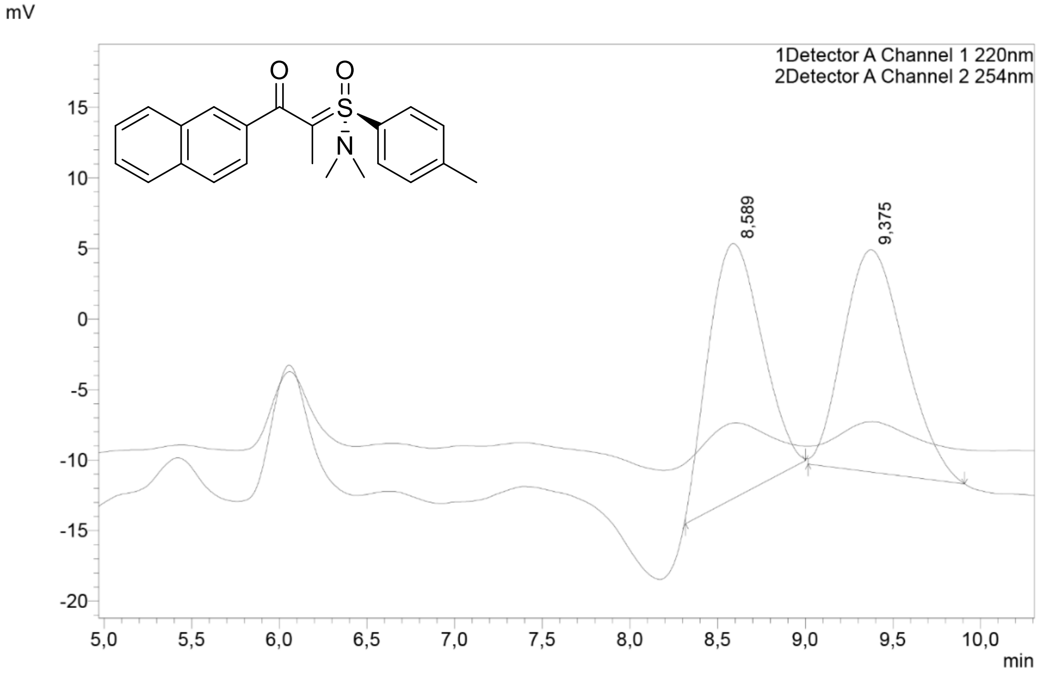

Detector A Channel 1 220nm

Peak#	Ret. Time	Area	Height	Conc.	Unit	Mark	Name
1	8,589	402610	18547	52,006		M	
2	9,375	371548	15409	47,994		M	
Total		774158	33956				

Chromatogram of enantioenriched molecule **1i**:

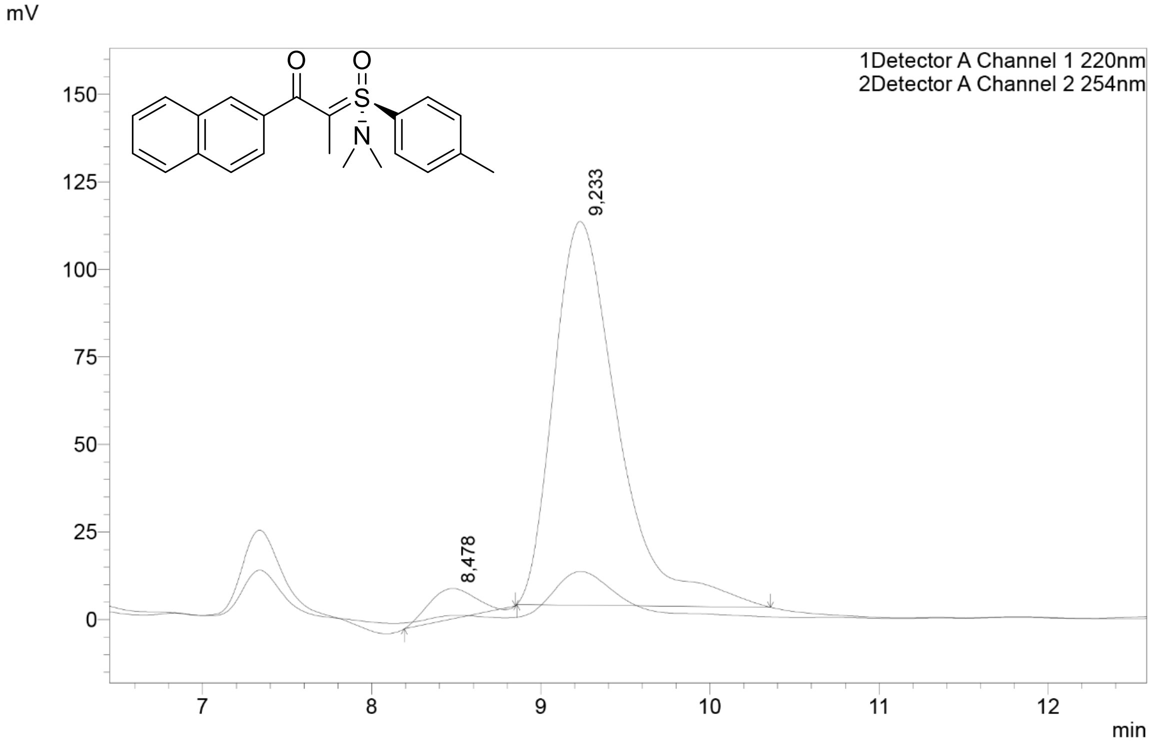

Detector A Channel 1 220nm

Peak#	Ret. Time	Area	Height	Conc.	Unit	Mark	Name
1	8,478	161514	8650	5,274		M	
2	9,233	2900690	109568	94,726		M	
Total		3062204	118218				

Chromatogram of racemic molecule **1j**:

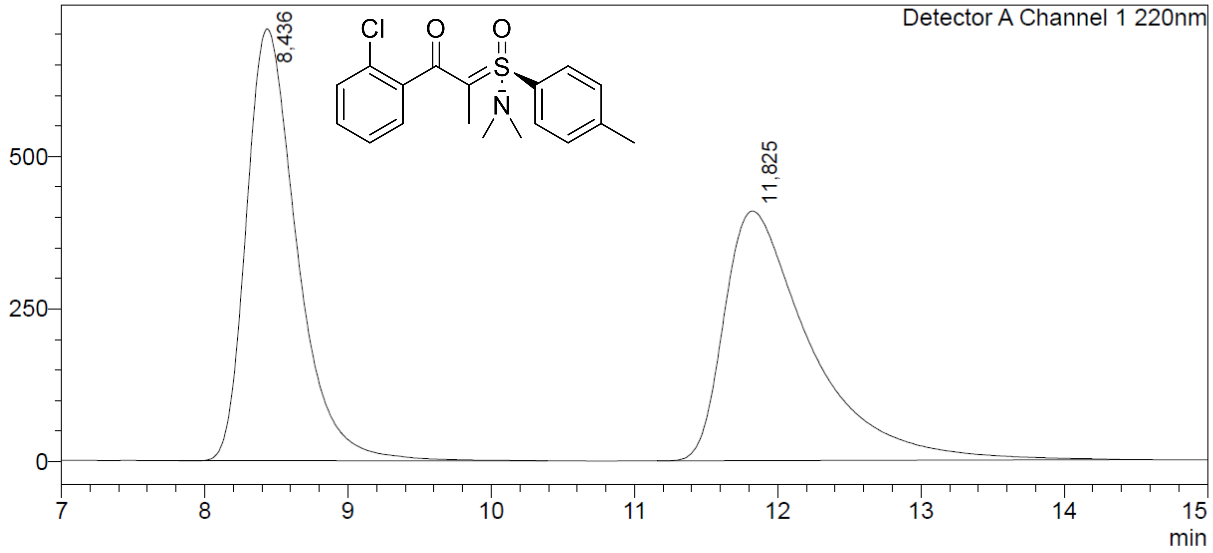

Peak#	Ret. Time	Area	Height	Conc.	Unit	Mark	Name
1	8,436	17386092	707653	50,178		M	
2	11,825	17262475	409052	49,822		M	
Total		34648567	1116705				

Chromatogram of enantioenriched molecule **1j**:

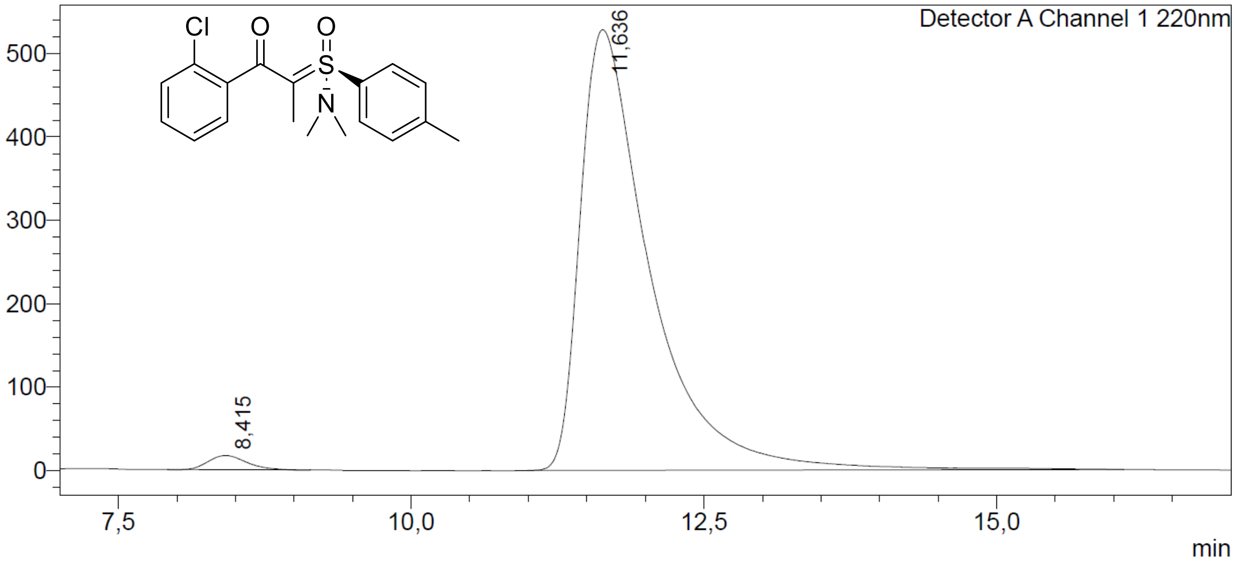

Peak#	Ret. Time	Area	Height	Conc.	Unit	Mark	Name
1	8,415	388670	17116	1,736		M	
2	11,636	21997215	528500	98,264		M	
Total		22385884	545617				

Chromatogram of racemic molecule **1k**:

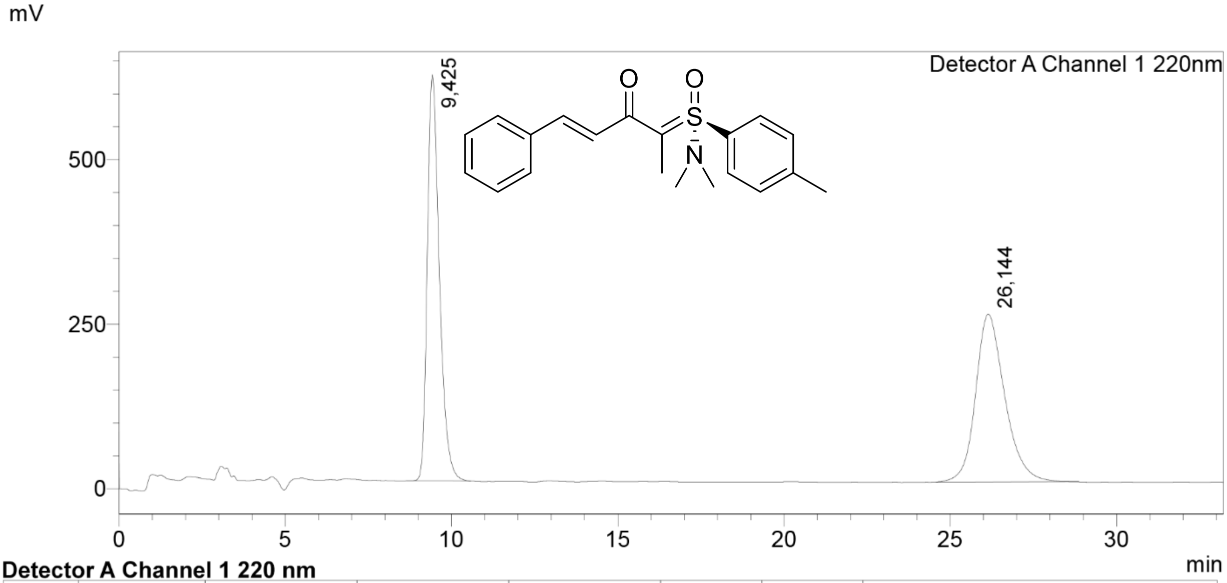

Detector A Channel 1 220 nm

Peak#	Ret. Time	Area	Height	Conc.	Unit	Mark	Name
1	9,425	15564106	616933	49,999		M	
2	26,144	15564454	254901	50,001		M	
Total		31128560	871834				

Chromatogram of enantioenriched molecule **1k**:

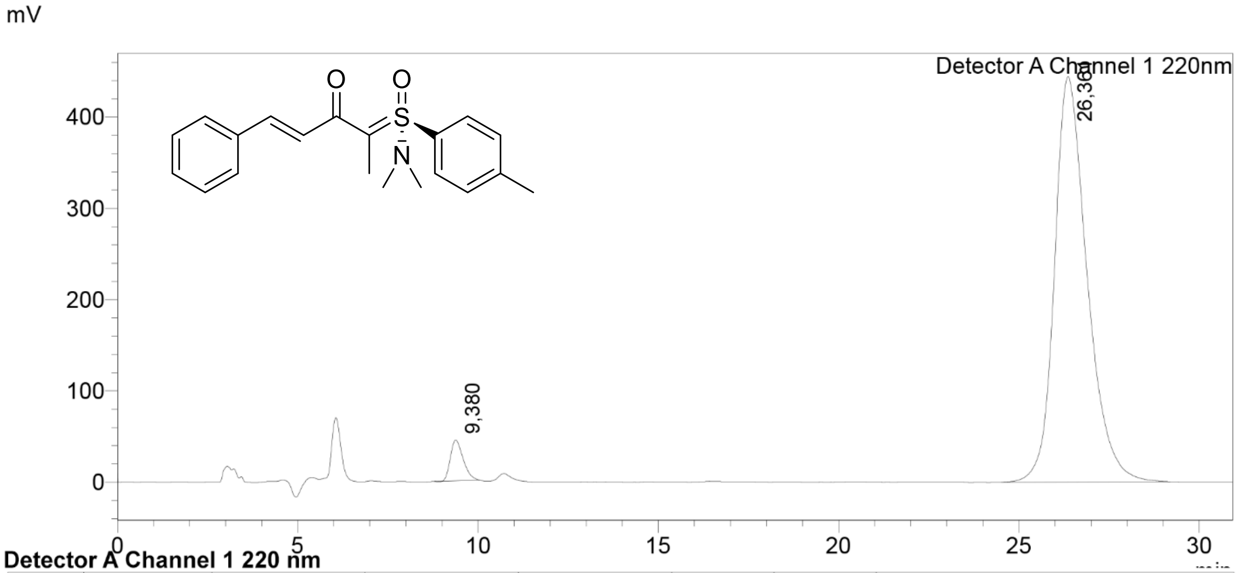

Detector A Channel 1 220 nm

Peak#	Ret. Time	Area	Height	Conc.	Unit	Mark	Name
1	9,380	1106375	44998	3,806		M	
2	26,361	27960027	444044	96,194		M	
Total		29066402	489042				

Chromatogram of racemic molecule **1l**:

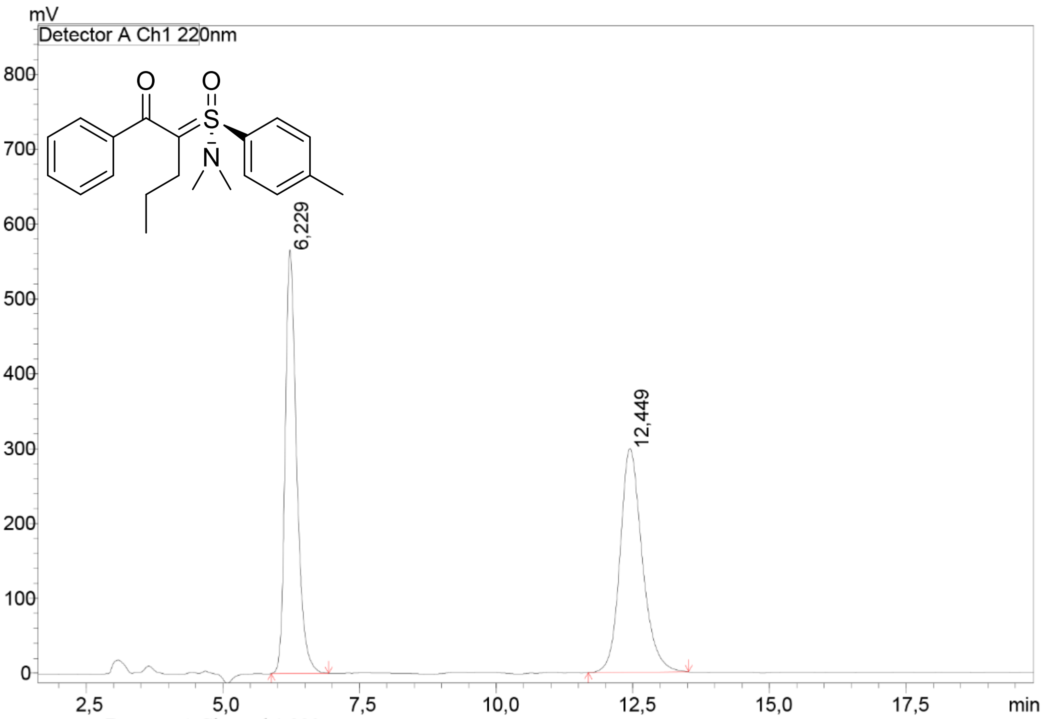

Detector A Channel 1 220nm

Peak#	Ret. Time	Area	Height	Conc.	Area%	Height%
1	6,229	8292693	565672	49,488	49,488	65,422
2	12,449	8464384	298978	50,512	50,512	34,578
Total		16757077	864650		100,000	100,000

Chromatogram of enantioenriched molecule **1l**:

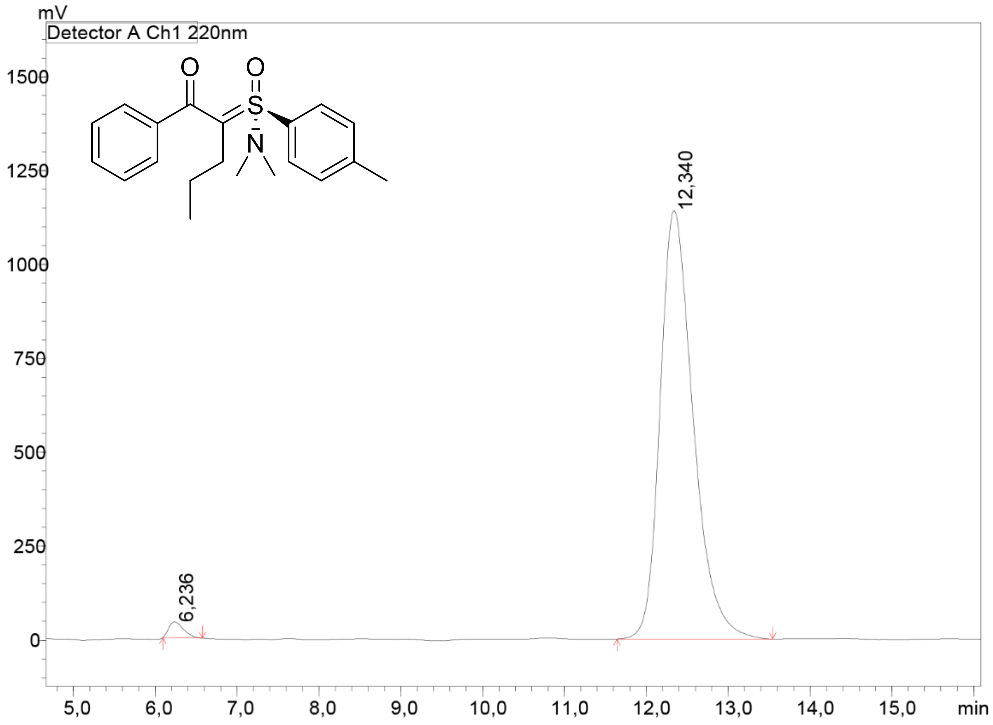

Detector A Channel 1 220nm

Peak#	Ret. Time	Area	Height	Area%	Height%
1	6,236	526768	42299	1,615	3,576
2	12,340	32086171	1140603	98,385	96,424
Total		32612940	1182902	100,000	100,000

Chromatogram of racemic molecule **1m**:

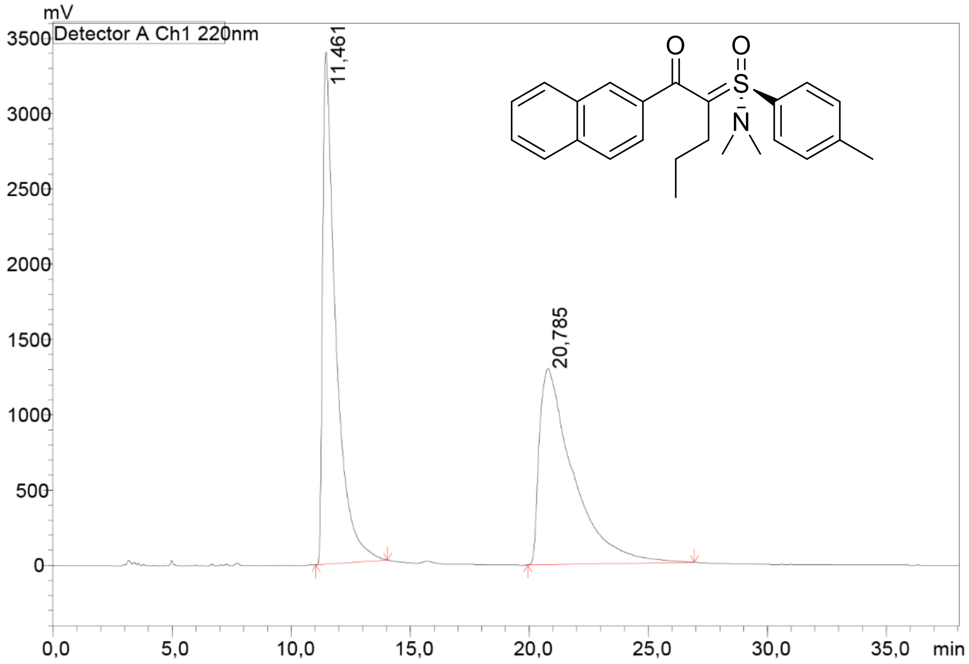

Detector A Channel 1 220nm

Peak#	Ret. Time	Area	Height	Area%	Height%
1	11,461	130922334	3400603	50,465	72,319
2	20,785	128510432	1301652	49,535	27,681
Total		259432766	4702255	100,000	100,000

Chromatogram of enantioenriched molecule **1m**:

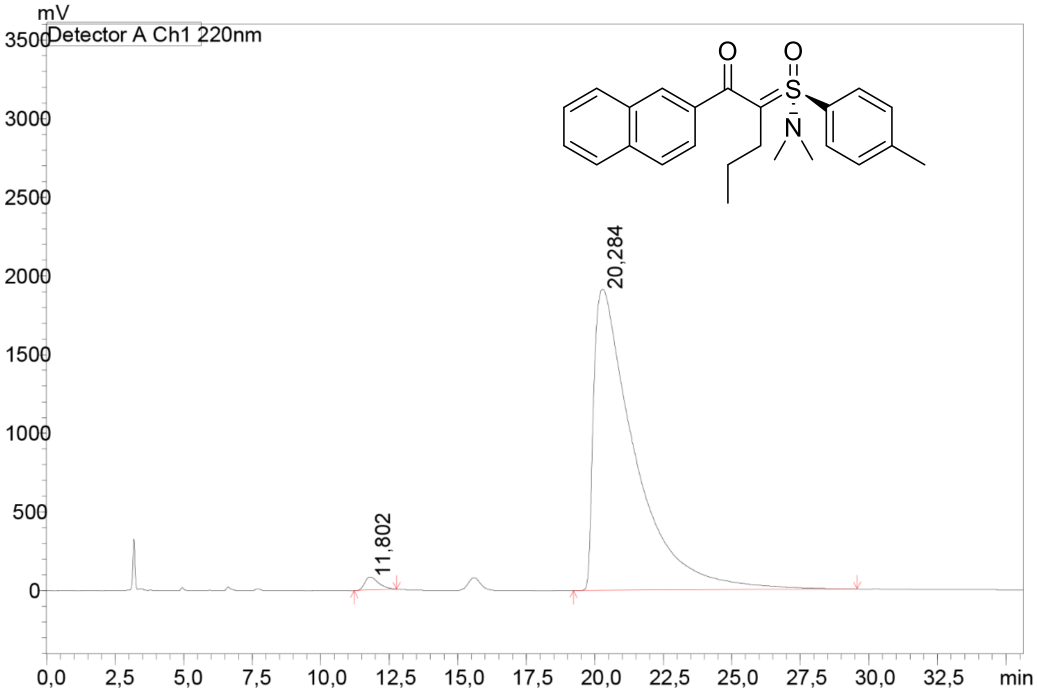

Detector A Channel 1 220nm

Peak#	Ret. Time	Area	Height	Area%	Height%
1	11,802	2915494	83299	1,437	4,169
2	20,284	199933685	1914985	98,563	95,831
Total		202849178	1998284	100,000	100,000

Chromatogram of racemic molecule **1n**:

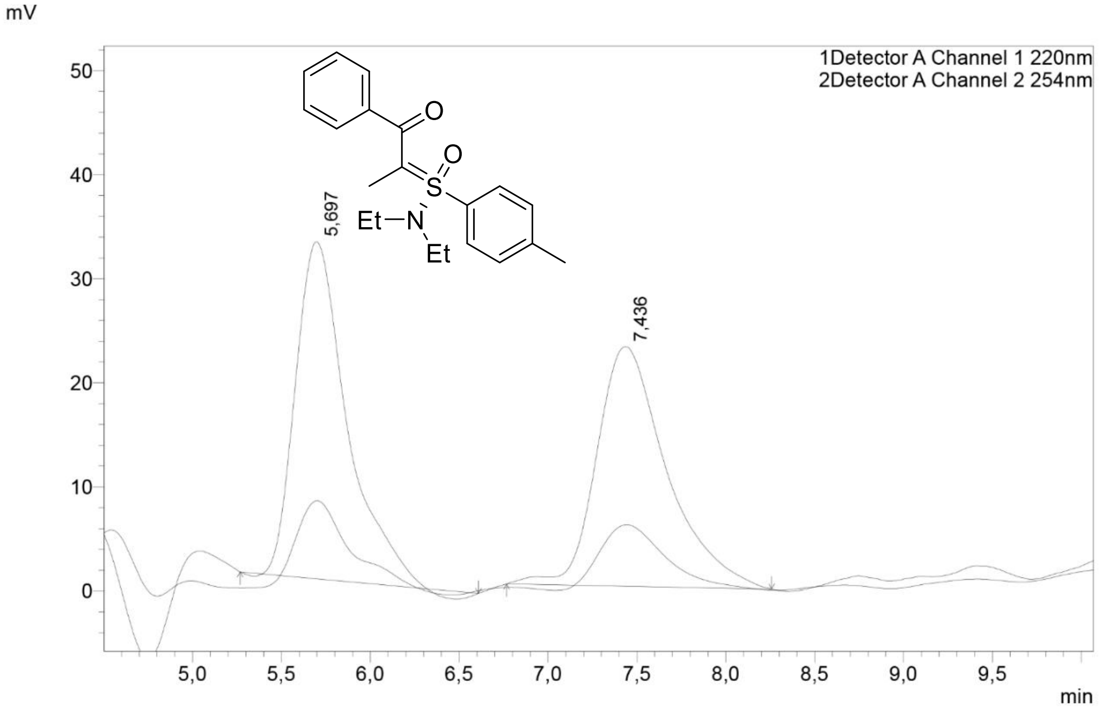

Detector A Channel 1 220nm

Peak#	Ret. Time	Area	Height	Conc.	Unit	Mark	Name
1	5,697	648065	32382	50,632		M	
2	7,436	631898	23028	49,368		M	
Total		1279963	55410				

Chromatogram of enantioenriched molecule **1n**:

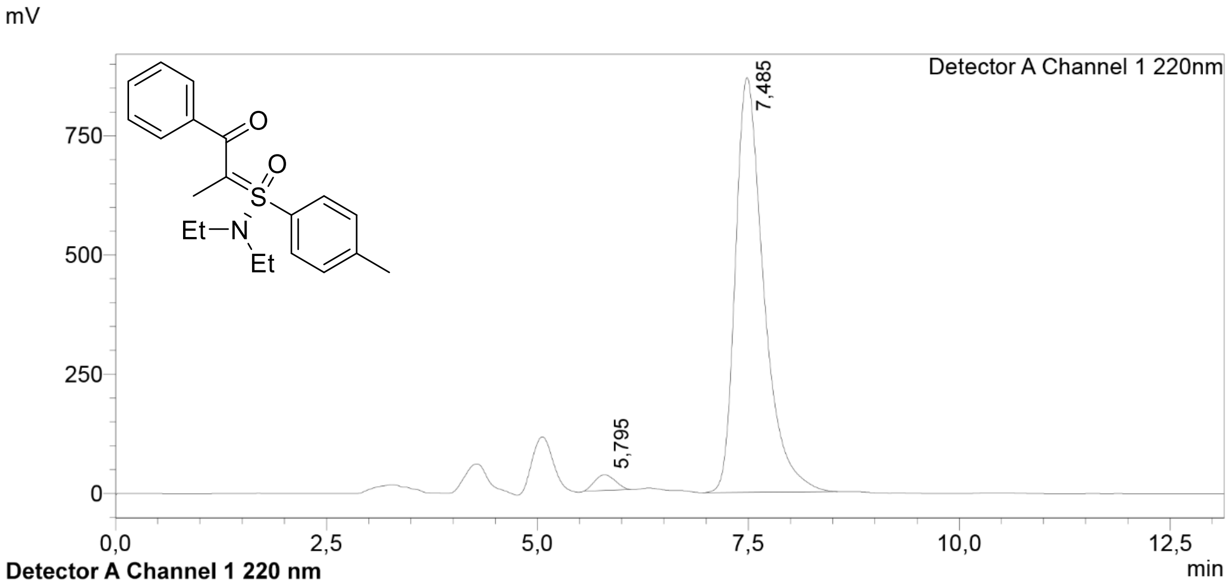

Detector A Channel 1 220 nm

Peak#	Ret. Time	Area	Height	Conc.	Unit	Mark	Name
1	5,795	561166	32832	2,682		M	
2	7,485	20363382	869775	97,318		M	
Total		20924548	902606				

Chromatogram of racemic molecule **3a**:

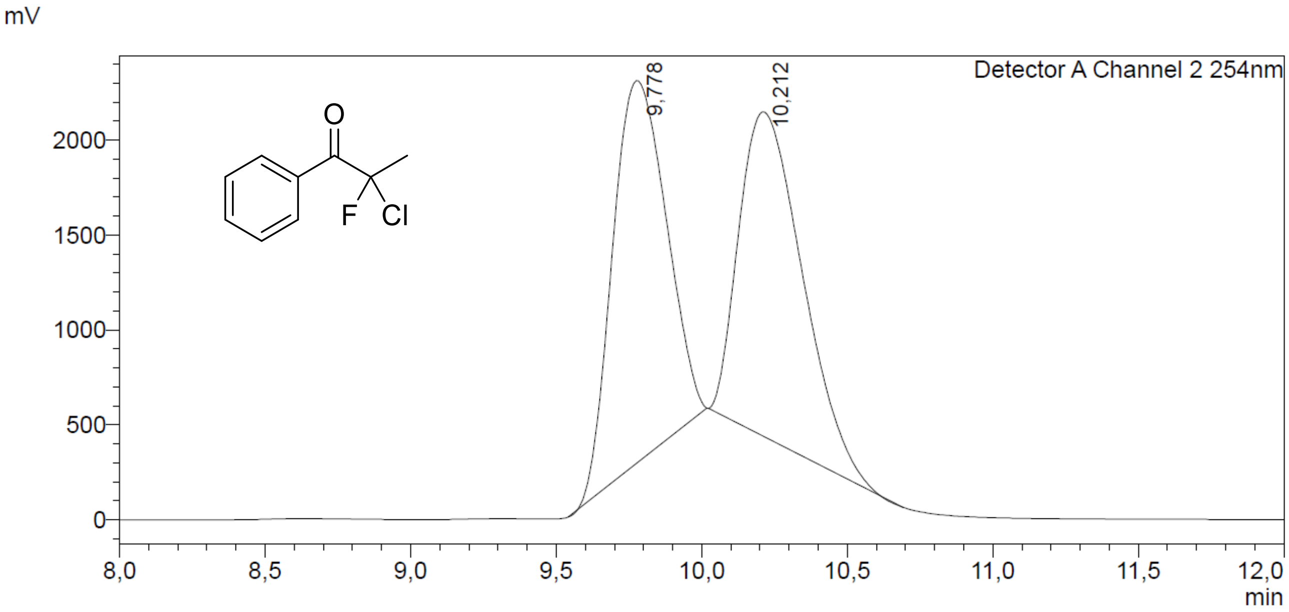

Detector A Channel 2 254nm

Peak#	Ret. Time	Area	Height	Conc.	Unit	Mark	Name
1	9,778	26033692	2012727	50,070		M	
2	10,212	25960901	1707437	49,930		M	
Total		51994592	3720164				

Chromatogram of enantioenriched molecule **3a** (via chiral ylide **1a**):

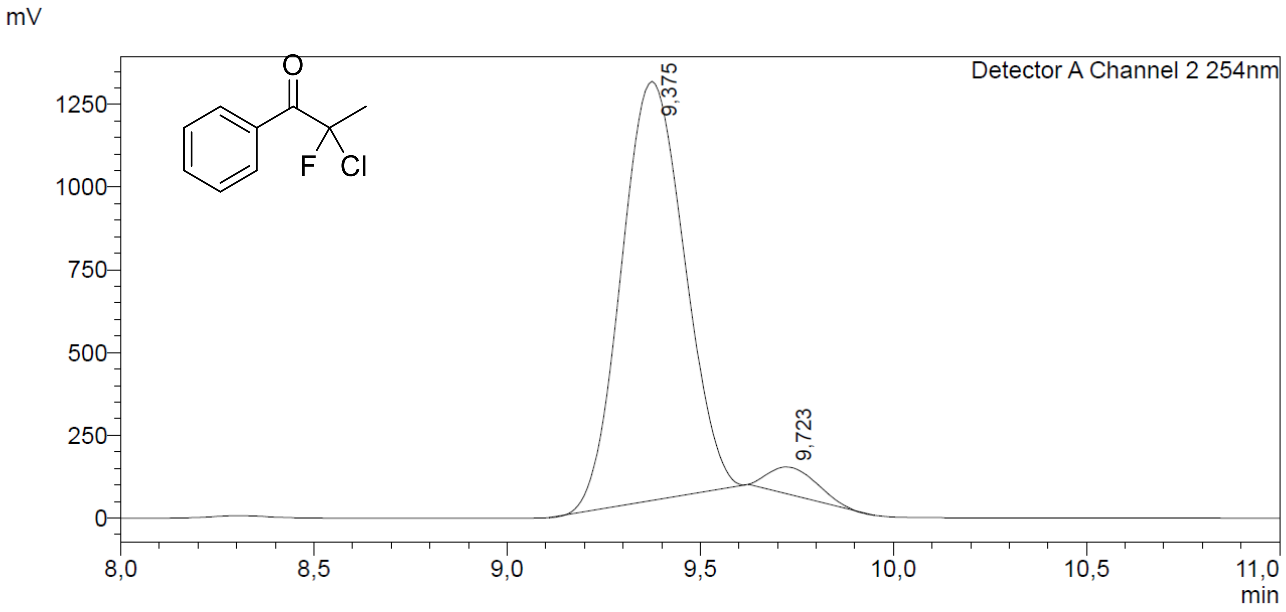

Detector A Channel 2 254nm

Peak#	Ret. Time	Area	Height	Conc.	Unit	Mark	Name
1	9,375	14383471	1266496	95,314		M	
2	9,723	707106	81029	4,686		M	
Total		15090577	1347525				

Chromatogram of enantioenriched molecule **3a** (via achiral ylide **2a**):

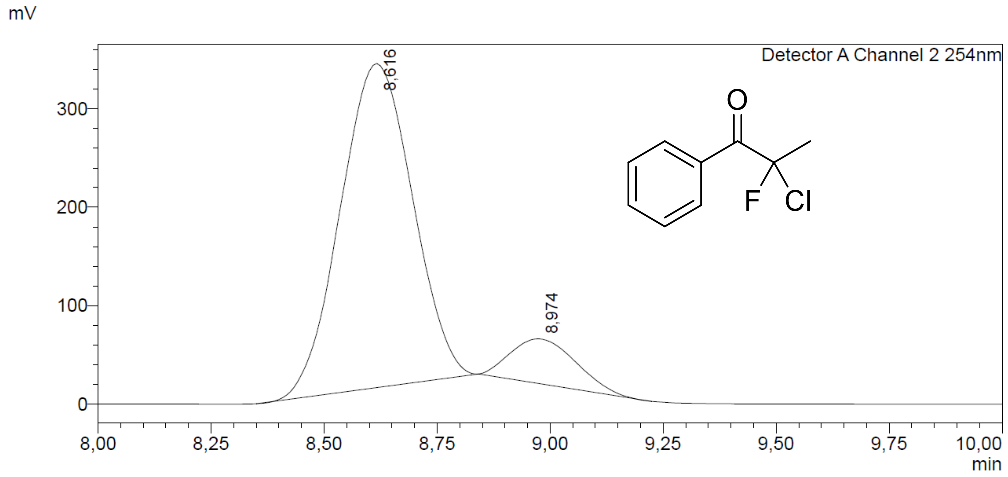

Detector A Channel 2 254nm

Peak#	Ret. Time	Area	Height	Conc.	Unit	Mark	Name
1	8,616	3666239	329396	88,979		M	
2	8,974	454118	45349	11,021		M	
Total		4120357	374745				

Chromatogram of enantioenriched molecule **3a** (via chiral ylide **1n**):

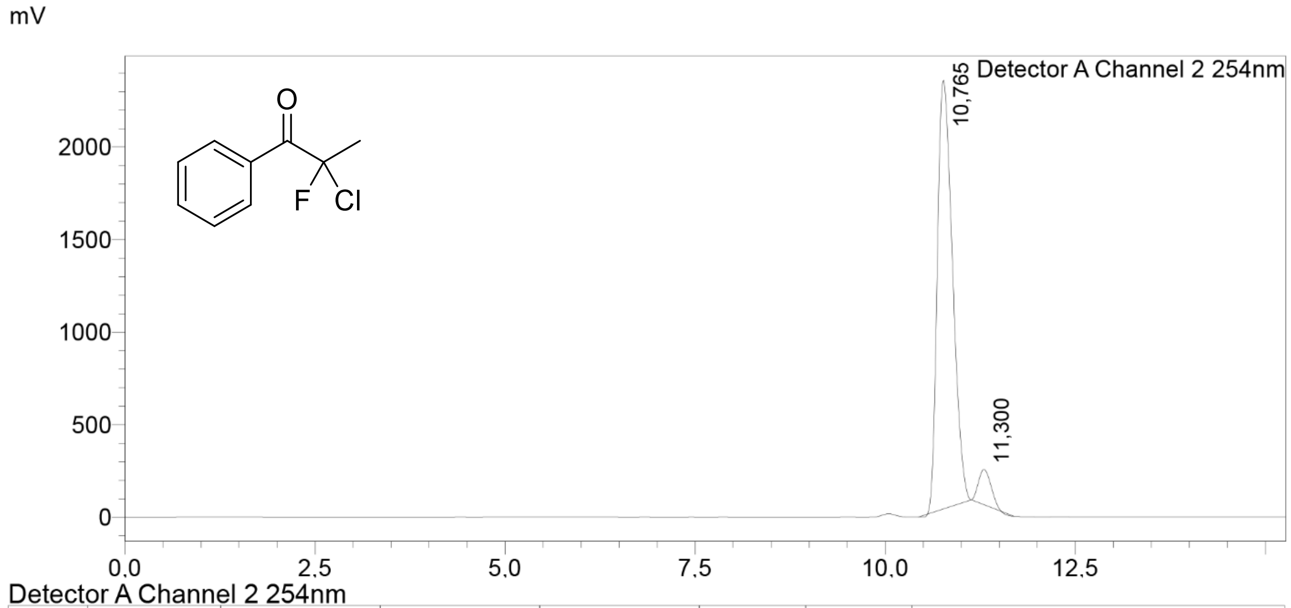

Detector A Channel 2 254nm

Peak#	Ret. Time	Area	Height	Conc.	Unit	Mark	Name
1	10,765	32069418	2315191	93,671		M	
2	11,300	2166895	191076	6,329		M	
Total		34236313	2506268				

Chromatogram of racemic molecule **3b** (via achiral ylide **2b**):

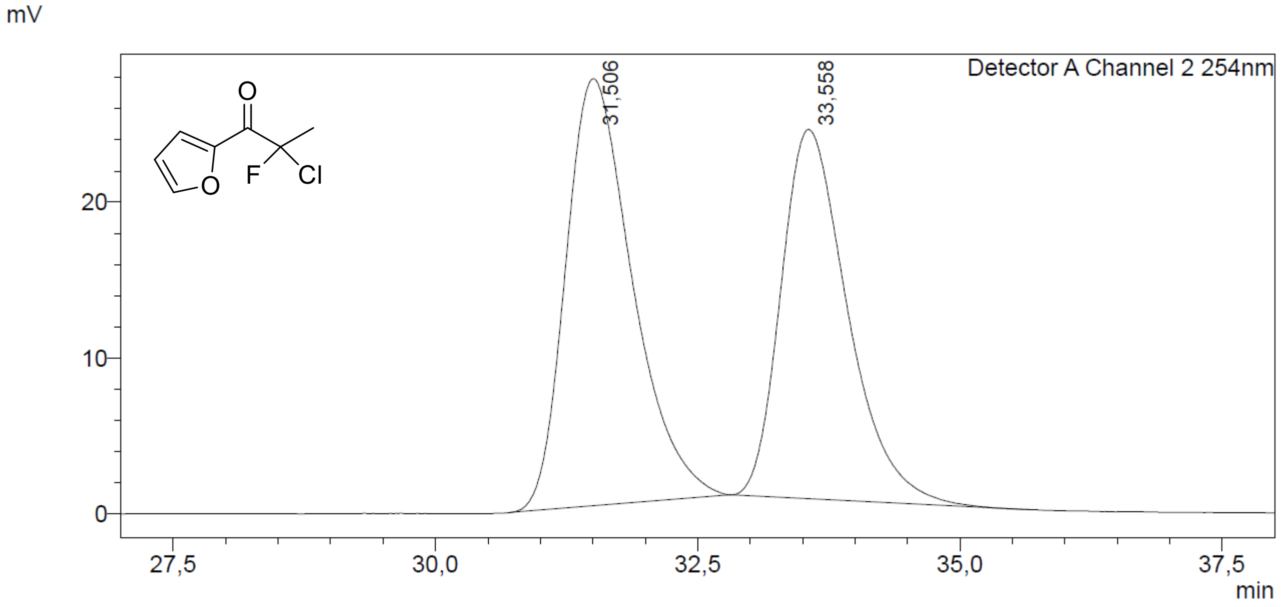

Detector A Channel 2 254nm							
Peak#	Ret. Time	Area	Height	Conc.	Unit	Mark	Name
1	31,506	1211017	27394	53,577		M	
2	33,558	1049310	23690	46,423		M	
Total		2260326	51084				

Chromatogram of enantioenriched molecule **3b** (via achiral ylide **2b**):

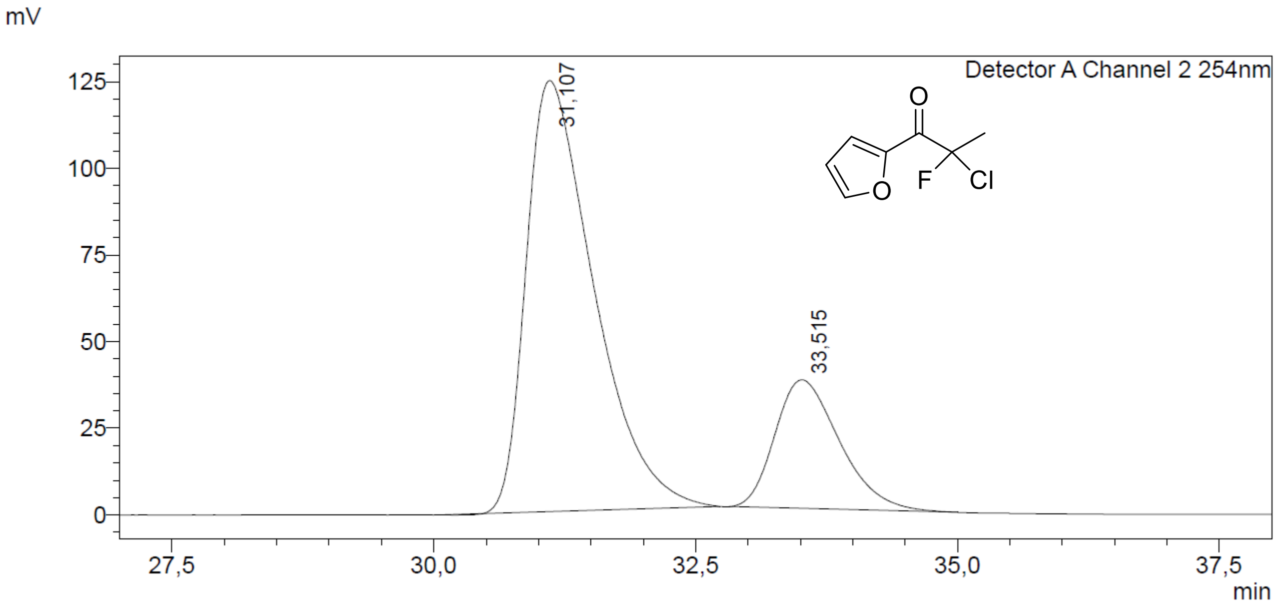

Detector A Channel 2 254nm							
Peak#	Ret. Time	Area	Height	Conc.	Unit	Mark	Name
1	31,107	5601162	124389	77,830		M	
2	33,515	1595545	37114	22,170		M	
Total		7196707	161503				

Chromatogram of racemic molecule **3b** (via chiral ylide **1b**):

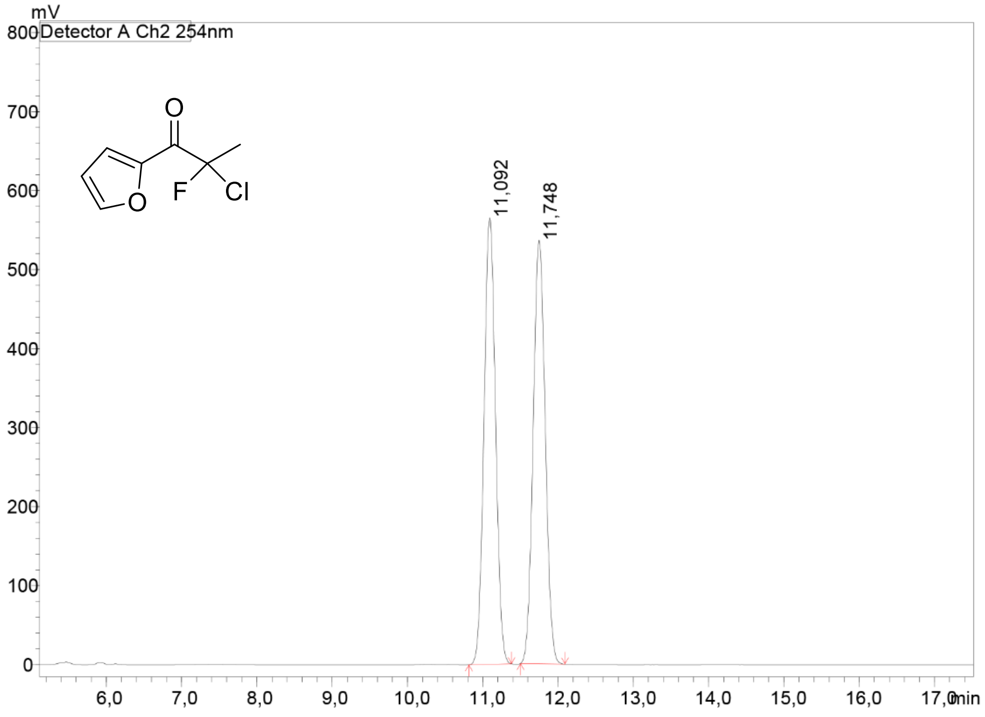

Peak Table

Detector A Channel 2 254nm

Peak#	Ret. Time	Area	Height	Area%	Height%
1	11,092	6013143	565143	50,095	51,320
2	11,748	5990335	536070	49,905	48,680
Total		12003478	1101213	100,000	100,000

Chromatogram of enantioenriched molecule **3b** (via chiral ylide **1b**):

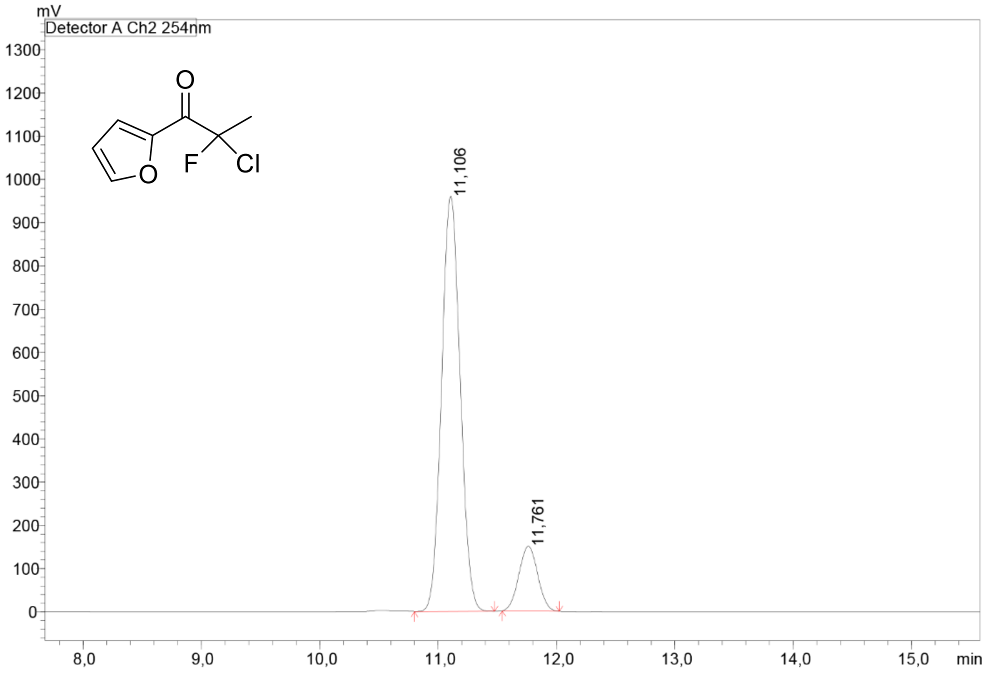

Peak Table

Detector A Channel 2 254nm

Peak#	Ret. Time	Area	Height	Conc.	Area%	Height%
1	11,106	10504827	959609	86,329	86,329	86,482
2	11,761	1663492	149991	13,671	13,671	13,518
Total		12168319	1109600		100,000	100,000

Chromatogram of racemic molecule **3c**:

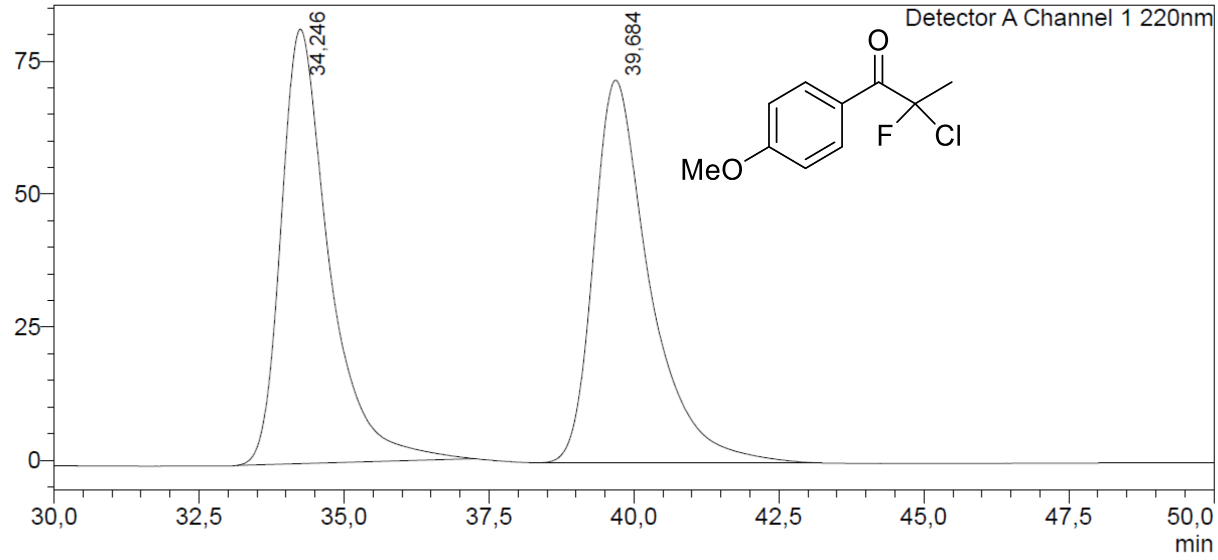

Peak#	Ret. Time	Area	Height	Conc.	Unit	Mark	Name
1	34,246	4731481	81627	49,332		M	
2	39,684	4859621	71915	50,668		M	
Total		9591102	153542				

Chromatogram of enantioenriched molecule **3c** (via chiral ylide **1c**):

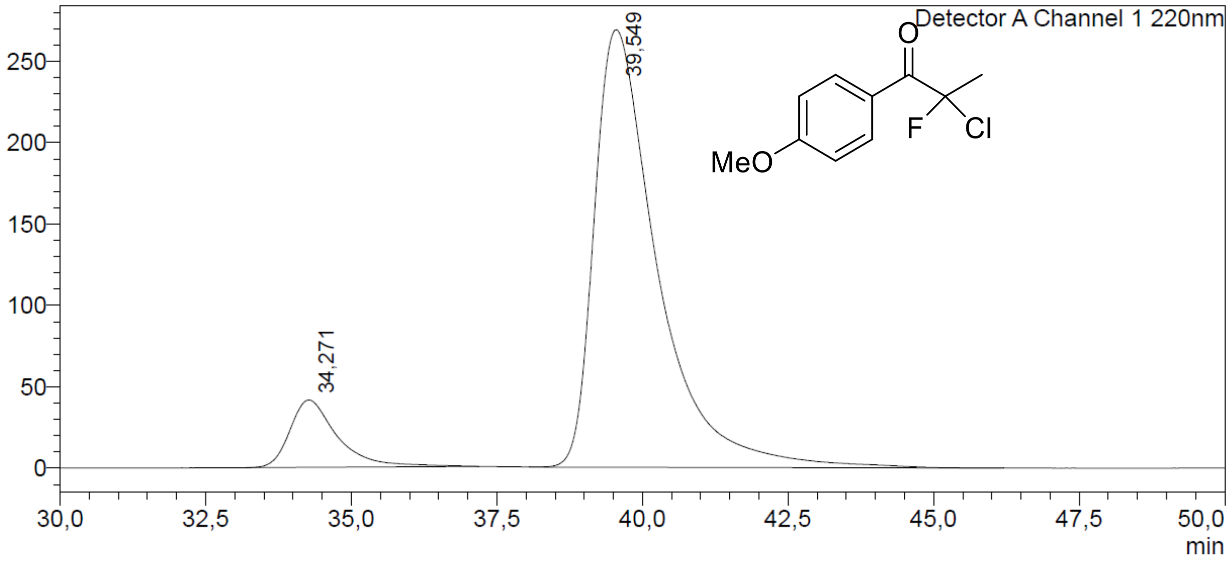

Peak#	Ret. Time	Area	Height	Conc.	Unit	Mark	Name
1	34,271	2350977	41388	10,370		M	
2	39,549	20320674	268675	89,630		M	
Total		22671651	310063				

Chromatogram of enantioenriched molecule **3c** (via achiral ylide **2c**):

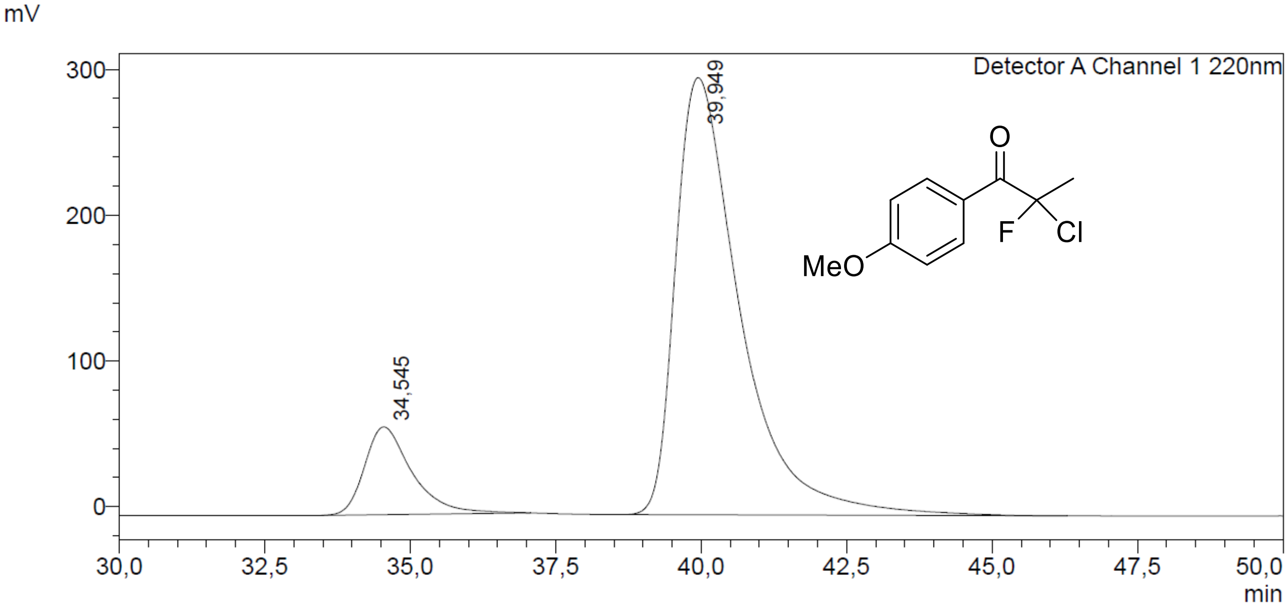

Peak#	Ret. Time	Area	Height	Conc.	Unit	Mark	Name
1	34,545	3422155	60163	12,841		M	
2	39,949	23228082	299947	87,159		M	
Total		26650237	360111				

Chromatogram of racemic molecule **3d**:

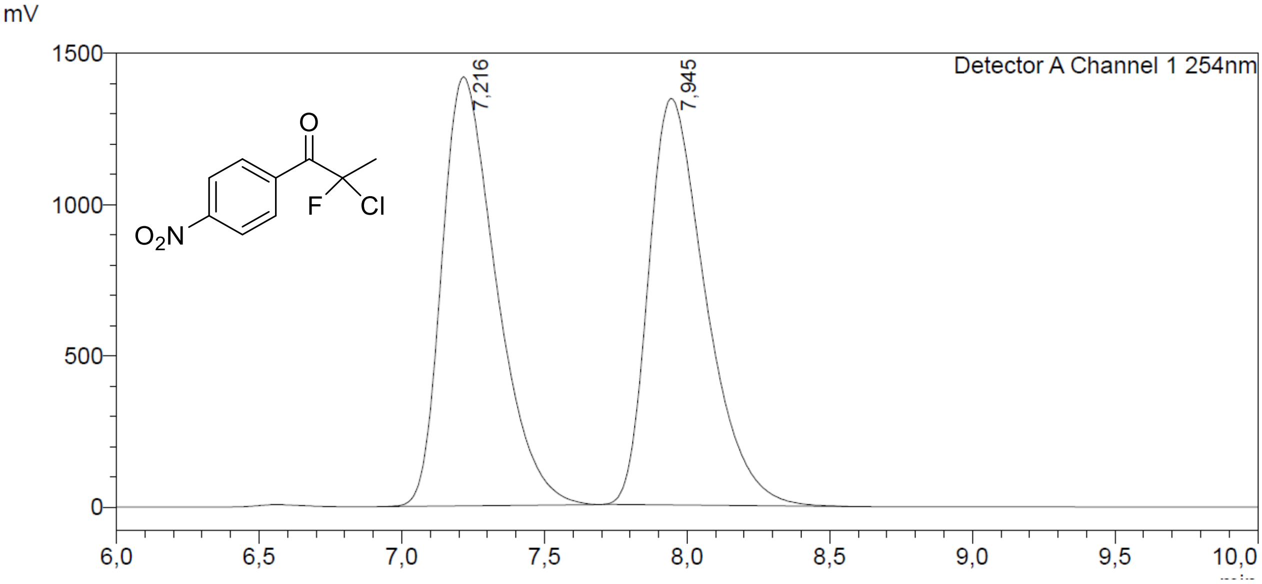

Peak#	Ret. Time	Area	Height	Conc.	Unit	Mark	Name
1	7,216	18773606	1417041	50,118		M	
2	7,945	18684995	1342571	49,882		M	
Total		37458601	2759612				

Chromatogram of enantioenriched molecule **3d** (via achiral ylide **2d**):

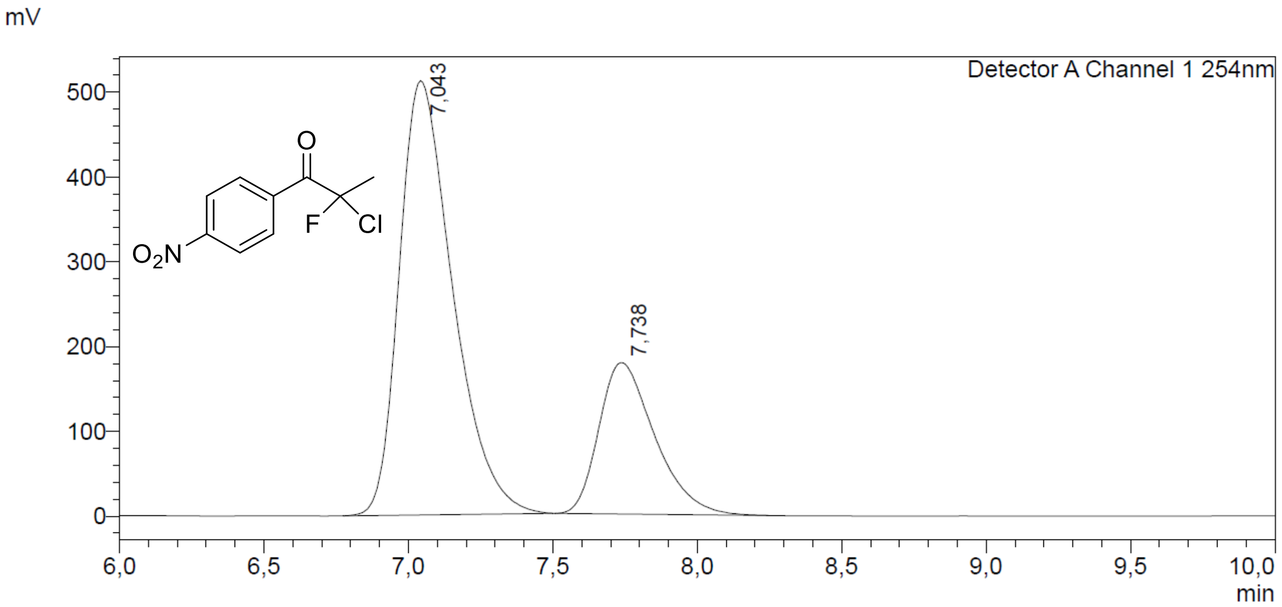

Peak#	Ret. Time	Area	Height	Conc.	Unit	Mark	Name
1	7,043	6592536	511844	73,297		M	
2	7,738	2401801	178478	26,703		M	
Total		8994338	690322				

Chromatogram of enantioenriched molecule **3d** (via chiral ylide **1d**):

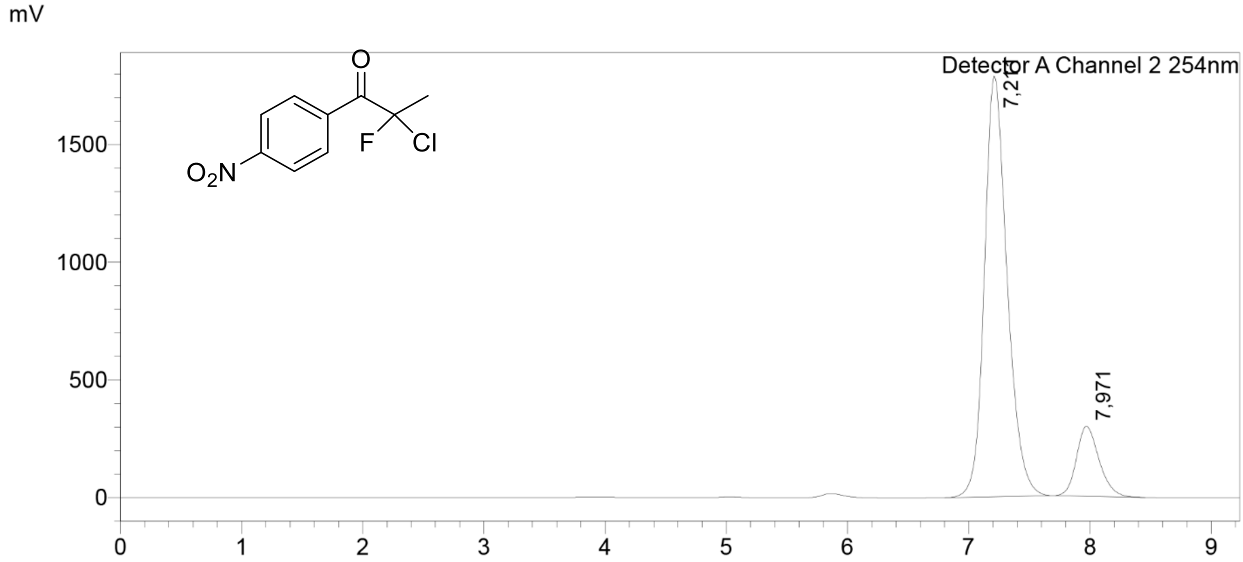

Detector A Channel 2 254nm

Peak#	Ret. Time	Area	Height	Conc.	Unit	Mark	Name
1	7,211	23307491	1786790	85,827		M	
2	7,971	3848918	296993	14,173		M	
Total		27156409	2083783				

Chromatogram of racemic molecule **3e**:

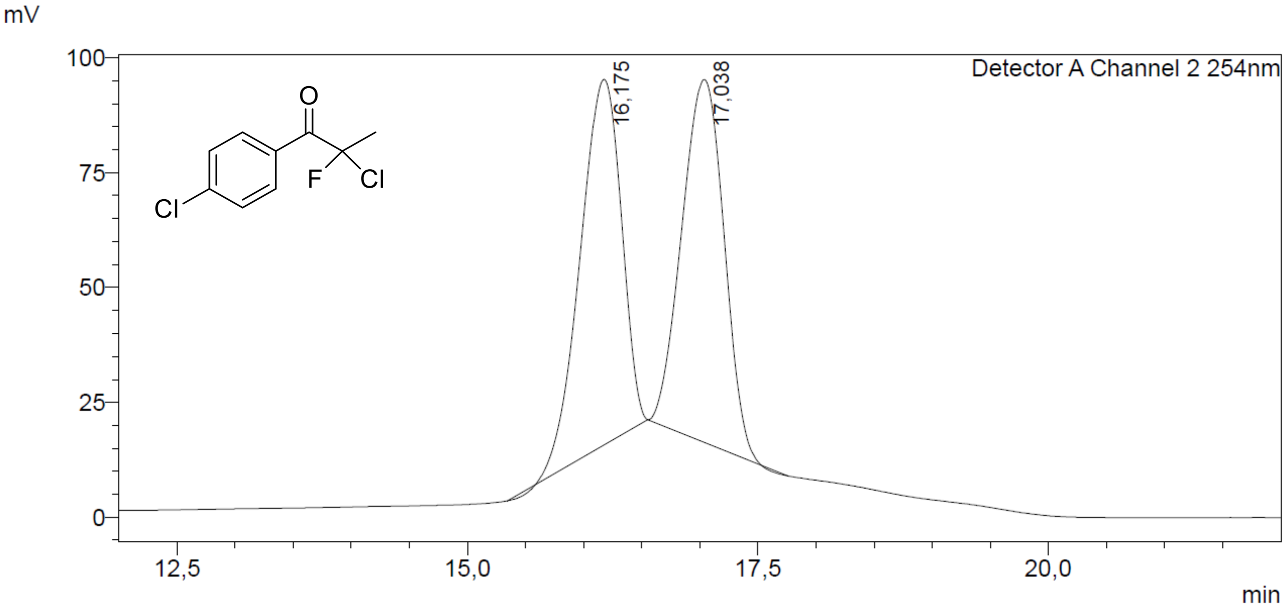

Detector A Channel 2 254nm

Peak#	Ret. Time	Area	Height	Conc.	Unit	Mark	Name
1	16,175	1965215	79518	49,690		M	
2	17,038	1989757	78922	50,310		M	
Total		3954972	158440				

Chromatogram of enantioenriched molecule **3e** (via chiral ylide **1e**):

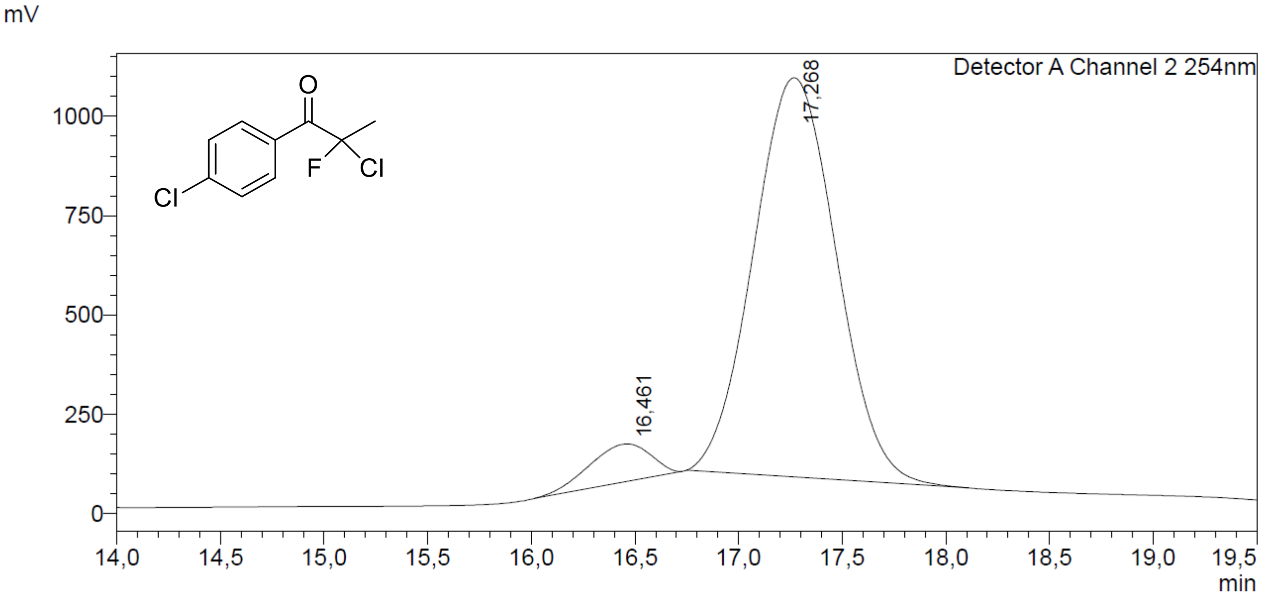

Detector A Channel 2 254nm

Peak#	Ret. Time	Area	Height	Conc.	Unit	Mark	Name
1	16,461	1989639	94465	6,638		M	
2	17,268	27984596	1004816	93,362		M	
Total		29974235	1099280				

Chromatogram of enantioenriched molecule **3e** (via achiral ylide **2f**):

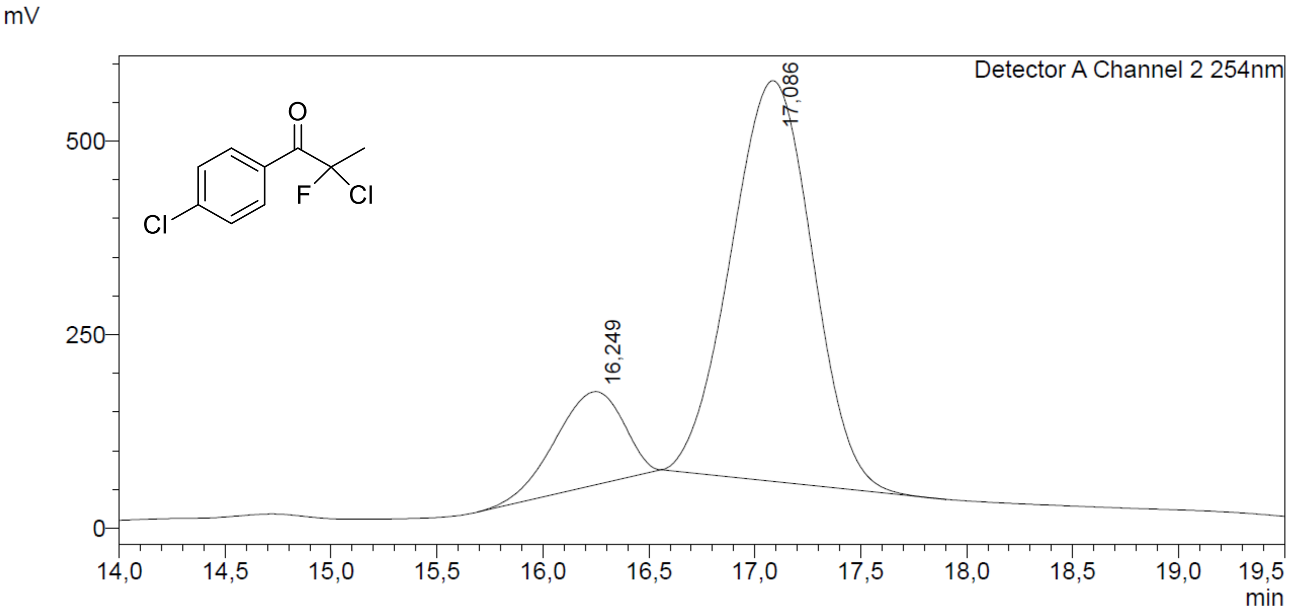

Detector A Channel 2 254nm

Peak#	Ret. Time	Area	Height	Conc.	Unit	Mark	Name
1	16,249	2744486	120493	16,505		M	
2	17,086	13883746	517594	83,495		M	
Total		16628232	638087				

Chromatogram of racemic molecule **3f**:

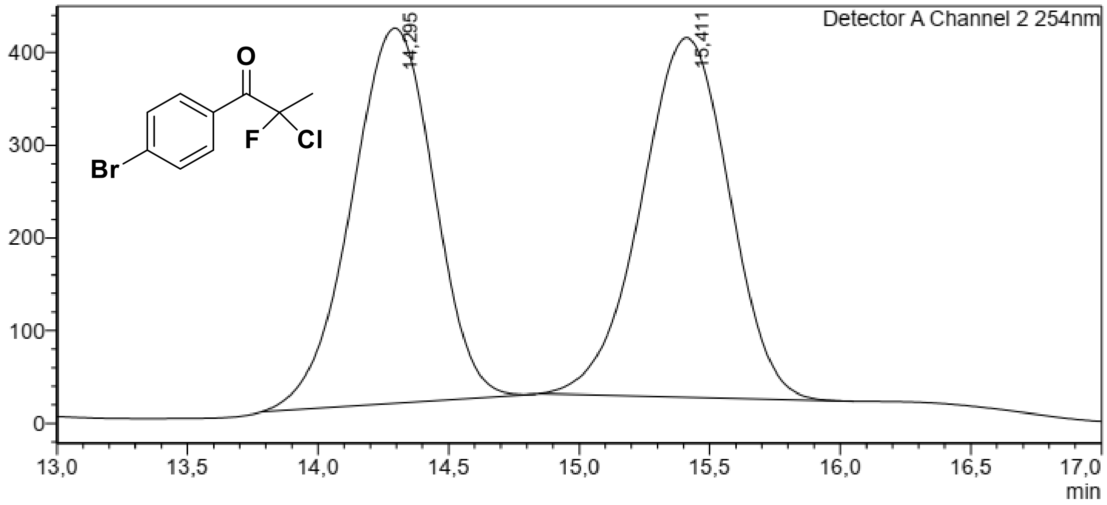

Detector A Channel 2 254nm

Peak#	Ret. Time	Area	Height	Conc.	Unit	Mark	Name
1	14,295	8927777	404708	49,435		M	
2	15,411	9131720	387752	50,565		M	
Total		18059498	792460				

Chromatogram of enantioenriched molecule **3f** (via achiral ylide **2g**):

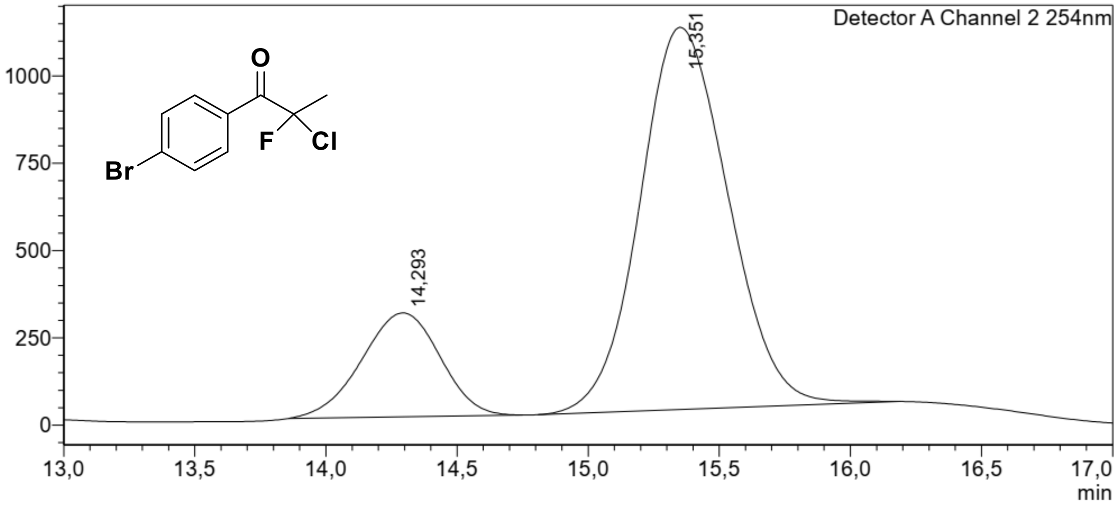

Detector A Channel 2 254nm

Peak#	Ret. Time	Area	Height	Conc.	Unit	Mark	Name
1	14,293	6204857	297486	19,434		M	
2	15,351	25723240	1094897	80,566		M	
Total		31928098	1392383				

Chromatogram of enantioenriched molecule **3f** (via chiral ylide **1f**):

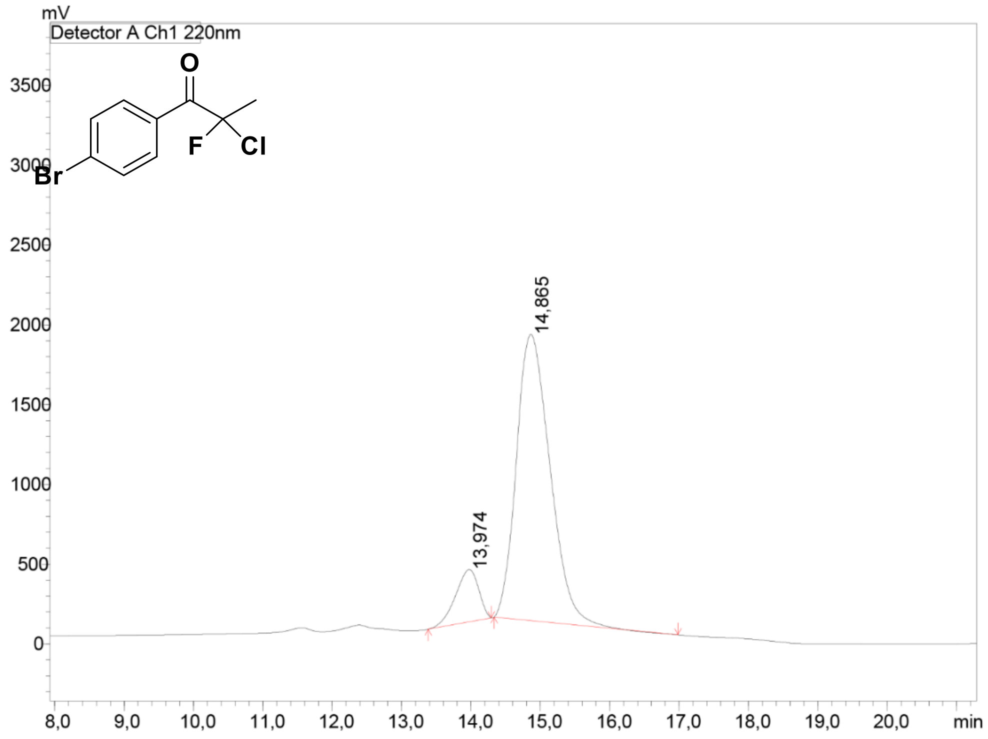

Detector A Channel 1 220nm

Peak#	Ret. Time	Area	Height	Area%	Height%
1	13,974	7610978	327773	11,174	15,448
2	14,865	60505188	1793986	88,826	84,552
Total		68116166	2121759	100,000	100,000

Chromatogram of racemic molecule **3g** (via achiral ylide **2h**):

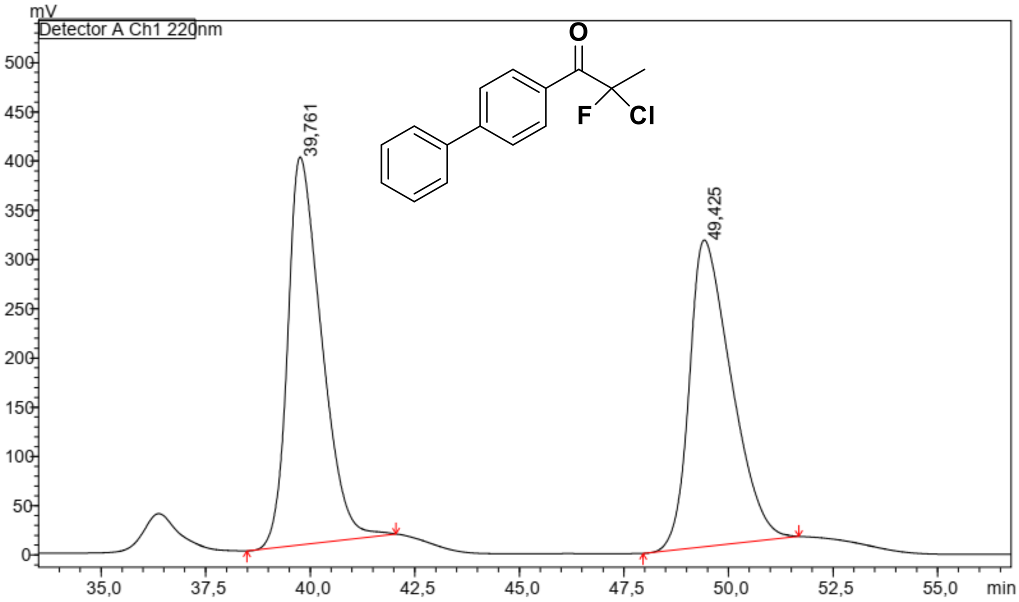

Peak Table

Detector A Channel 1 220nm

Peak#	Ret. Time	Area	Height	Height%	Area%
1	39,761	22190544	393783	55,841	50,493
2	49,425	21757625	311404	44,159	49,507
Total		43948169	705187	100,000	100,000

Chromatogram of enantioenriched molecule **3g** (via achiral ylide **2h**):

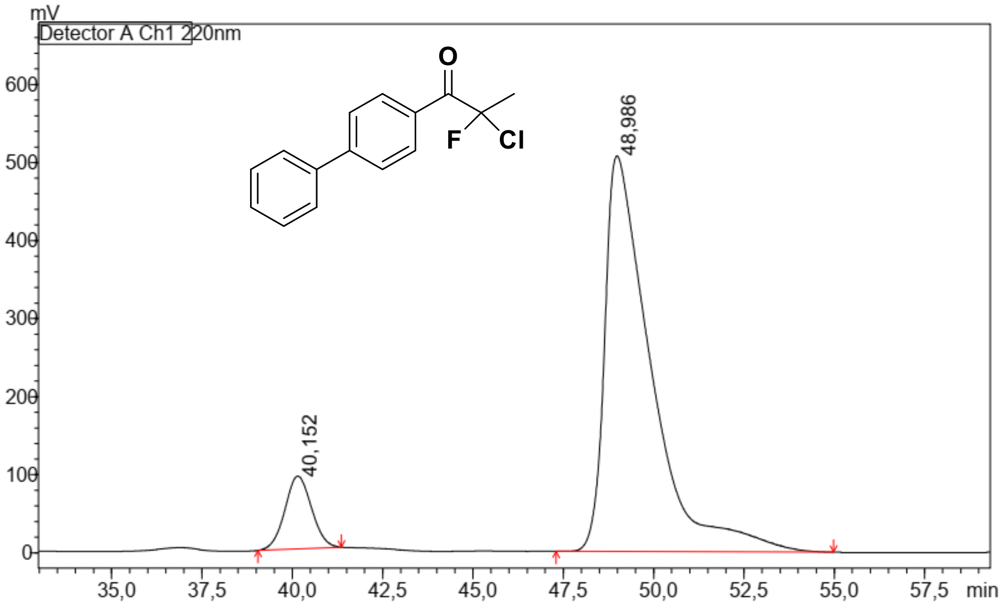

Peak Table

Detector A Channel 1 220nm

Peak#	Ret. Time	Area	Height	Height%	Area%
1	40,152	4789164	93249	15,534	9,609
2	48,986	45049801	507038	84,466	90,391
Total		49838965	600287	100,000	100,000

Chromatogram of racemic molecule **3g** (via chiral ylide **1h**):

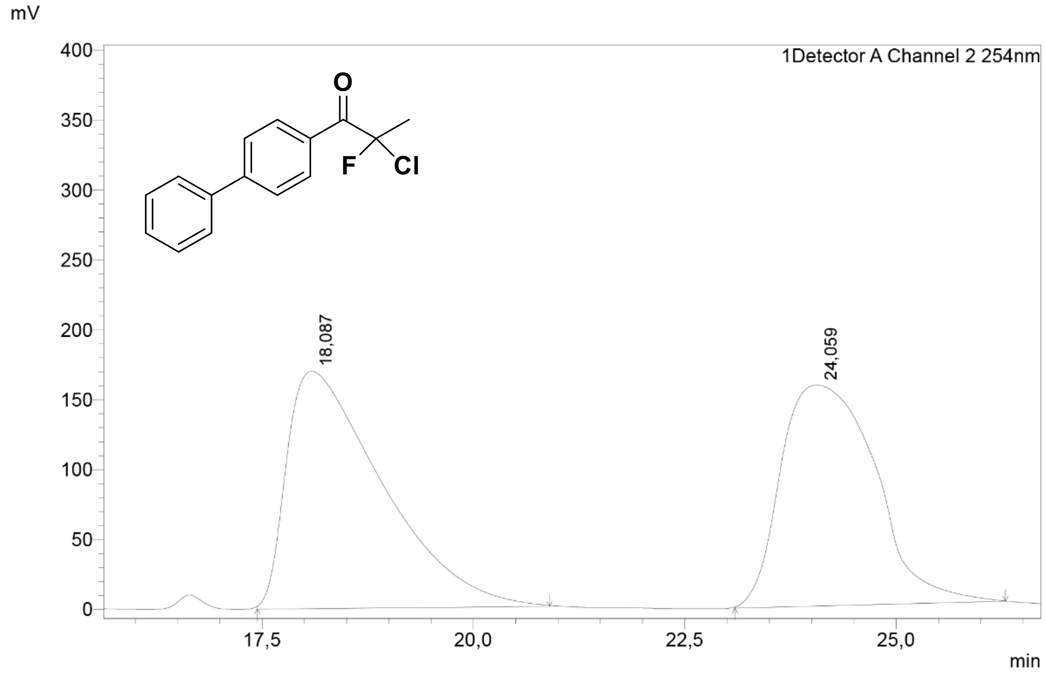

<Peak Table>

Detector A Channel 2 254nm

Peak#	Ret. Time	Area	Height	Conc.	Unit	Mark	Name
1	18,087	13413355	169744	52,071		M	
2	24,059	12346576	158219	47,929		M	
Total		25759932	327963				

Chromatogram of enantioenriched molecule **3g** (via chiral ylide **1h**):

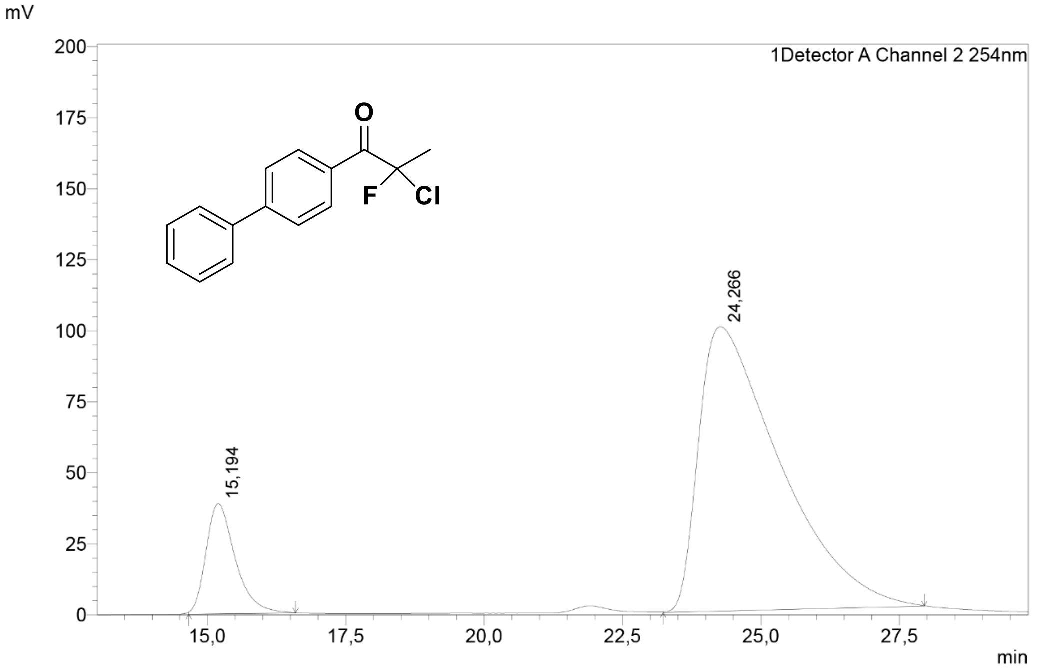

<Peak Table>

Detector A Channel 2 254nm

Peak#	Ret. Time	Area	Height	Conc.	Unit	Mark	Name
1	15,194	1429083	38766	12,221		M	
2	24,266	10264981	100047	87,779		M	
Total		11694064	138813				

Chromatogram of racemic molecule **3h**:

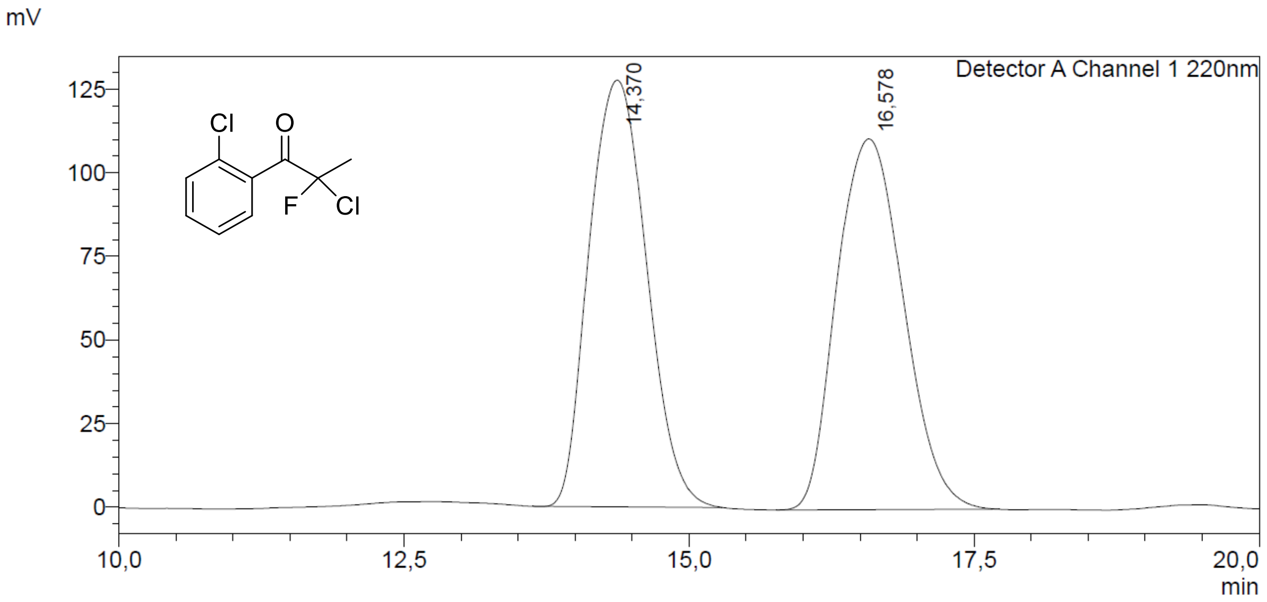

Peak#	Ret. Time	Area	Height	Conc.	Unit	Mark	Name
1	14,370	4497275	127595	49,827		M	
2	16,578	4528549	110895	50,173		M	
Total		9025824	238489				

Chromatogram of enantioenriched molecule **3h** (via chiral ylide **1j**):

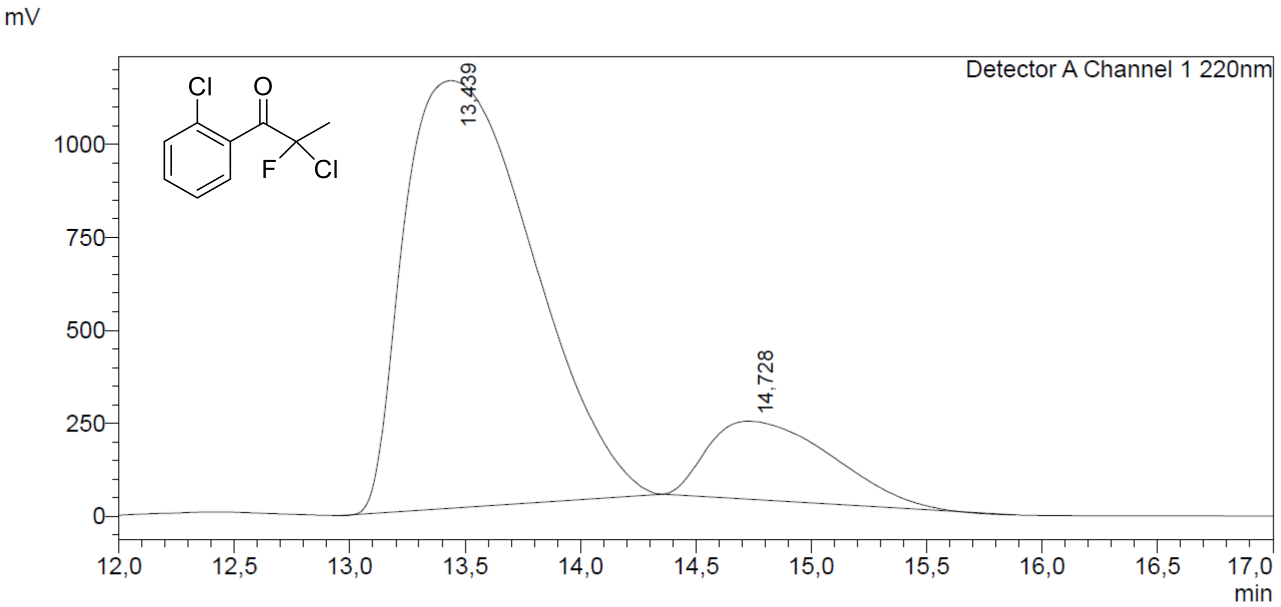

Peak#	Ret. Time	Area	Height	Conc.	Unit	Mark	Name
1	13,439	44630167	1149894	84,884		M	
2	14,728	7947517	209613	15,116		M	
Total		52577684	1359507				

Chromatogram of enantioenriched molecule **3h** (via achiral ylide **2i**):

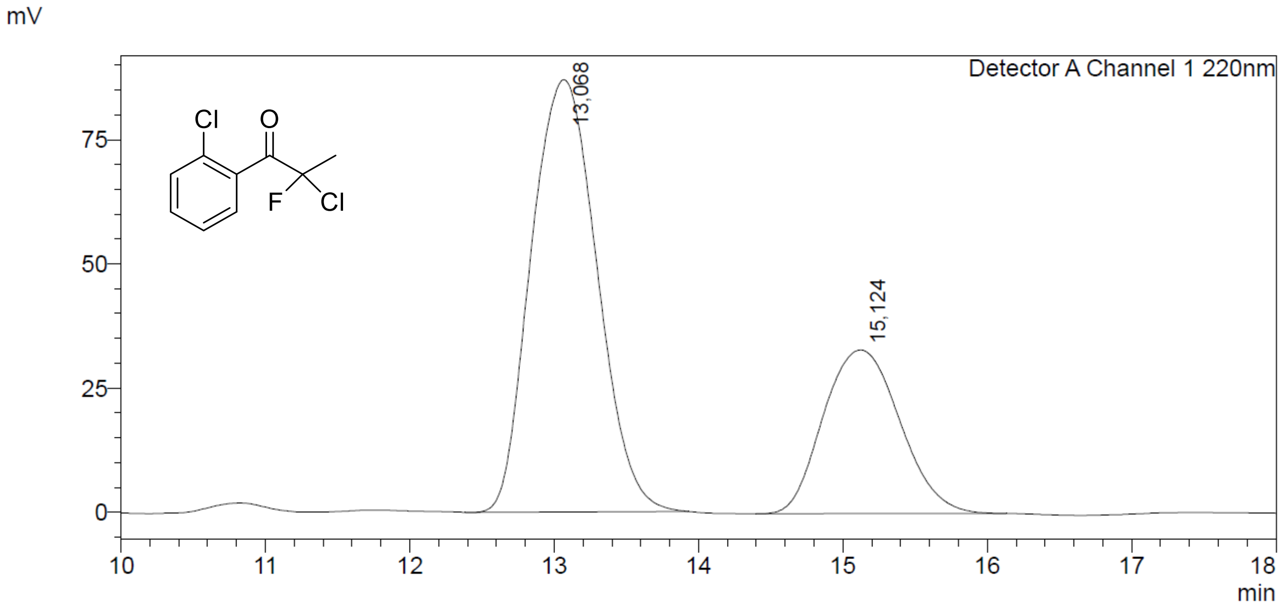

Peak#	Ret. Time	Area	Height	Conc.	Unit	Mark	Name
1	13,068	2767467	87005	69,451		M	
2	15,124	1217306	32945	30,549		M	
Total		3984773	119950				

Chromatogram of racemic molecule **3i** (via achiral ylide **2j**):

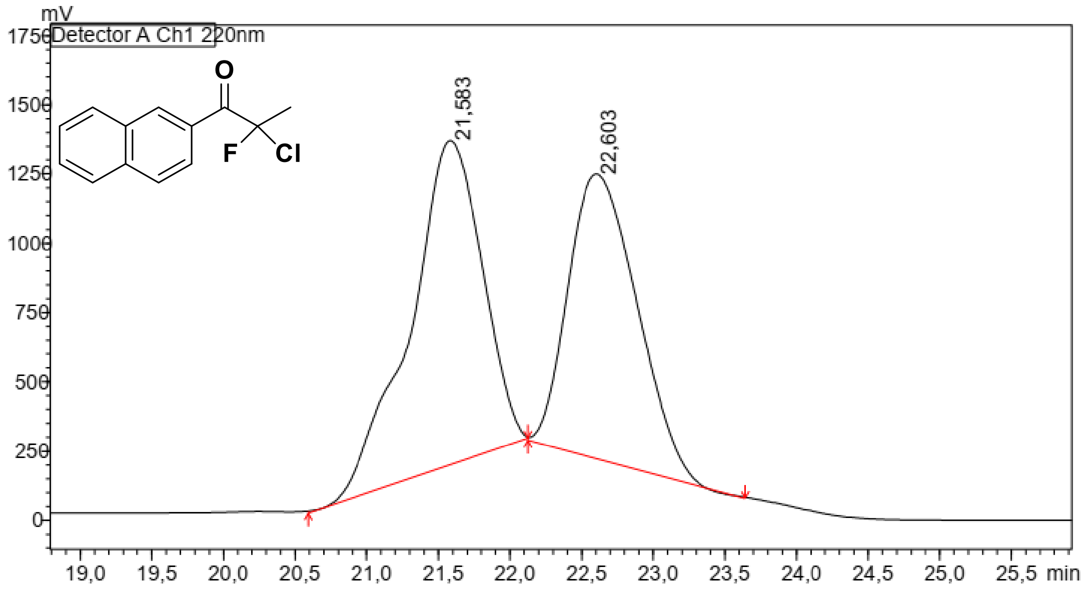

Peak Table

Detector A Channel 1 220nm

Peak#	Ret. Time	Area	Height	Height%	Area%
1	21,583	40829422	1169843	53,240	54,226
2	22,603	34465014	1027476	46,760	45,774
Total		75294436	2197319	100,000	100,000

Chromatogram of enantioenriched molecule **3i** (via achiral ylide **2j**):

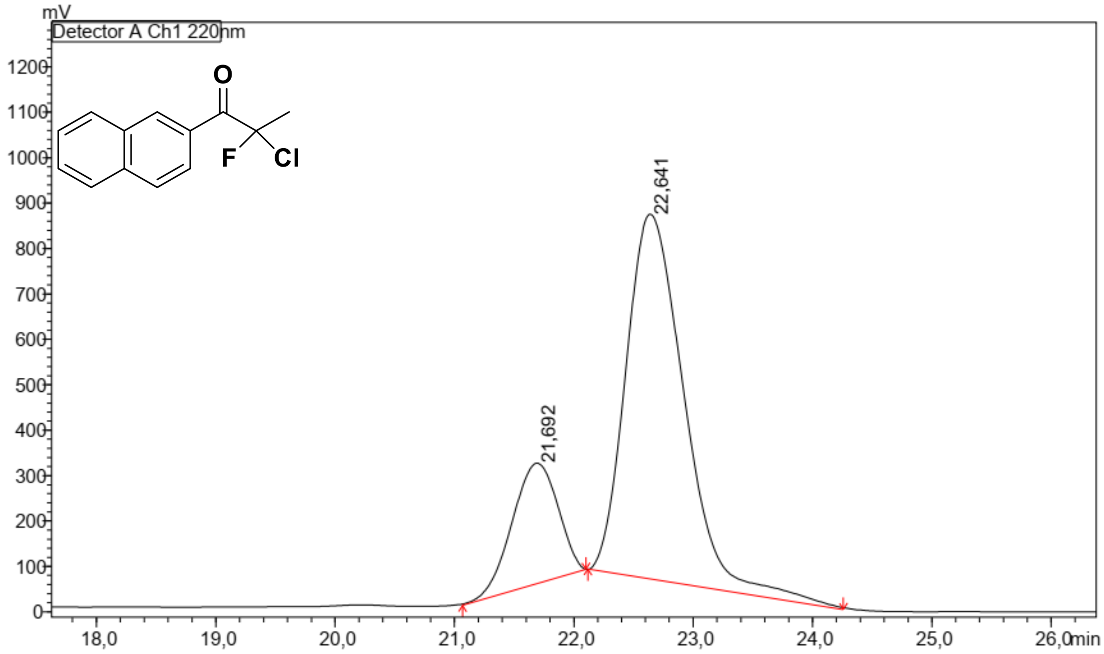

Peak Table

Detector A Channel 1 220nm

Peak#	Ret. Time	Area	Height	Height%	Area%
1	21,692	7092811	264750	24,792	20,677
2	22,641	27210782	803149	75,208	79,323
Total		34303593	1067899	100,000	100,000

Chromatogram of racemic molecule **3i** (via chiral ylide **1i**):

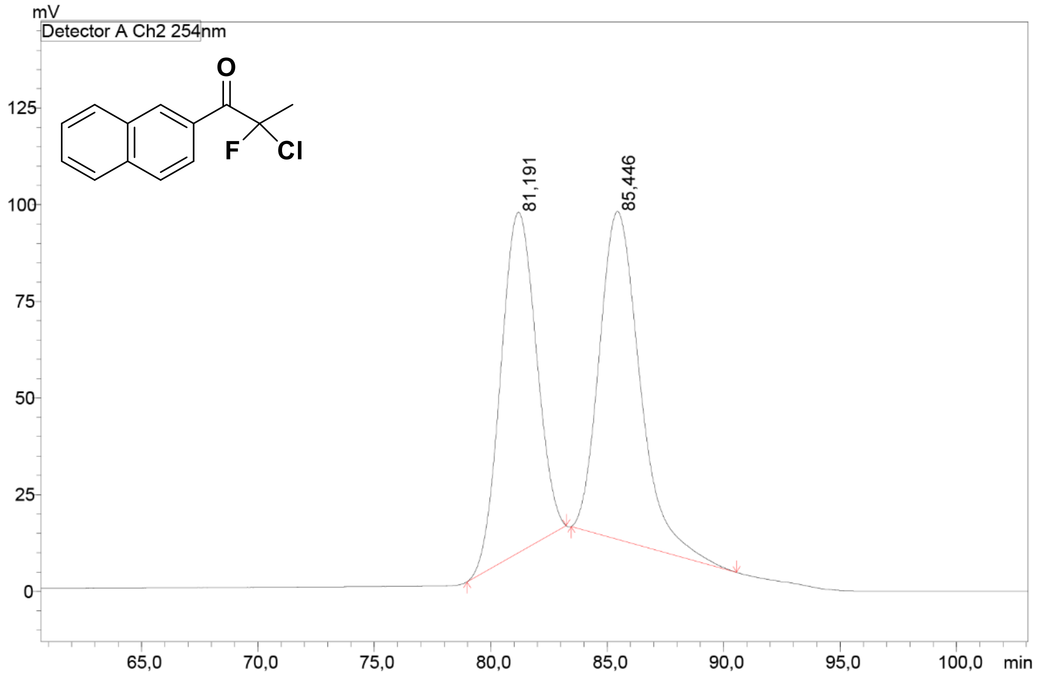

Detector A Channel 2 254nm

Peak#	Ret. Time	Area	Area%	Height%
1	81,191	9315997	47,644	50,950
2	85,446	10237186	52,356	49,050
Total		19553183	100,000	100,000

Chromatogram of enantioenriched molecule **3i** (via chiral ylide **1i**):

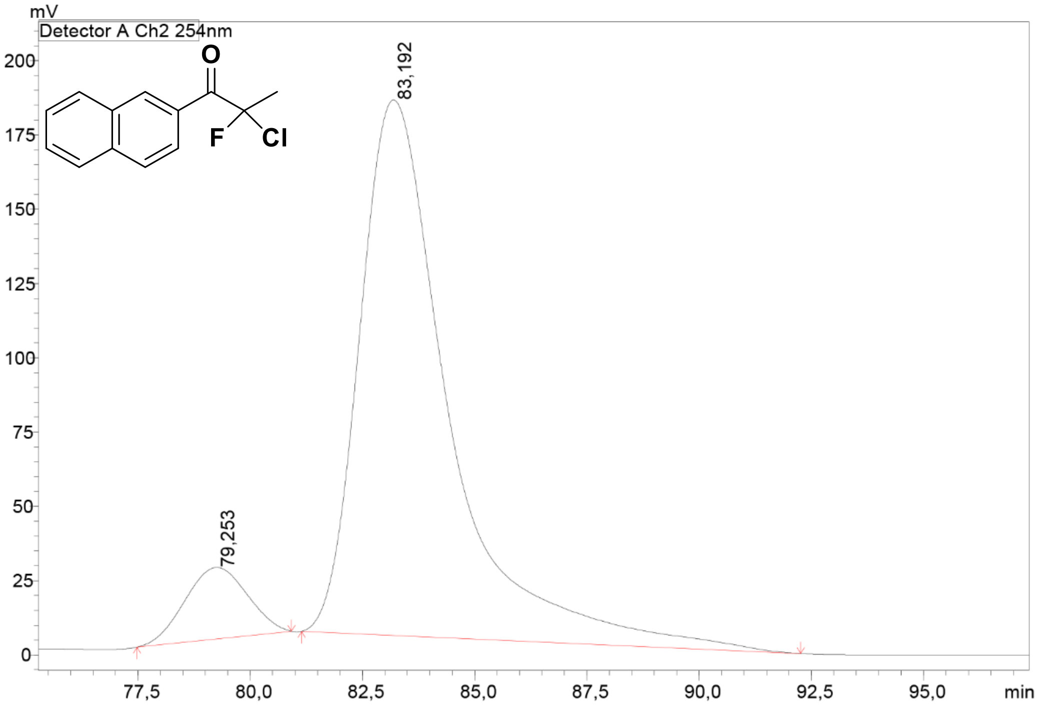

Detector A Channel 2 254nm

Peak#	Ret. Time	Area	Height	Unit	Height%	Area%
1	79,253	2326588	24014		11,757	8,529
2	83,192	24952632	180242		88,243	91,471
Total		27279220	204257		100,000	100,000

Chromatogram of racemic molecule **3j** (via chiral ylide **1k**):

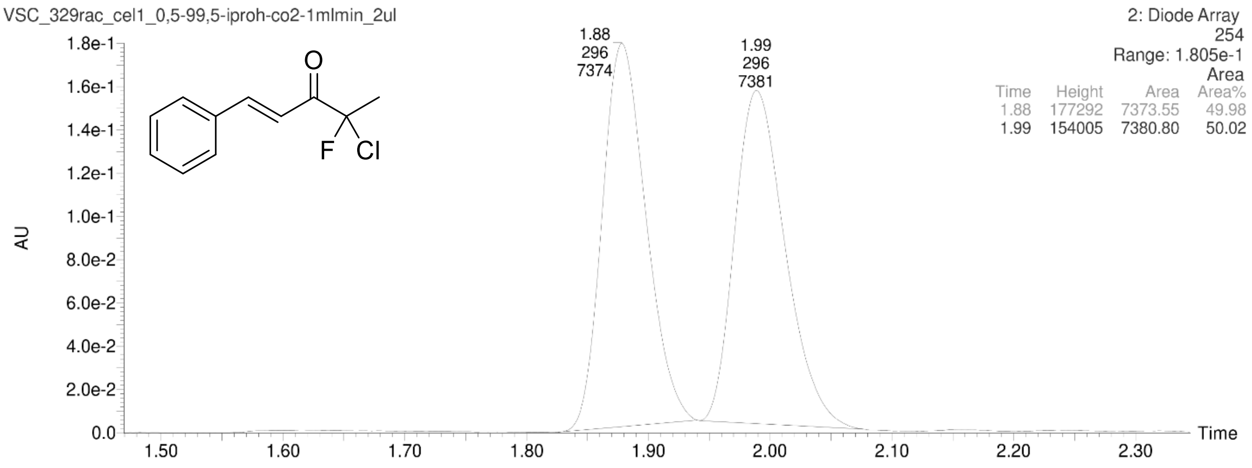

Chromatogram of enantioenriched molecule **3j** (via chiral ylide **1k**):

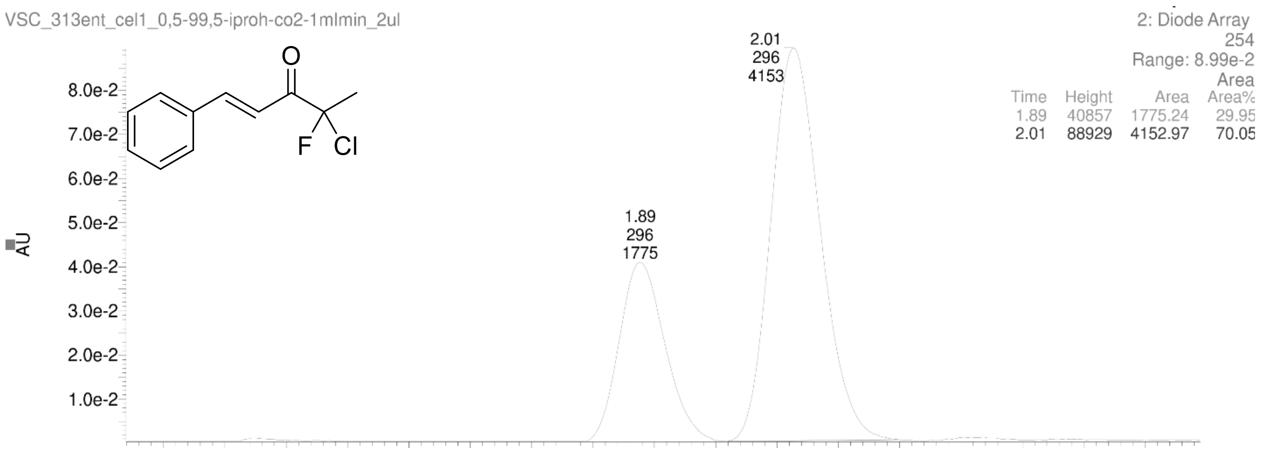

Chromatogram of racemic molecule **3k** (via chiral ylide **1g**):

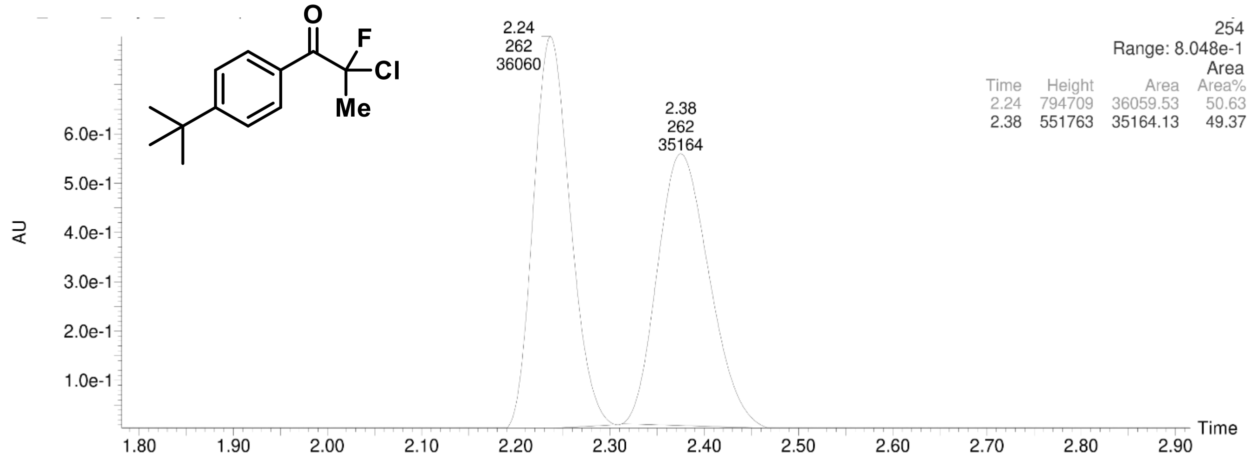

Chromatogram of enantioenriched molecule **3k** (via chiral ylide **1g**):

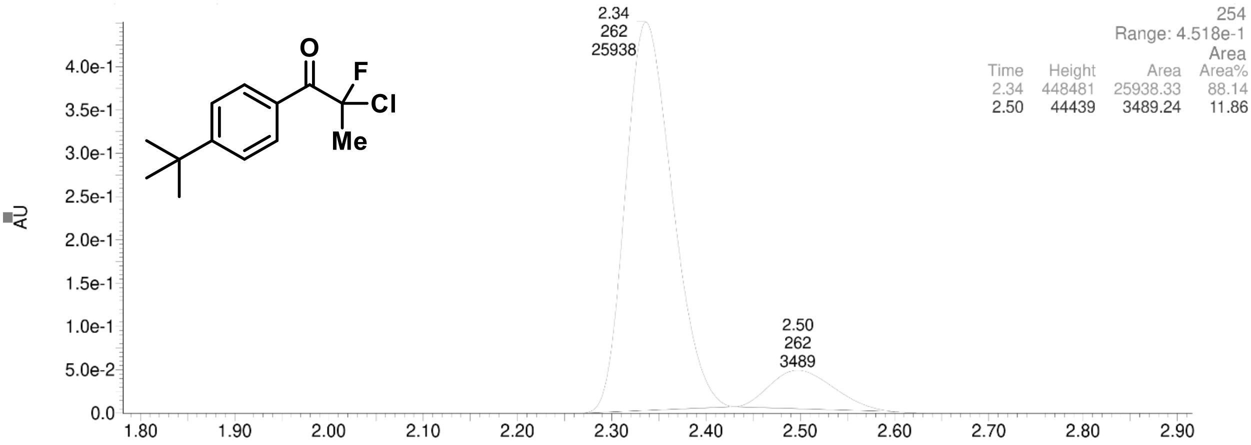

Chromatogram of racemic molecule **3l** (via chiral ylide **1l**):

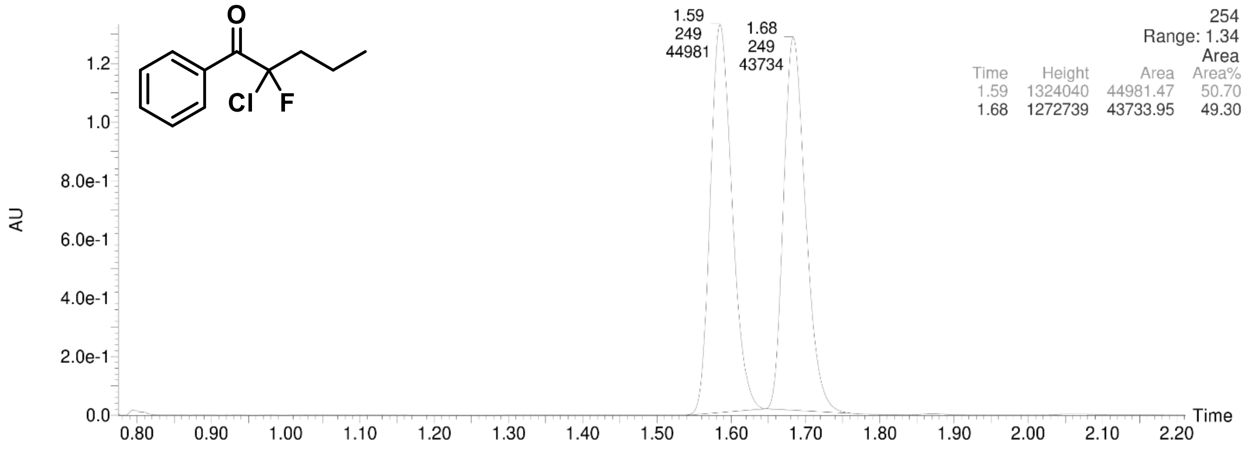

Chromatogram of enantioenriched molecule **3l** (via chiral ylide **1l**):

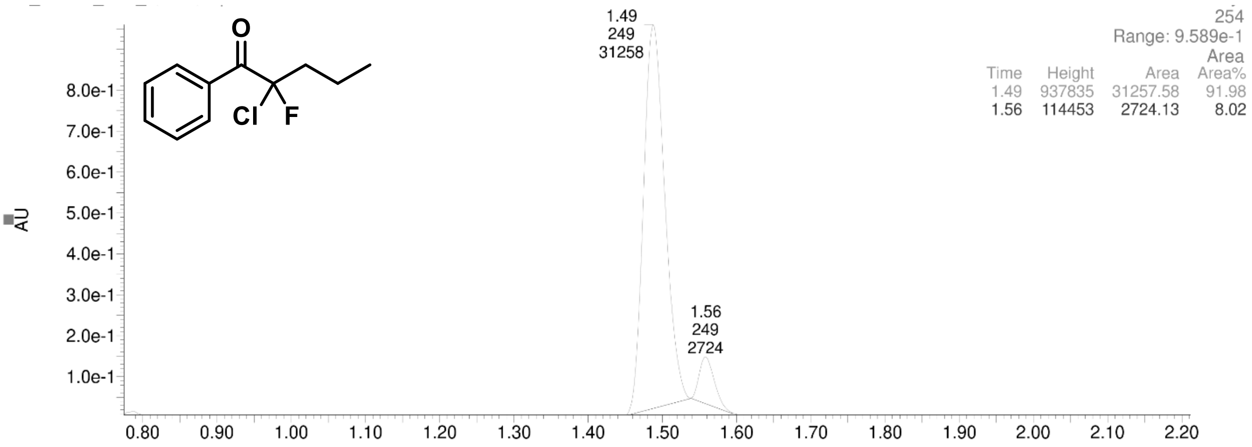

Chromatogram of racemic molecule **3m** (via chiral ylide **1m**):

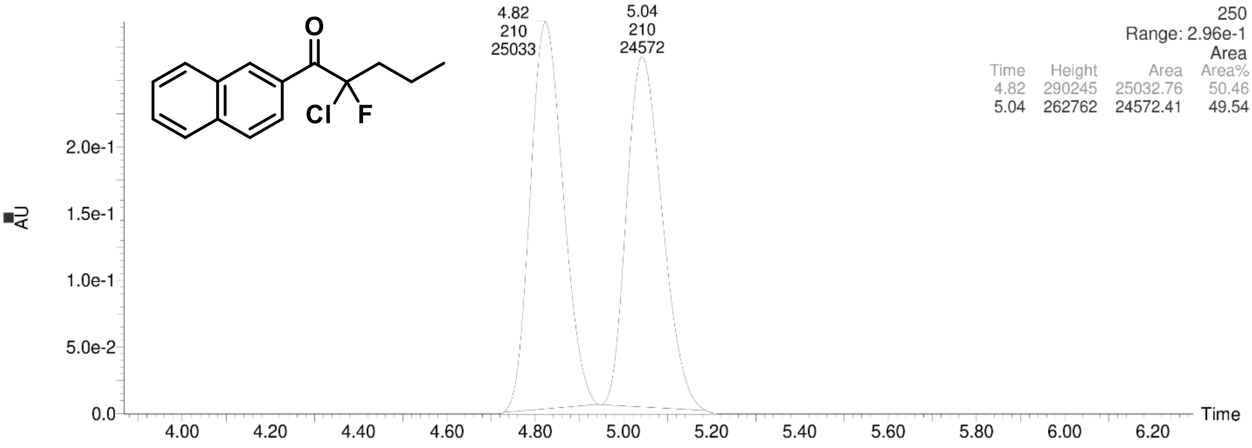

Chromatogram of enantioenriched molecule **3m** (via chiral ylide **1m**):

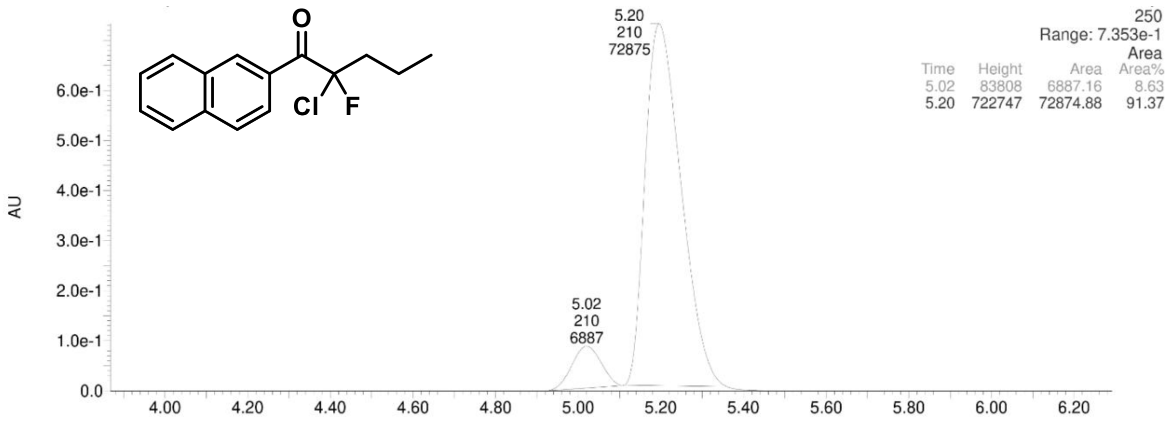

Chromatogram of racemic molecule **3n** (via achiral ylide **2e**):

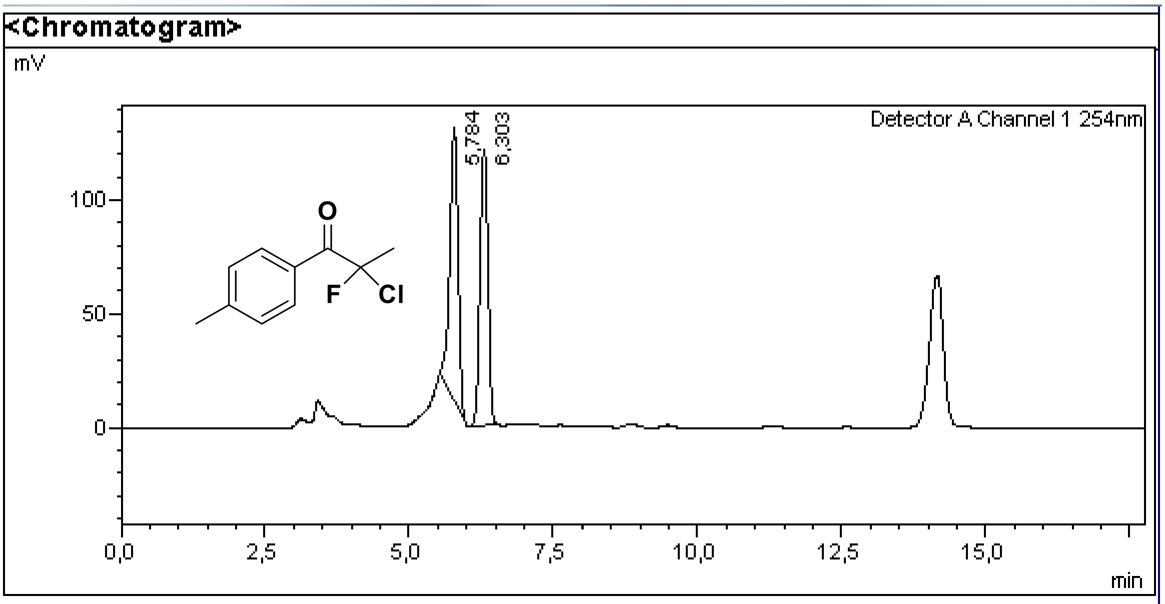

<Peak Table>

Detector A Channel 1 254nm

Peak#	Ret. Time	Area	Height	Conc.	Unit	Mark	Name
1	5,784	1230074	120687	51,364		M	
2	6,303	1164753	121392	48,636		M	

Chromatogram of enantioenriched molecule **3j** (via achiral ylide **2e**):

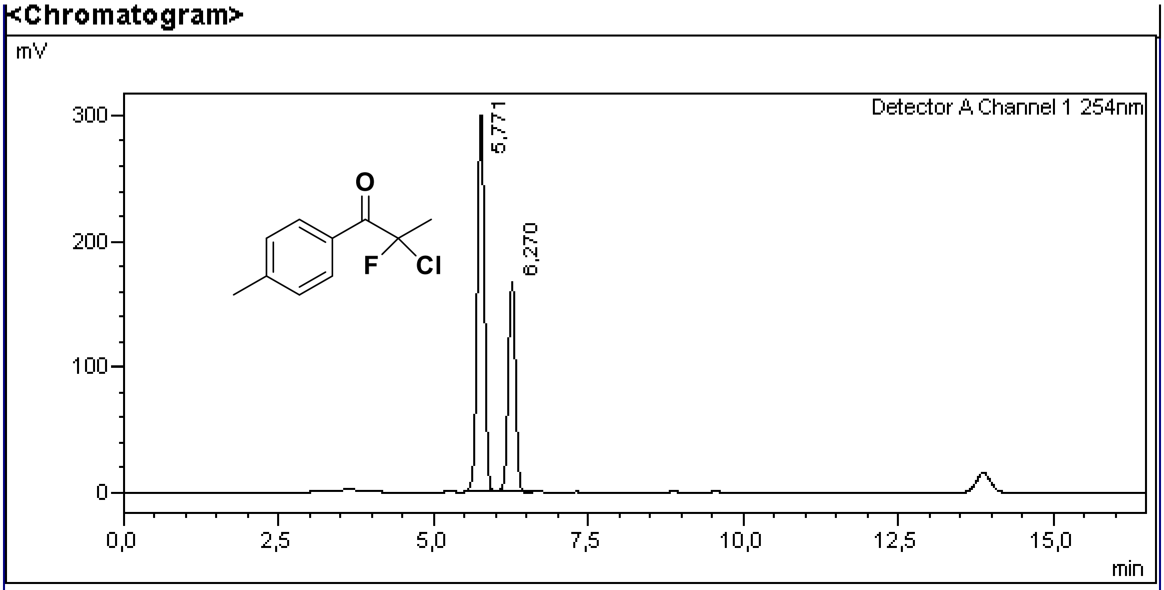

<Peak Table>

Detector A Channel 1 254nm

Peak#	Ret. Time	Area	Height	Conc.	Unit	Mark	Name
1	5,771	2357192	298937	64,063		M	
2	6,270	1322327	166293	35,937		M	

Chromatogram of racemic molecule **4a** (via chiral ylide **1a**):

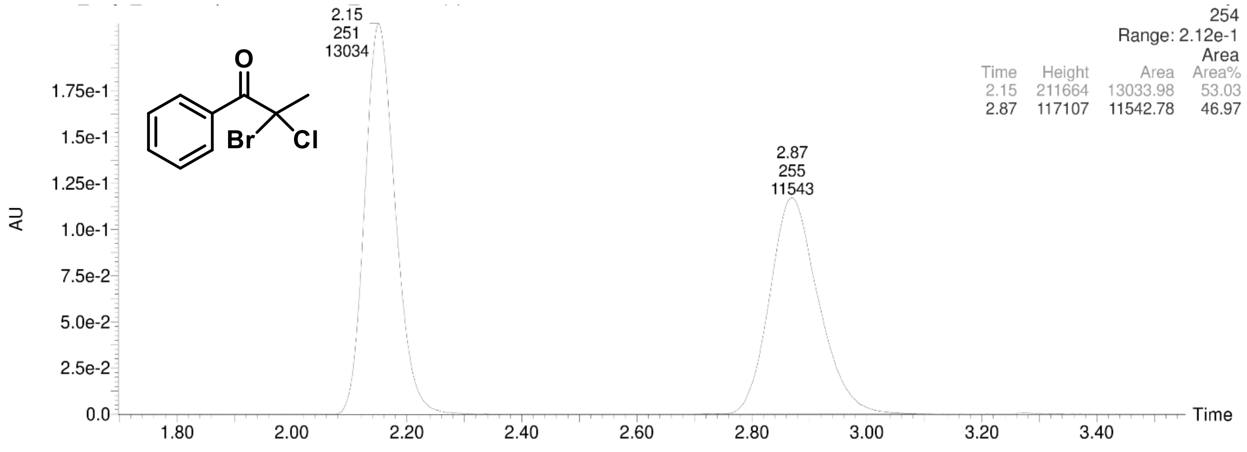

Chromatogram of enantioenriched molecule **4a** (via achiral ylide **1a**):

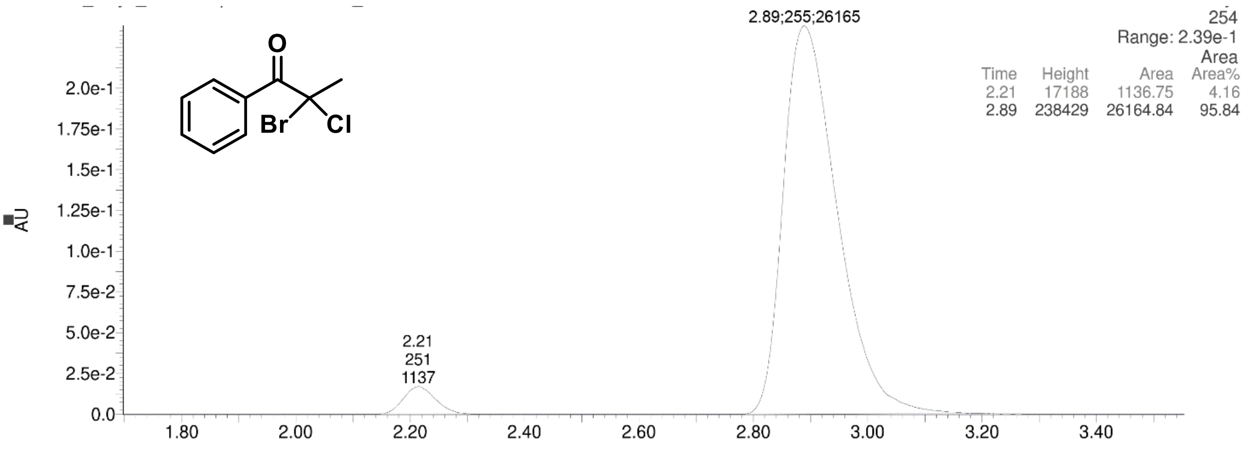

Chromatogram of racemic molecule **3'a**:

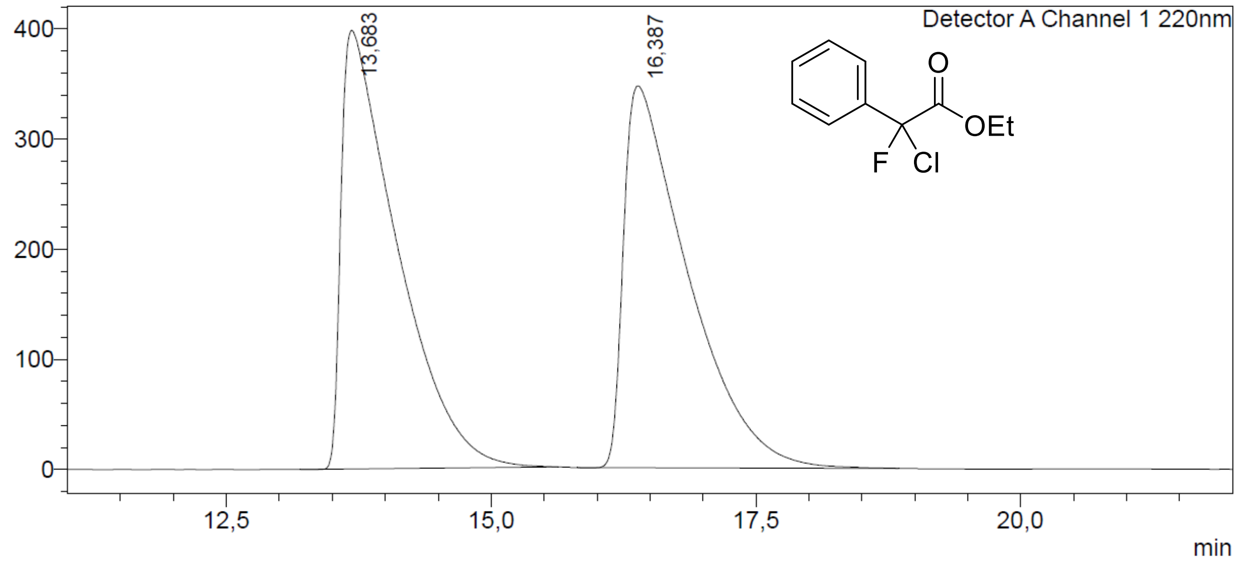

Peak#	Ret. Time	Area	Height	Conc.	Unit	Mark	Name
1	13,683	14644084	398304	49,951		M	
2	16,387	14673050	346856	50,049		M	
Total		29317134	745160				

Chromatogram of enantioenriched molecule **3'a**:

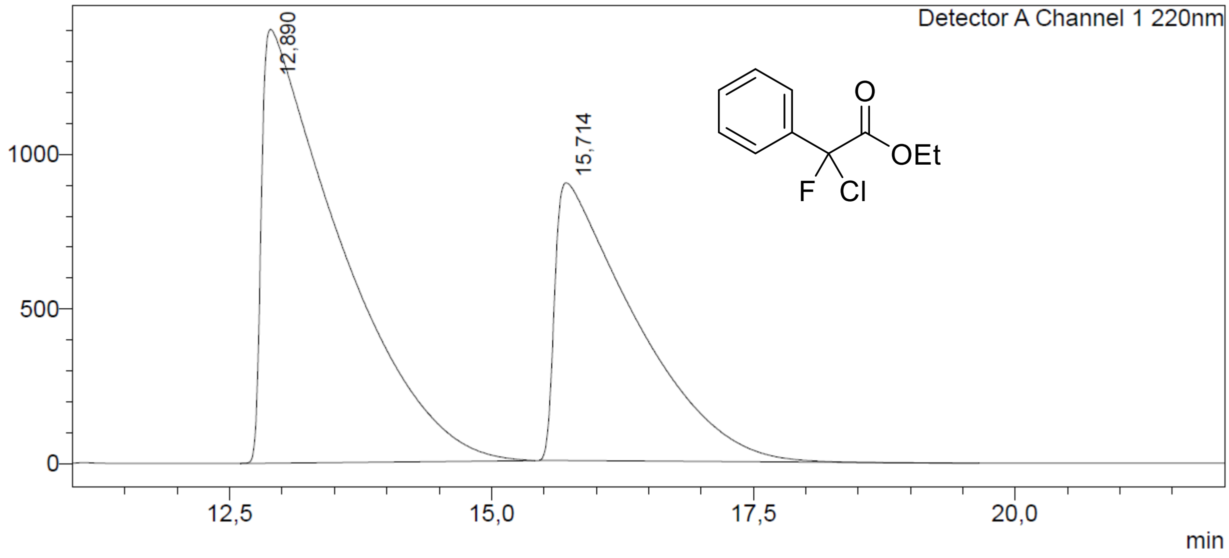

Peak#	Ret. Time	Area	Height	Conc.	Unit	Mark	Name
1	12,890	73644057	1402976	60,500		M	
2	15,714	48081420	898357	39,500		M	
Total		121725477	2301333				

Chromatogram of racemic molecule **3'b**:

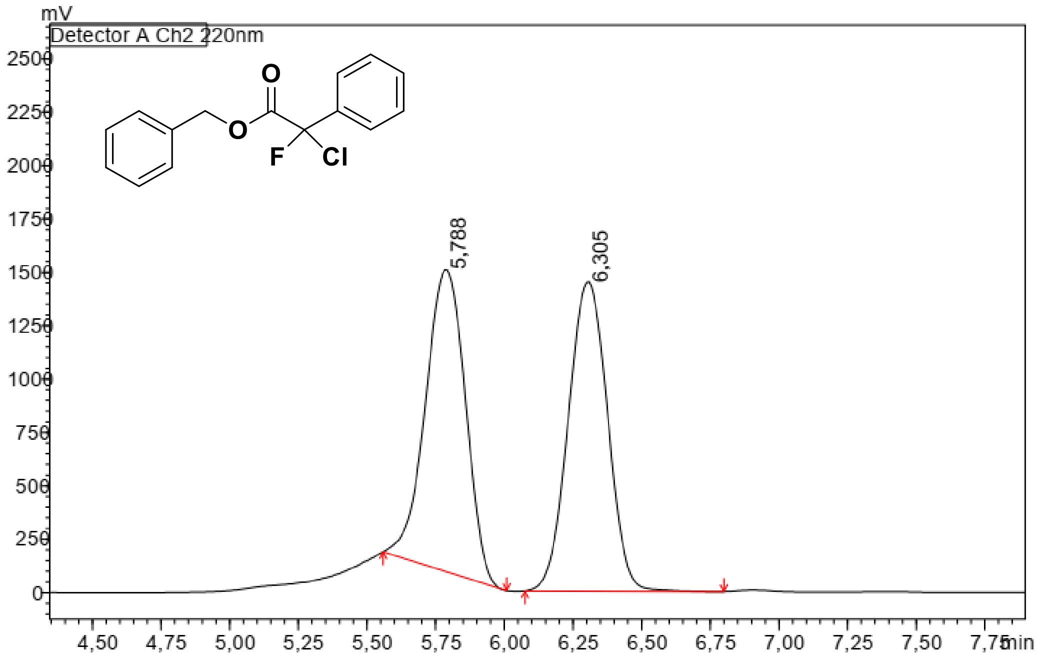

Peak Table

Detector A Channel 2 220nm

Peak#	Ret. Time	Area	Height	Conc.	Height%	Area%
1	5,788	14268248	1414100	49,792	49,411	49,792
2	6,305	14387455	1447819	50,208	50,589	50,208
Total		28655702	2861918		100,000	100,000

Chromatogram of enantioenriched molecule **3’b**:

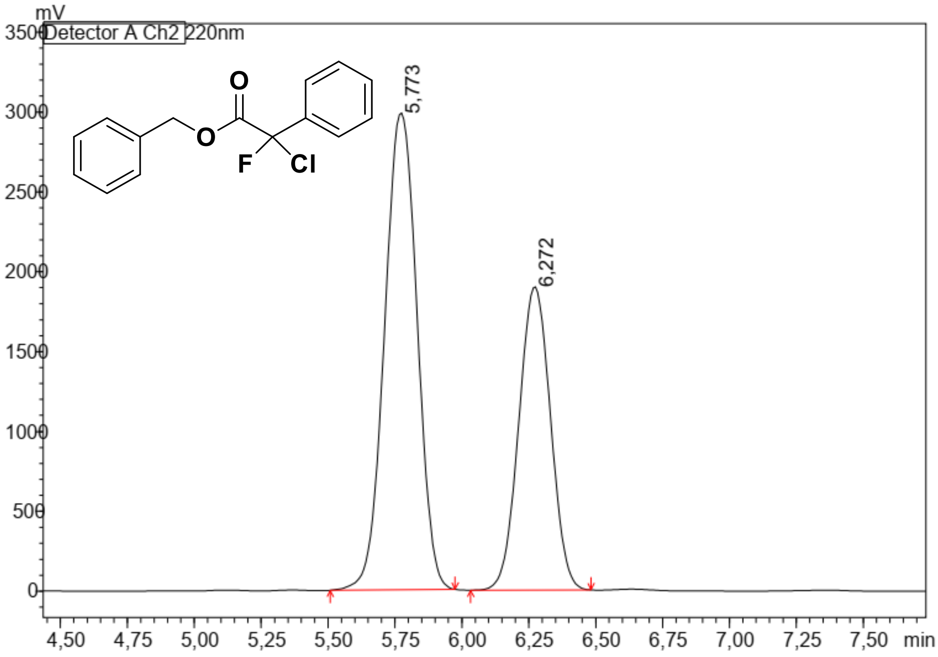

Peak Table

Detector A Channel 2 220nm

Peak#	Ret. Time	Area	Height	Height%	Area%
1	5,773	25244449	2984774	61,142	61,708
2	6,272	15665346	1896919	38,858	38,292
Total		40909795	4881693	100,000	100,000

Chromatogram of racemic molecule **3’c**:

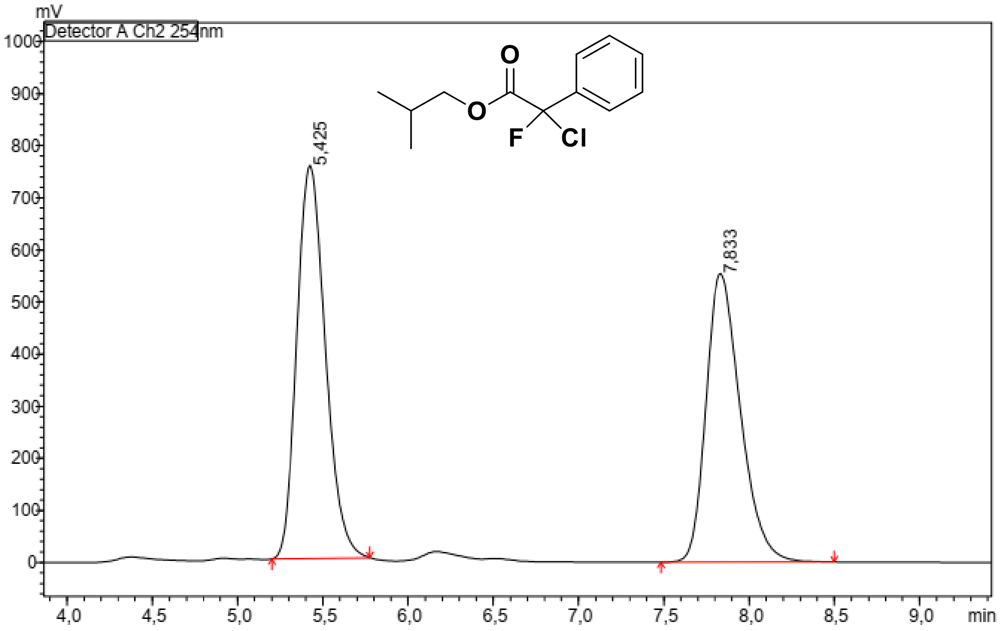

Peak Table

Detector A Channel 2 254nm

Peak#	Ret. Time	Area	Height	Height%	Area%
1	5,425	8726252	753686	57,639	52,765
2	7,833	7811560	553909	42,361	47,235
Total		16537812	1307595	100,000	100,000

Chromatogram of enantioenriched molecule **3’c**:

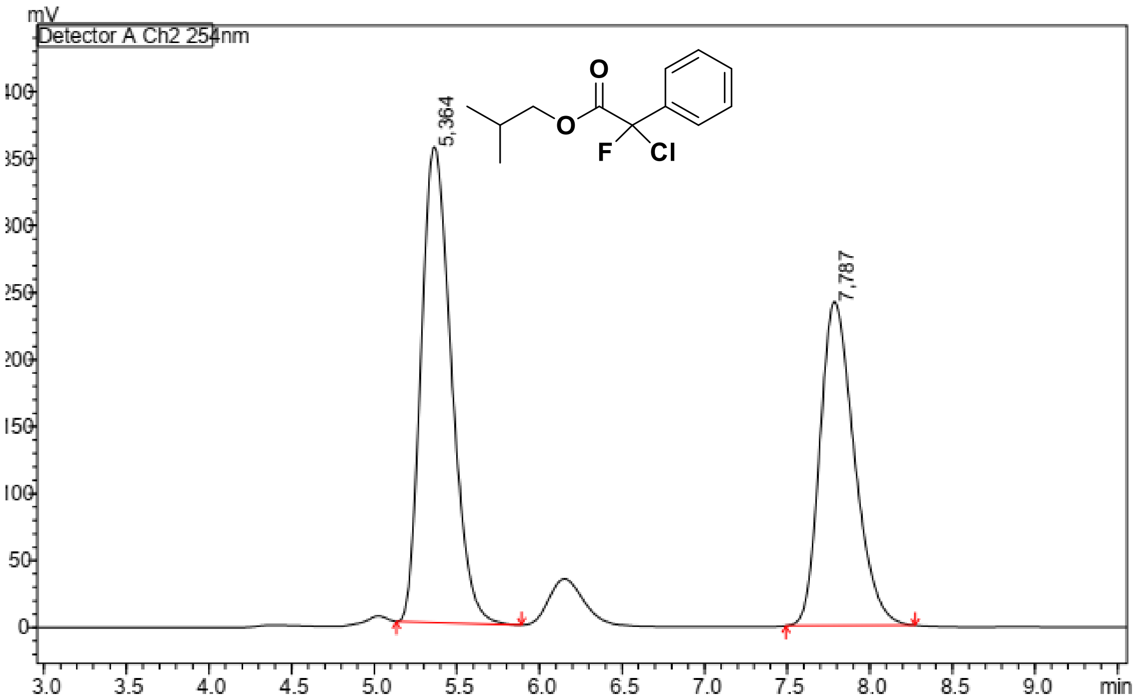

Peak Table

Detector A Channel 2 254nm

Peak#	Ret. Time	Area	Conc.	Height	Height%	Area%
1	5,364	4529008	56,352	355171	59,491	56,352
2	7,787	3507950	43,648	241848	40,509	43,648
Total		8036958		597019	100,000	100,000

Chromatogram of racemic molecule **3’d**:

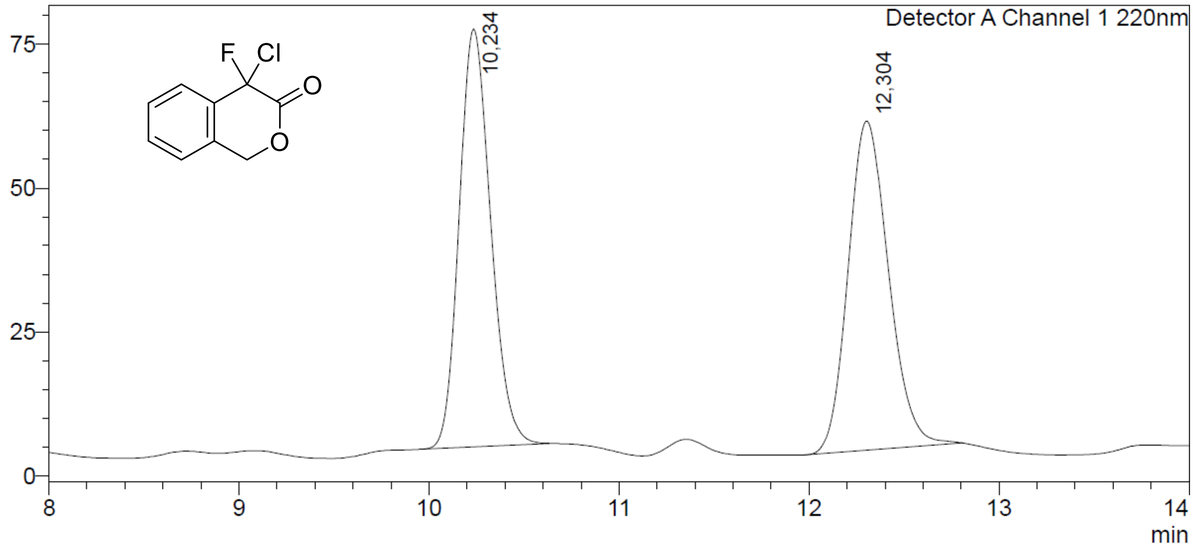

Peak#	Ret. Time	Area	Height	Conc.	Unit	Mark	Name
1	10,234	837142	72663	49,670		M	
2	12,304	848271	57239	50,330		M	
Total		1685413	129903				

Chromatogram of enantioenriched molecule **3’d**:

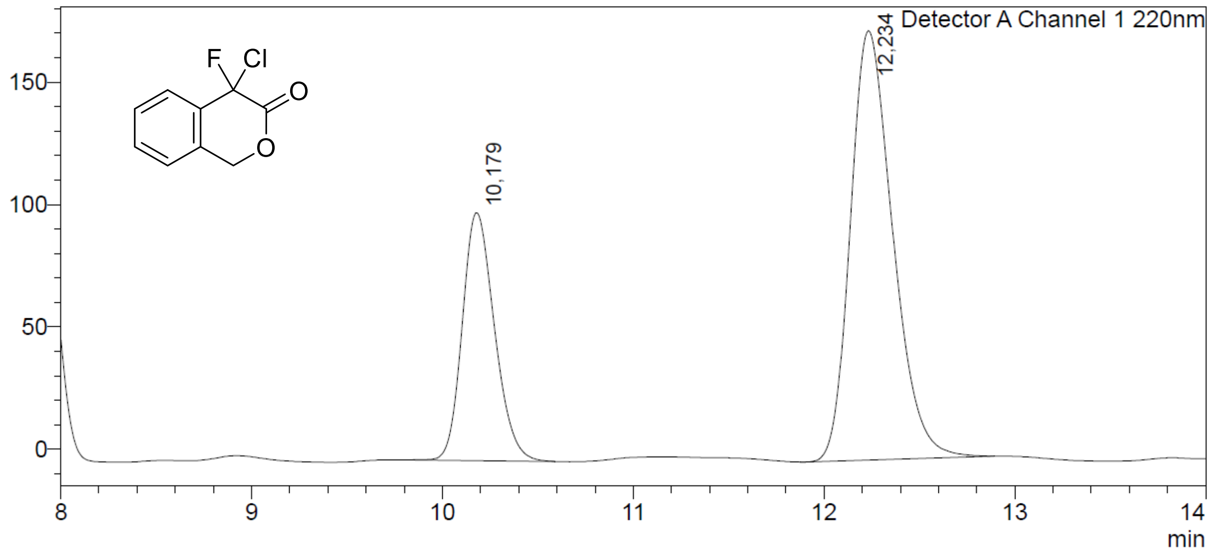

Peak#	Ret. Time	Area	Height	Conc.	Unit	Mark	Name
1	10,179	1185179	101161	30,444		M	
2	12,234	2707844	175401	69,556		M	
Total		3893023	276562				

Chromatogram of racemic molecule **3’e**:

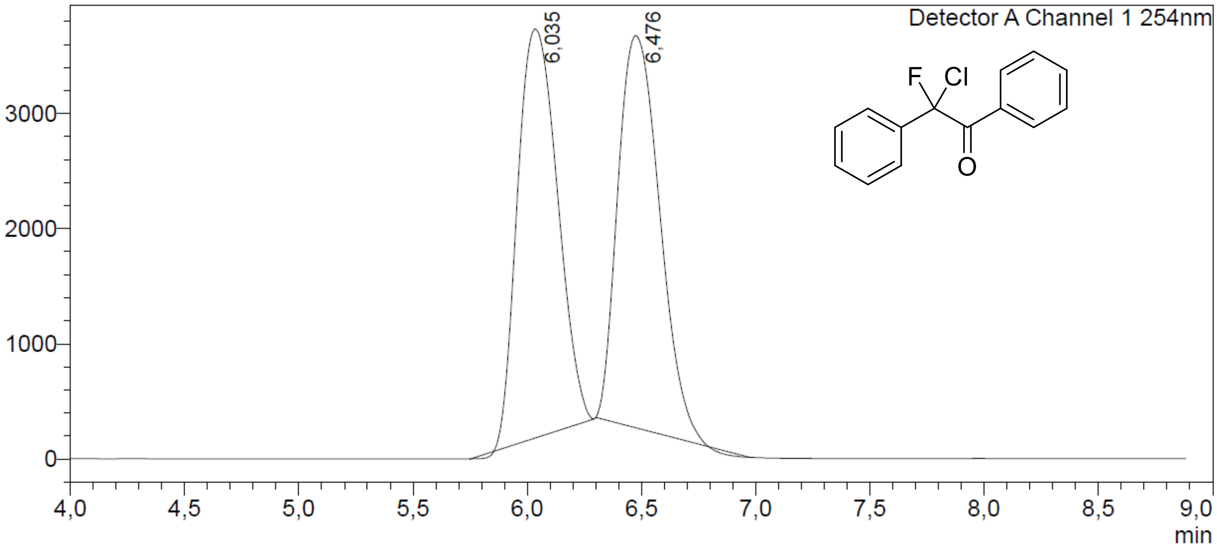

Peak#	Ret. Time	Area	Height	Conc.	Unit	Mark	Name
1	6,035	43408049	3553522	50,107		M	
2	6,476	43222888	3410250	49,893		M	
Total		86630937	6963771				

Chromatogram of enantioenriched molecule **3'e**:

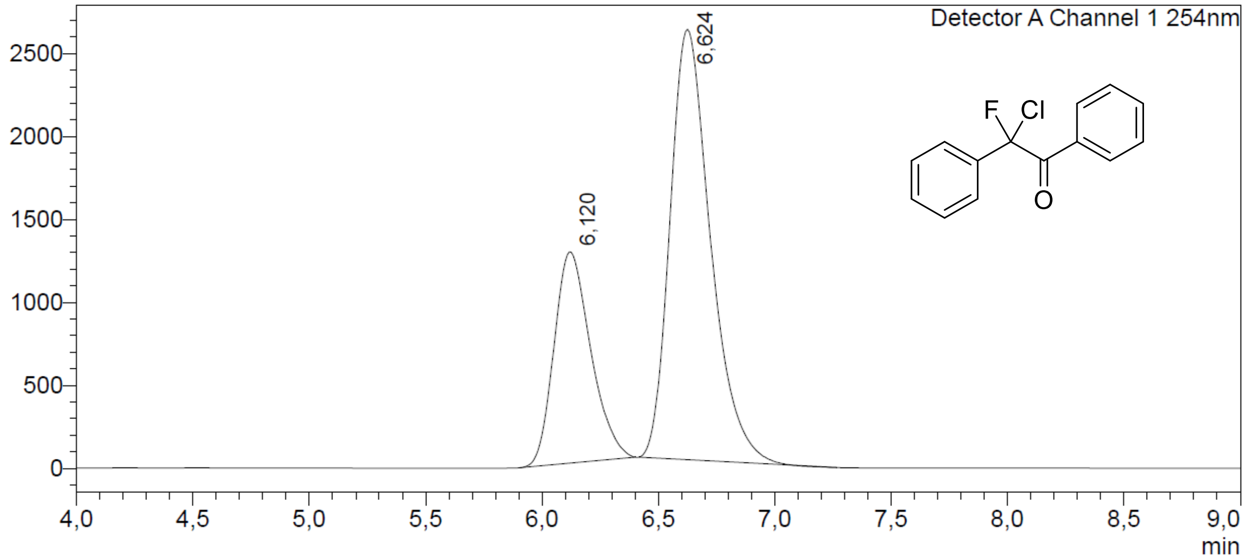

Peak#	Ret. Time	Area	Height	Conc.	Unit	Mark	Name
1	6,120	13947839	1273384	30,739		M	
2	6,624	31426606	2593271	69,261		M	
Total		45374445	3866655				

Chromatogram of racemic molecule **3'f**:

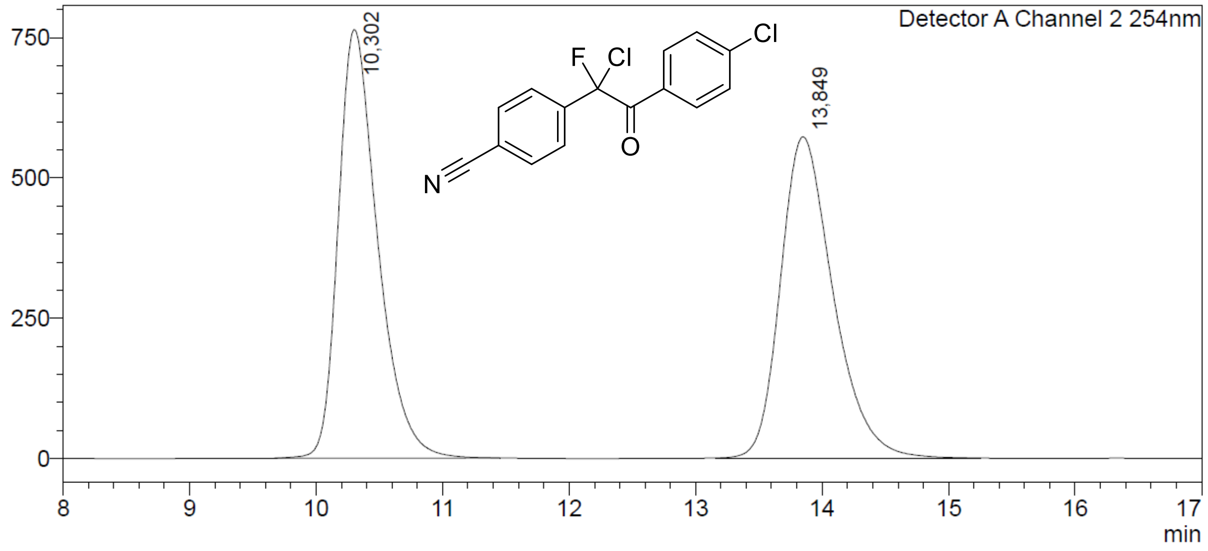

Detector A Channel 2 254nm

Peak#	Ret. Time	Area	Height	Conc.	Unit	Mark	Name
1	10,302	16597729	764020	50,027		M	
2	13,849	16579716	572503	49,973		M	
Total		33177444	1336523				

Chromatogram of enantioenriched molecule **3’f**:

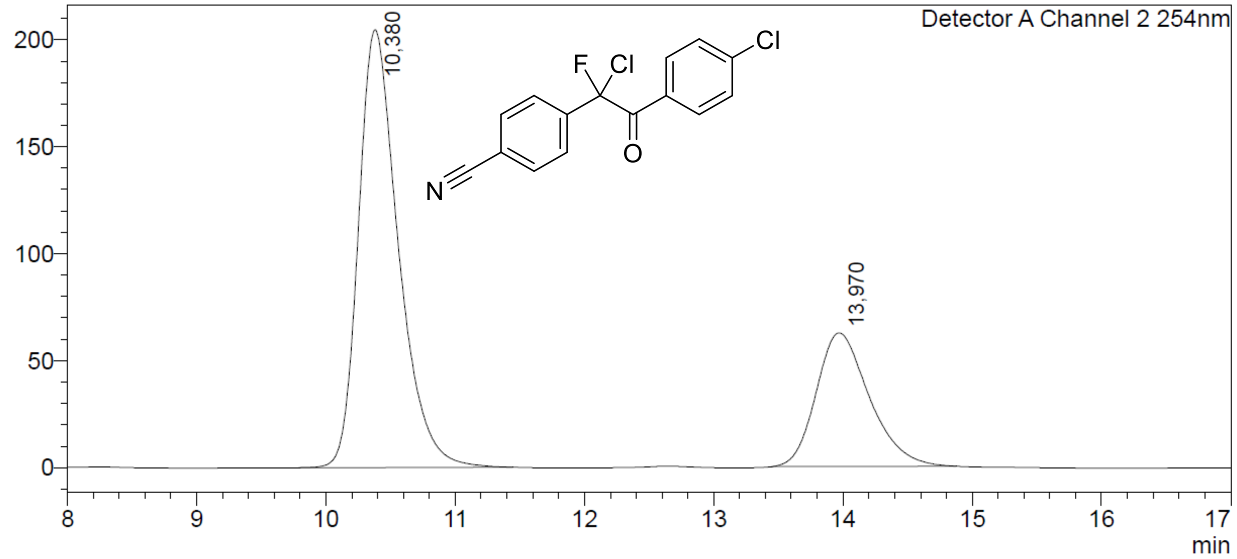

Detector A Channel 2 254nm

Peak#	Ret. Time	Area	Height	Conc.	Unit	Mark	Name
1	10,380	4398788	204704	71,101		M	
2	13,970	1787890	62494	28,899		M	
Total		6186678	267197				

Chromatogram of racemic molecule **3’g**:

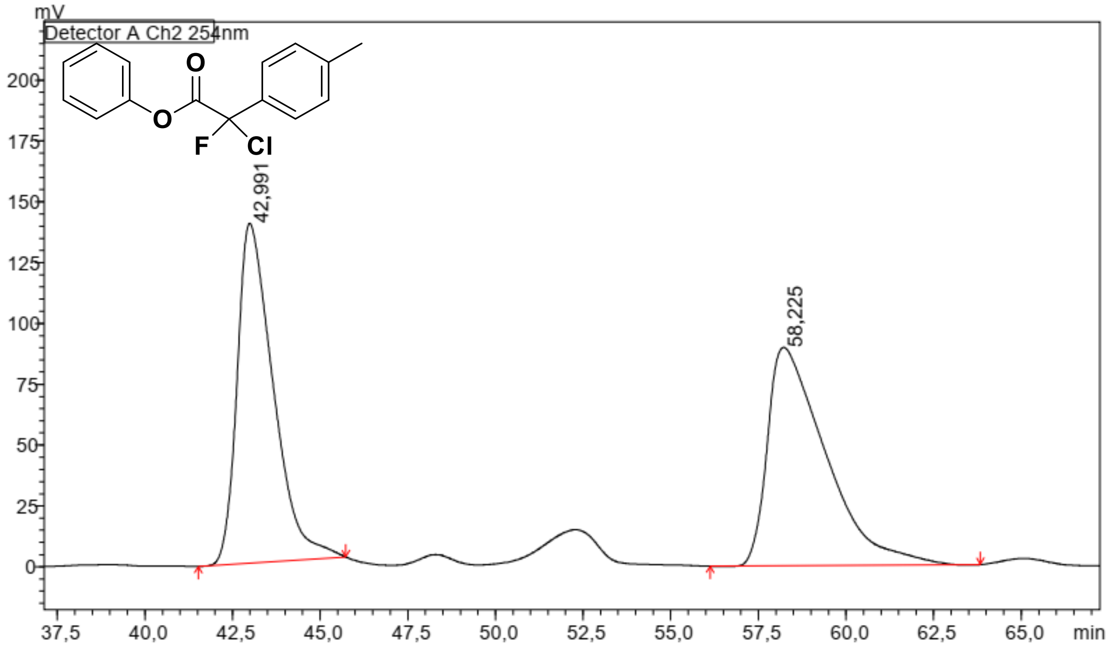

Peak Table

Detector A Channel 2 254nm

Peak#	Ret. Time	Area	Height	Height%	Area%
1	42,991	9968329	139639	60,882	48,990
2	58,225	10379149	89721	39,118	51,010
Total		20347478	229360	100,000	100,000

Chromatogram of enantioenriched molecule **3’g**:

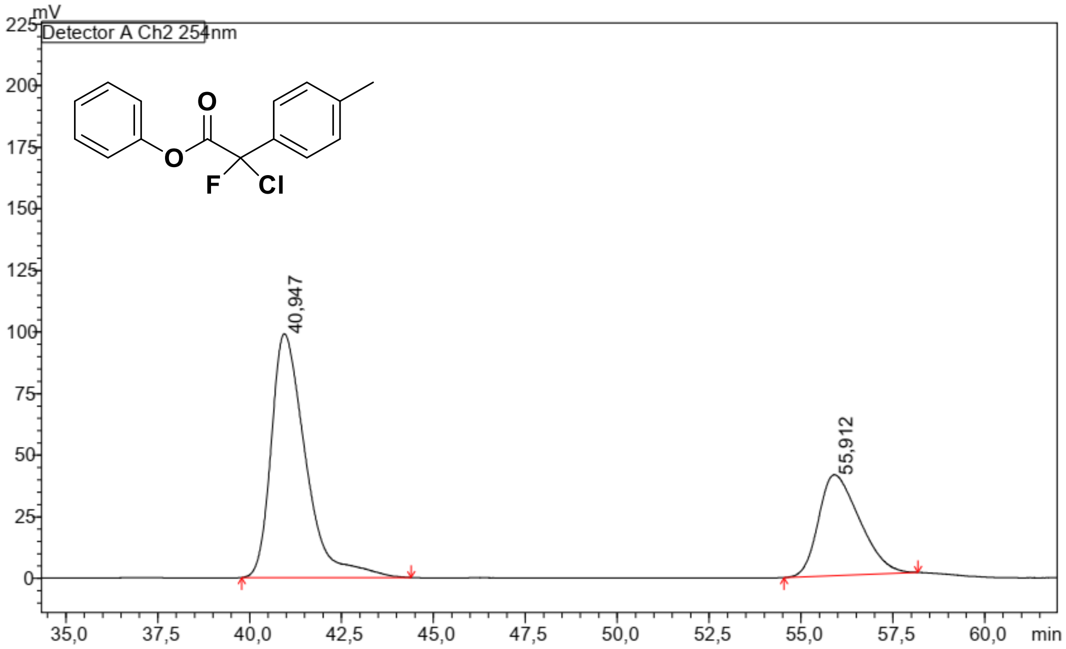

Peak Table

Detector A Channel 2 254nm

Peak#	Ret. Time	Area	Height	Height%	Area%
1	40,947	6447286	99035	70,735	66,944
2	55,912	3183611	40974	29,265	33,056
Total		9630896	140009	100,000	100,000

Chromatogram of racemic molecule **3’h**:

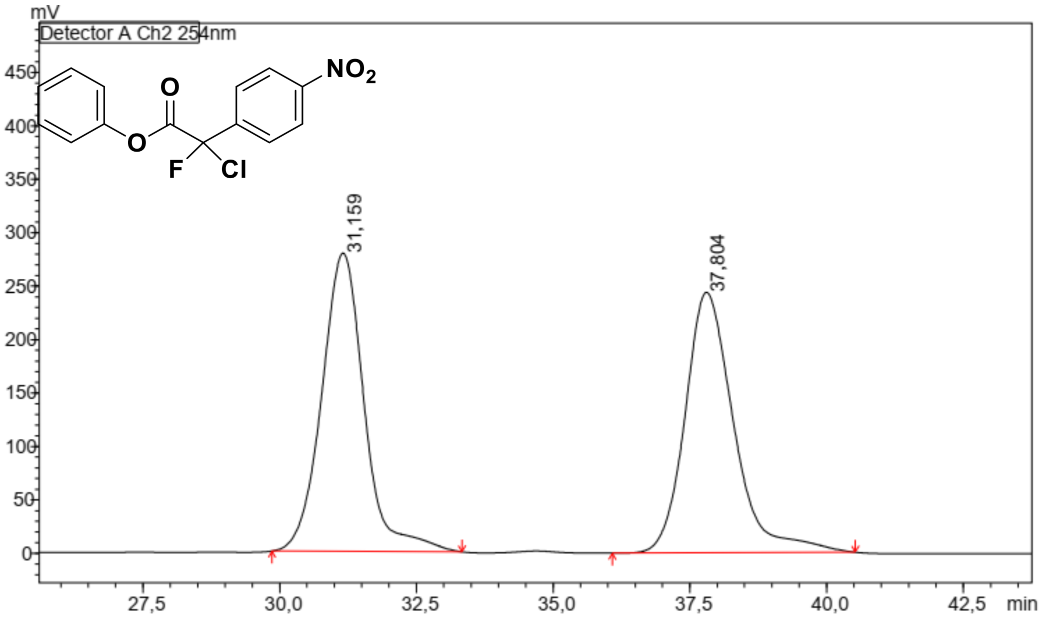

Peak Table

Detector A Channel 2 254nm

Peak#	Ret. Time	Area	Height	Height%	Area%
1	31,159	15094206	279152	53,386	49,967
2	37,804	15113904	243742	46,614	50,033
Total		30208110	522893	100,000	100,000

Chromatogram of enantioenriched molecule **3'h**:

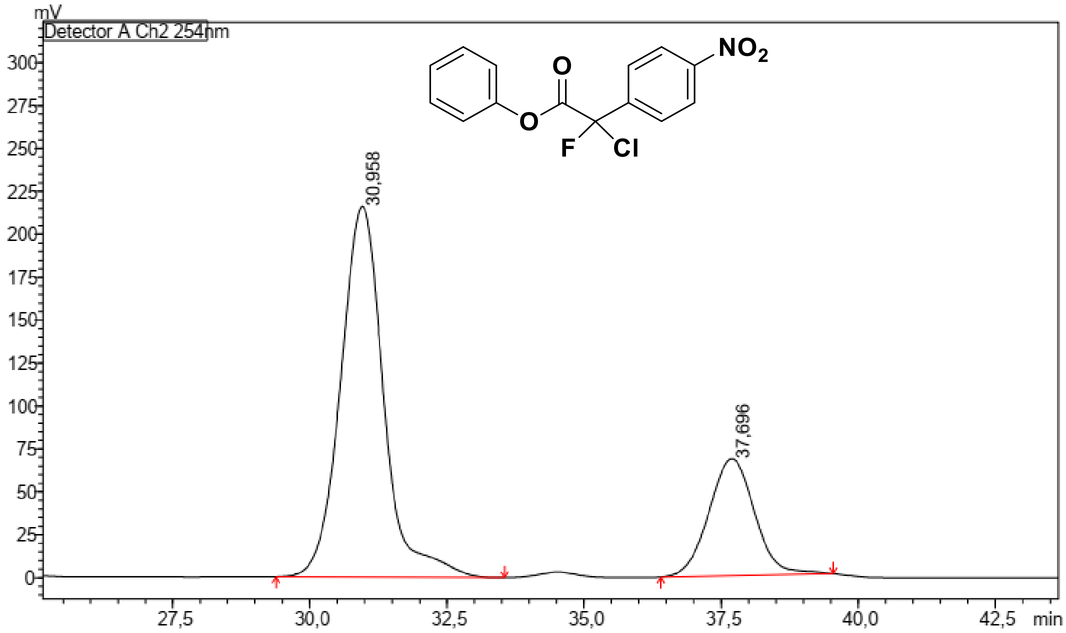

Peak Table

Detector A Channel 2 254nm

Peak#	Ret. Time	Area	Height	Height%	Area%
1	30,958	11790435	215950	76,016	74,874
2	37,696	3956625	68134	23,984	25,126
Total		15747060	284083	100,000	100,000

8. ^{1}H, ^{13}C{H} AND ^{19}F NMR SPECTRA

^{1}H NMR *(400 MHz, $CDCl_3$)* of Molecule **A1**:

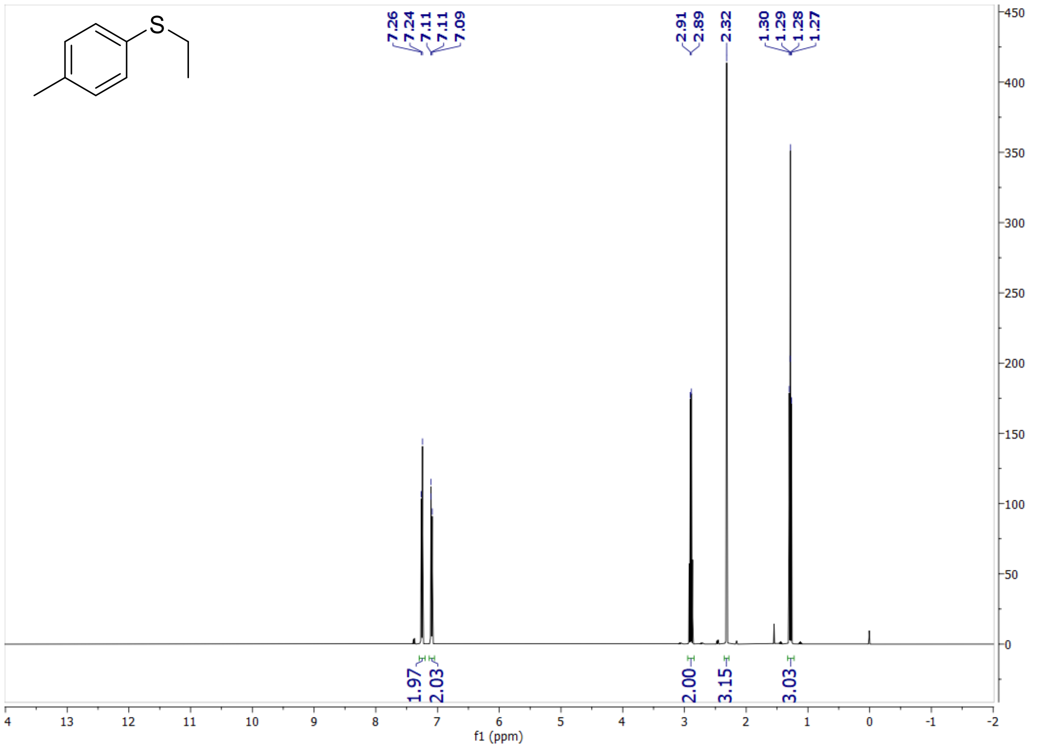

^{13}C{H} NMR *(100 MHz, $CDCl_3$)* of Molecule **A1**:

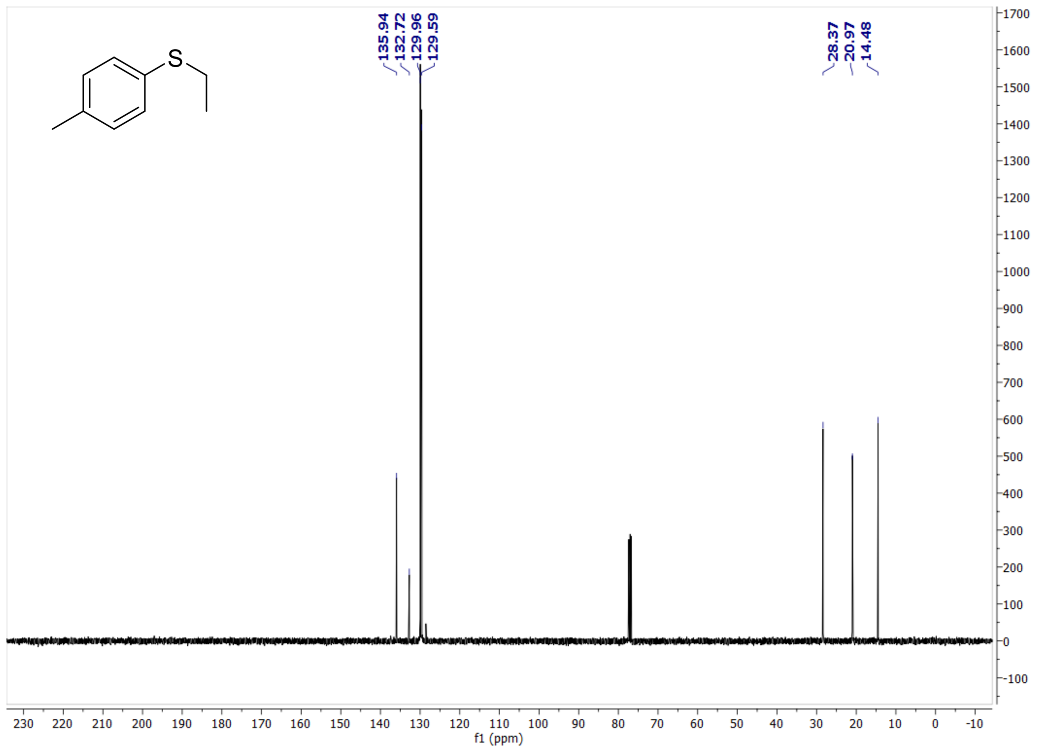

^{1}H NMR *(500 MHz, $CDCl_3$)* of Molecule **A2**:

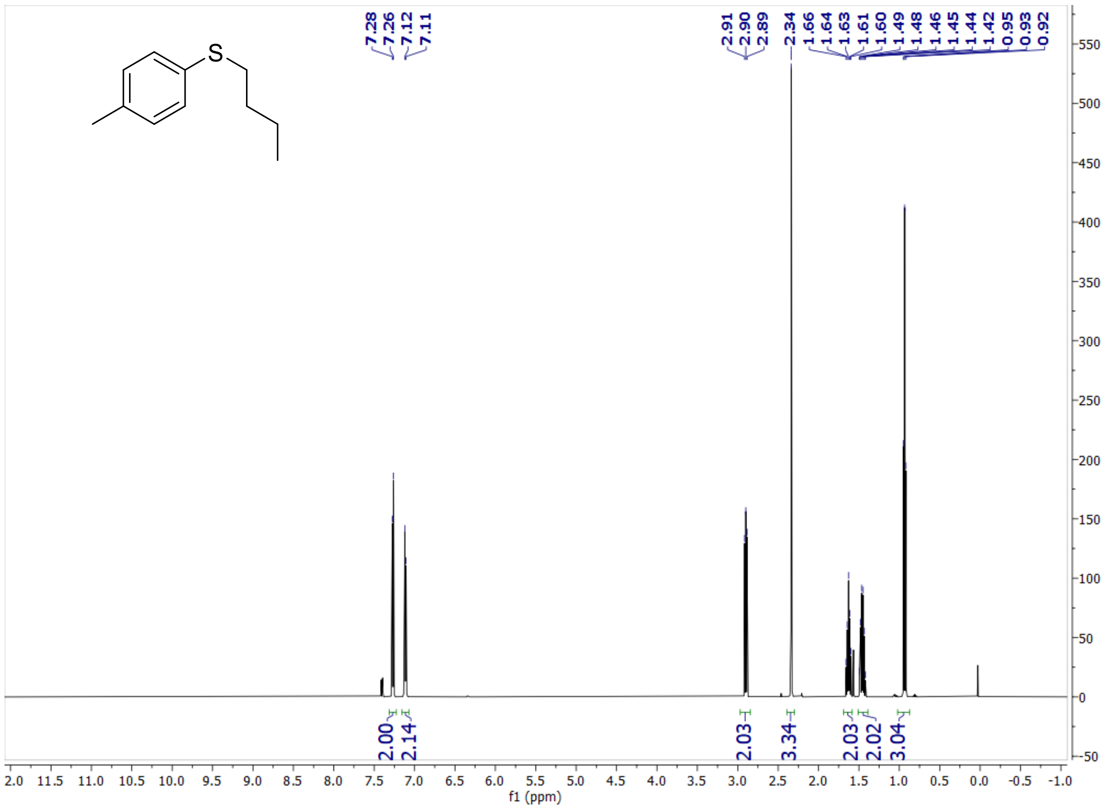

^{13}C{H} NMR *(125 MHz, $CDCl_3$)* of Molecule **A2**:

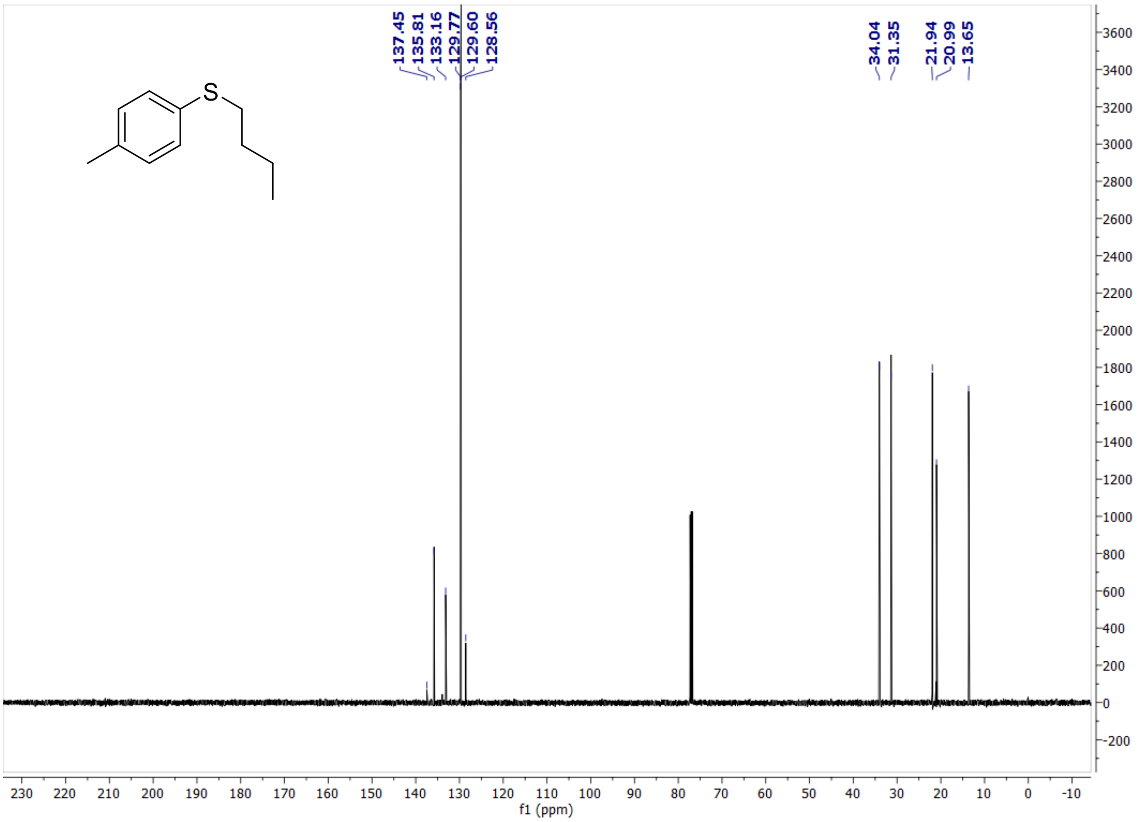

^{1}H NMR *(500 MHz, $CDCl_3$)* of Molecule **B1**:

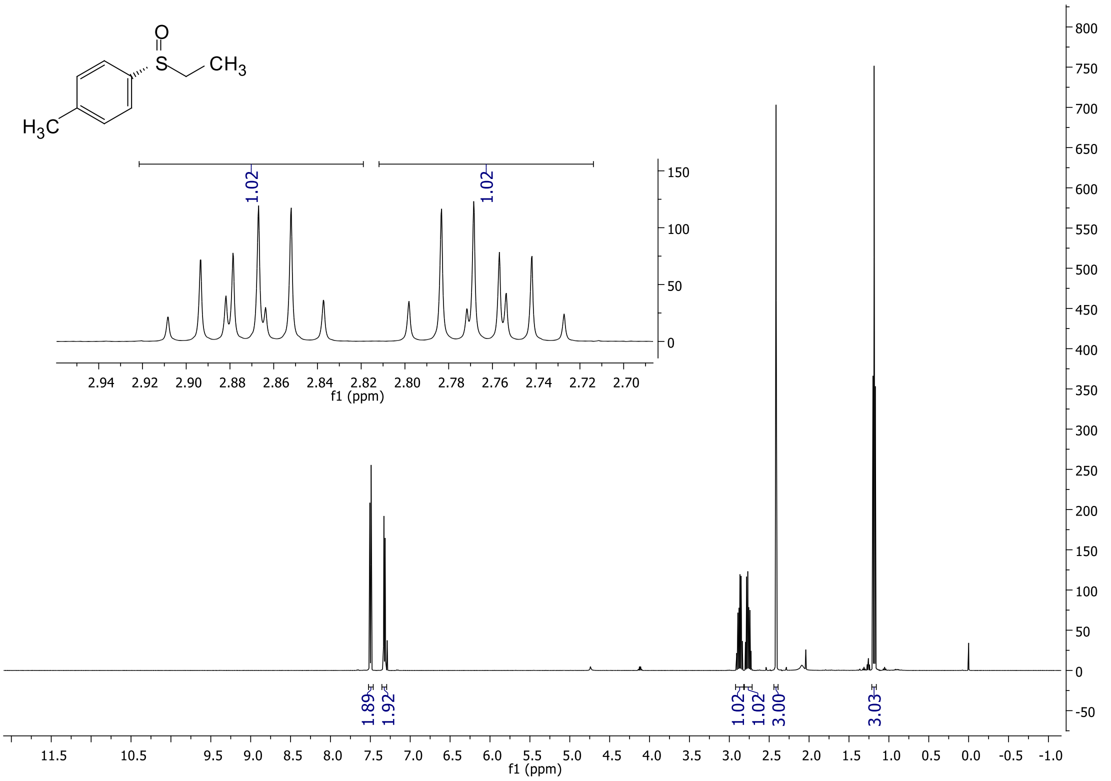

^{13}C{H} NMR *(125 MHz, $CDCl_3$)* of Molecule **B1**:

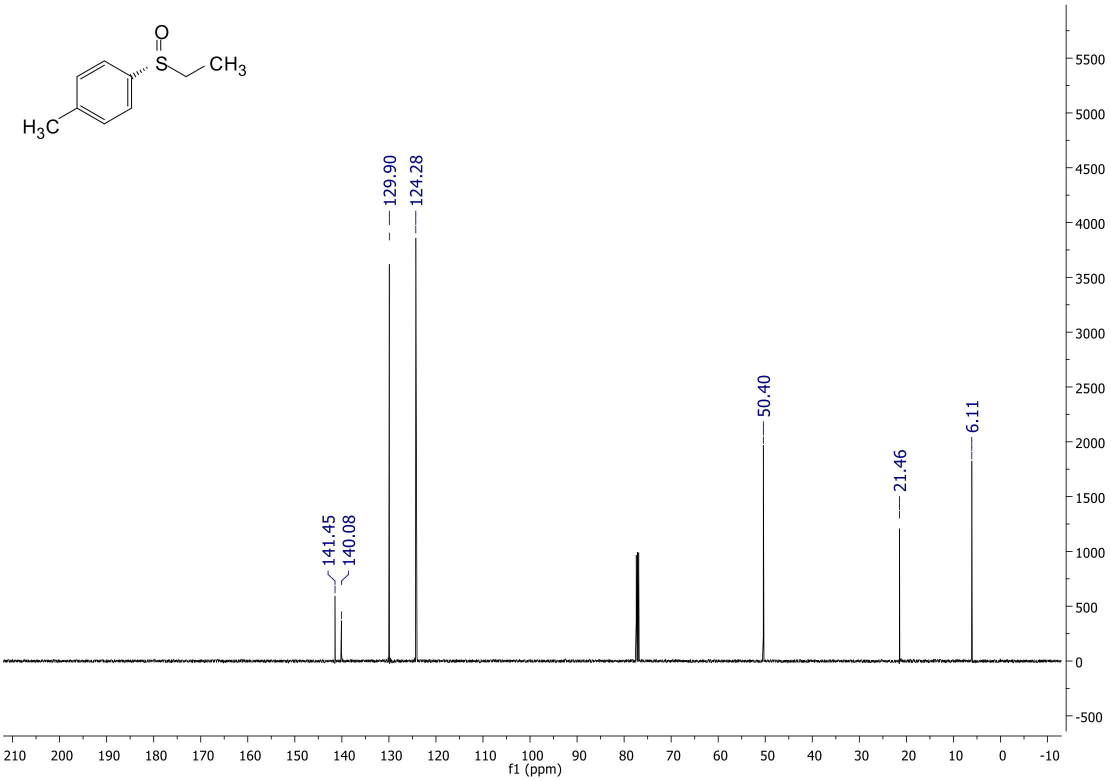

^{1}H NMR *(500 MHz, CDCl3)* of Molecule **B2**:

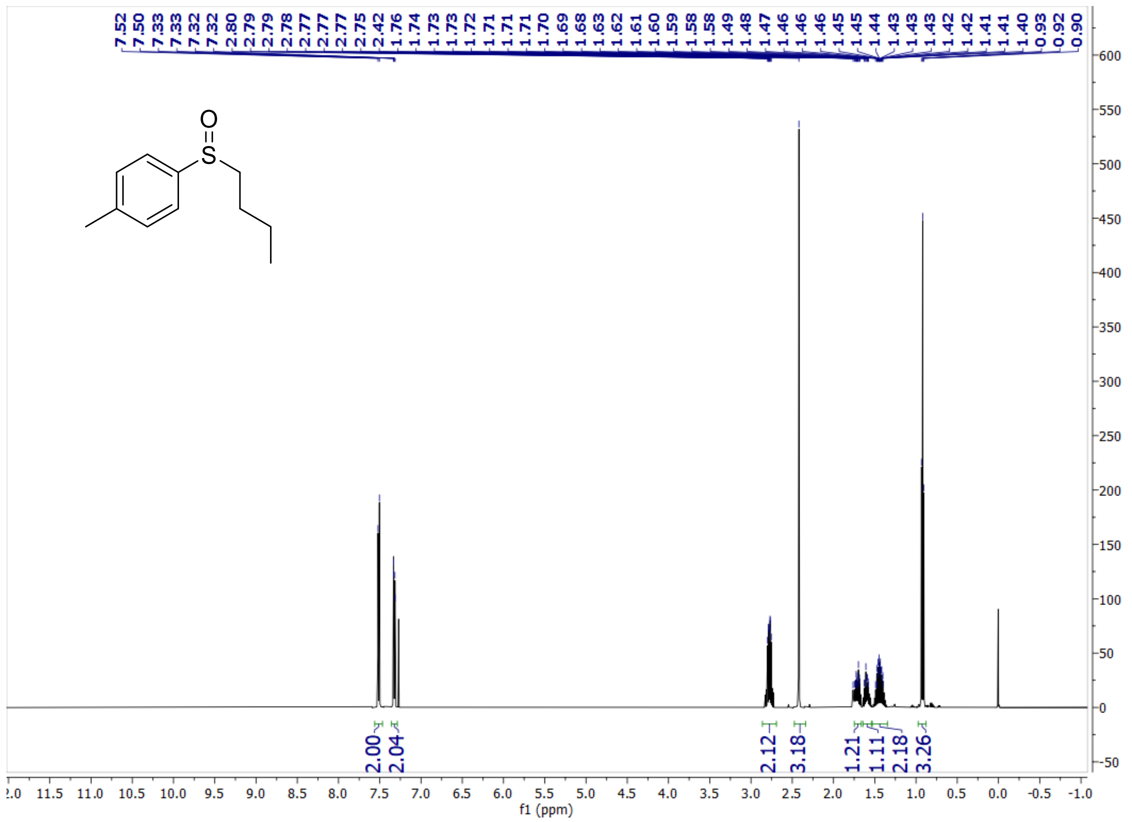

^{13}C{H} NMR *(125 MHz, $CDCl_3$)* of Molecule **B2**:

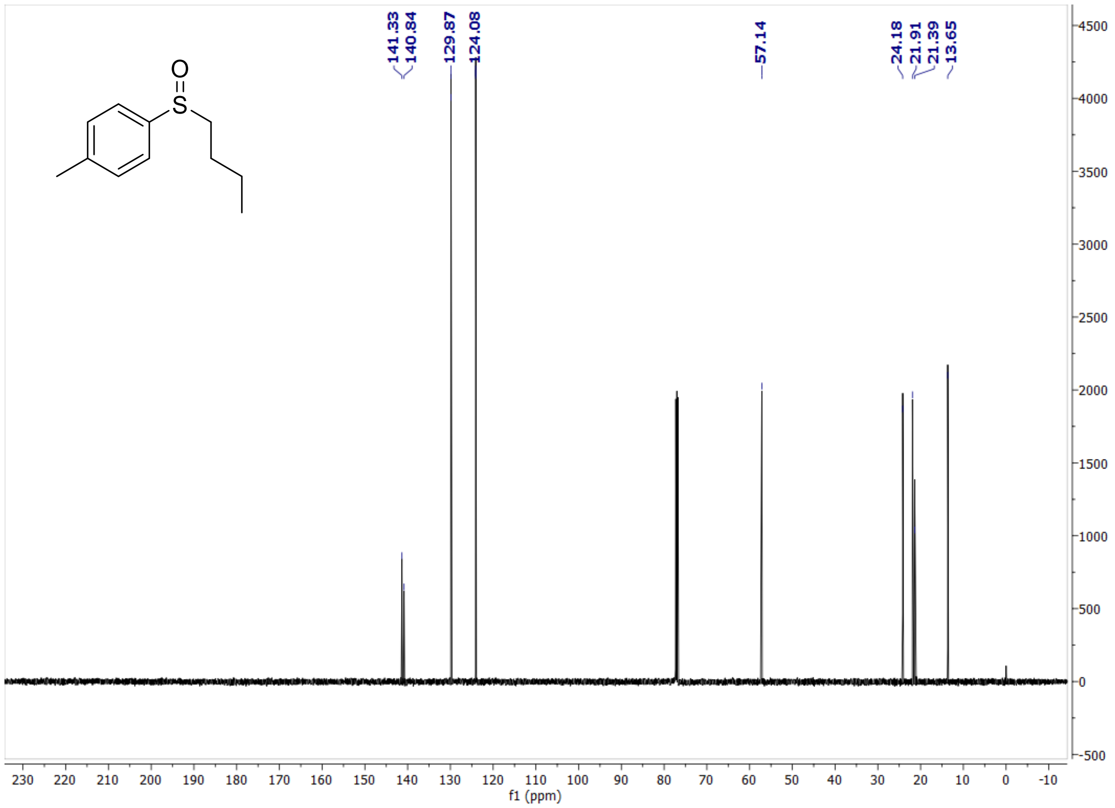

^{1}H NMR *(500 MHz, $CDCl_3$)* of Molecule **C1**:

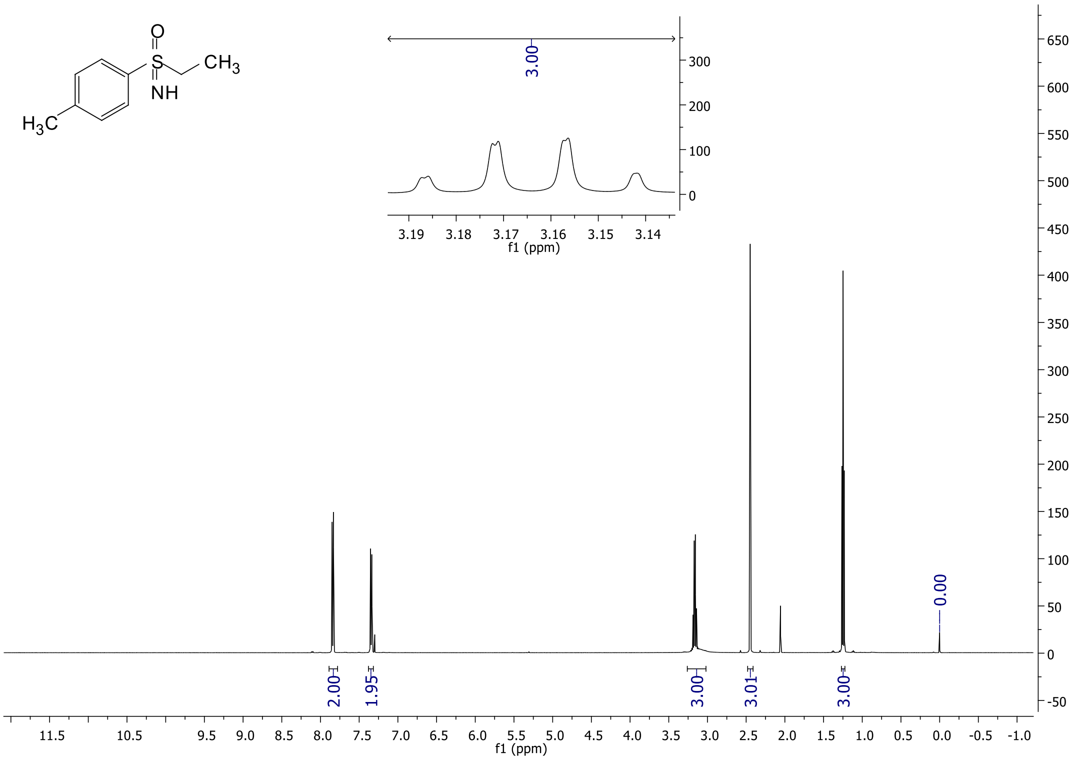

^{13}C{H} NMR *(125 MHz, $CDCl_3$)* of Molecule **C1**:

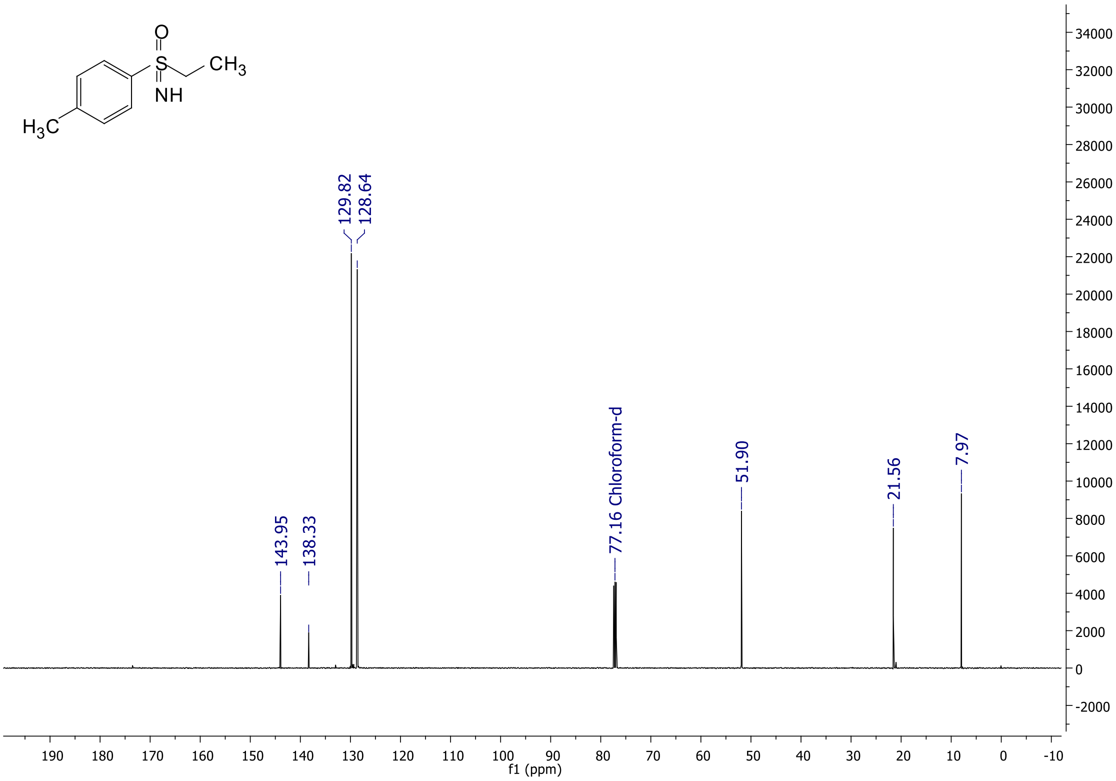

^{1}H NMR *(400 MHz, $CDCl_3$)* of Molecule **C2**:

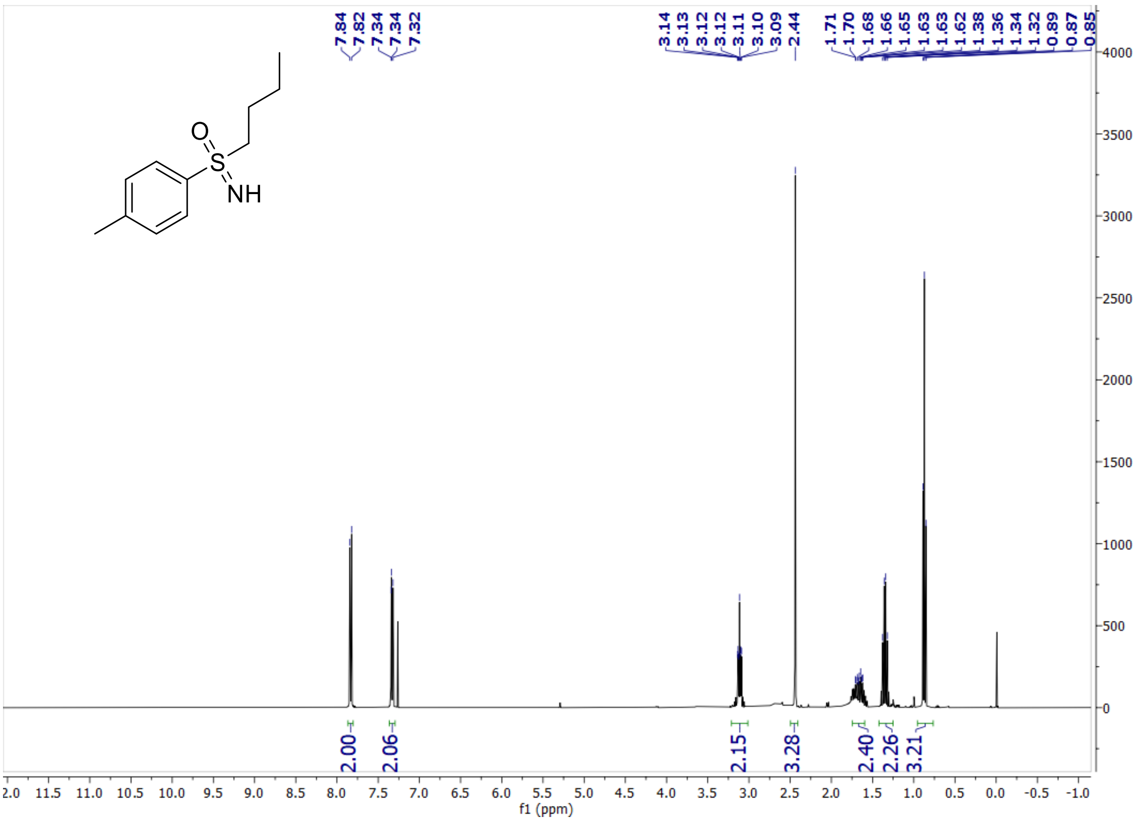

^{13}C{H} NMR *(100 MHz, $CDCl_3$)* of Molecule **C2**:

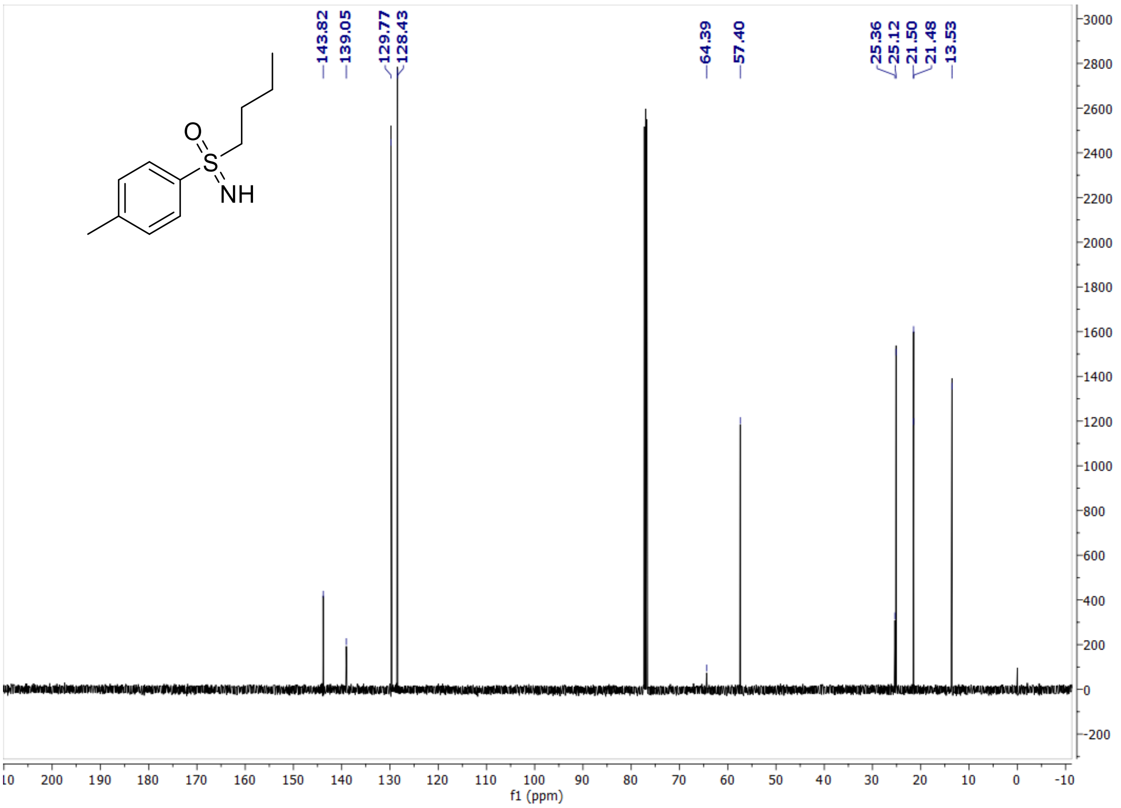

^{1}H NMR *(500 MHz, $CDCl_3$)* of Molecule **D1**:

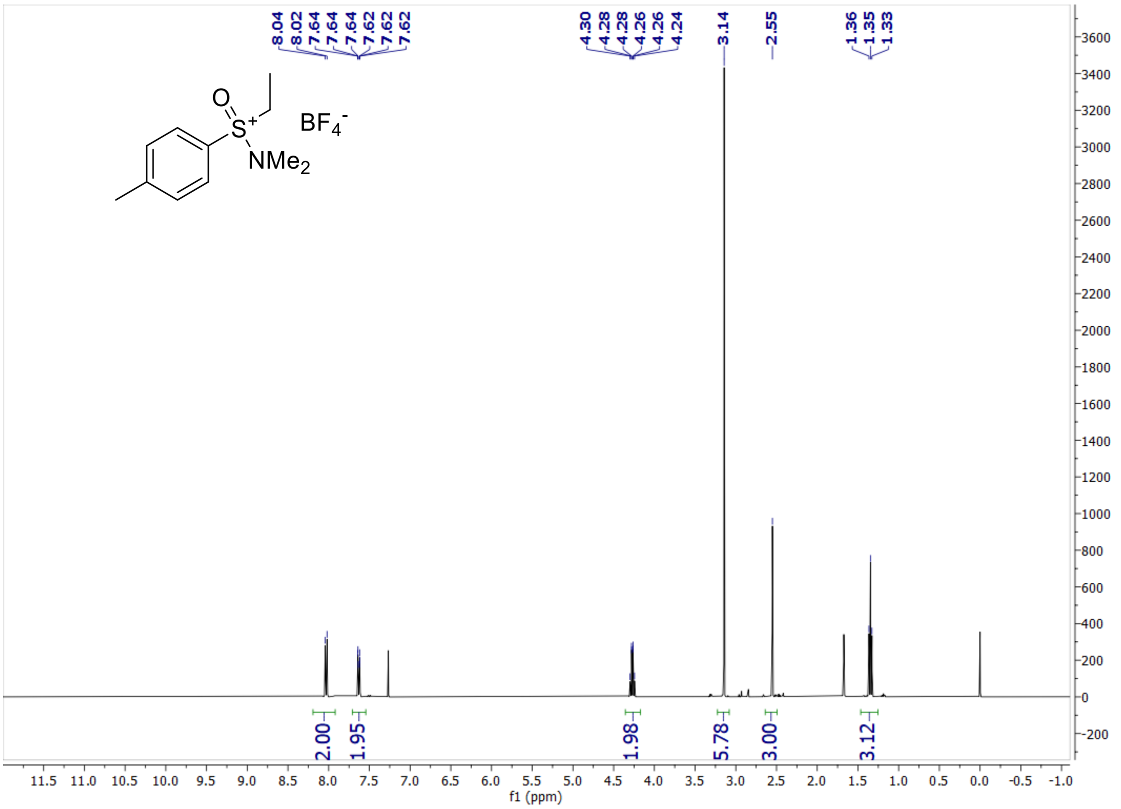

$^{13}C\{H\}$ NMR *(125 MHz, $CDCl_3$)* of Molecule **D1**:

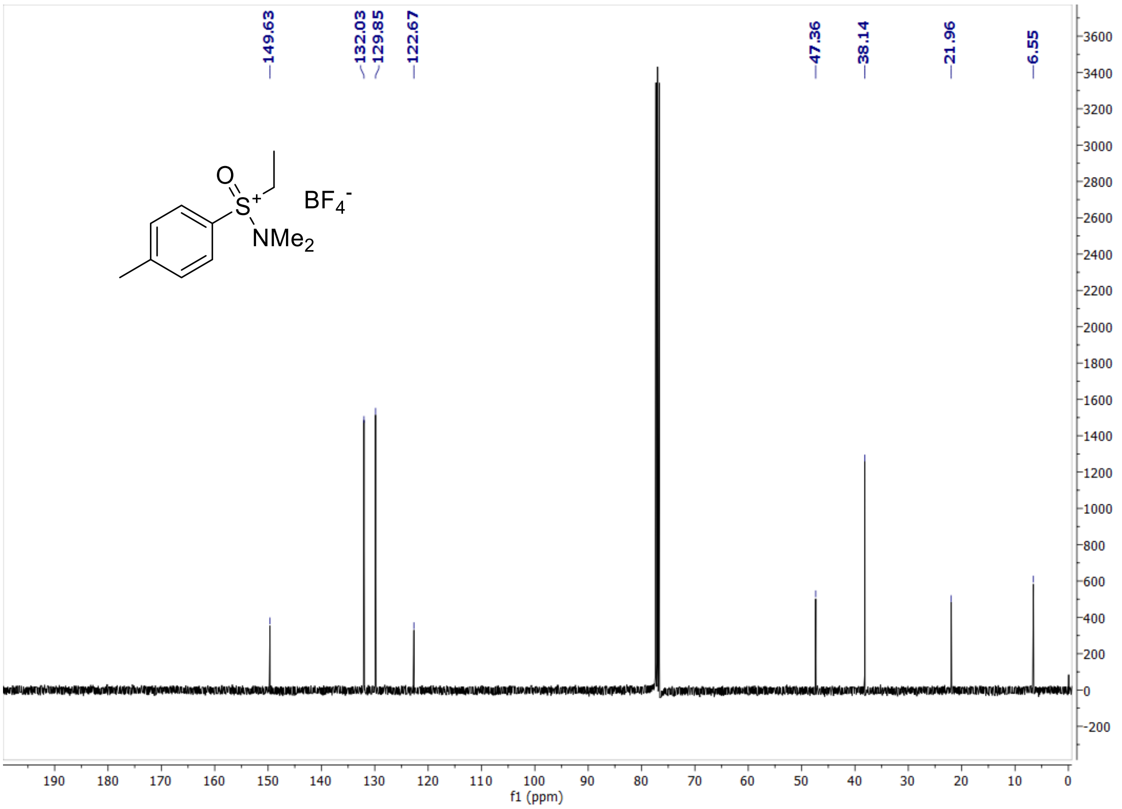

^{1}H NMR *(400 MHz, $CDCl_3$)* of Molecule **D2**:

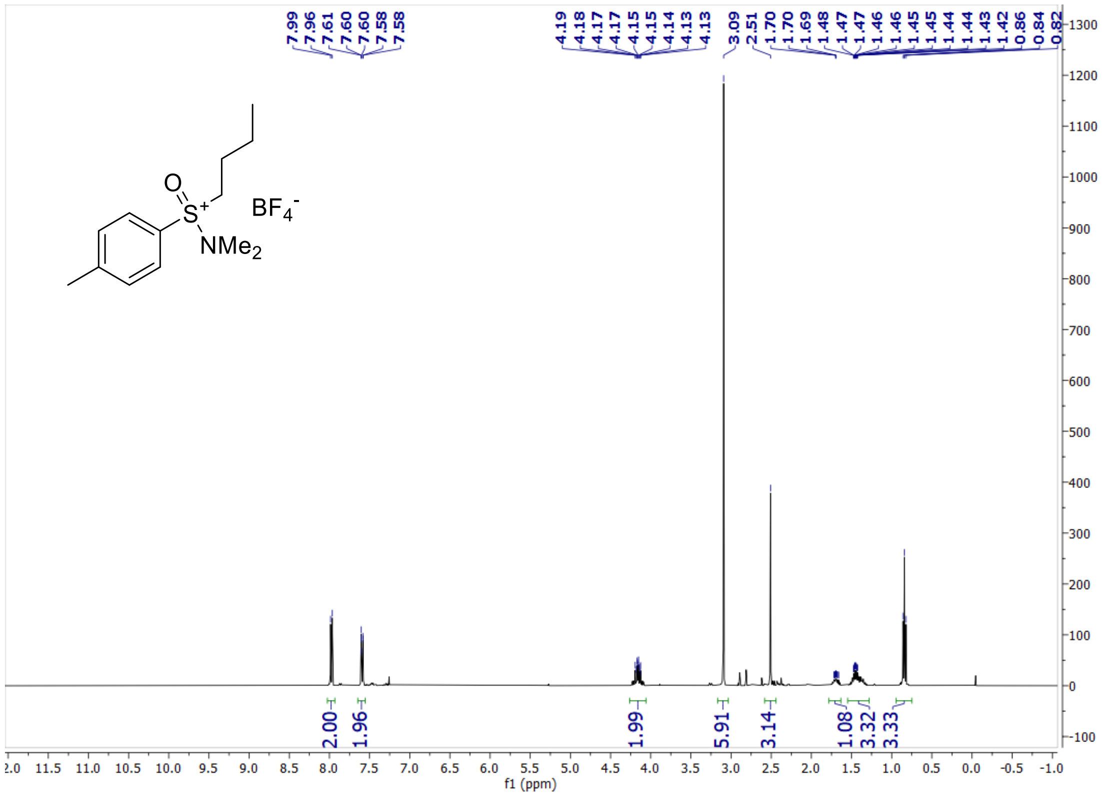

^{13}C{H} NMR *(100 MHz, $CDCl_3$)* of Molecule **D2**:

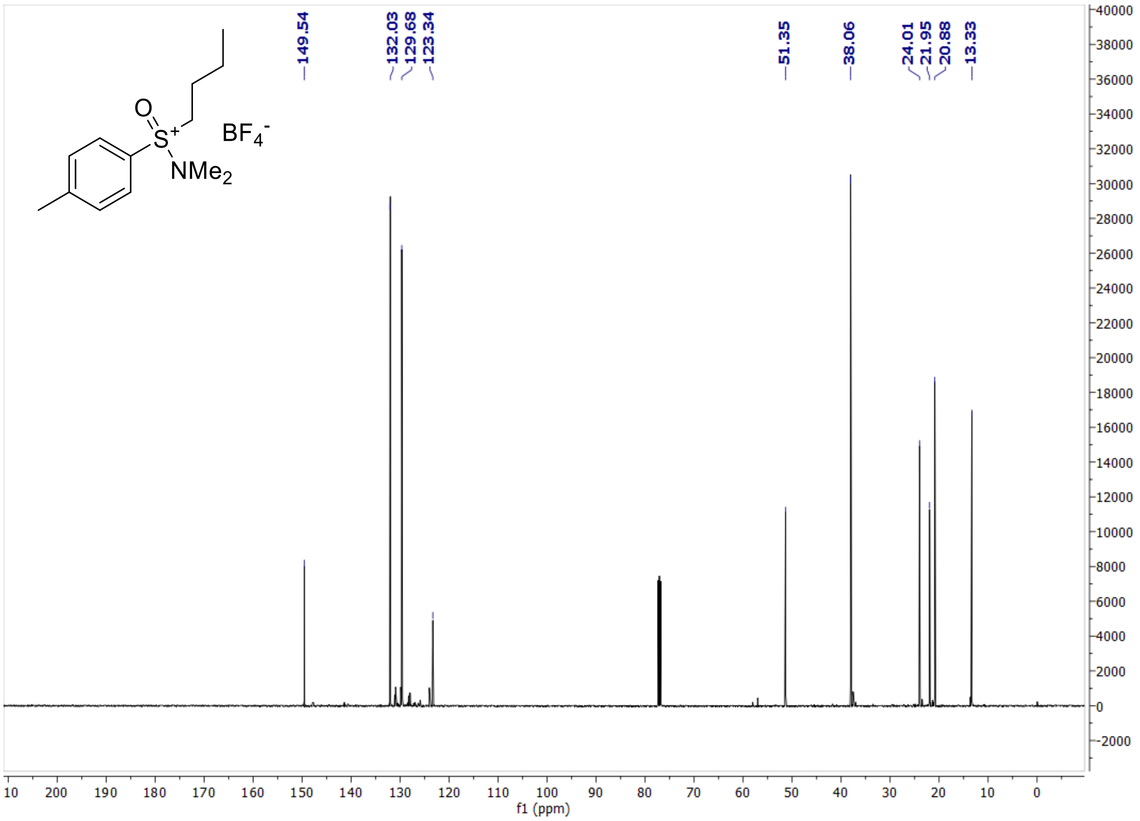

^{1}H NMR *(500 MHz, $CDCl_3$)* of Molecule **D3**:

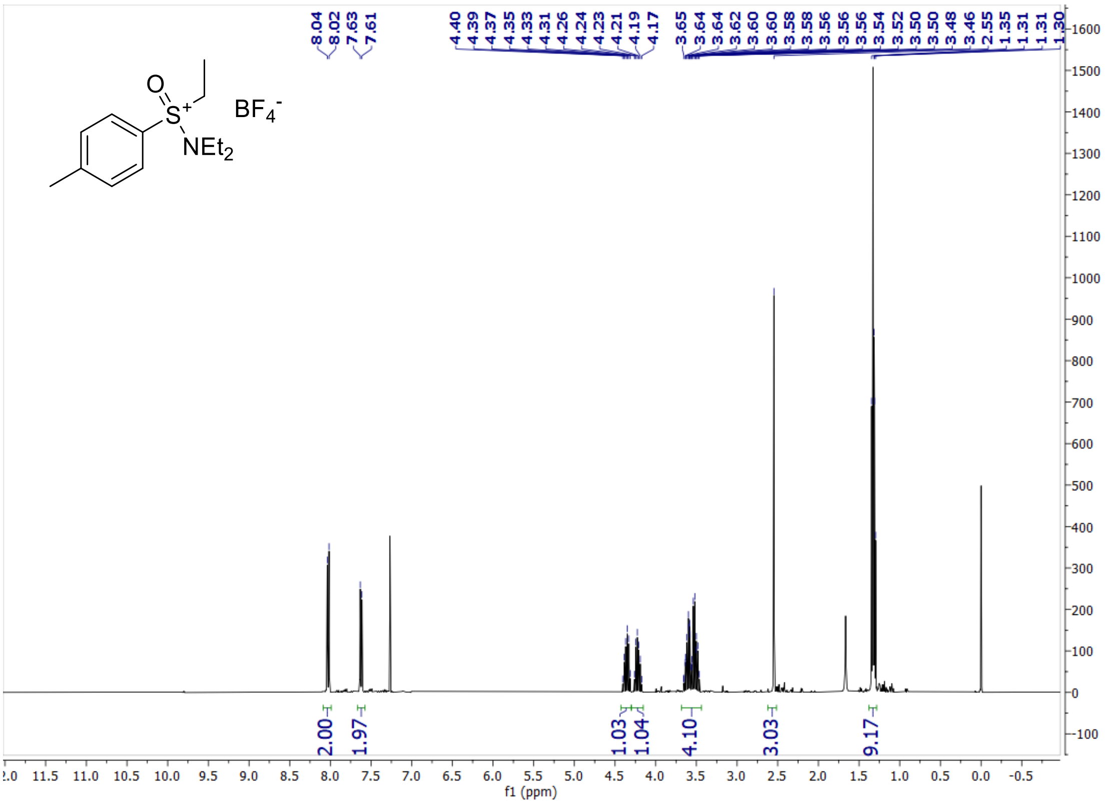

$^{13}C\{H\}$ NMR *(126 MHz, $CDCl_3$)* of Molecule **D3**:

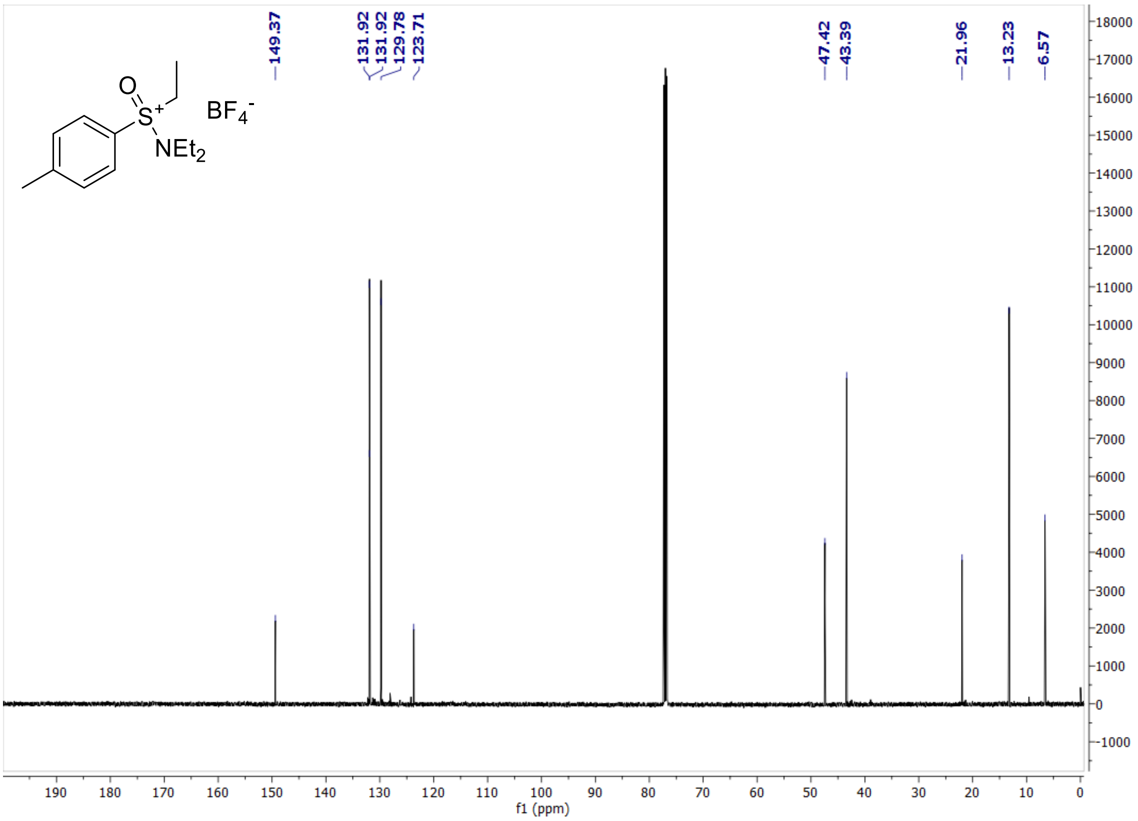

^{1}H NMR *(500 MHz, $CDCl_3$)* of Molecule **1a**:

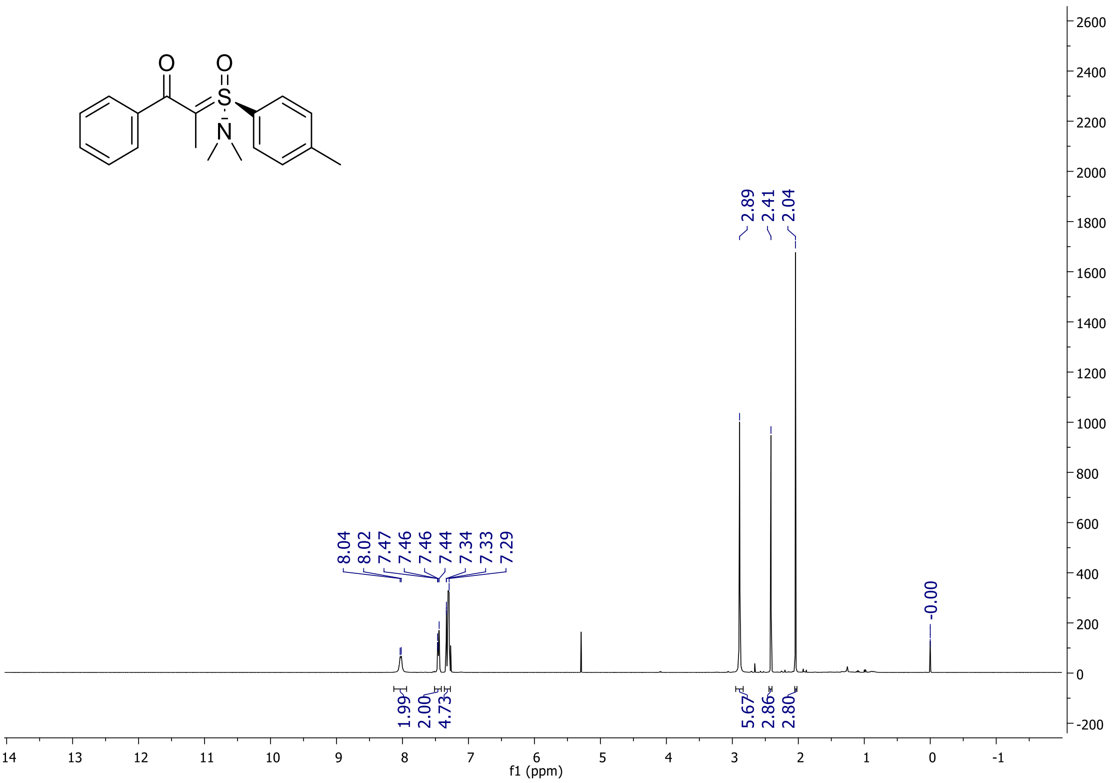

^{13}C{H} NMR *(125 MHz, $CDCl_3$)* of Molecule **1a**:

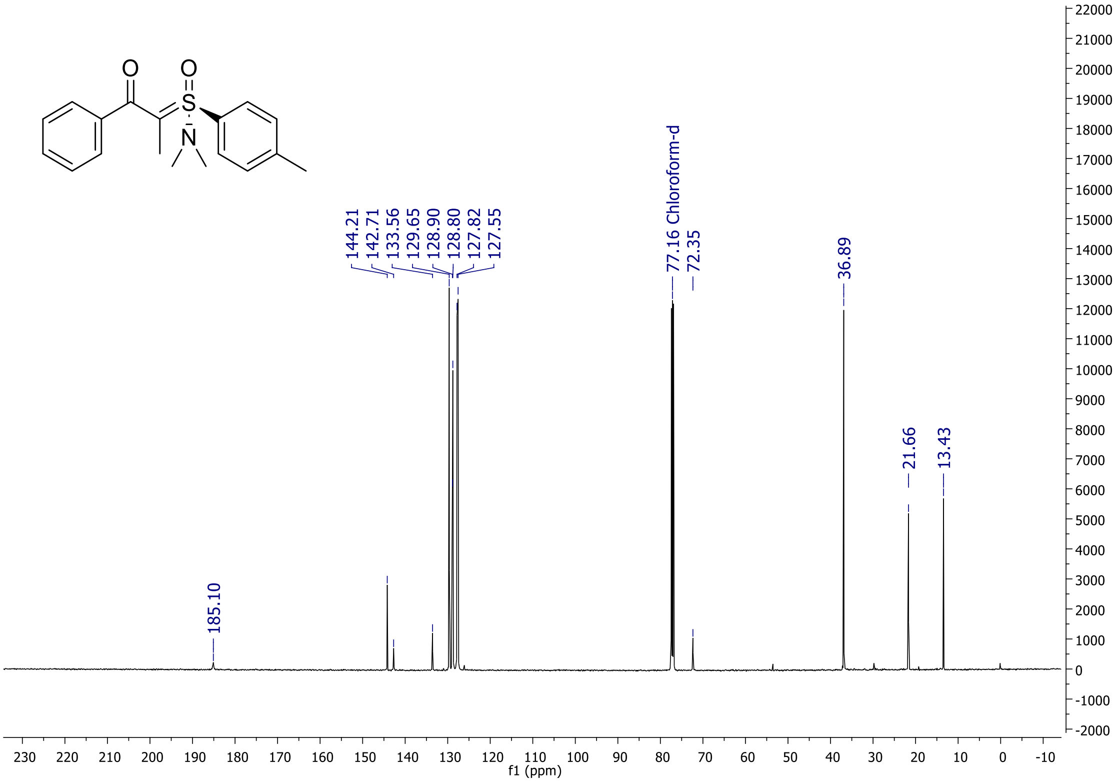

^{1}H NMR *(500 MHz, $CDCl_3$)* of Molecule **1b**:

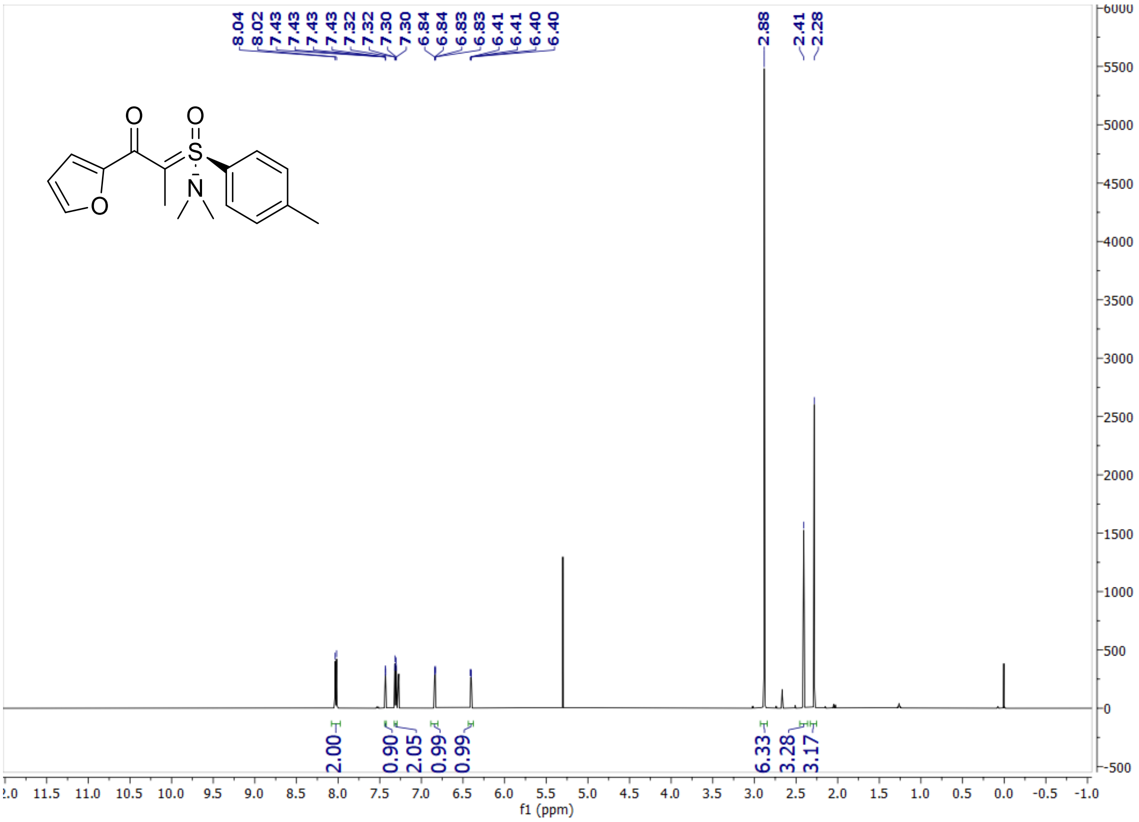

^{13}C{H} NMR *(125 MHz, $CDCl_3$)* of Molecule **1b**:

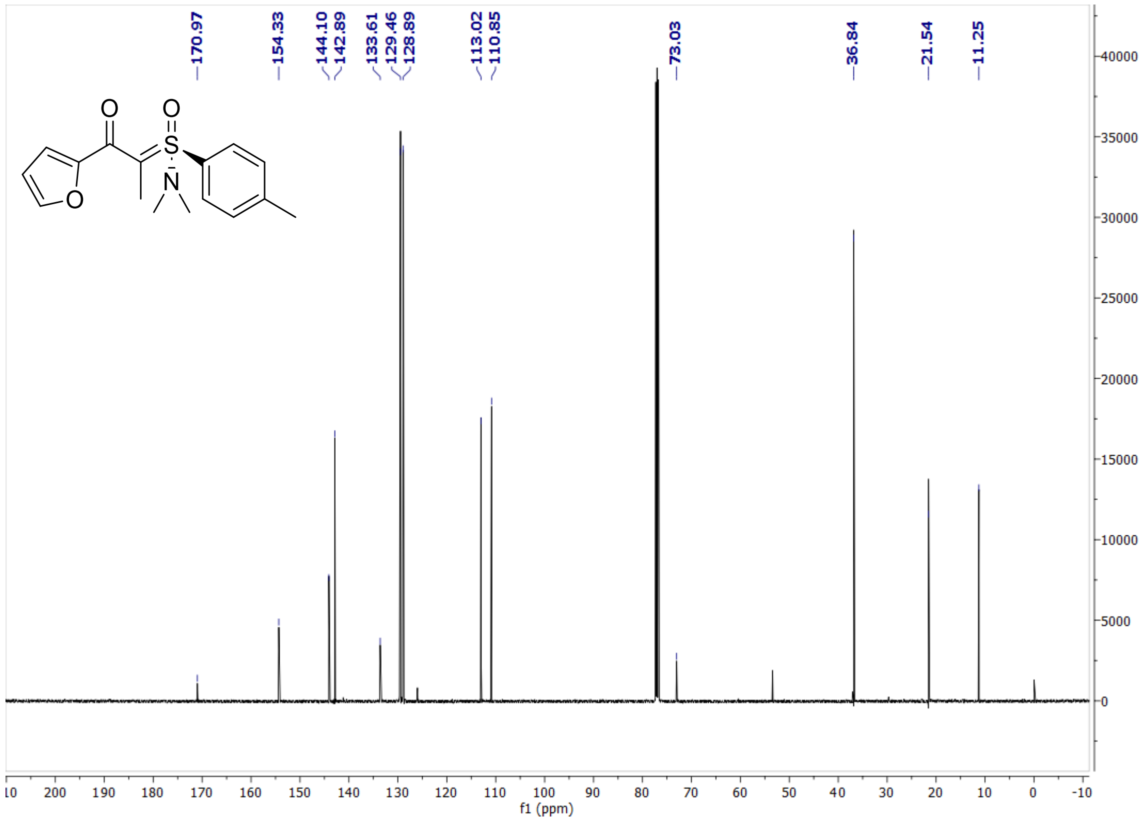

^{1}H NMR *(500 MHz, $CDCl_3$)* of Molecule **1c**:

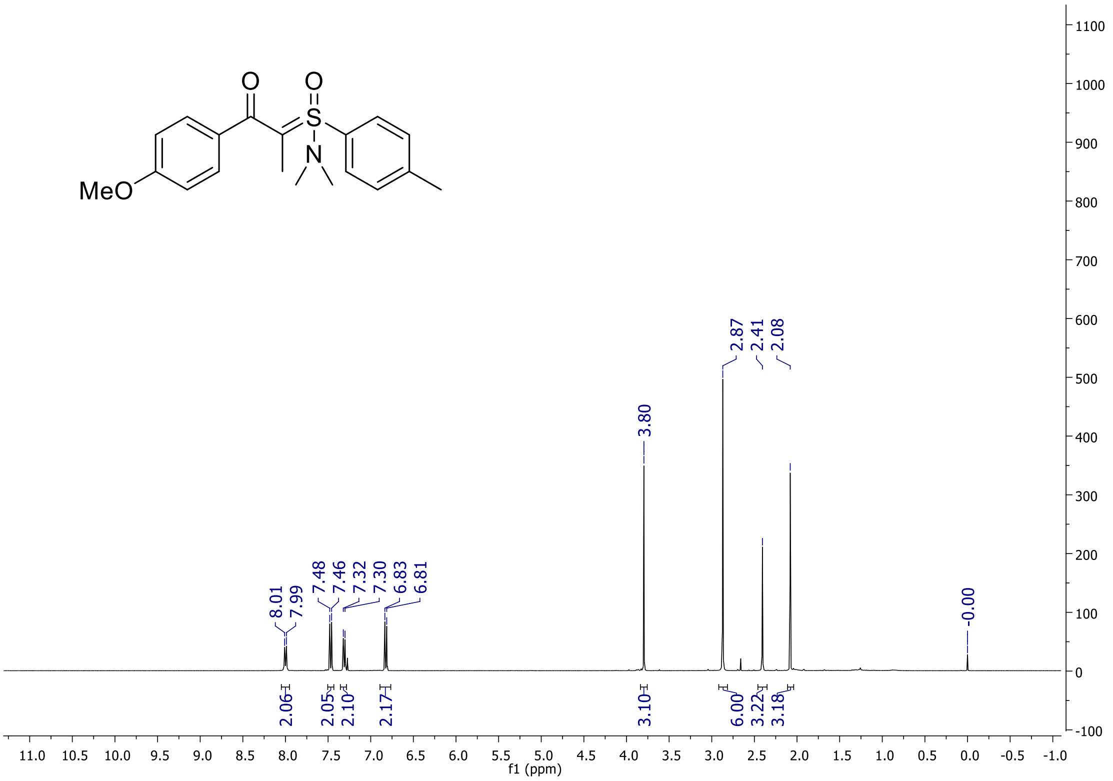

$^{13}C\{H\}$ NMR *(125 MHz, $CDCl_3$)* of Molecule **1c**:

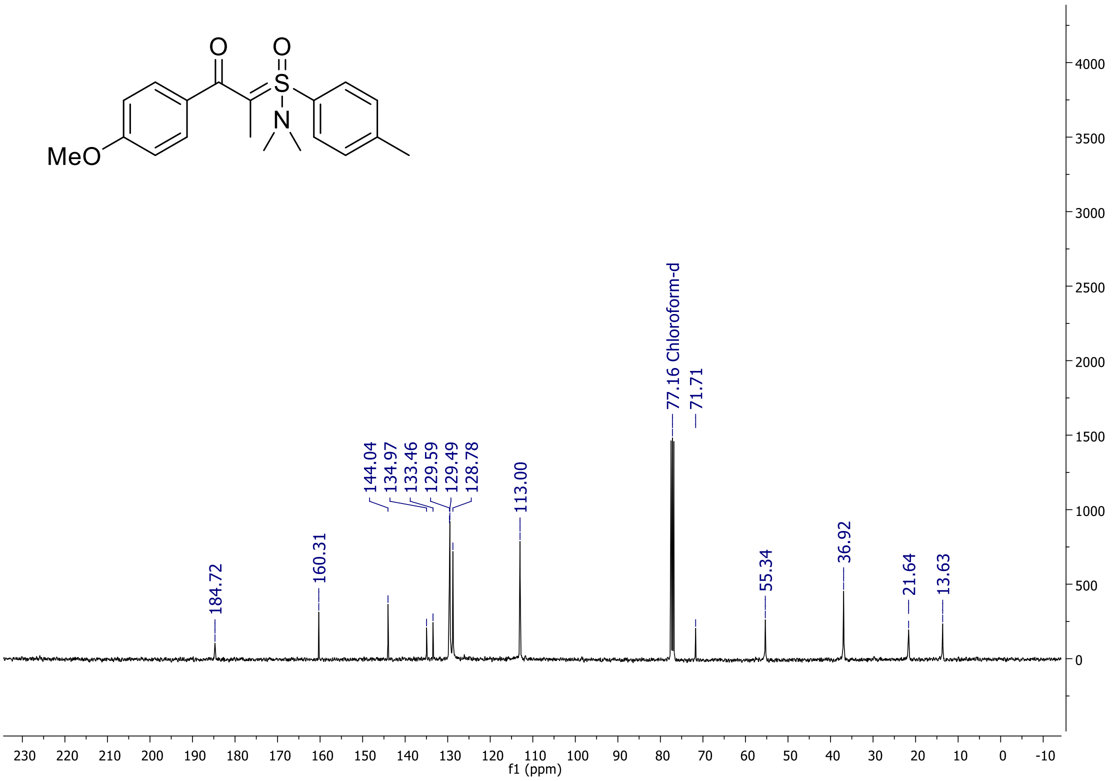

^{1}H NMR *(500 MHz, $CDCl_3$)* of Molecule **1d**:

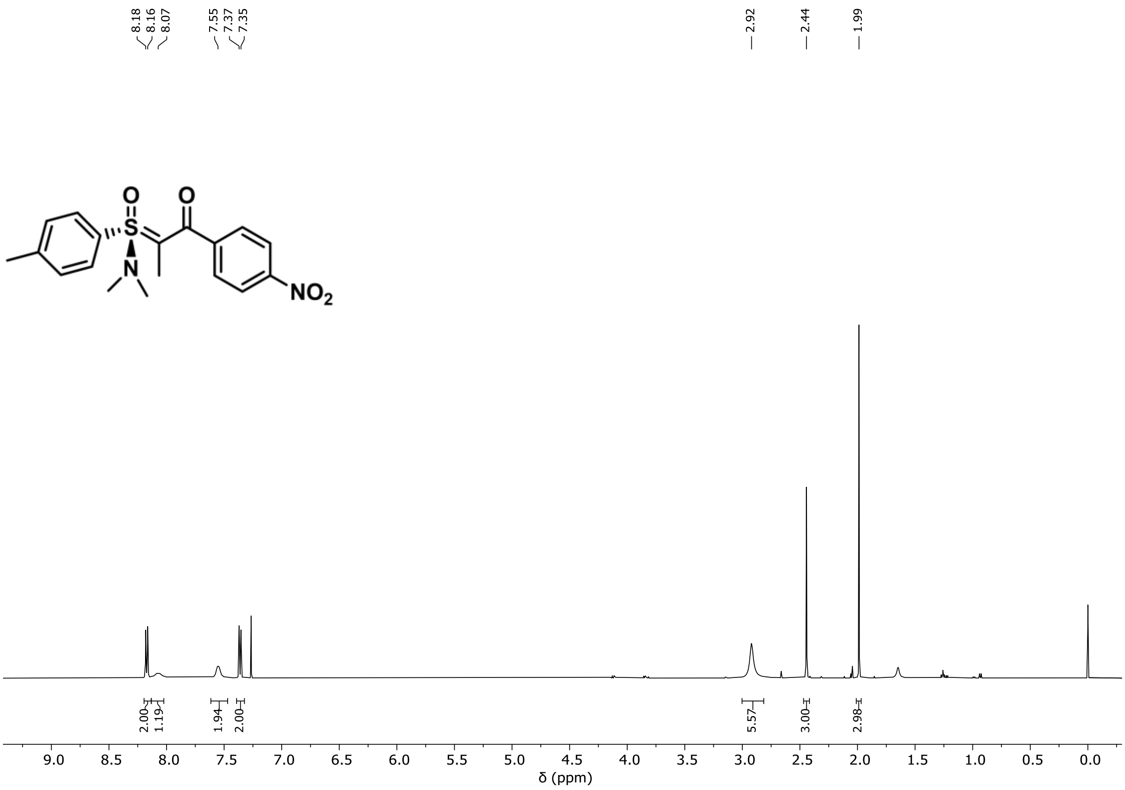

^{13}C{H} NMR *(125 MHz, $CDCl_3$)* of Molecule **1d**:

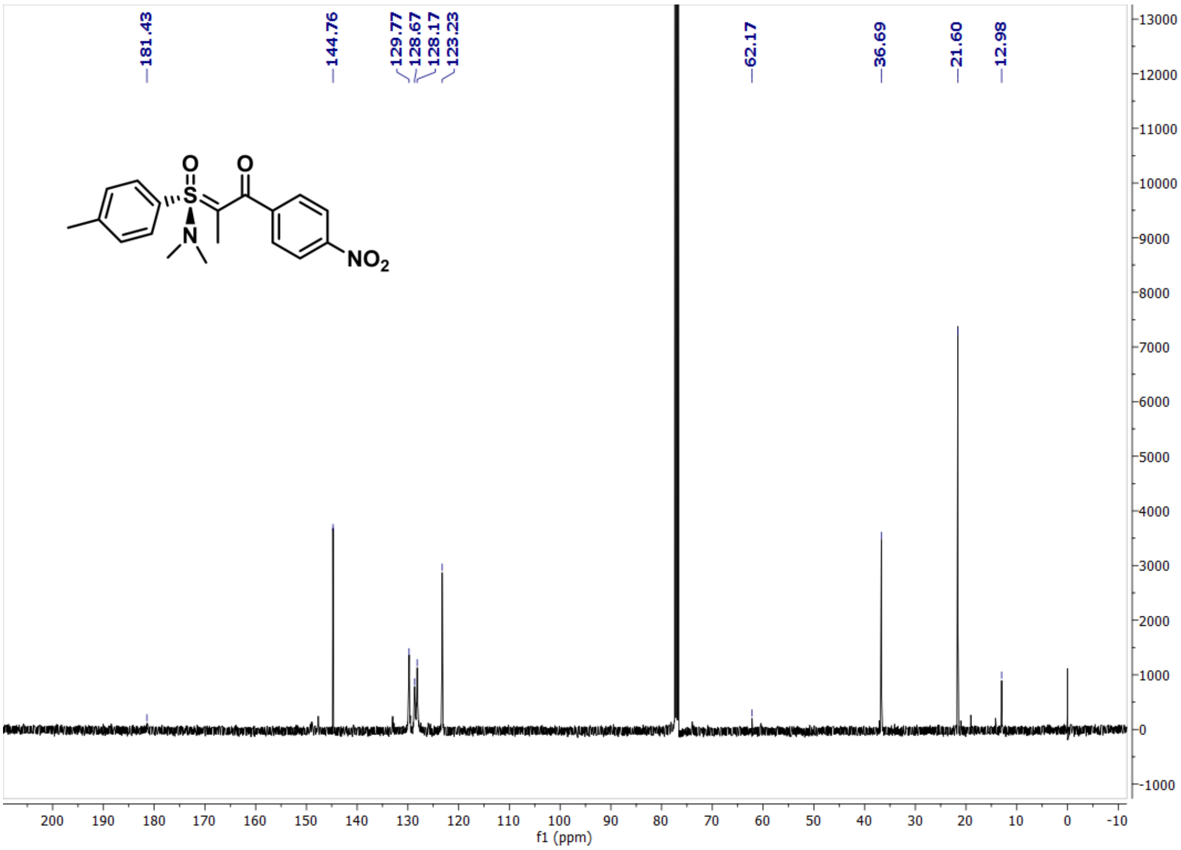

^{1}H NMR *(500 MHz, $CDCl_3$)* of Molecule **1e**:

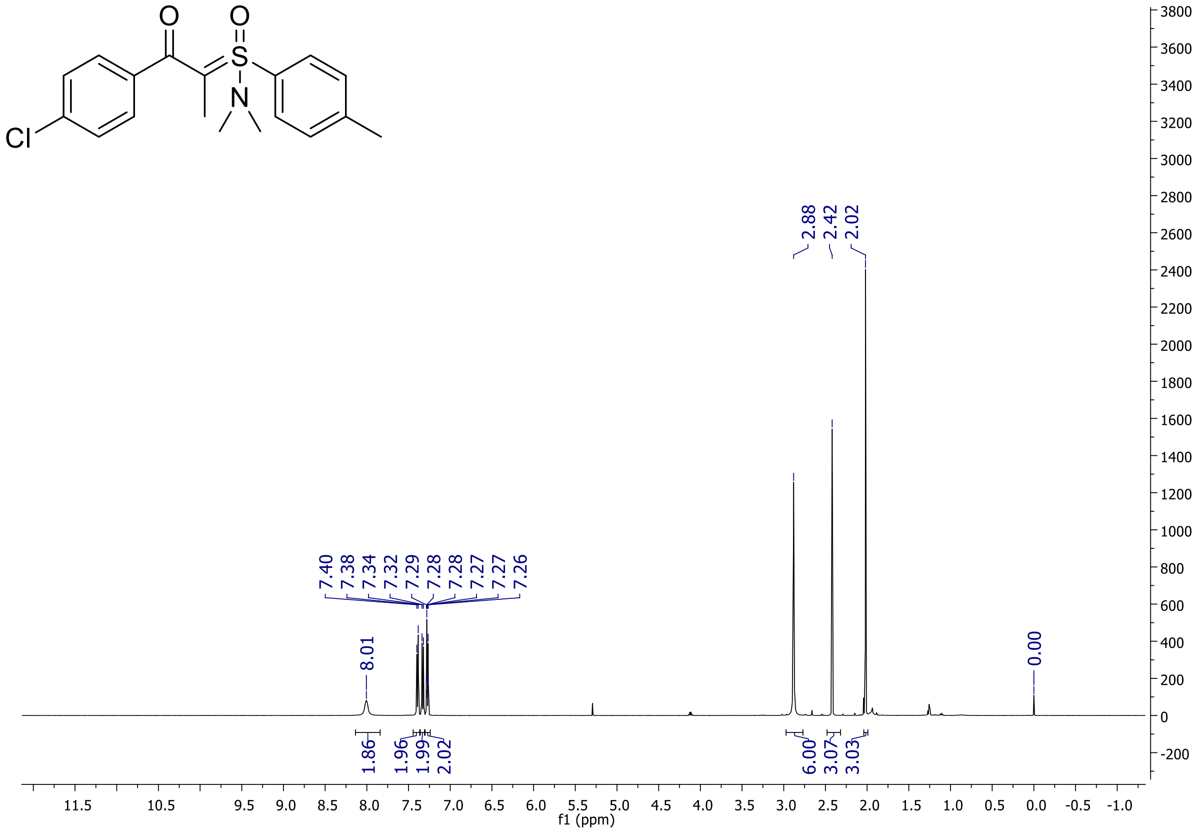

$^{13}C\{H\}$ NMR *(125 MHz, $CDCl_3$)* of Molecule **1e**:

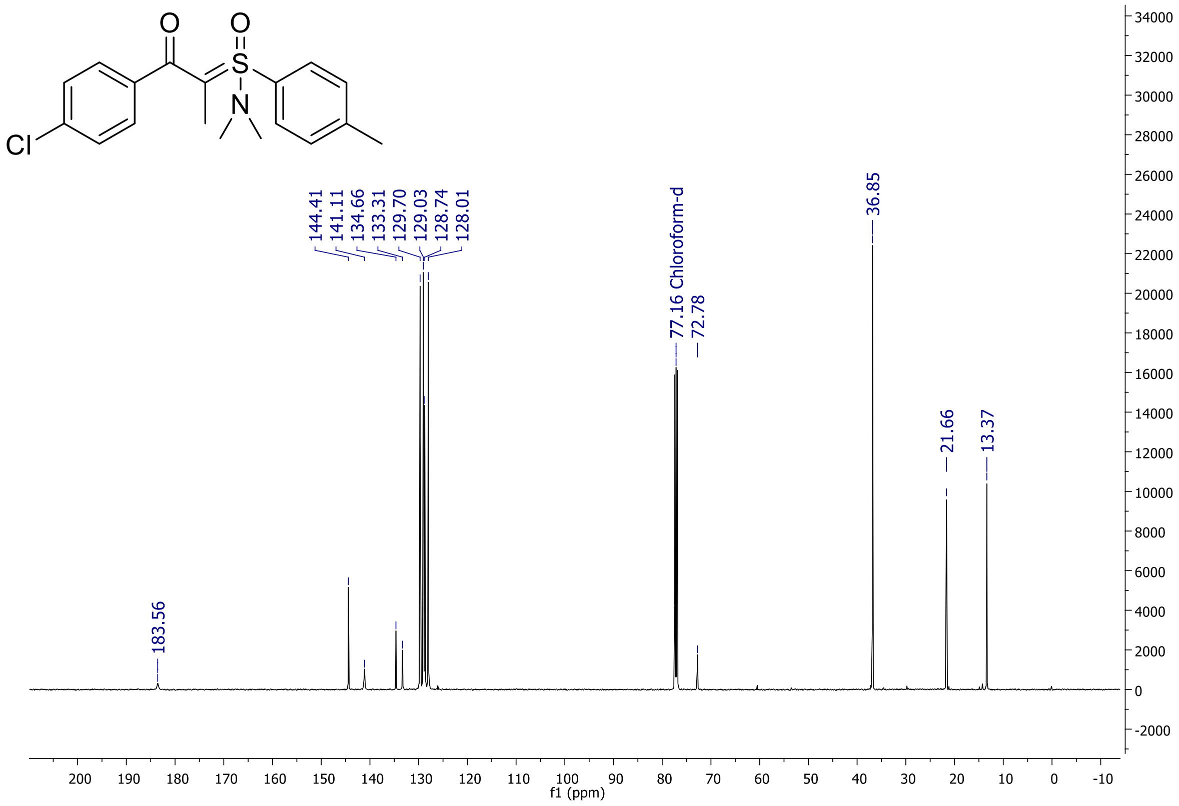

^{1}H NMR *(500 MHz, $CDCl_3$)* of Molecule **1f**:

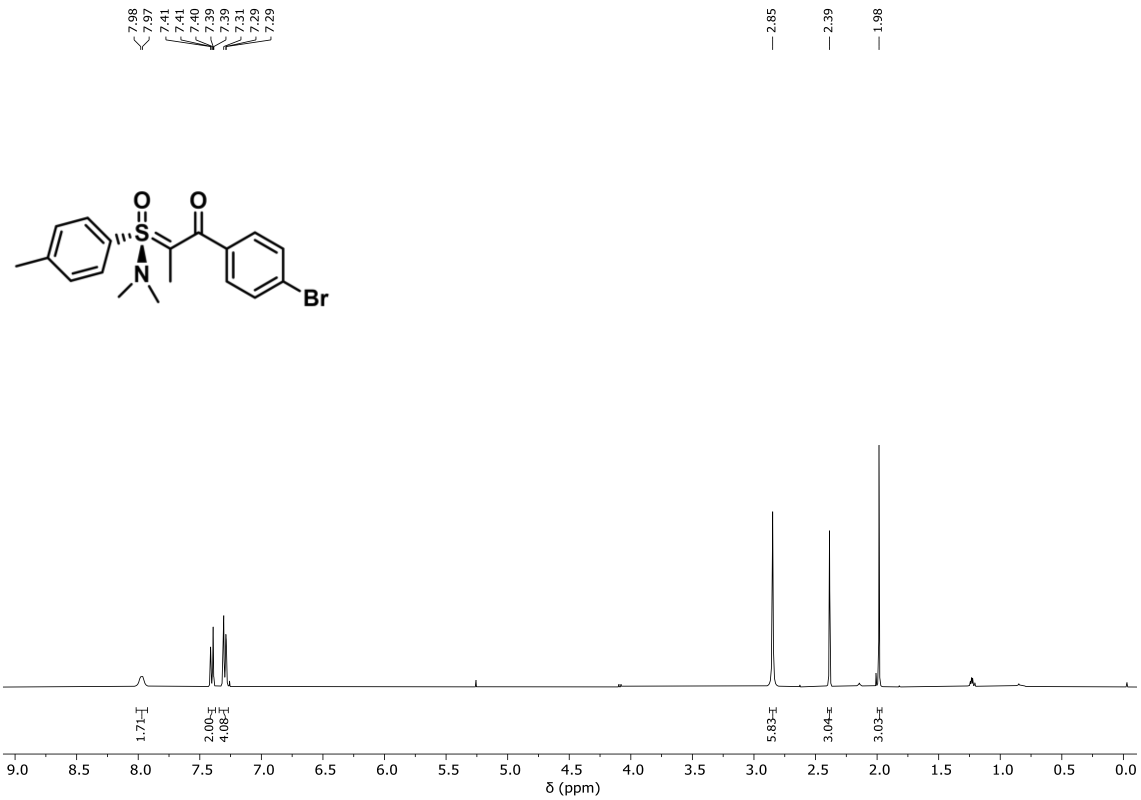

$^{13}C\{H\}$ NMR *(125 MHz, $CDCl_3$)* of Molecule **1f**:

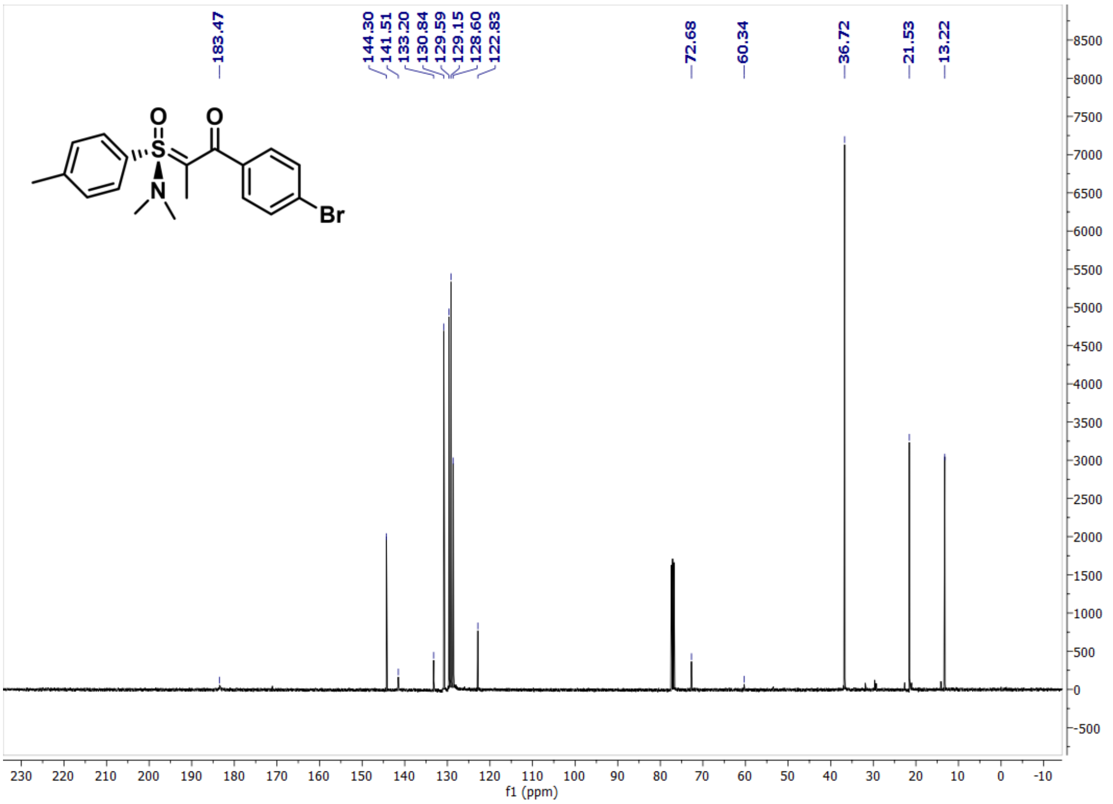

^{1}H NMR *(500 MHz, $CDCl_3$)* of Molecule **1g**:

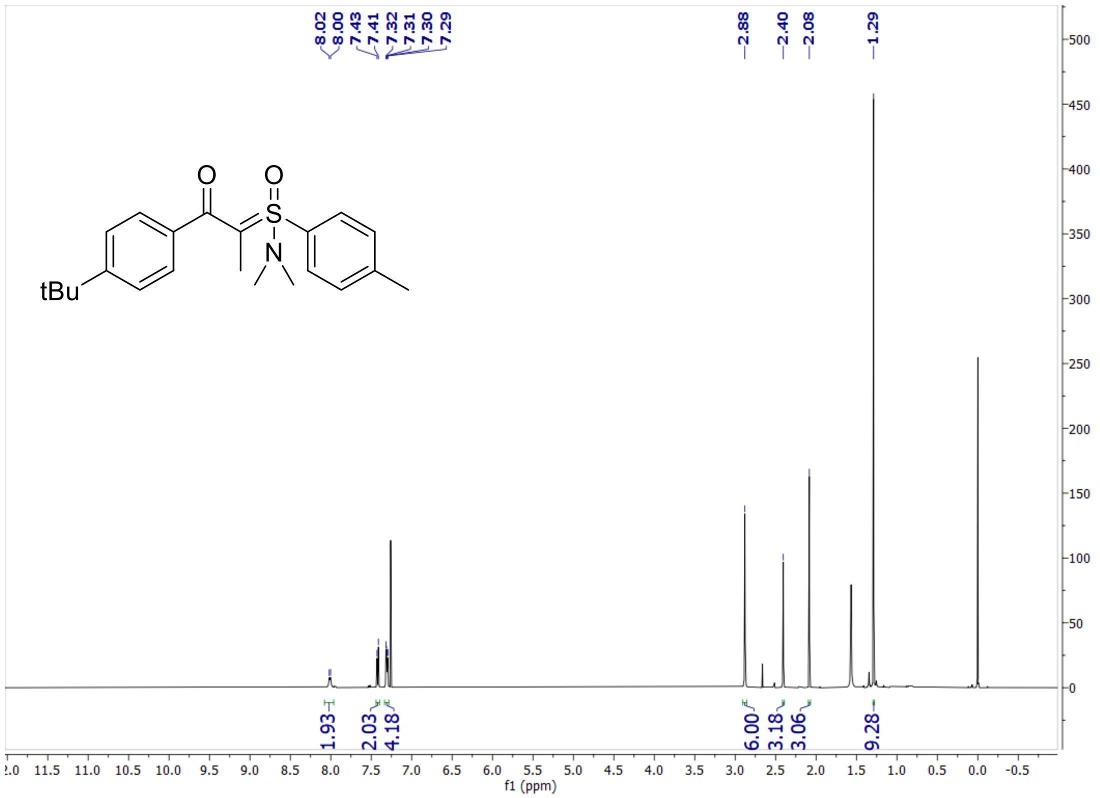

$^{13}C\{H\}$ NMR *(125 MHz, $CDCl_3$)* of Molecule **1g**:

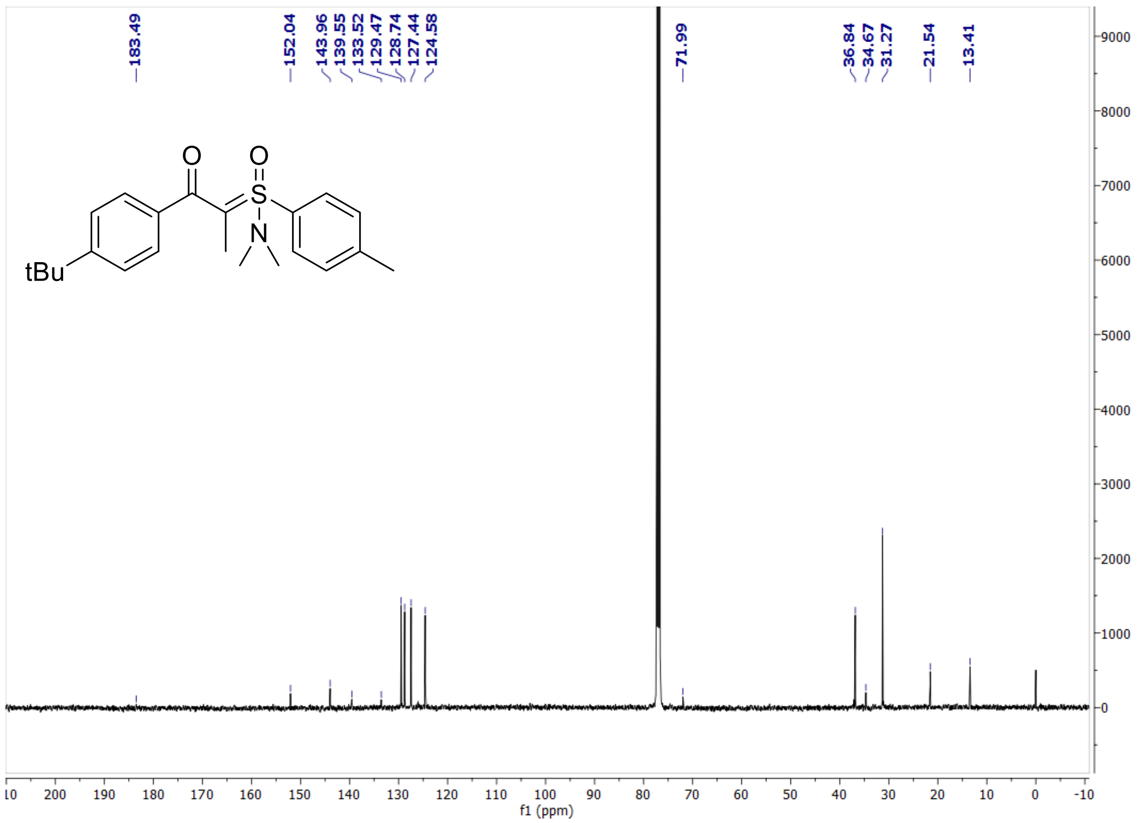

^{1}H NMR *(500 MHz, $CDCl_3$)* of Molecule **1h**:

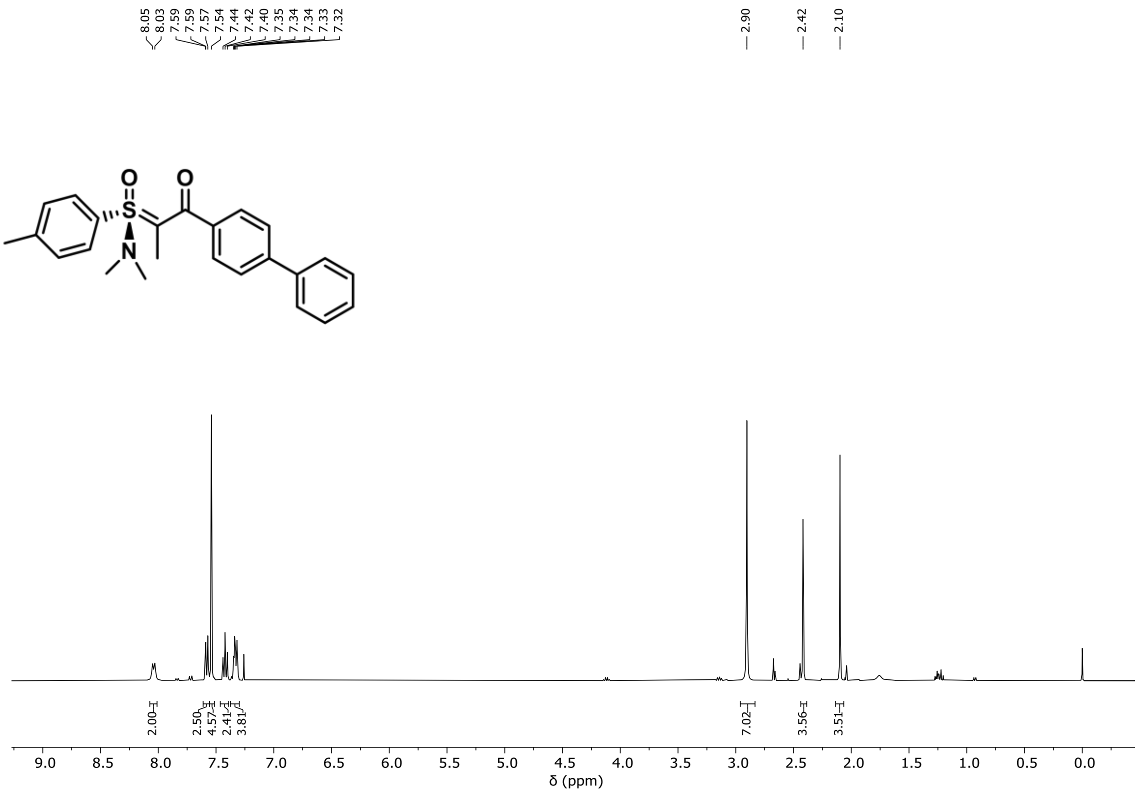

$^{13}C\{H\}$ NMR *(125 MHz, $CDCl_3$)* of Molecule **1h**:

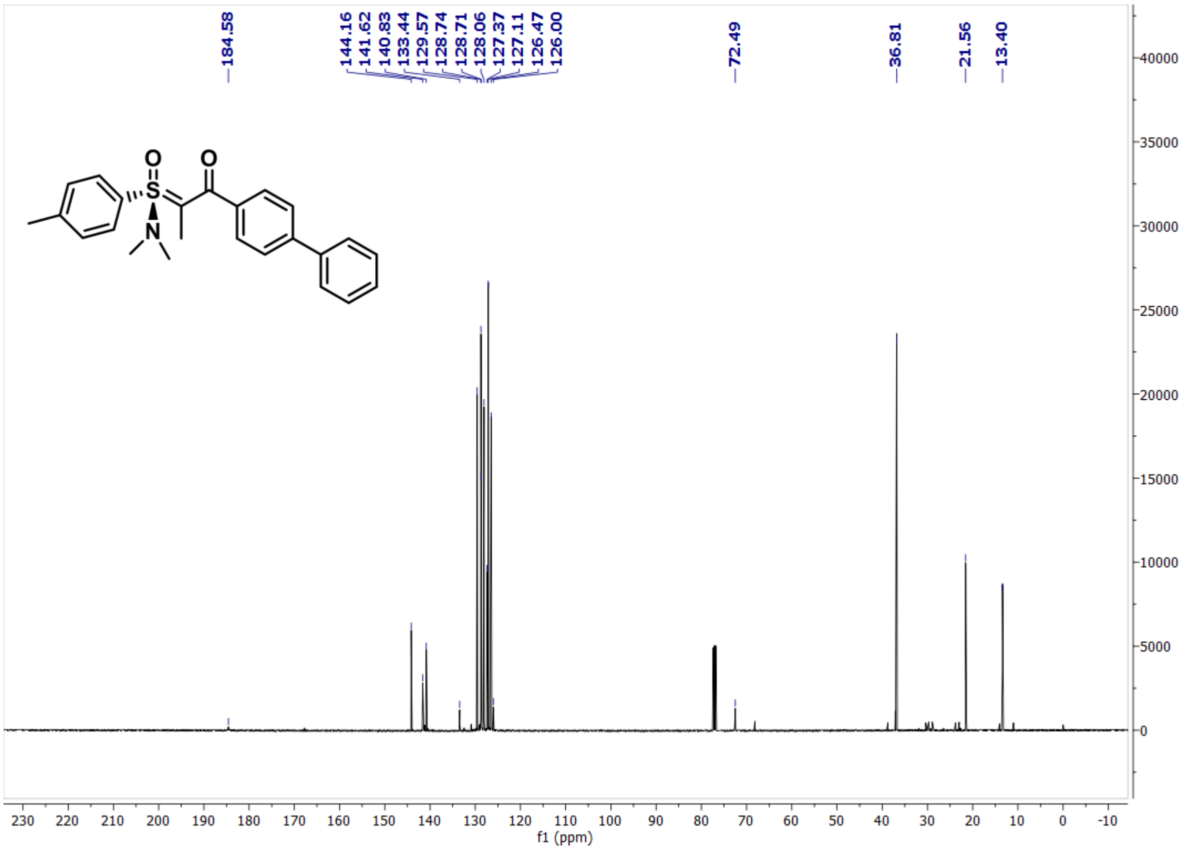

^{1}H NMR *(500 MHz, $CDCl_3$)* of Molecule **1i**:

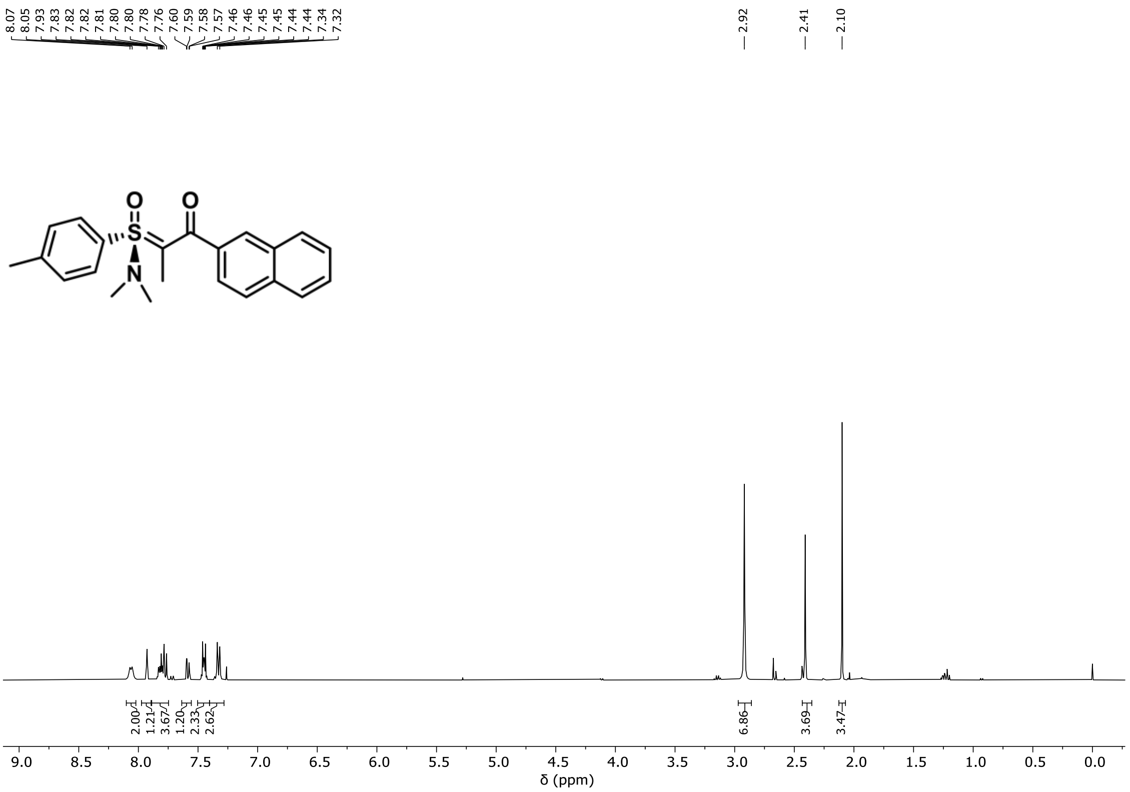

^{13}C{H} NMR *(125 MHz, $CDCl_3$)* of Molecule **1i**:

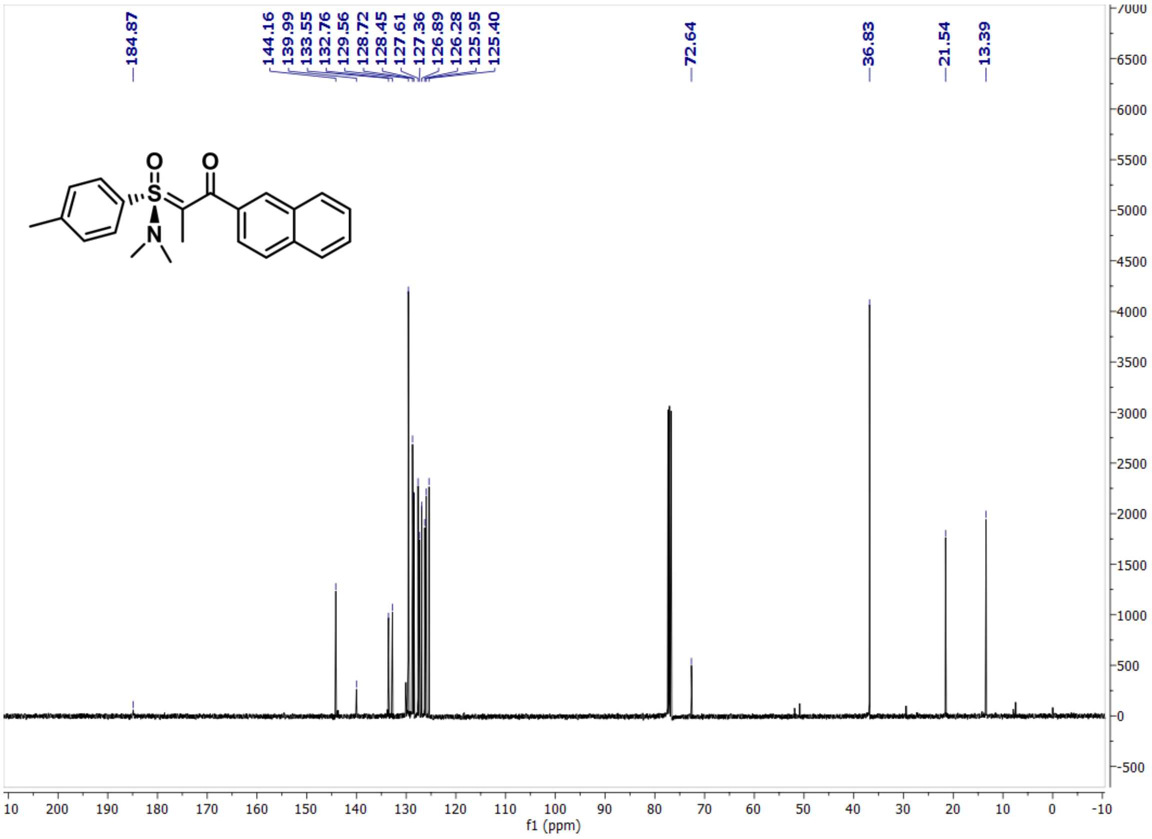

^{1}H NMR *(500 MHz, $CDCl_3$)* of Molecule **1j**:

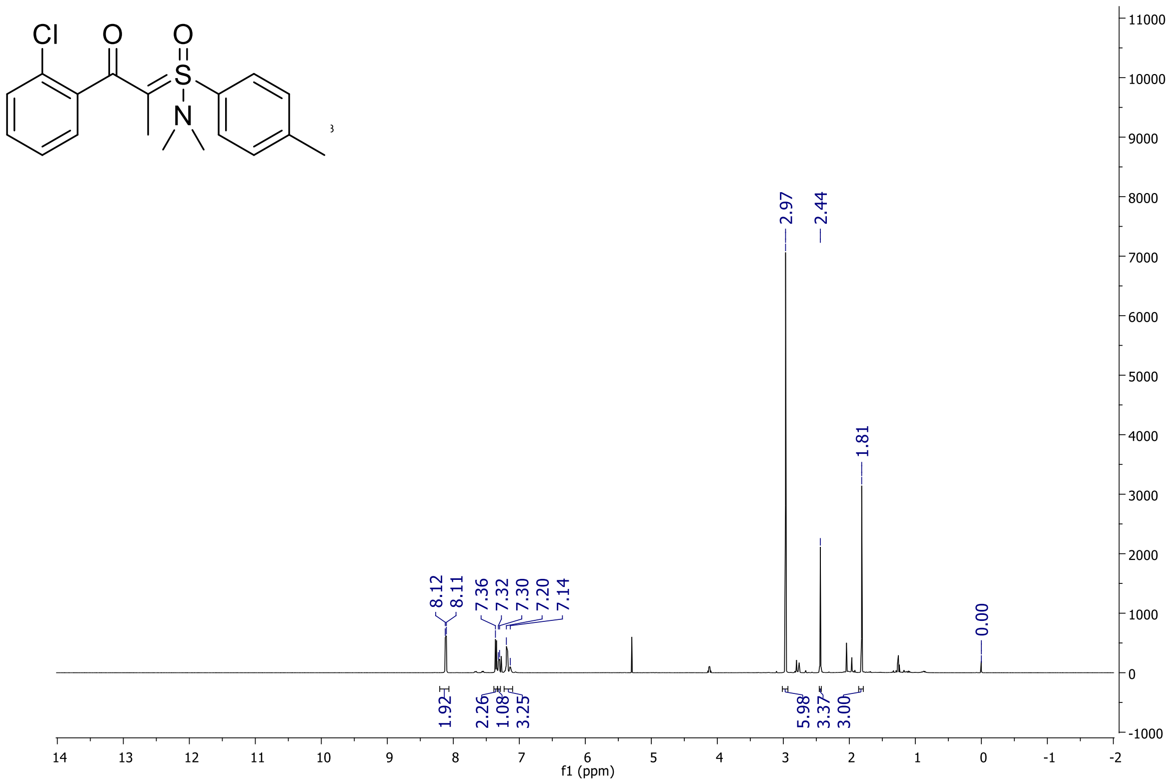

^{13}C{H} NMR *(125 MHz, $CDCl_3$)* of Molecule **1j**:

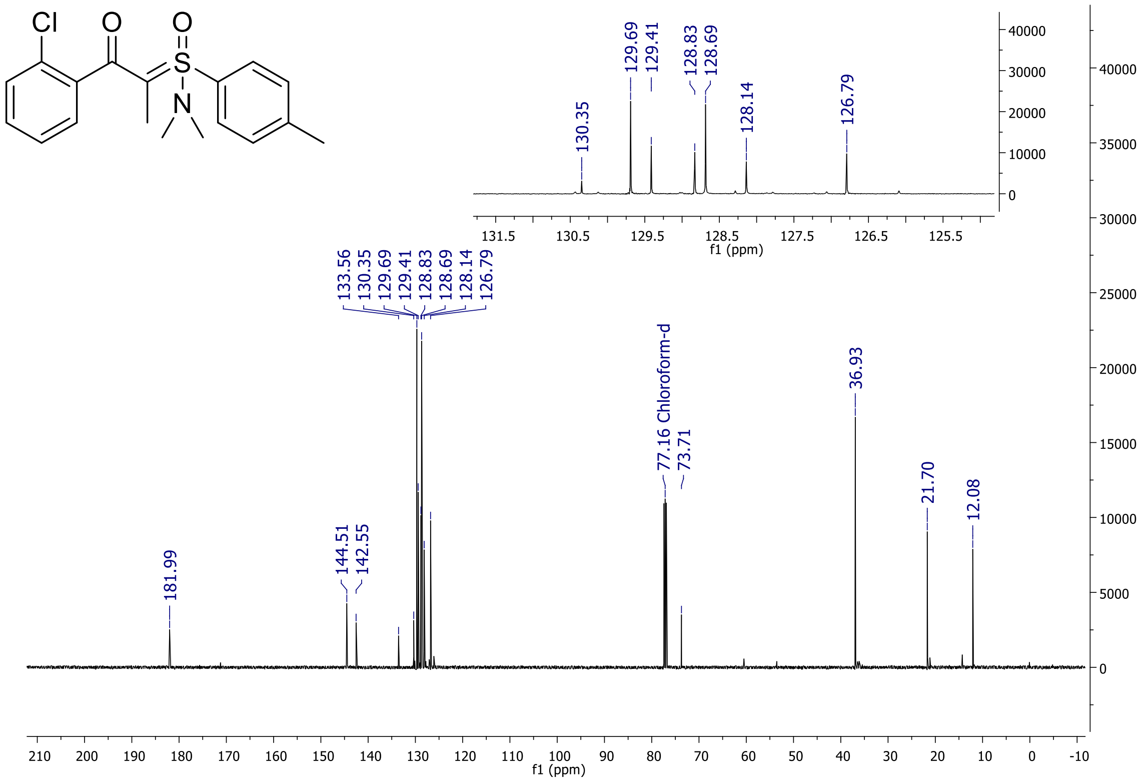

^{1}H NMR *(400 MHz, CDCl$_3$)* of Molecule **1k**:

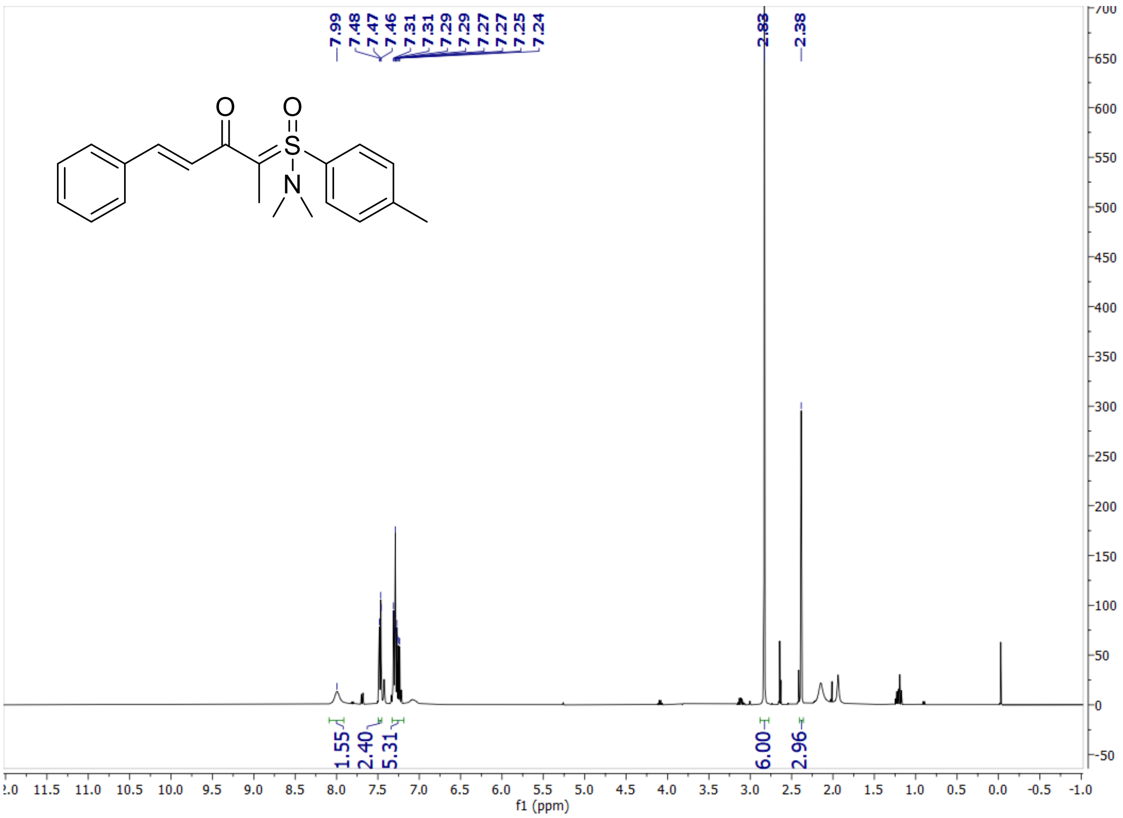

^{13}C{H} NMR *(100 MHz, CDCl$_3$)* of Molecule **1k**:

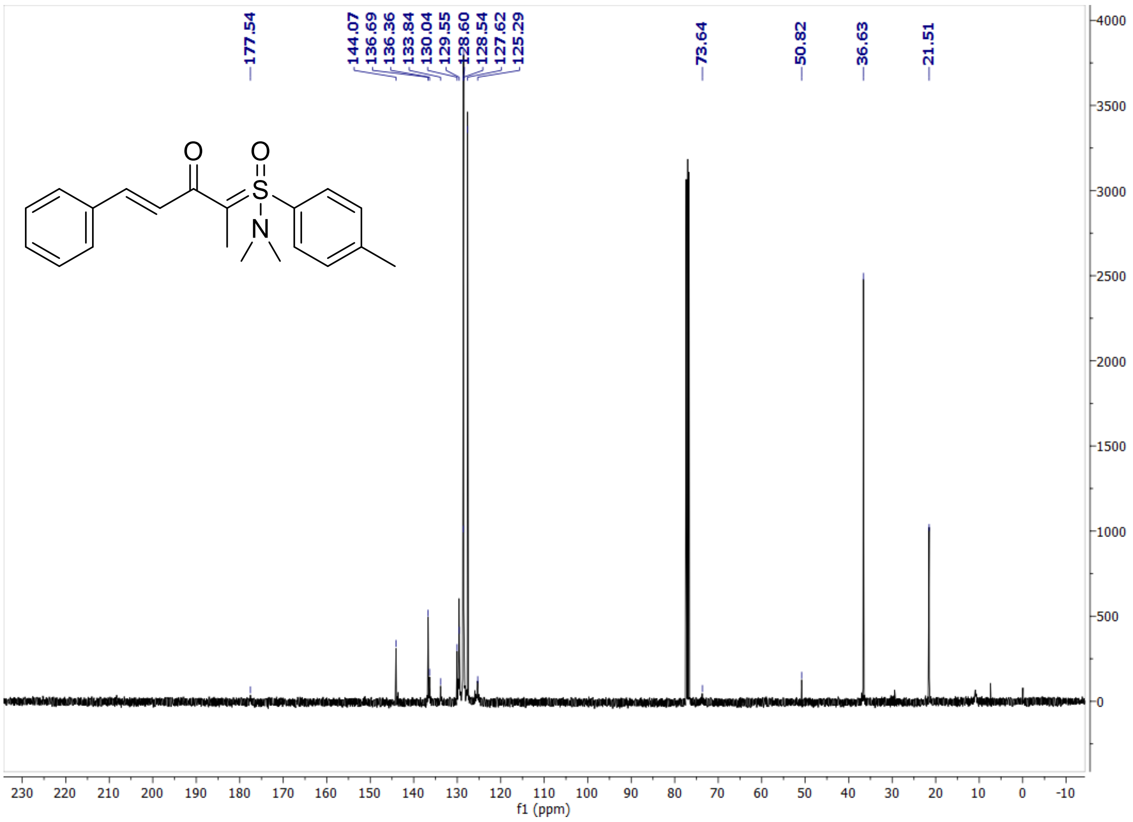

^{1}H NMR *(400 MHz, CDCl$_3$)* of Molecule **1l**:

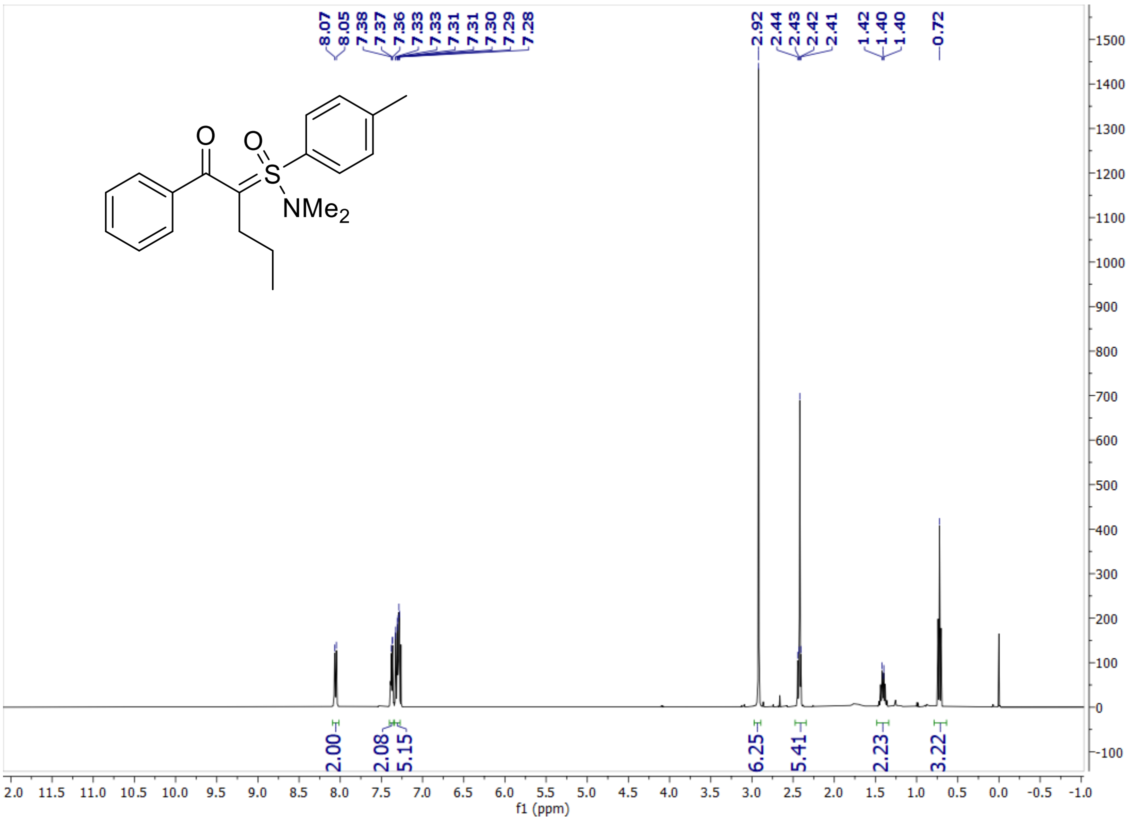

^{13}C{H} NMR *(125 MHz, CDCl$_3$)* of Molecule **1l**:

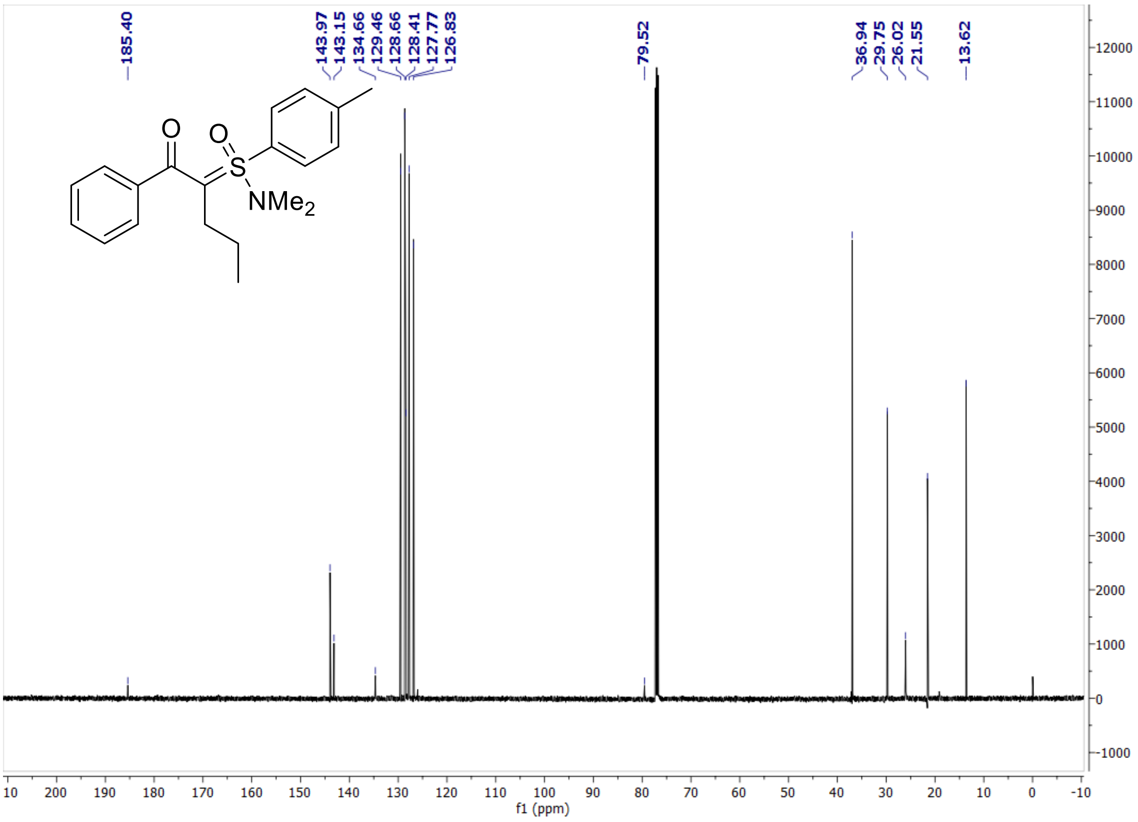

^{1}H NMR *(400 MHz, $CDCl_3$)* of Molecule **1m**:

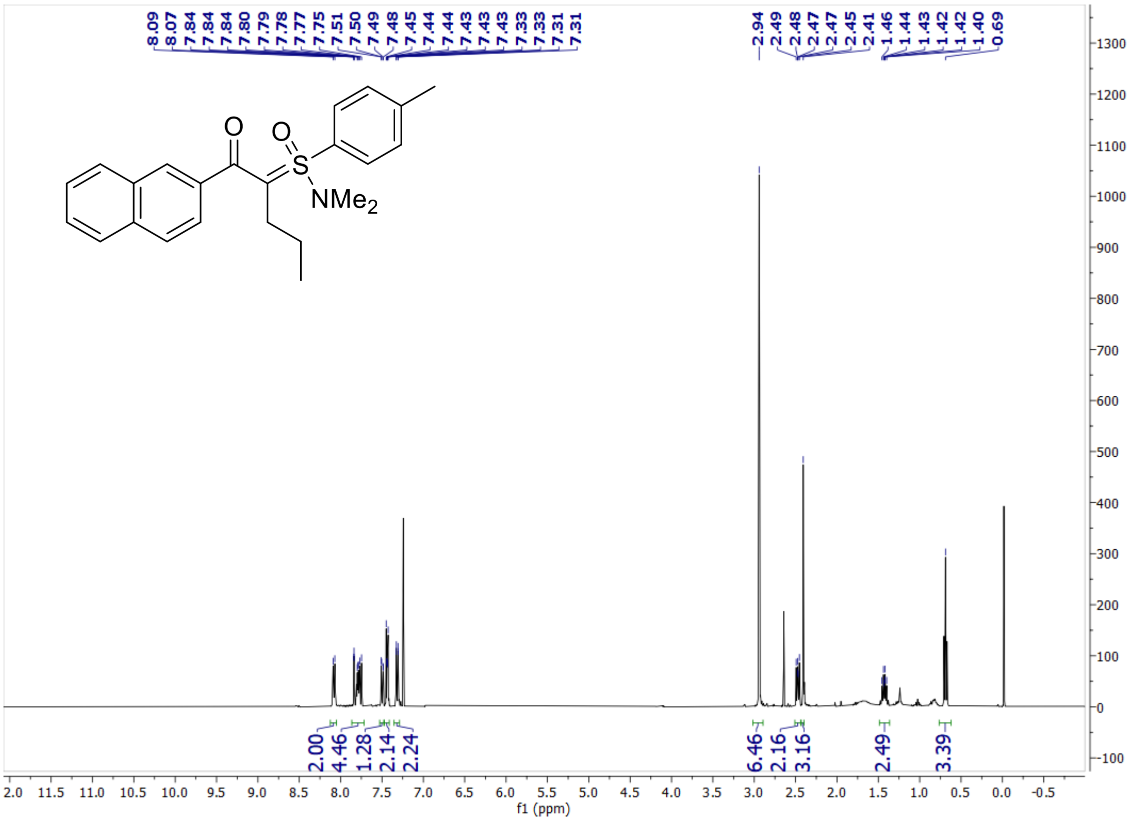

^{13}C{H} NMR *(100 MHz, $CDCl_3$)* of Molecule **1m**:

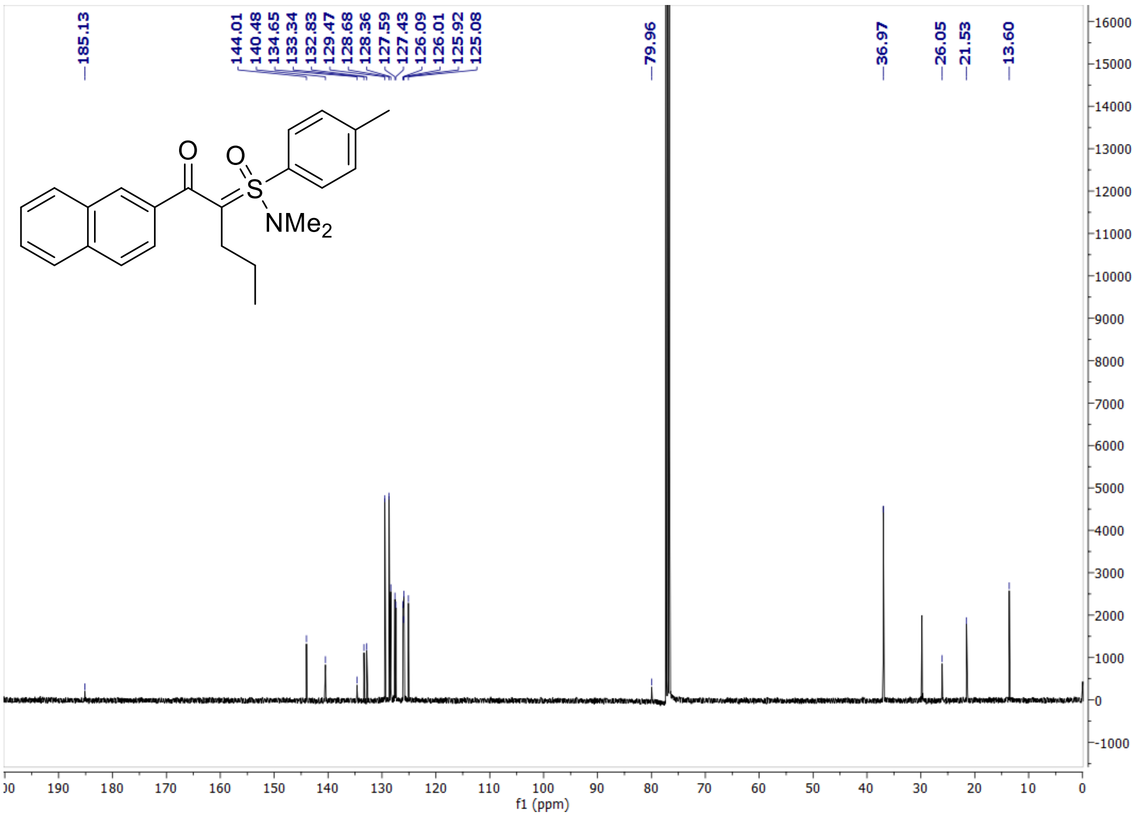

^{1}H NMR *(400 MHz, $CDCl_3$)* of Molecule **1n**:

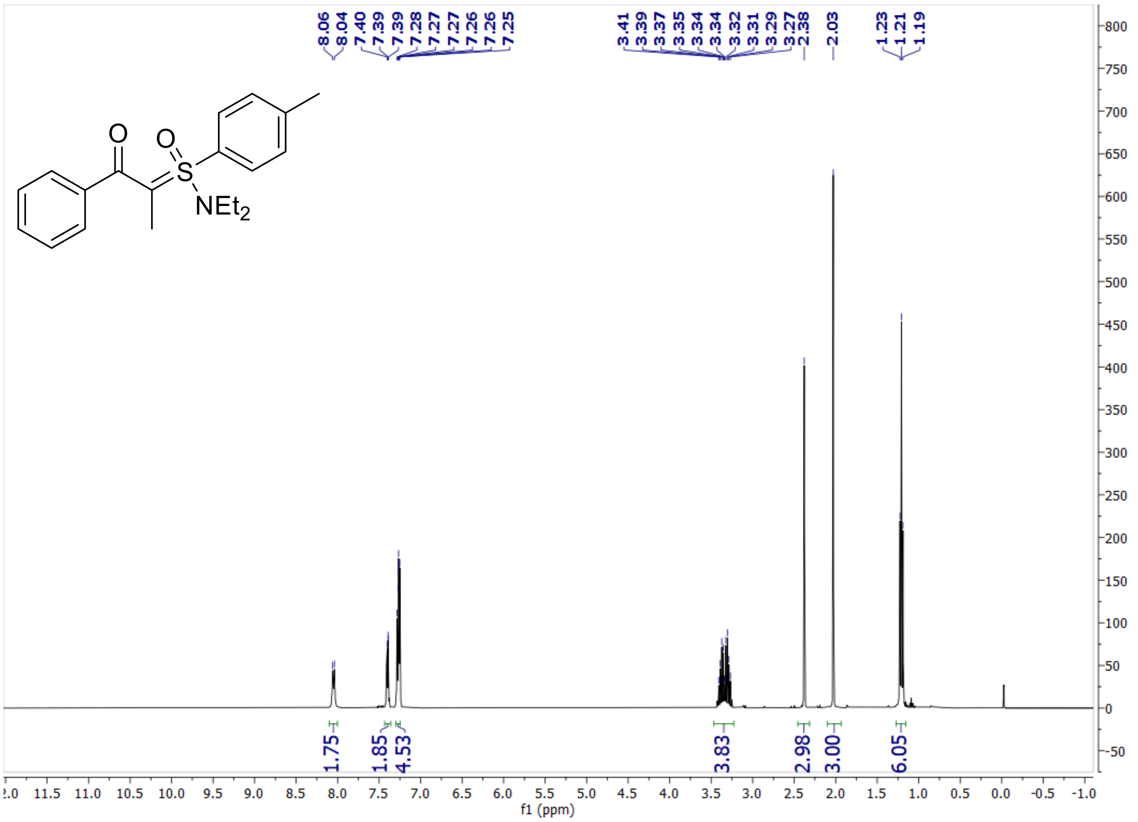

^{13}C{H} NMR *(100 MHz, $CDCl_3$)* of Molecule **1n**:

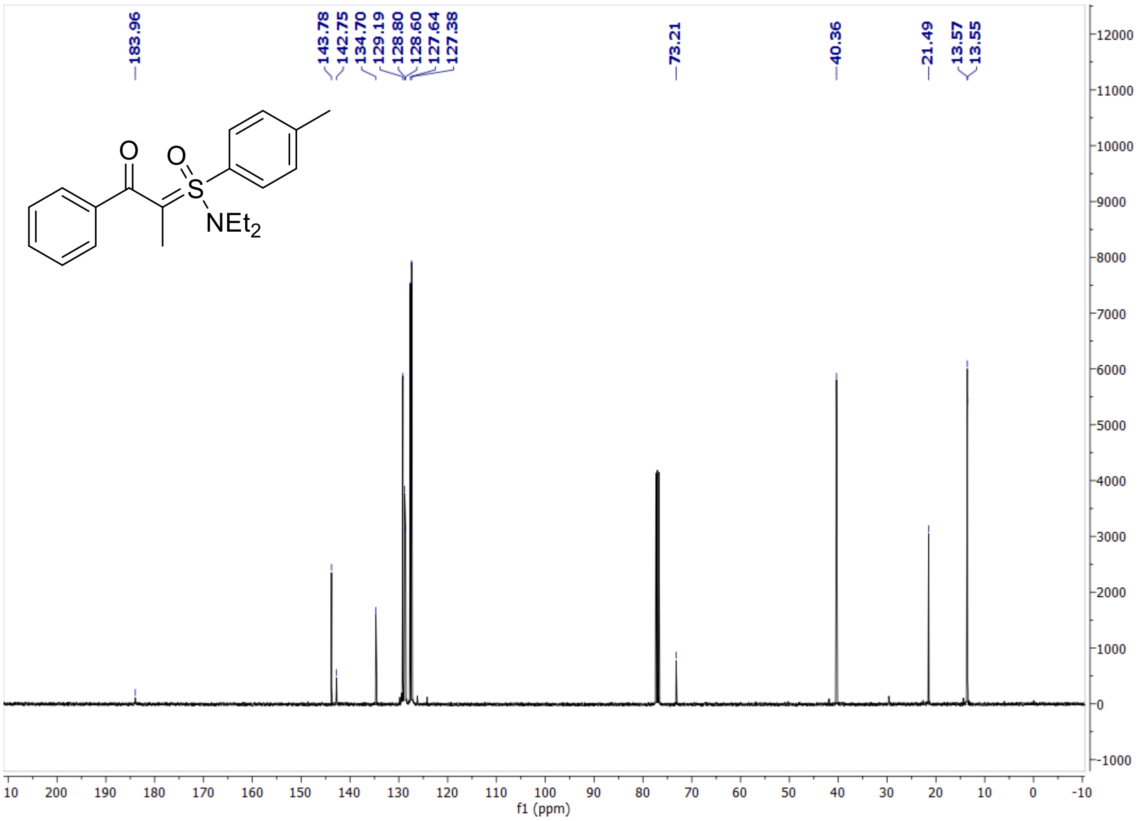

^{1}H NMR *(500 MHz, $CDCl_3$)* of Molecule **2a**:

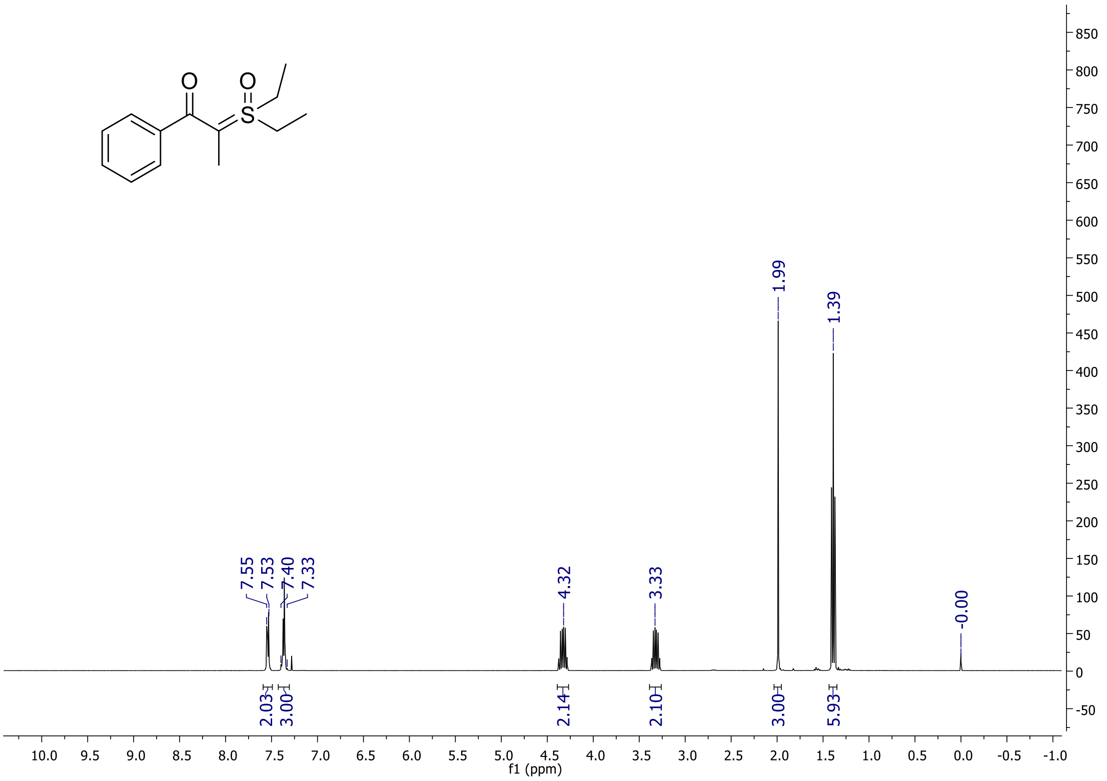

^{13}C{H} NMR *(125 MHz, $CDCl_3$)* of Molecule **2a**:

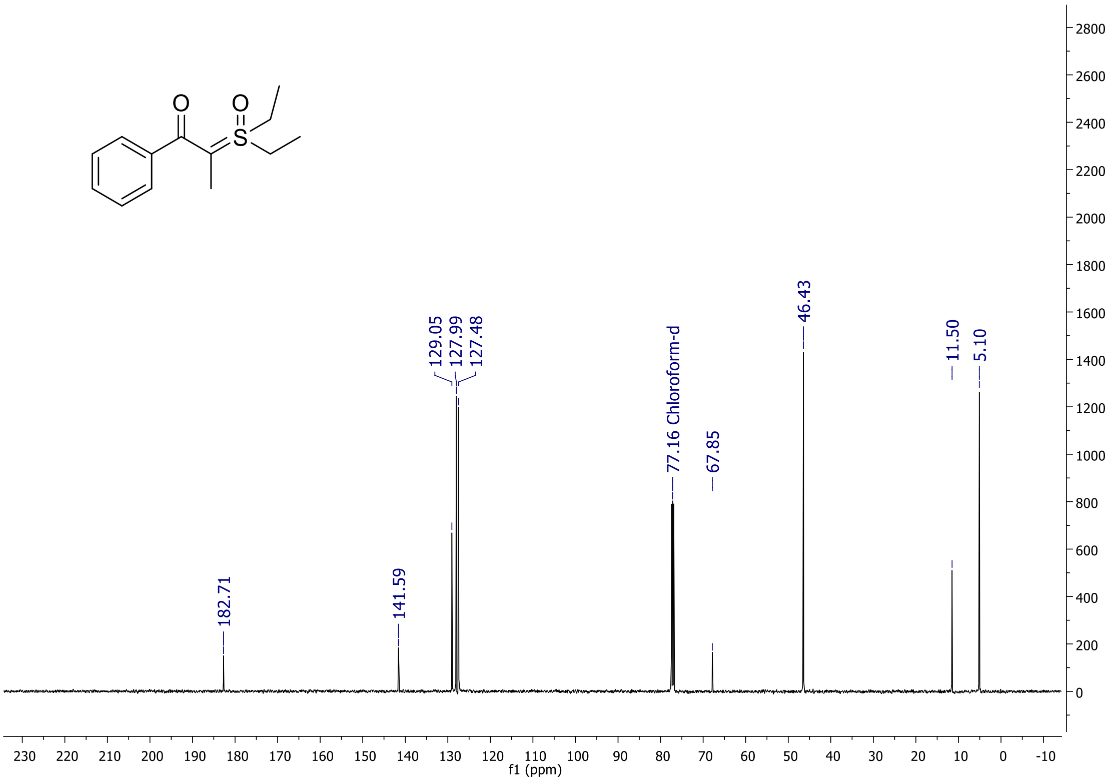

^{1}H NMR *(500 MHz, $CDCl_3$)* of Molecule **2b**:

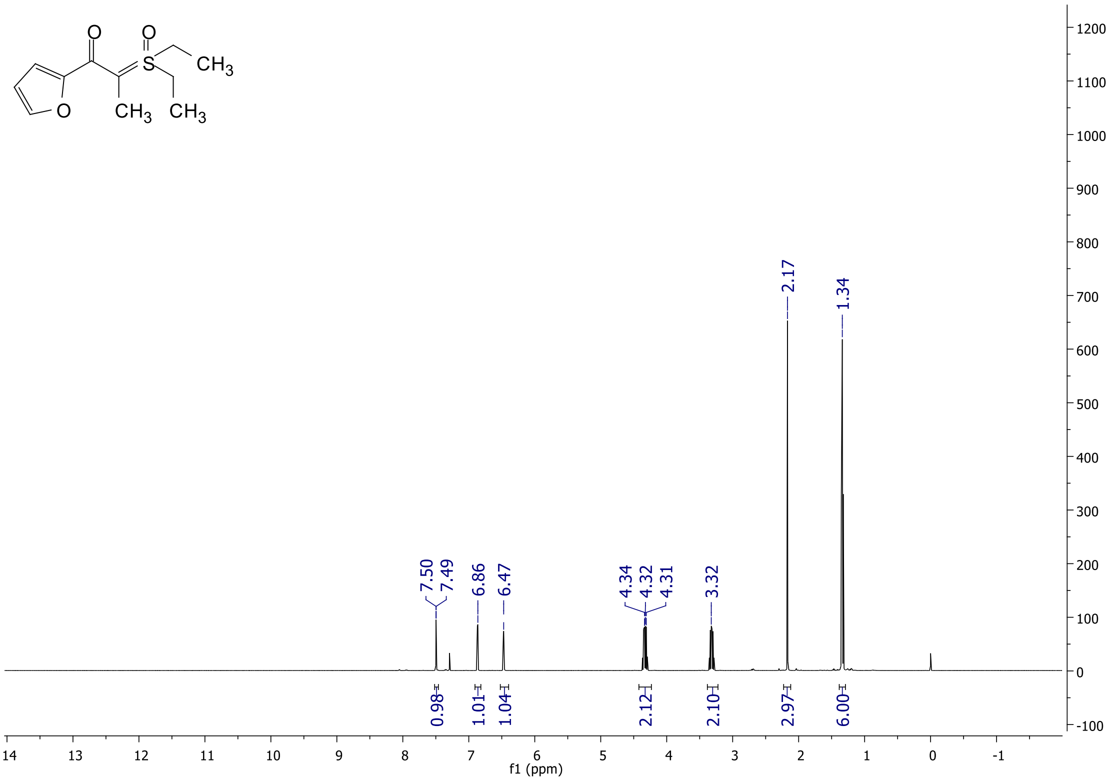

$^{13}C\{H\}$ NMR *(125 MHz, $CDCl_3$)* of Molecule **2b**:

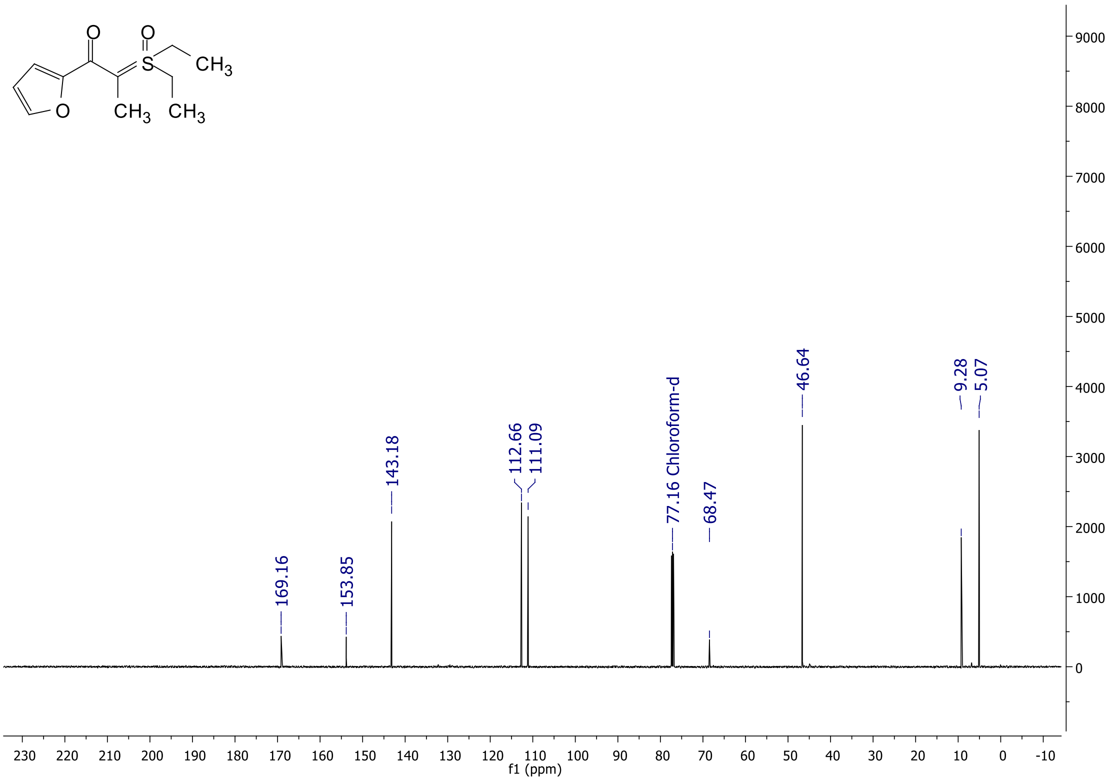

^{1}H NMR *(500 MHz, $CDCl_3$)* of Molecule **2c**:

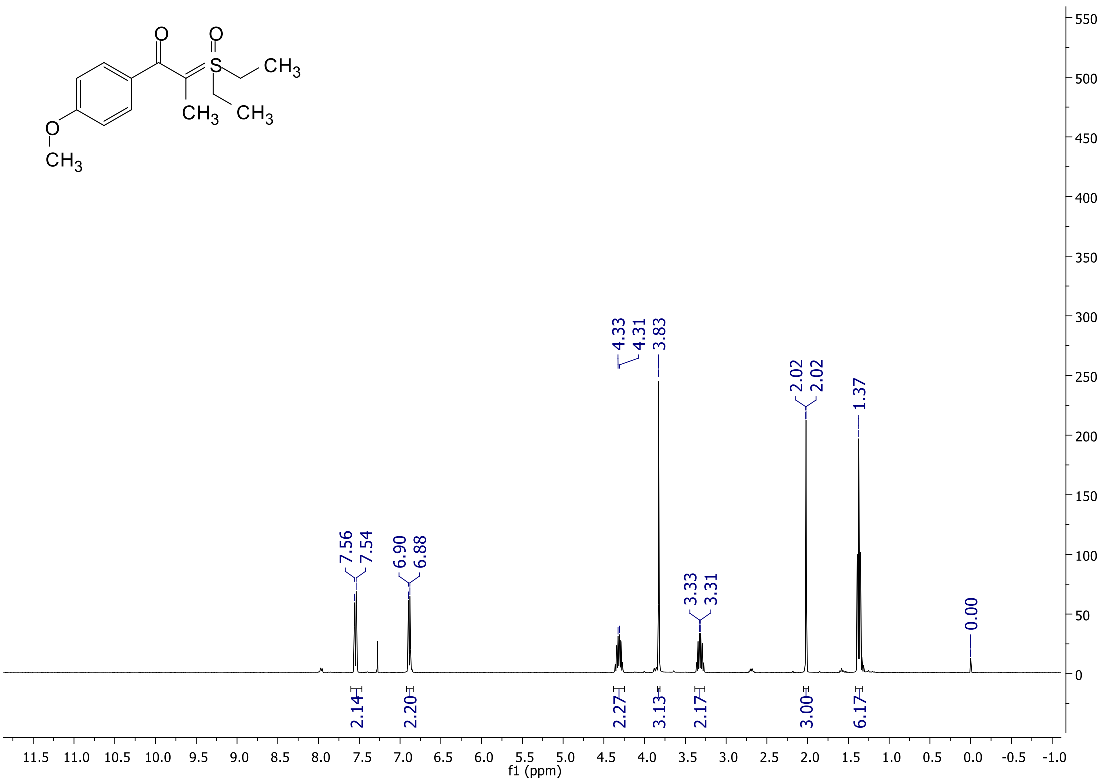

^{13}C{H} NMR *(125 MHz, $CDCl_3$)* of Molecule **2c**:

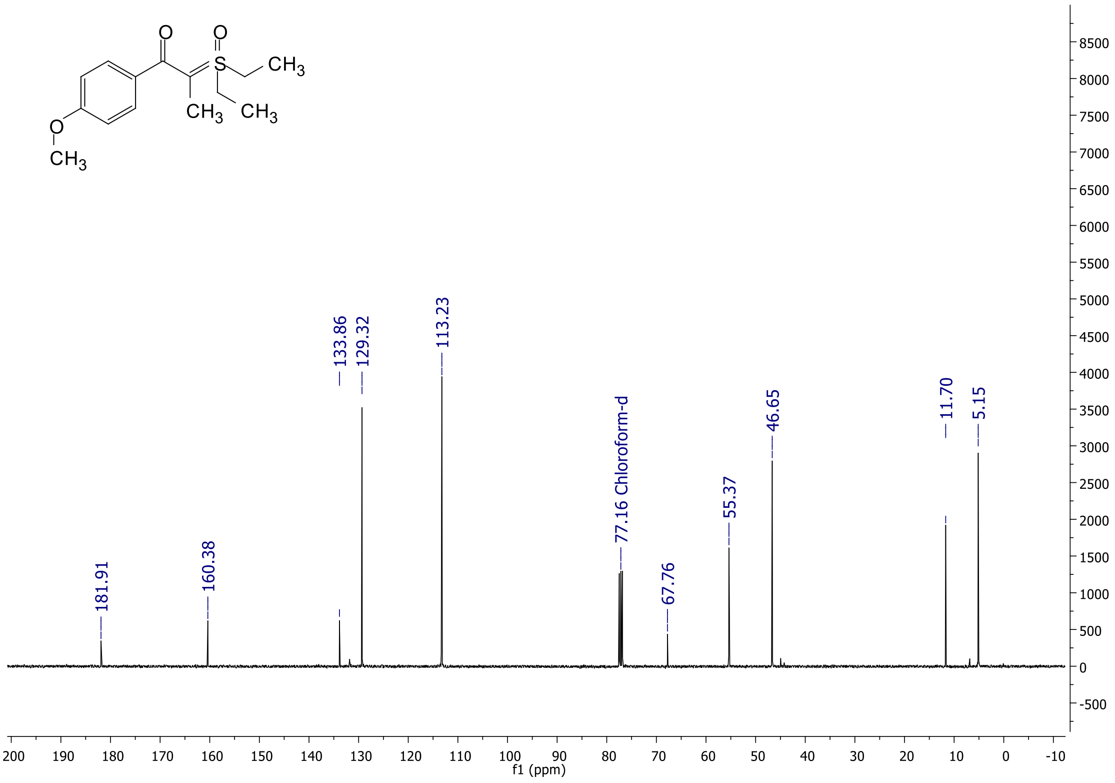

^{1}H NMR *(500 MHz, CDCl$_3$)* of Molecule **2d**:

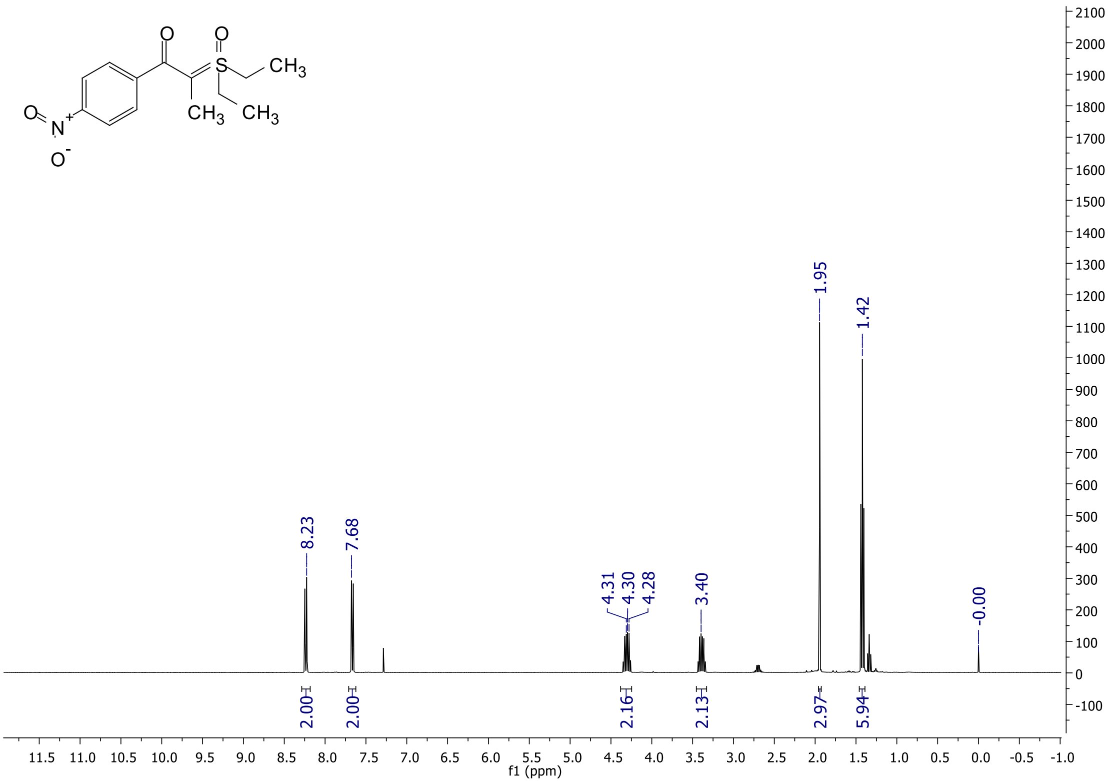

^{13}C{H} NMR *(125 MHz, CDCl$_3$)* of Molecule **2d**:

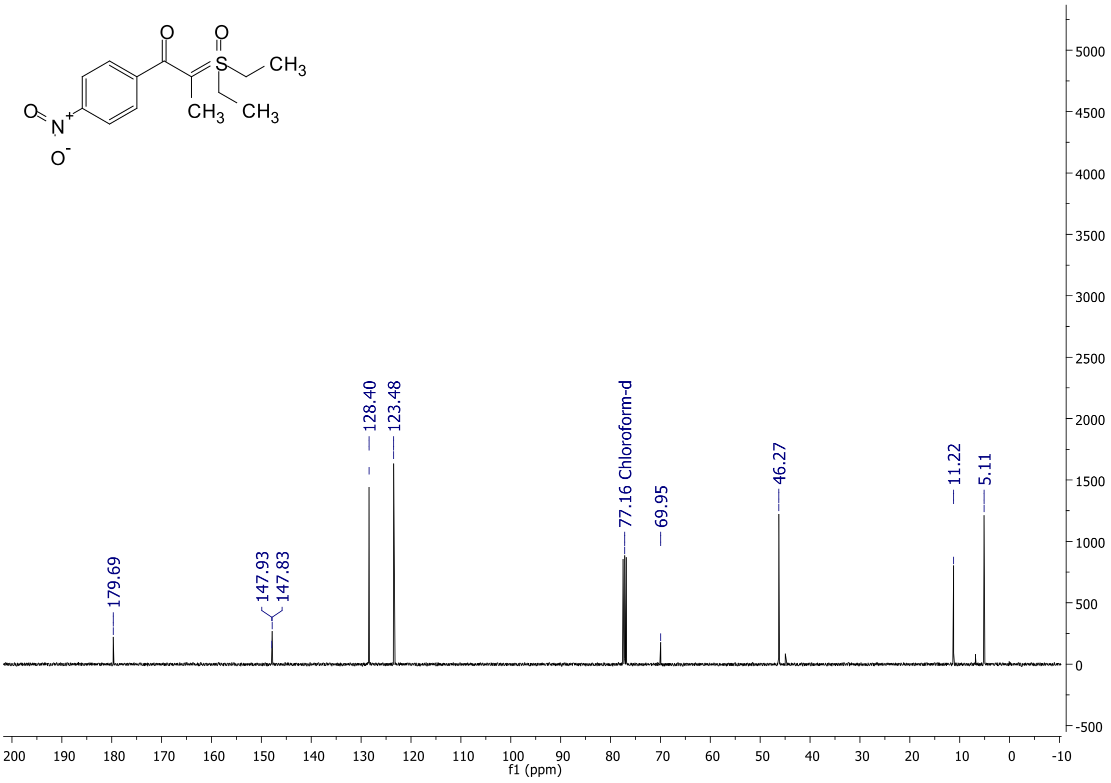

^{1}H NMR *(500 MHz, $CDCl_3$)* of Molecule **2e**:

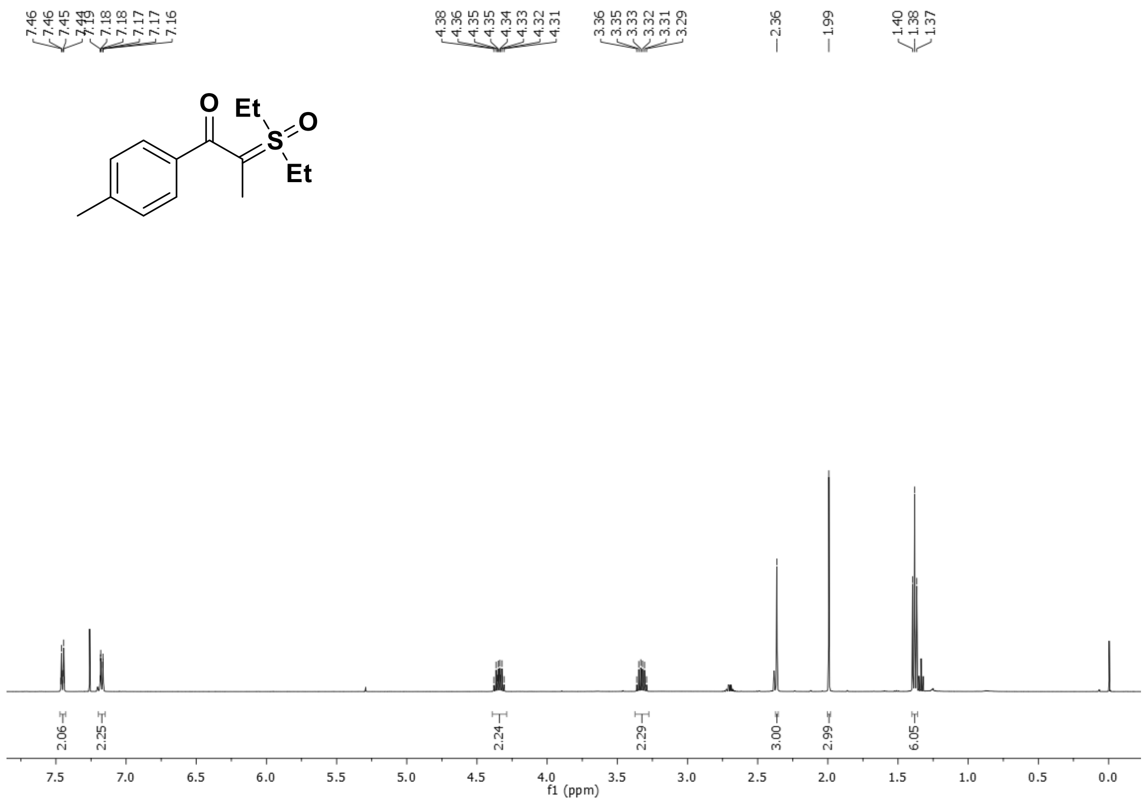

^{13}C{H} NMR *(125 MHz, $CDCl_3$)* of Molecule **2e**:

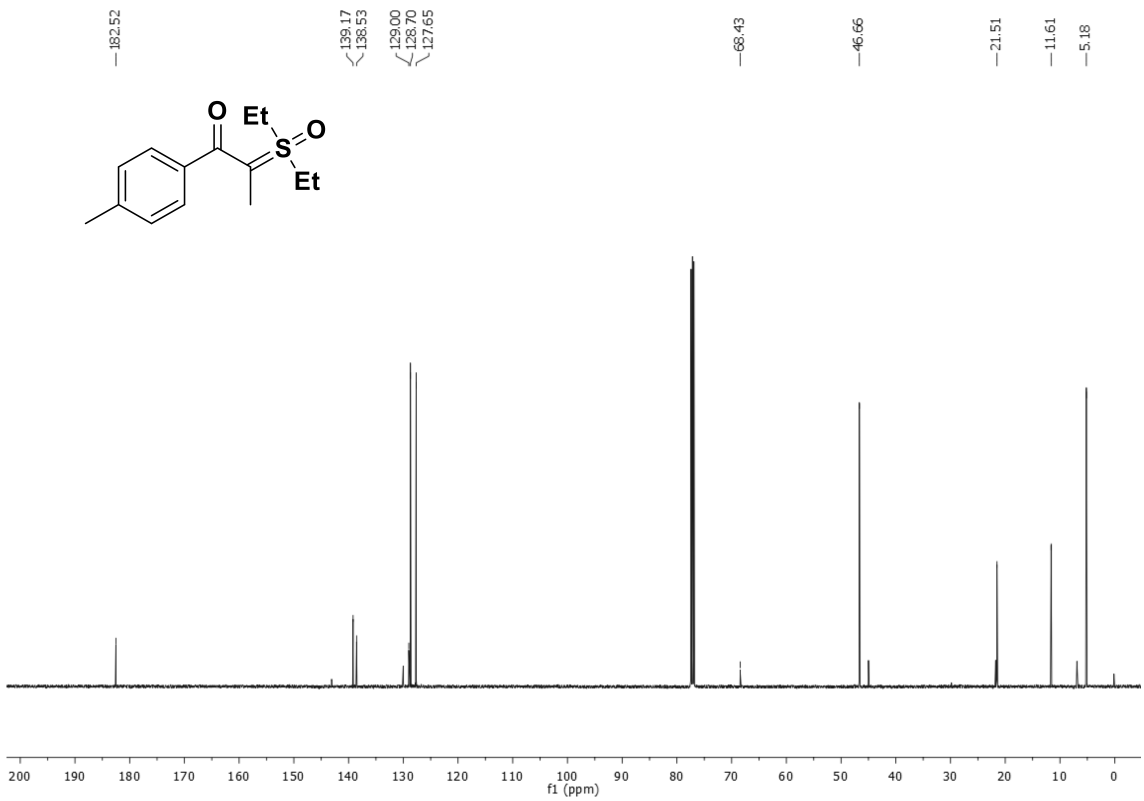

^1H NMR *(500 MHz, $CDCl_3$)* of Molecule **2f**:

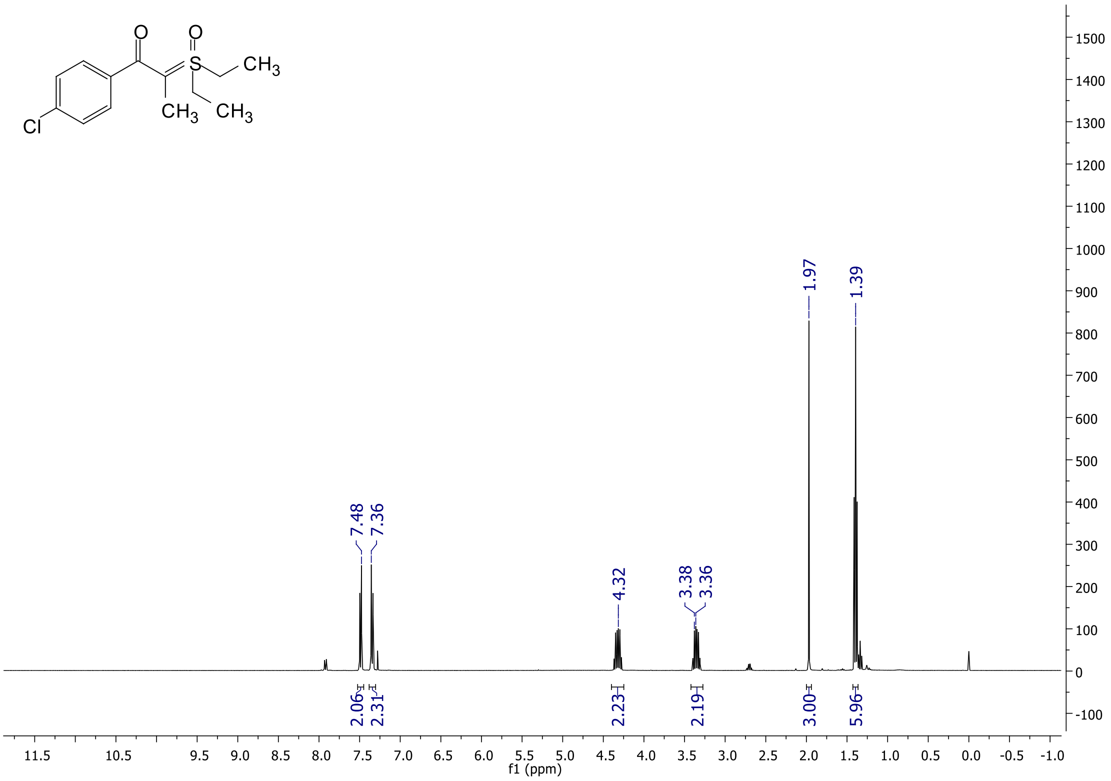

$^{13}C\{H\}$ NMR *(125 MHz, $CDCl_3$)* of Molecule **2f**:

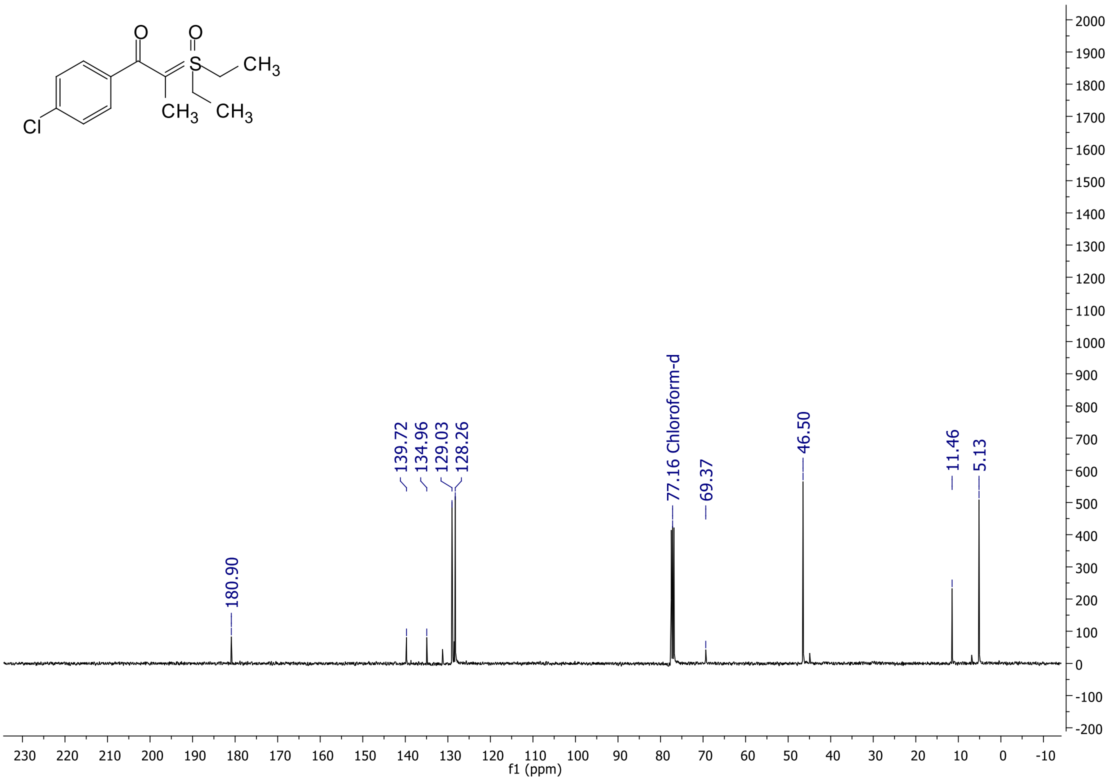

^{1}H NMR *(500 MHz, $CDCl_3$)* of Molecule **2g**:

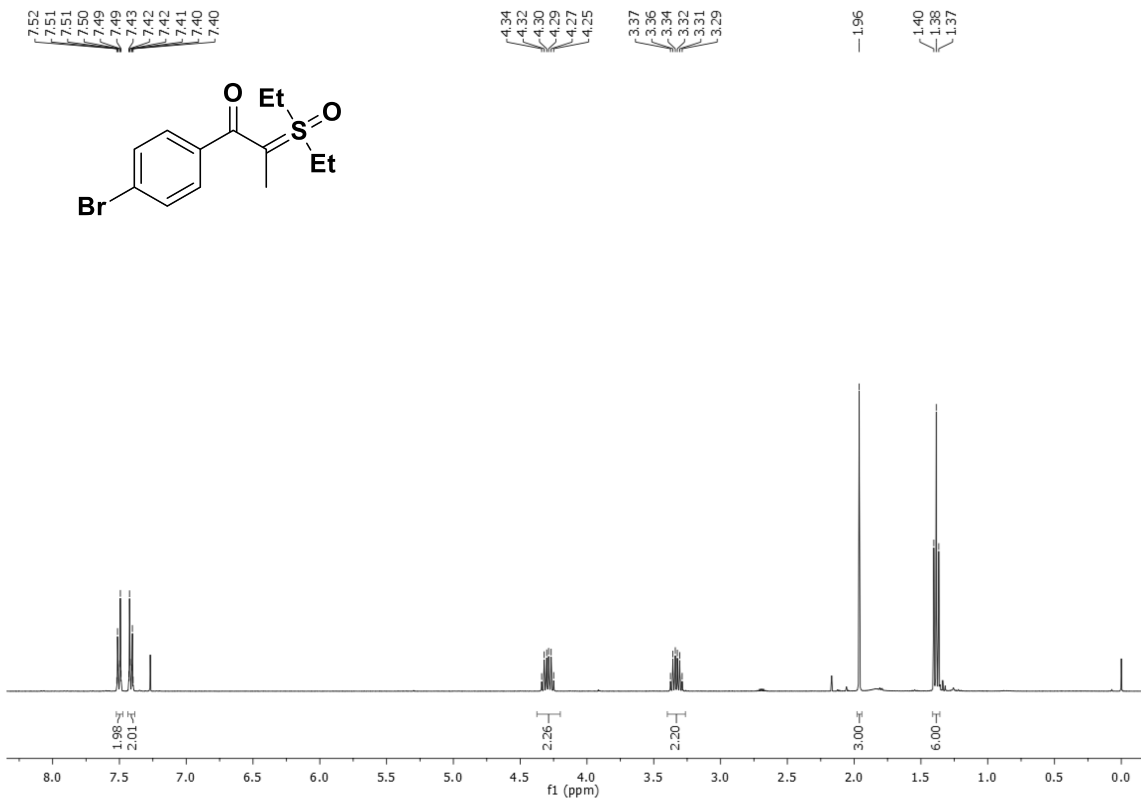

^{13}C{H} NMR *(125 MHz, $CDCl_3$)* of Molecule **2g**:

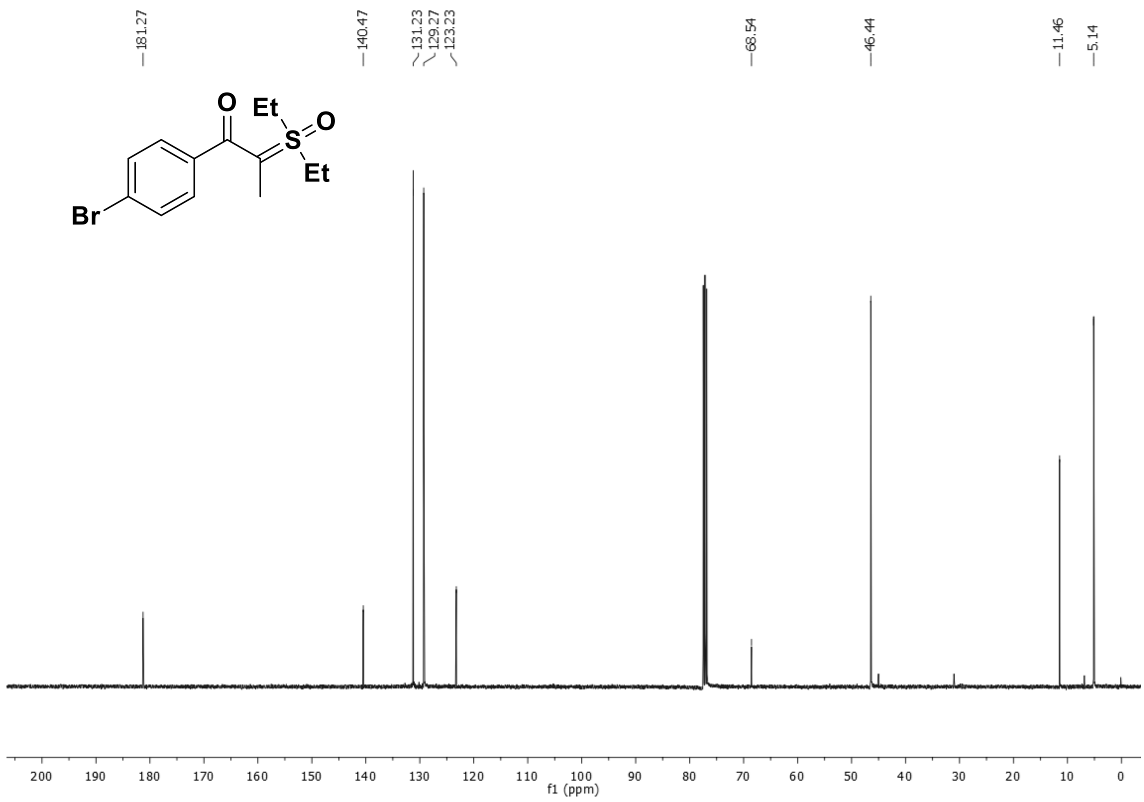

^{1}H NMR *(500 MHz, $CDCl_3$)* of Molecule **2h**:

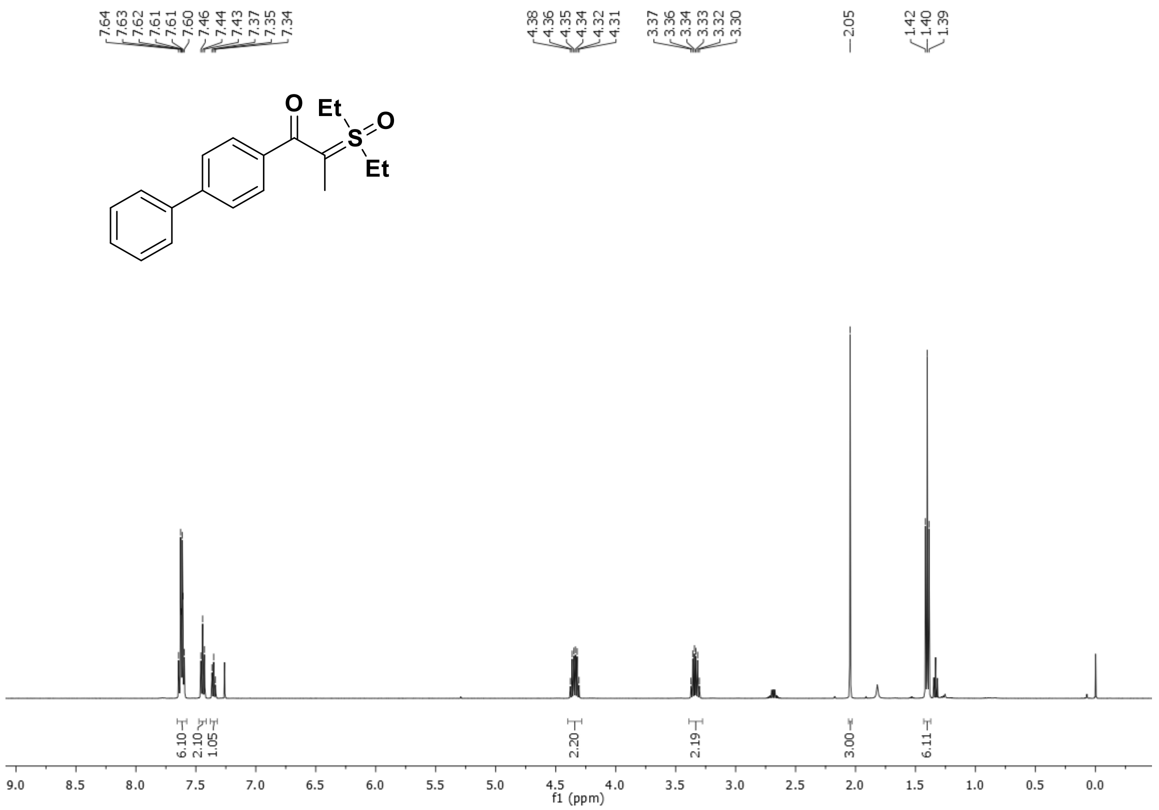

^{13}C{H} NMR *(125 MHz, $CDCl_3$)* of Molecule **2h**:

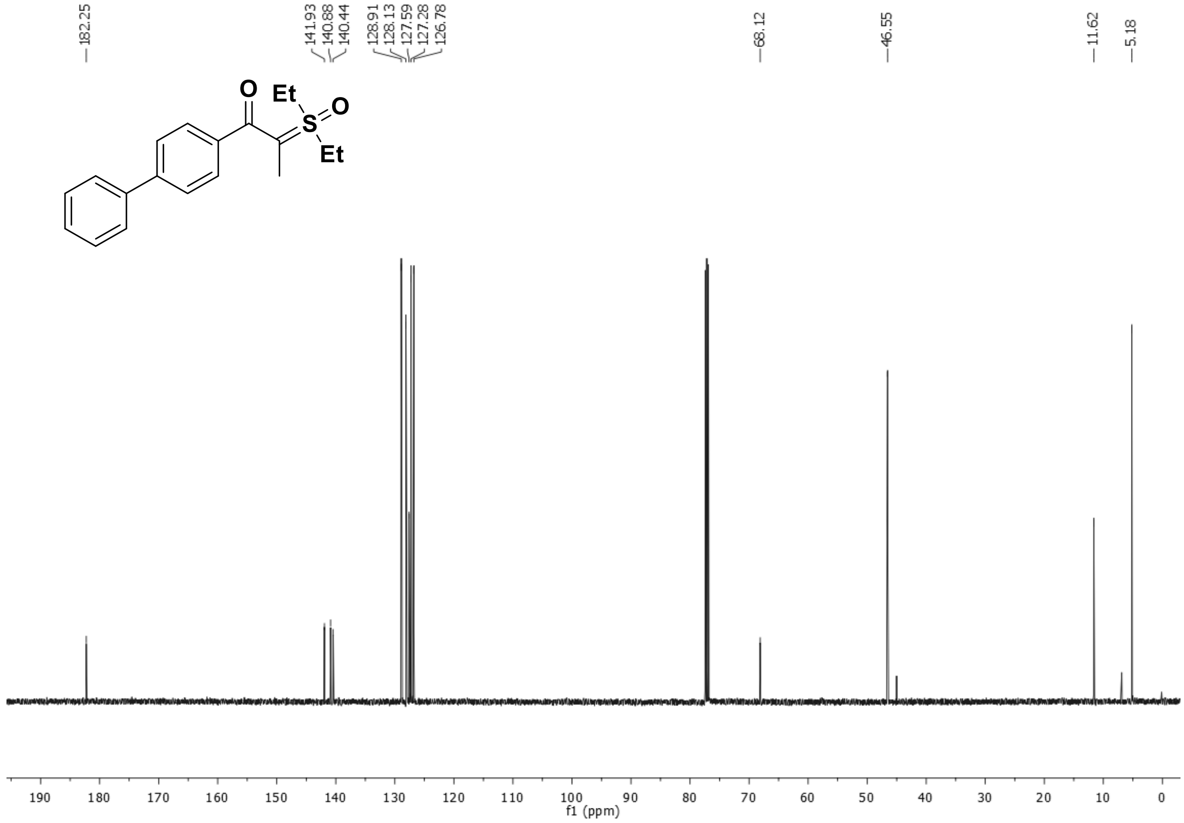

^{1}H NMR *(500 MHz, $CDCl_3$)* of Molecule **2i**:

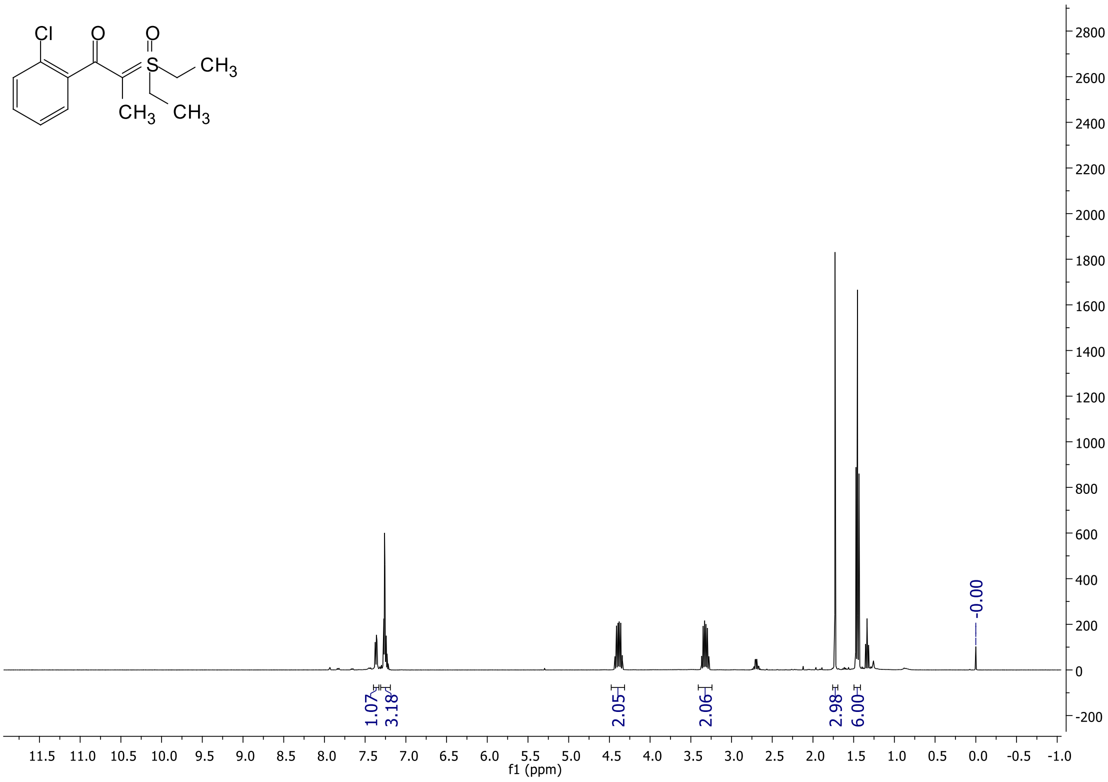

^{13}C{H} NMR *(125 MHz, $CDCl_3$)* of Molecule **2i**:

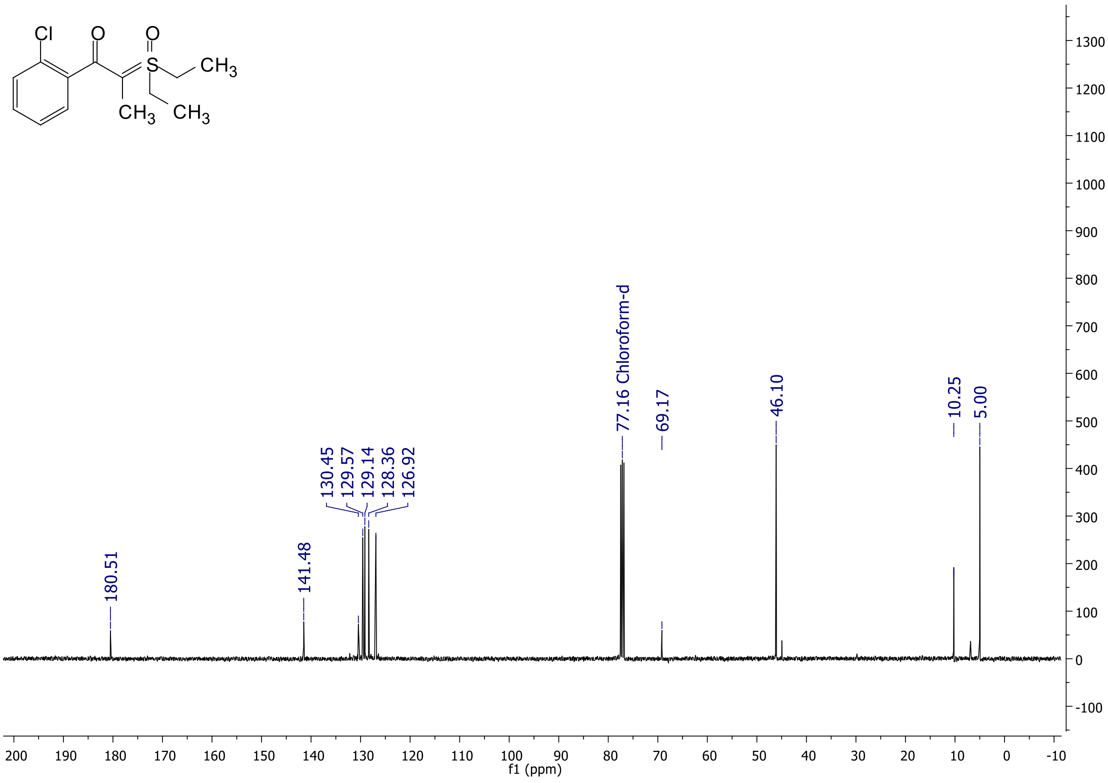

^{1}H NMR *(500 MHz, $CDCl_3$)* of Molecule **2j**:

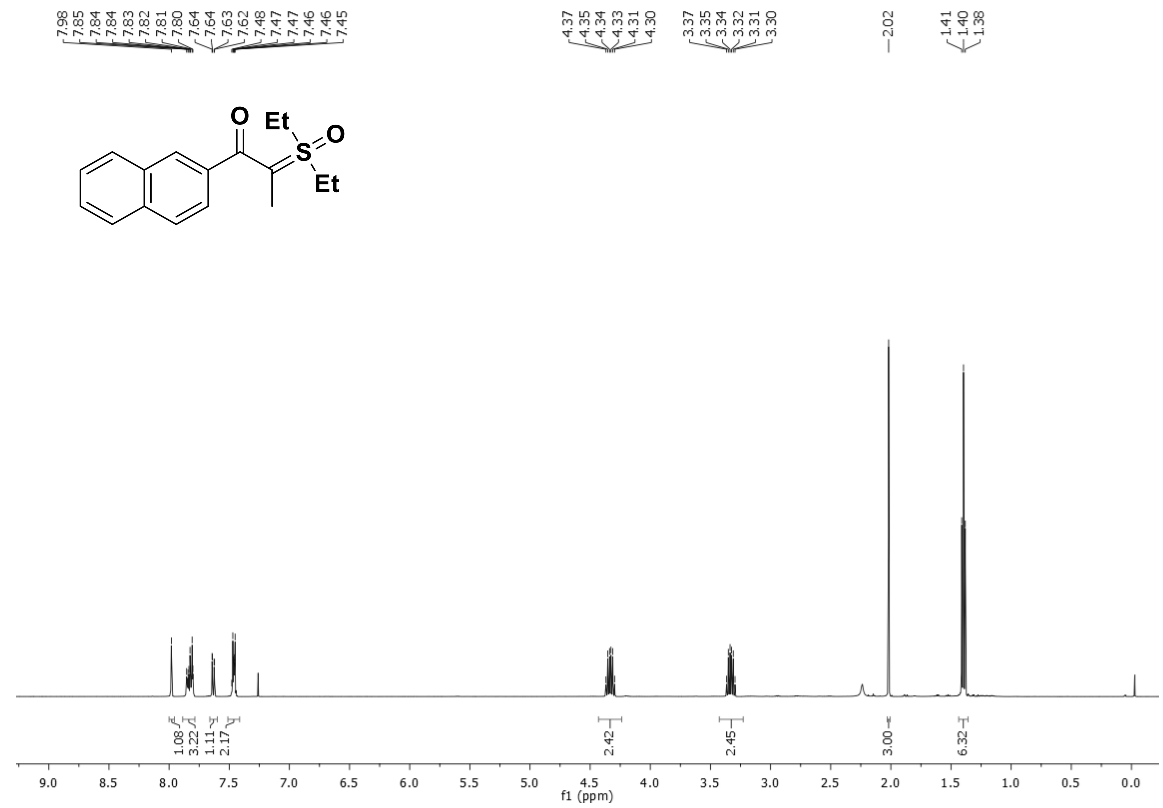

^{13}C{H} NMR *(125 MHz, $CDCl_3$)* of Molecule **2j**:

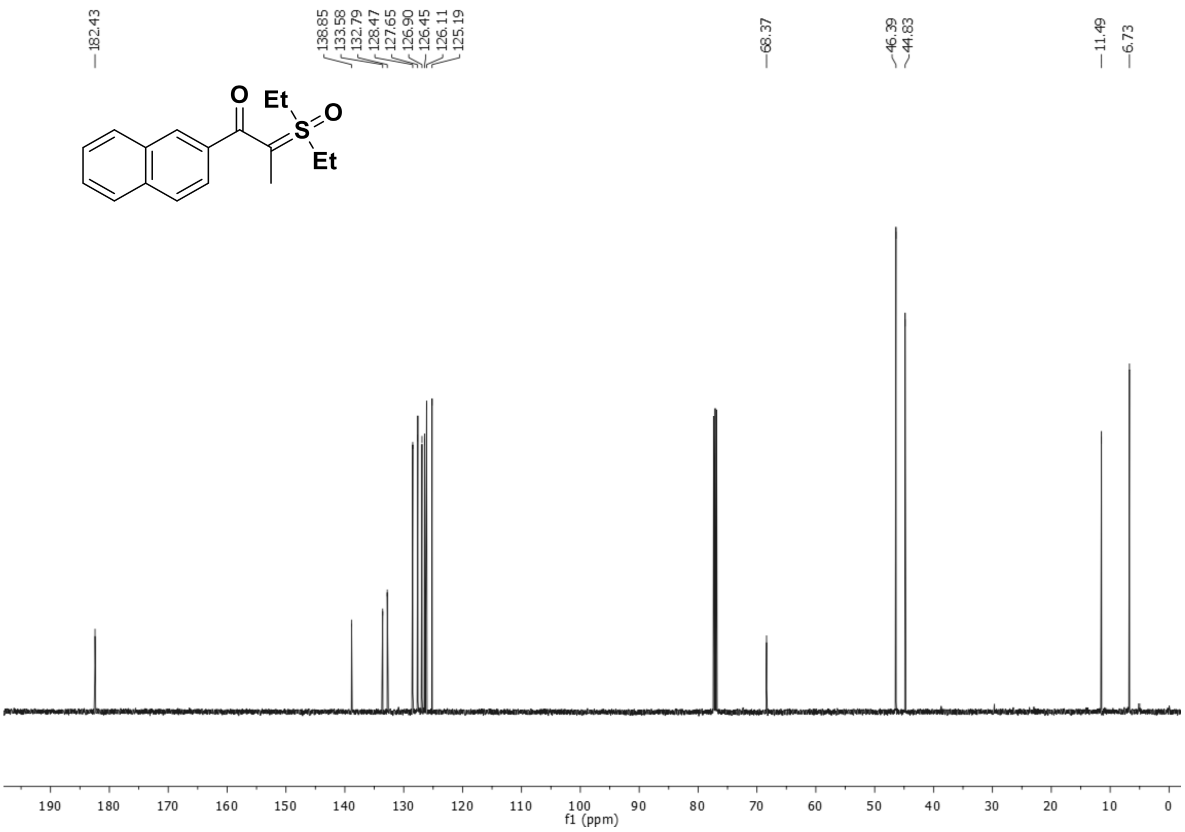

^{1}H NMR *(500 MHz, $CDCl_3$)* of Molecule **2’d**:

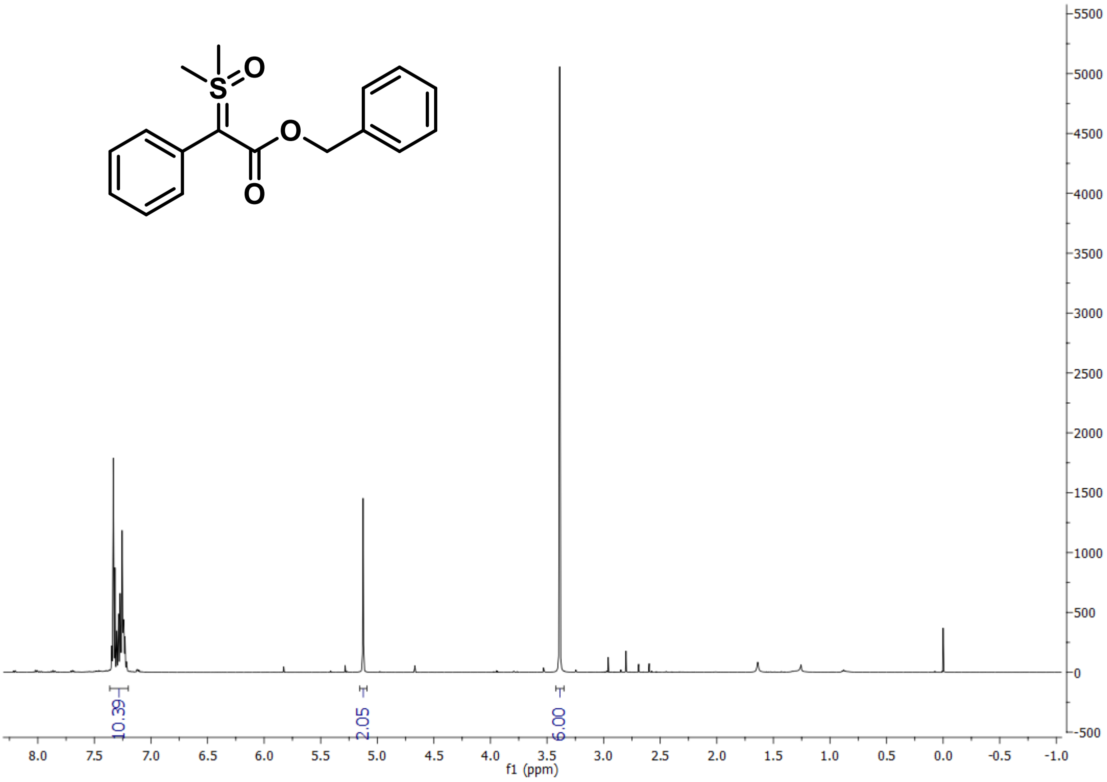

^{13}C{H} NMR *(100 MHz, $CDCl_3$)* of Molecule **2’d**:

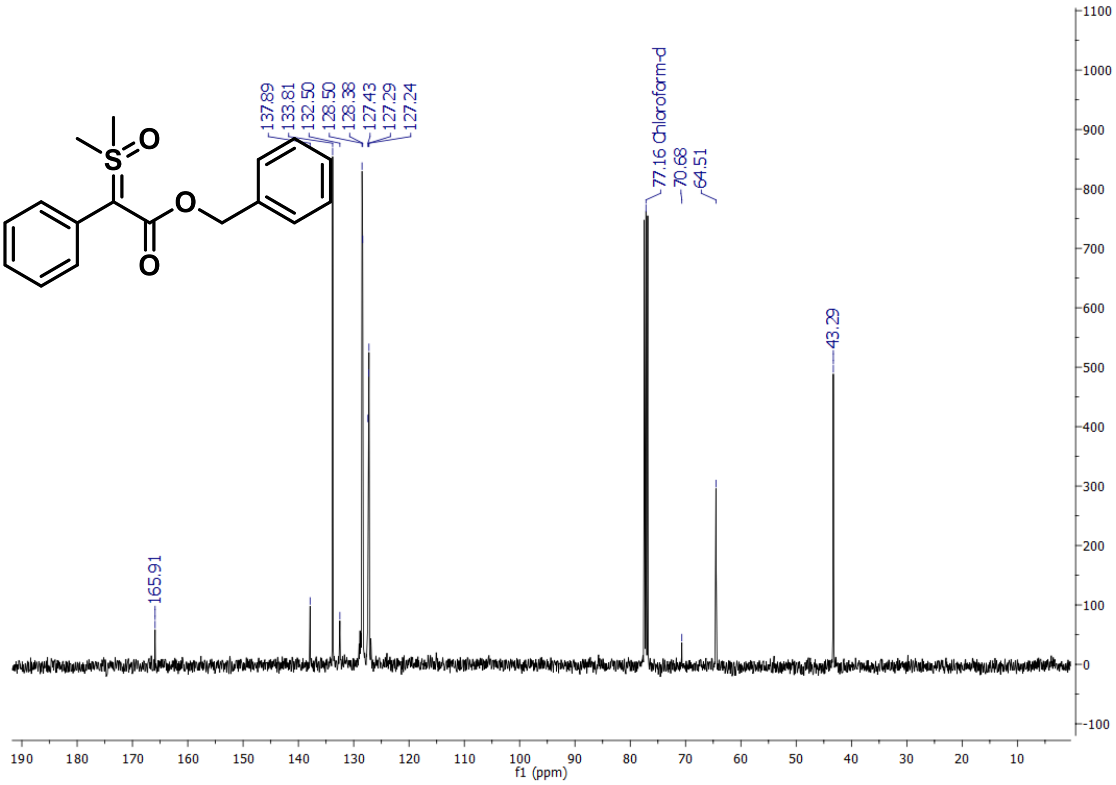

^{1}H NMR *(500 MHz, $CDCl_3$)* of Molecule **2’i**:

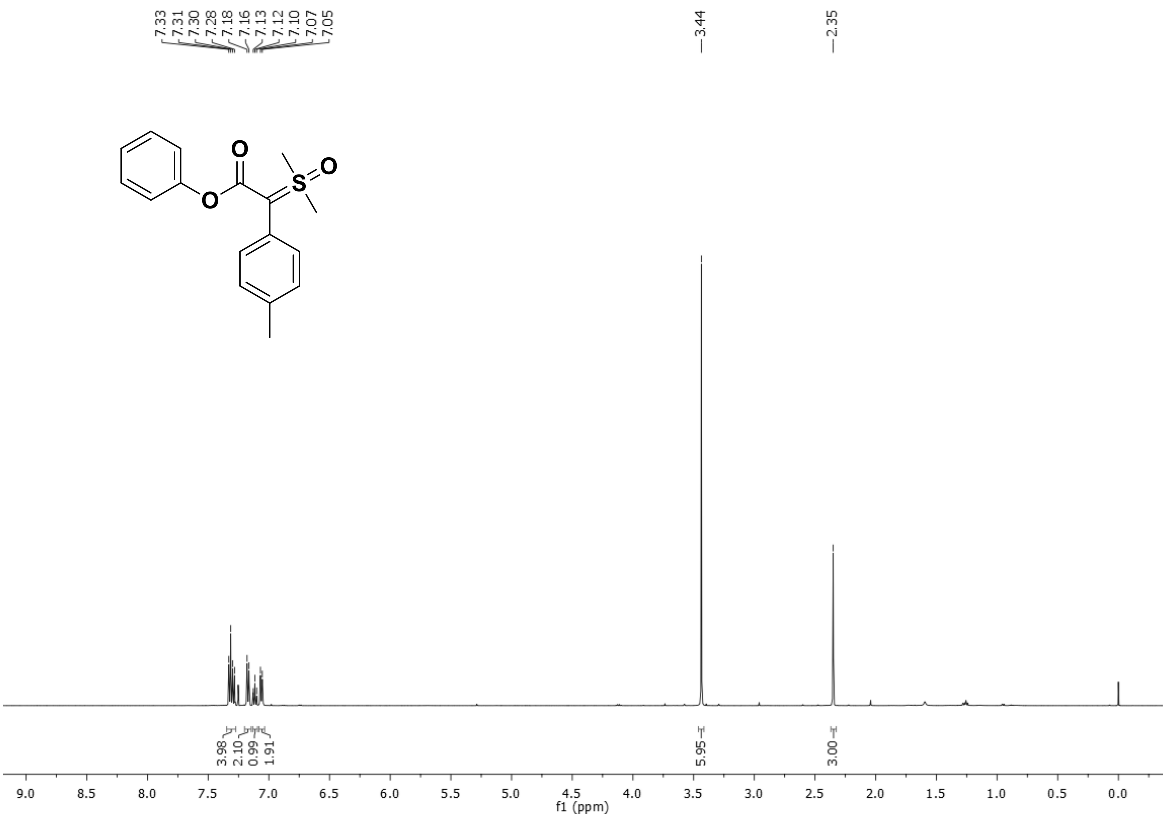

$^{13}C\{H\}$ NMR *(125 MHz, $CDCl_3$)* of Molecule **2’i**:

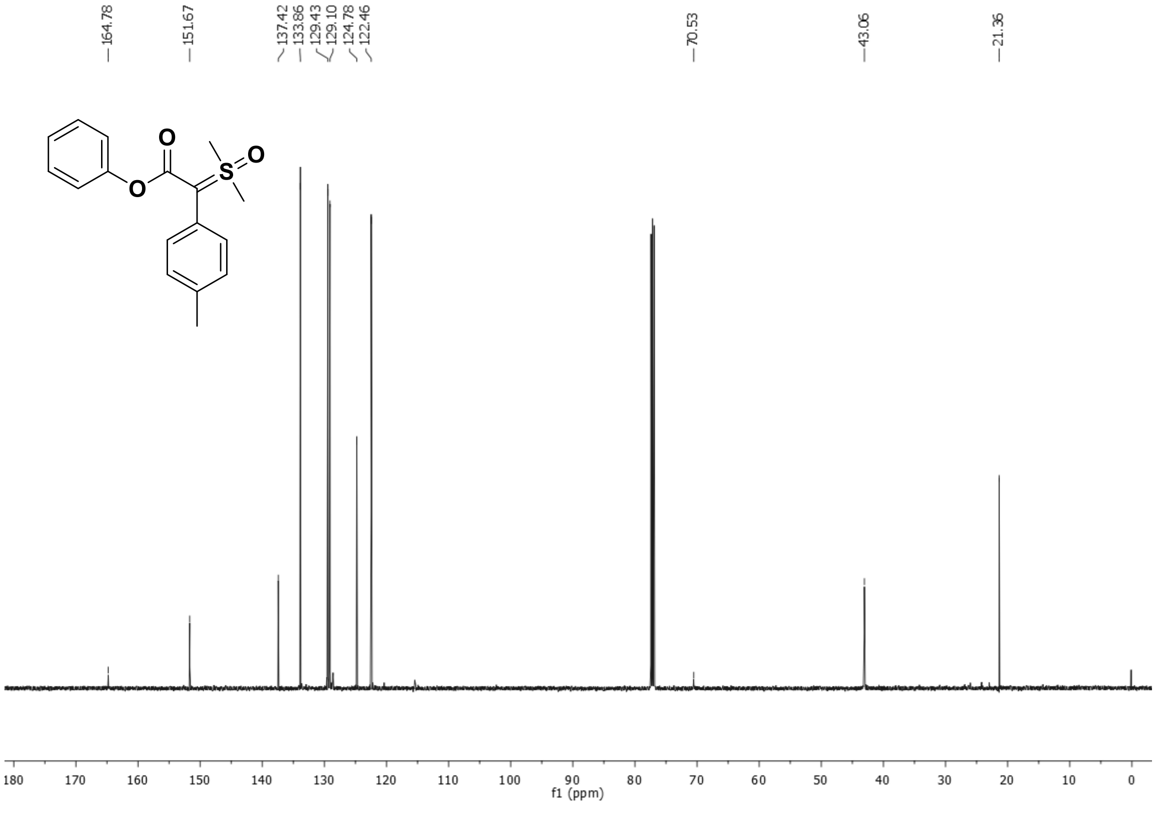

^{1}H NMR *(500 MHz, $CDCl_3$)* of Molecule **2’j**:

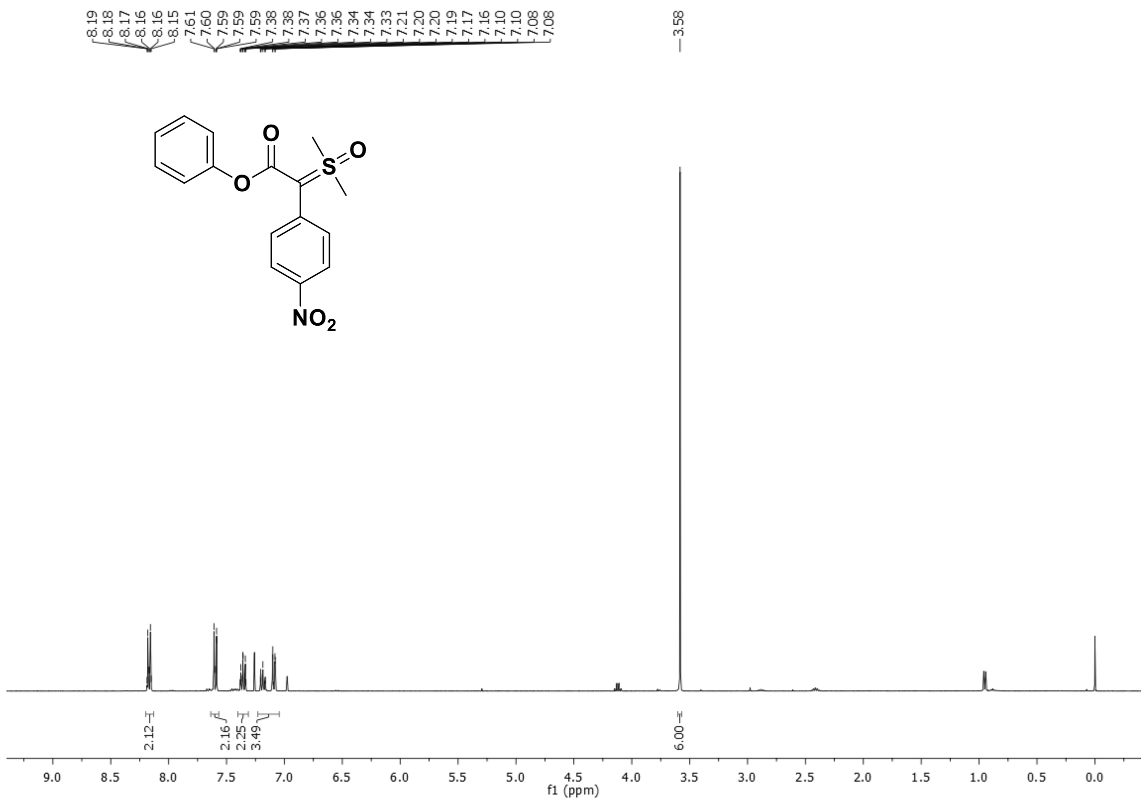

^{13}C{H} NMR *(125 MHz, $CDCl_3$)* of Molecule **2’j**:

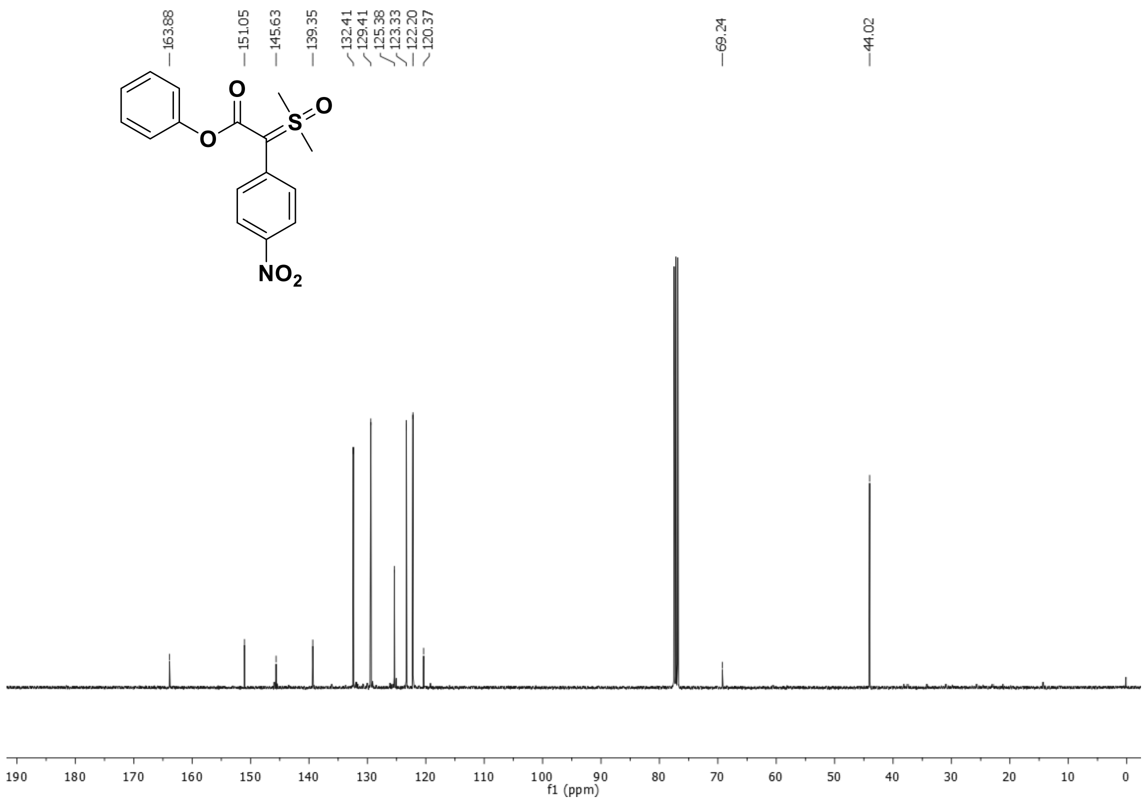

^{1}H NMR *(400 MHz, $CDCl_3$)* of Molecule **3a**:

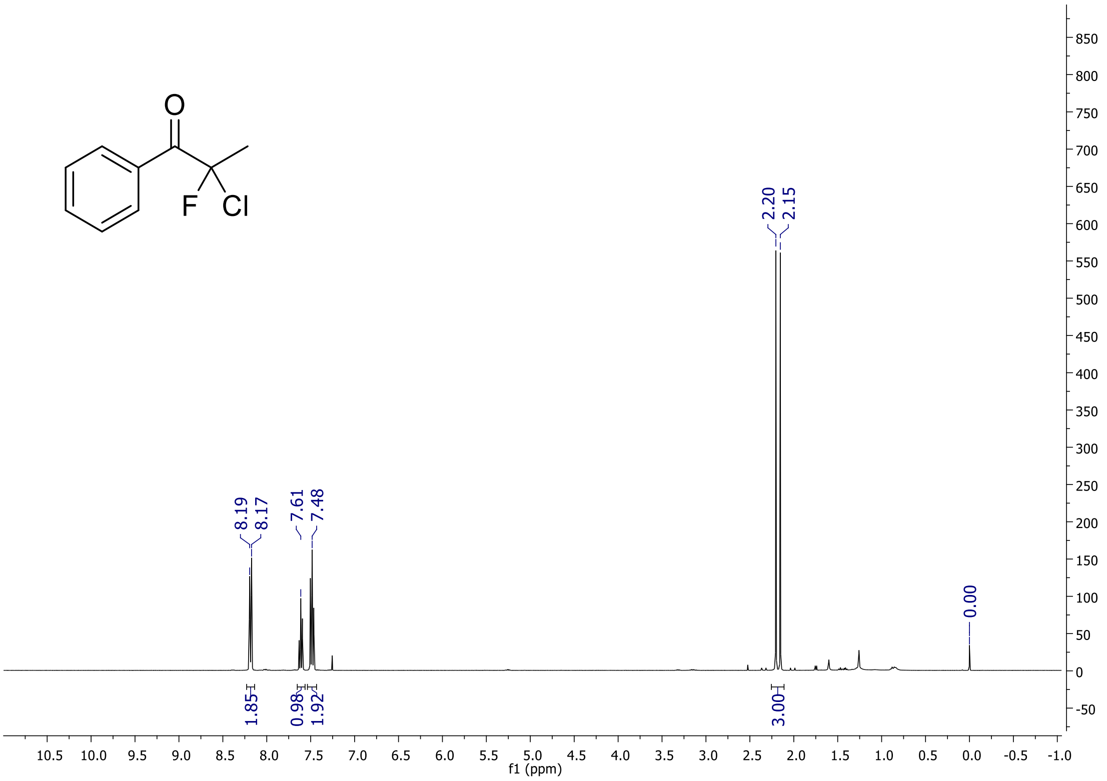

$^{13}C\{H\}$ NMR *(100 MHz, $CDCl_3$)* of Molecule **3a**:

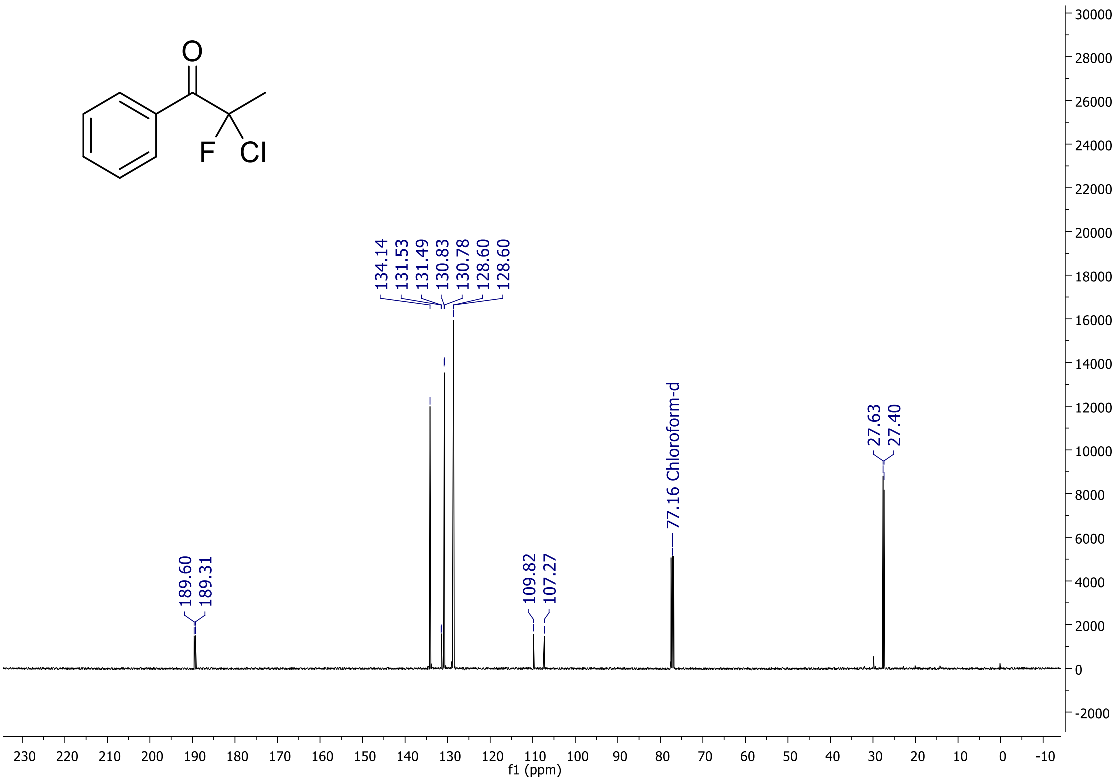

^{19}F NMR (470 MHz, $CDCl_3$) of Molecule **3a**:

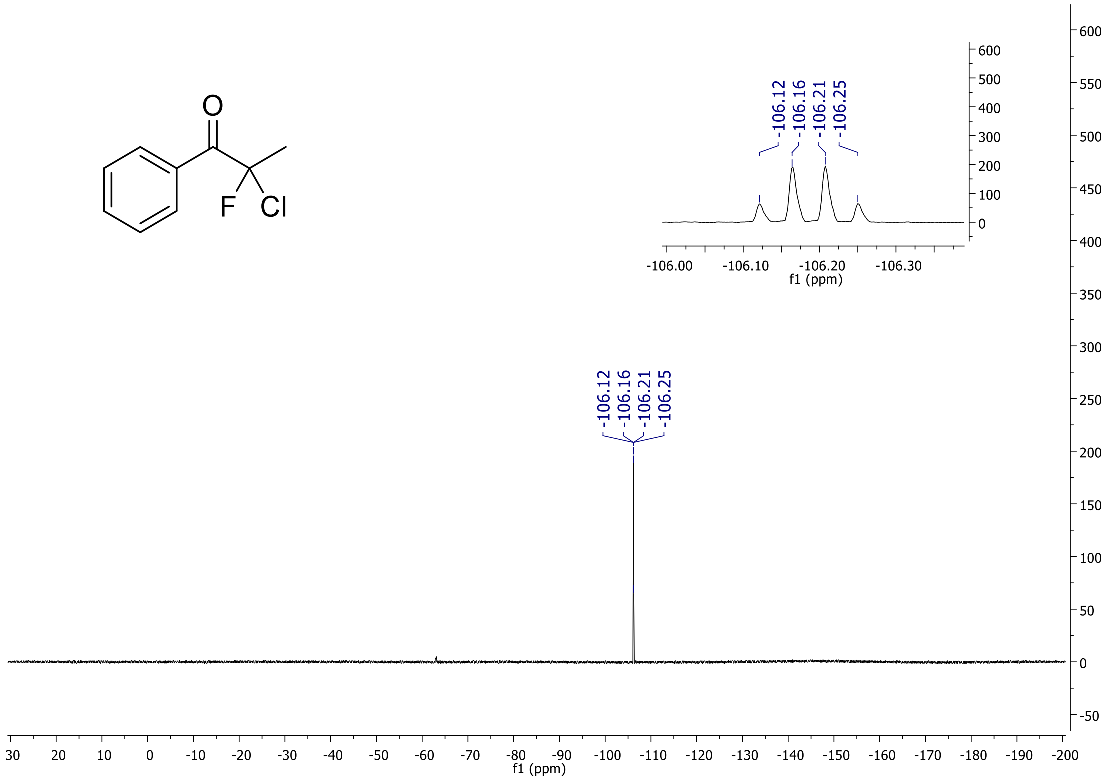

^{1}H NMR *(500 MHz, $CDCl_3$)* of Molecule **3b**:

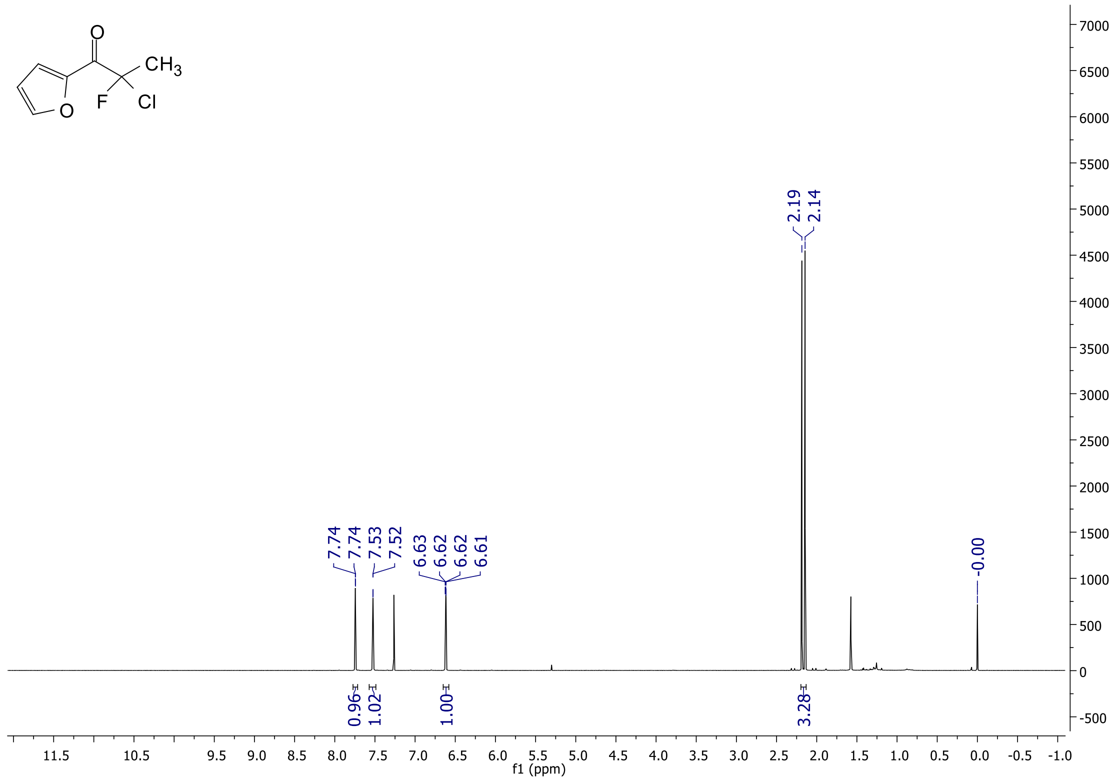

$^{13}C\{H\}$ NMR *(125 MHz, $CDCl_3$)* of Molecule **3b**:

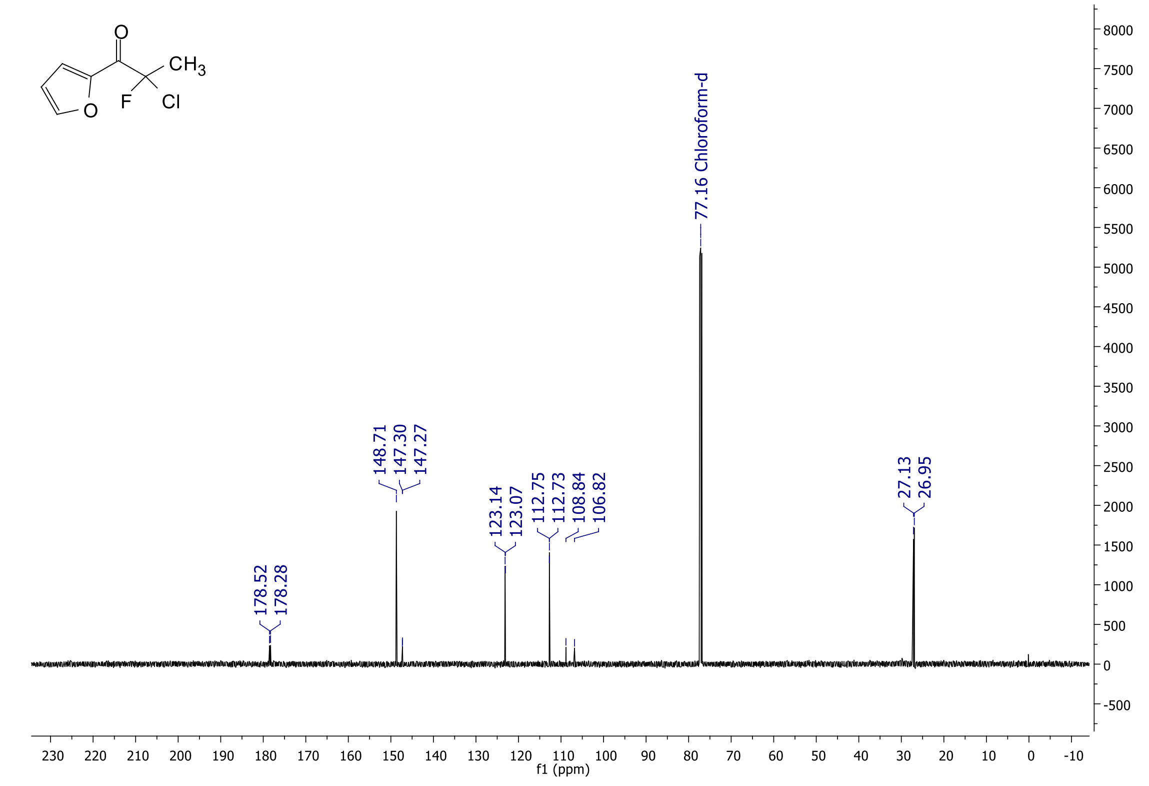

^{19}F NMR (376 MHz, $CDCl_3$) of Molecule **3b**:

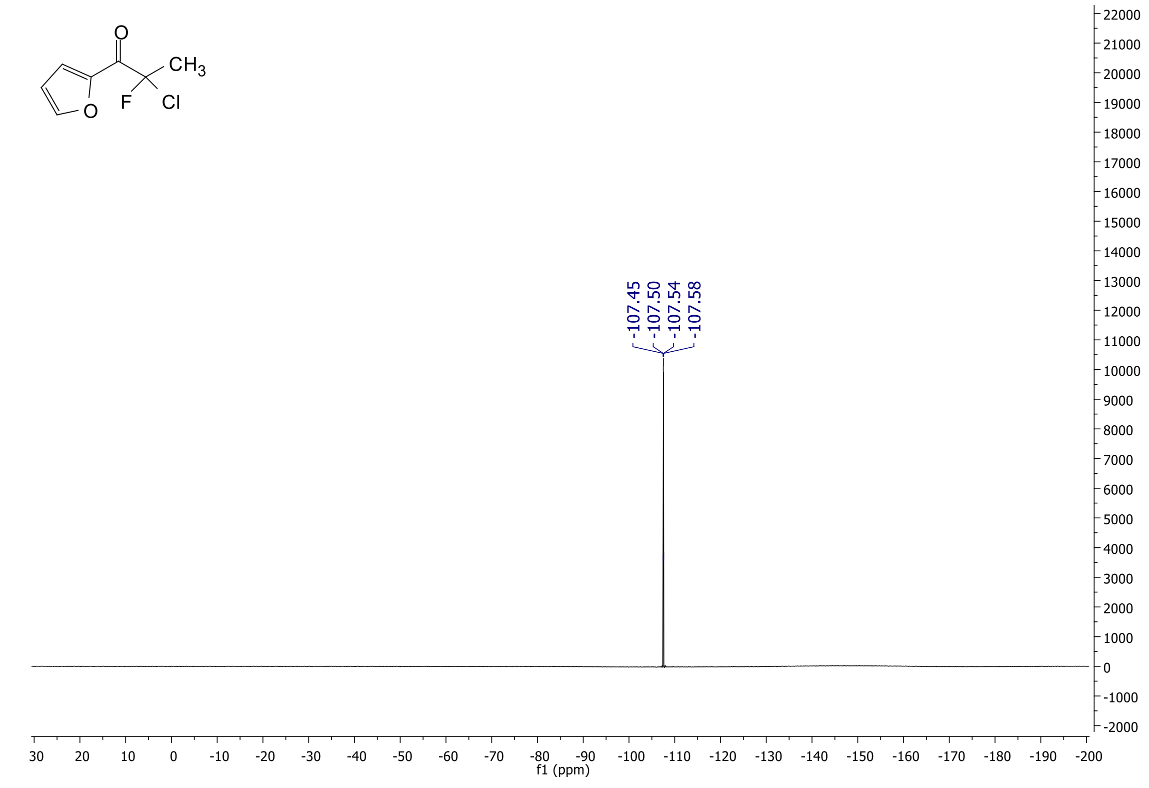

^{1}H NMR *(500 MHz, $CDCl_3$)* of Molecule **3c**:

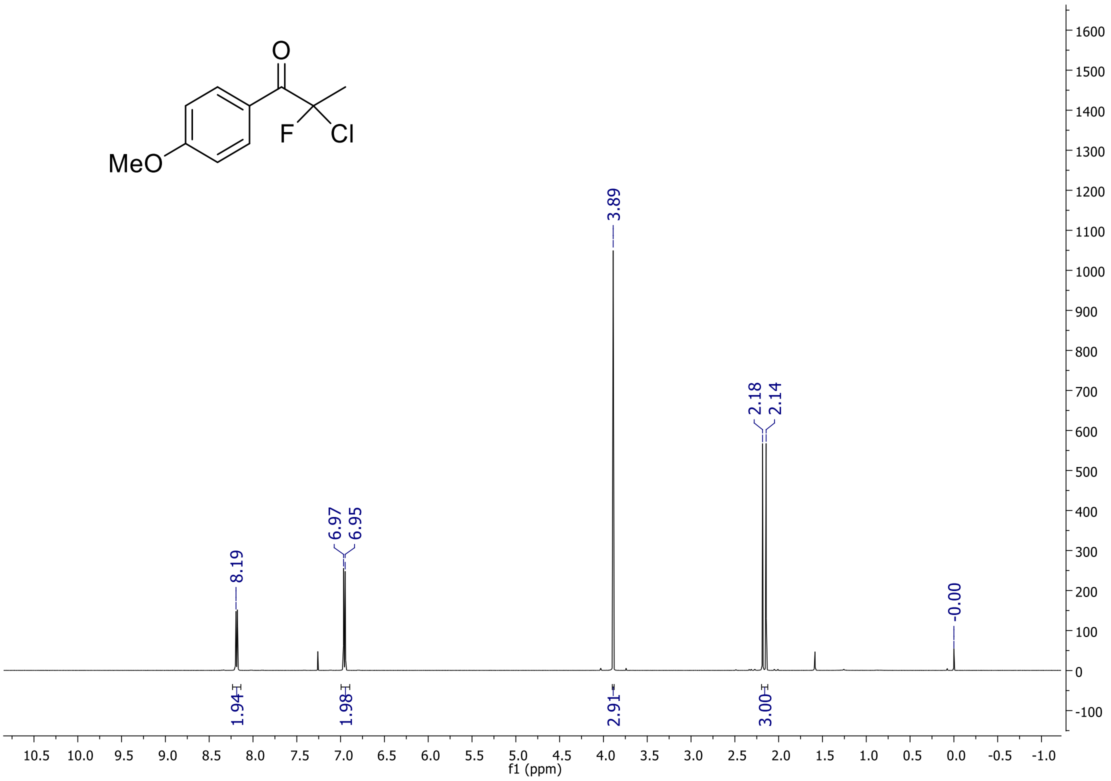

$^{13}C\{H\}$ NMR *(125 MHz, $CDCl_3$)* of Molecule **3c**:

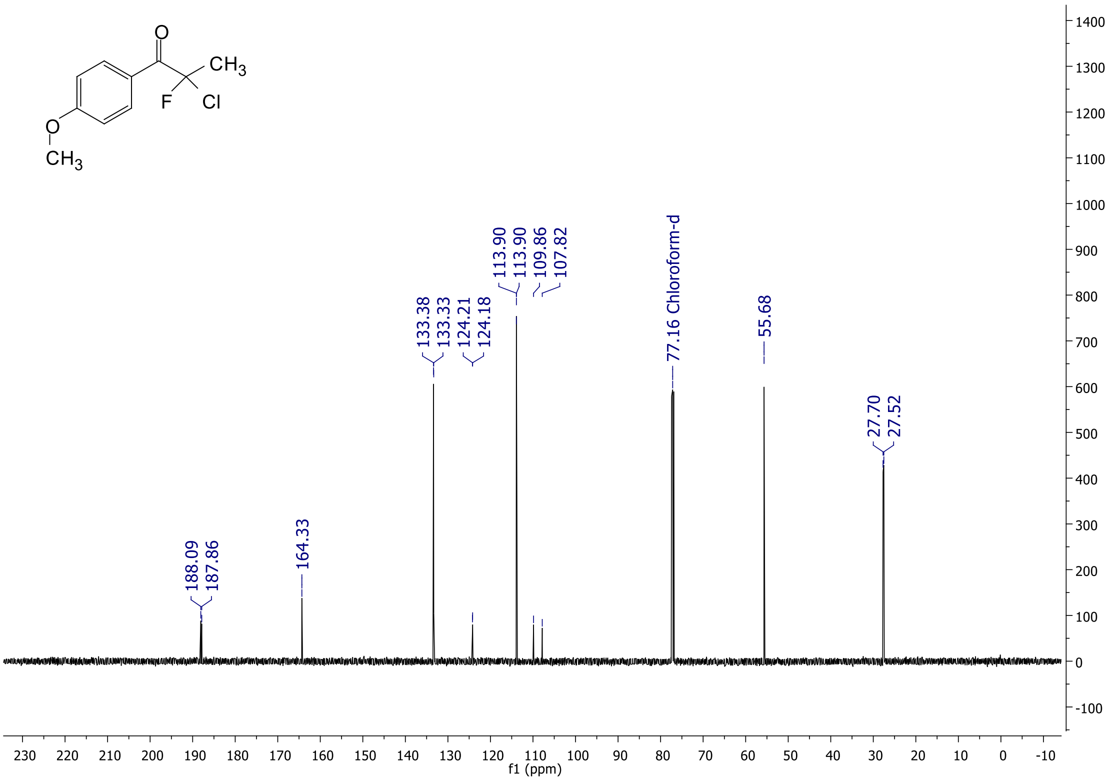

^{19}F NMR (470 MHz, $CDCl_3$) of Molecule **3c**:

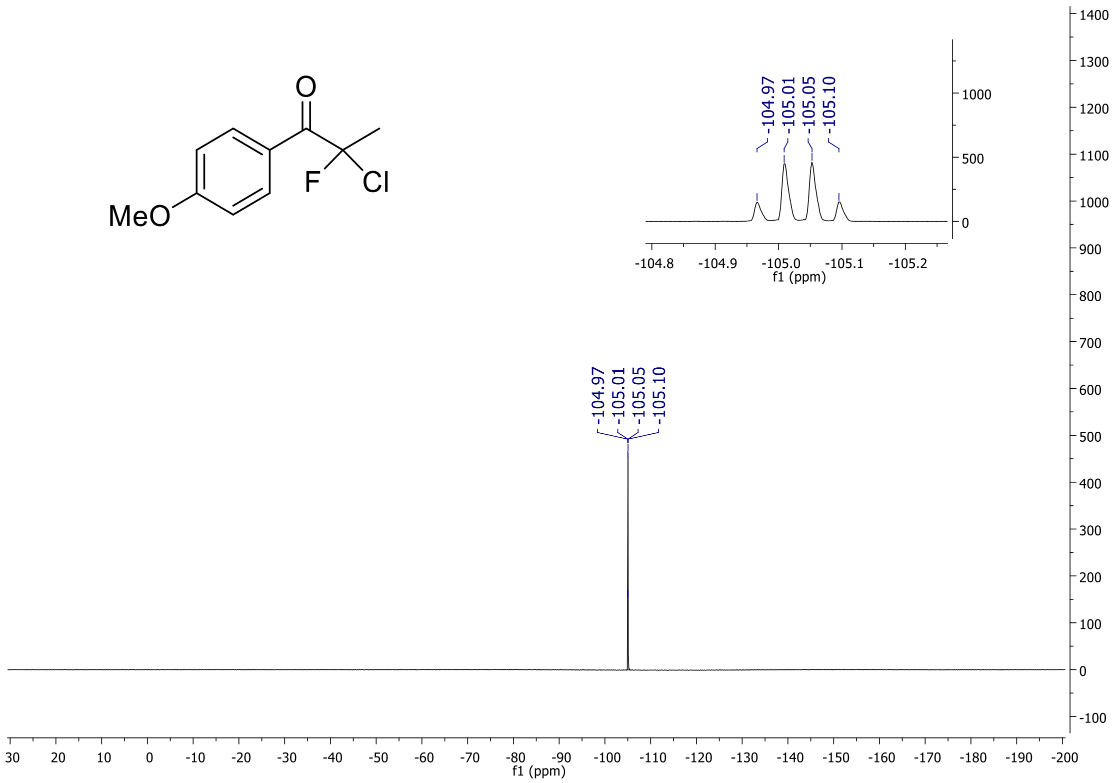

^{1}H NMR *(500 MHz, $CDCl_3$)* of Molecule **3d**:

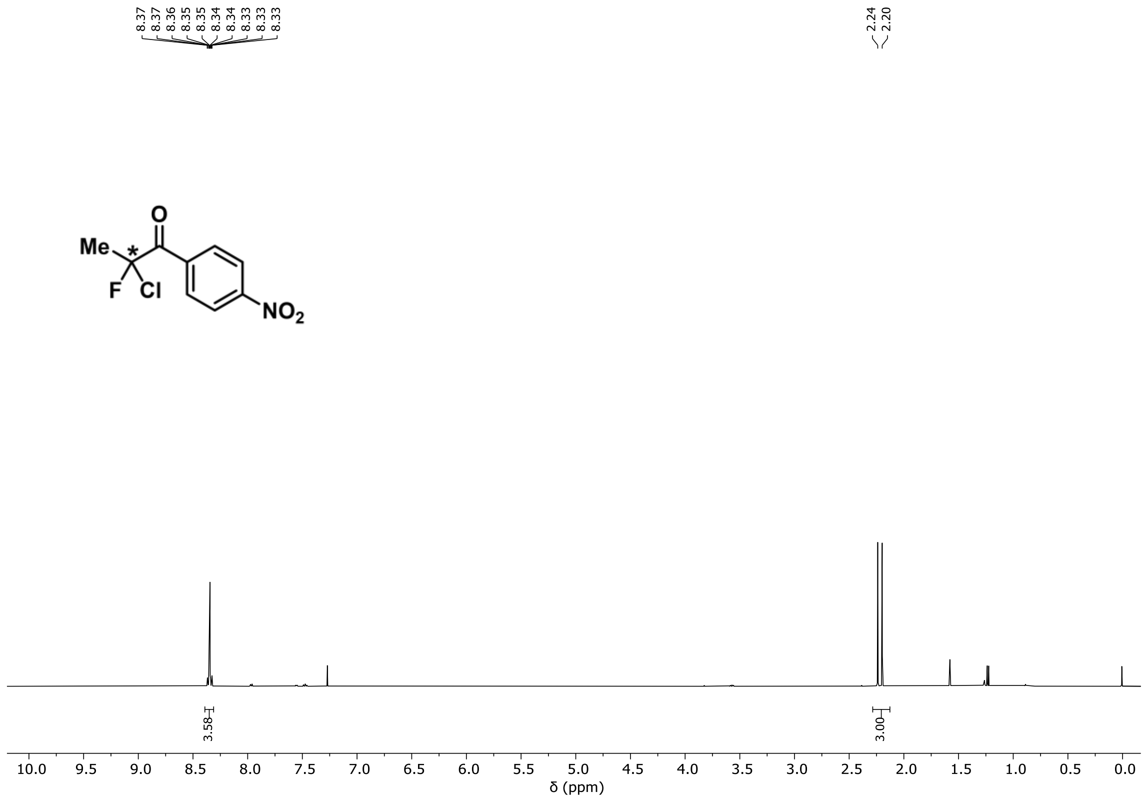

^{13}C{H} NMR *(125 MHz, $CDCl_3$)* of Molecule **3d**:

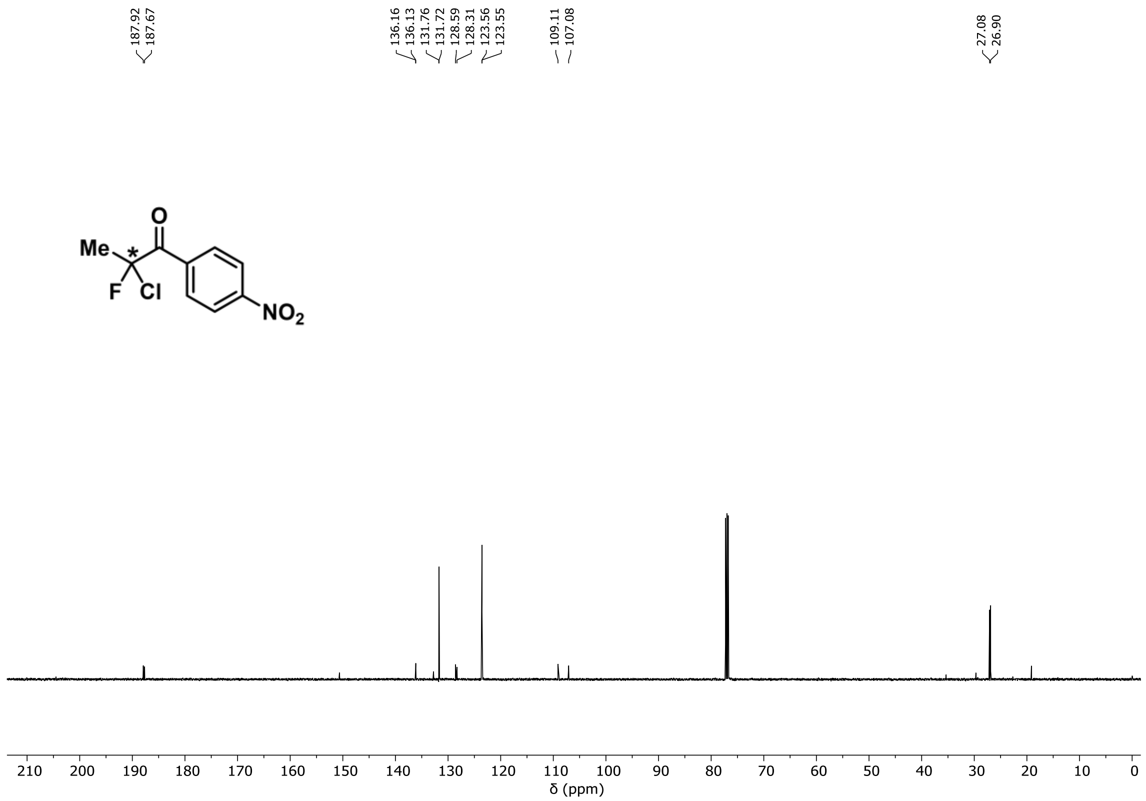

^{19}F NMR (376 MHz, $CDCl_3$) of Molecule **3d**:

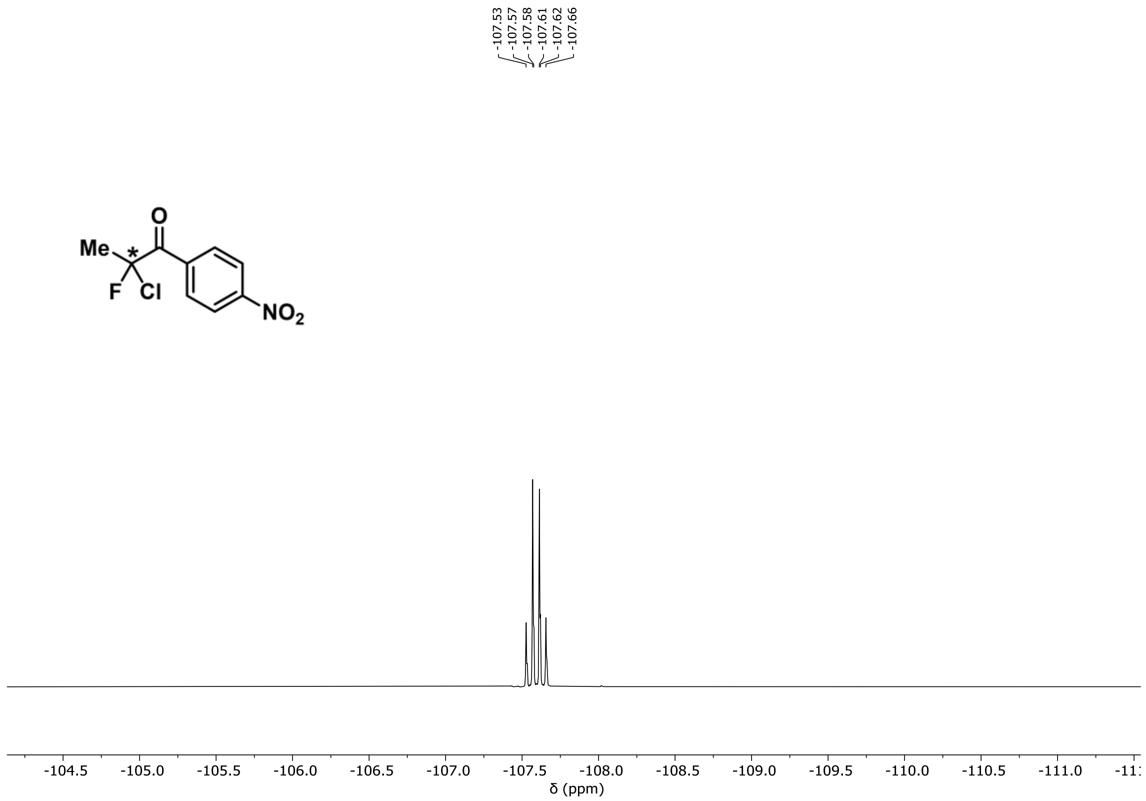

^{1}H NMR *(500 MHz, $CDCl_3$)* of Molecule **3e**:

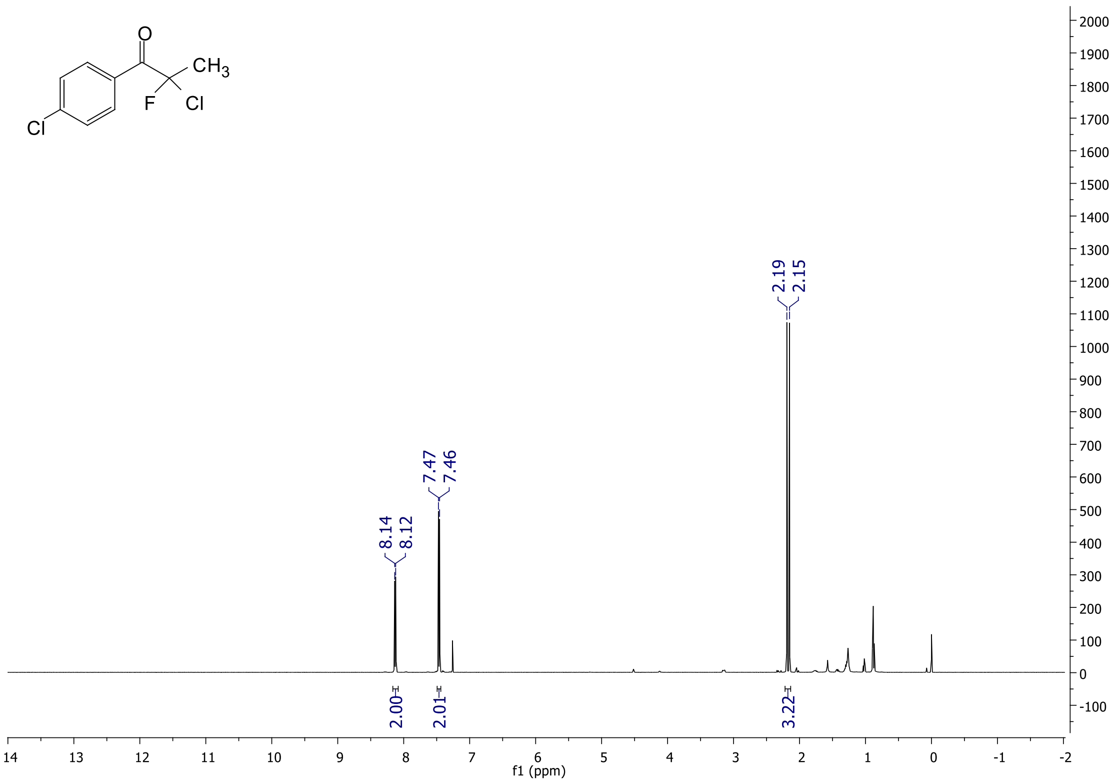

$^{13}C\{H\}$ NMR *(125 MHz, $CDCl_3$)* of Molecule **3e**:

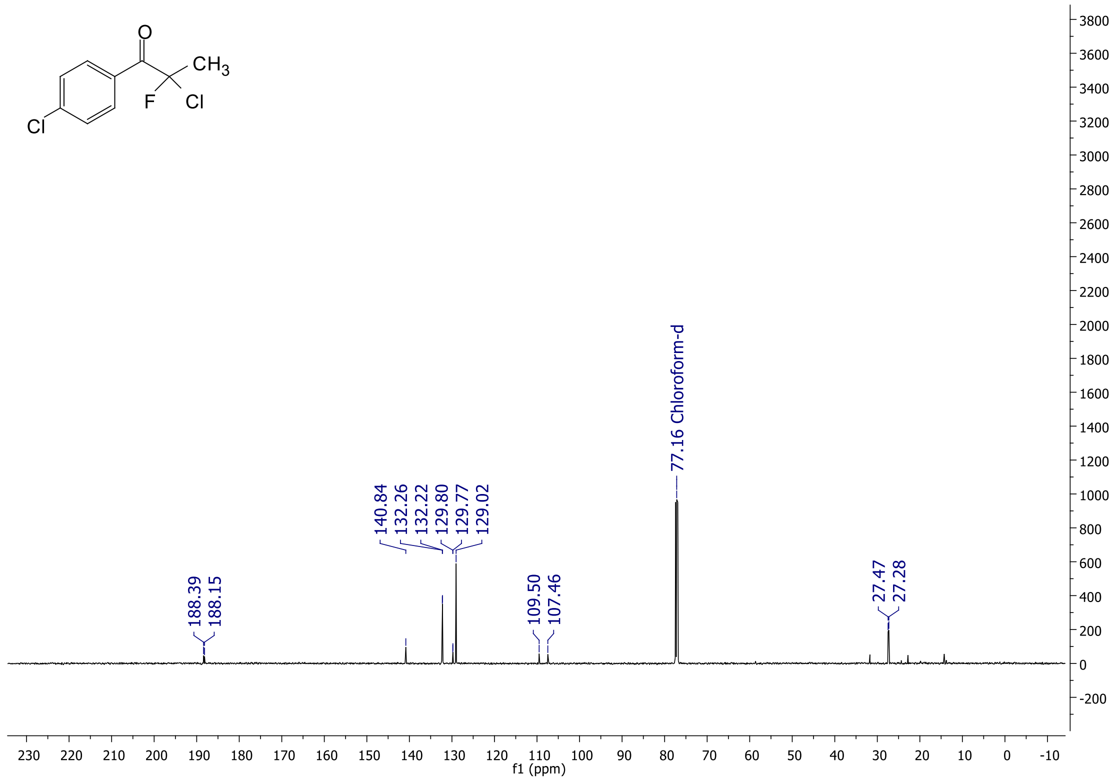

^{19}F NMR (376 MHz, $CDCl_3$) of Molecule **3e**:

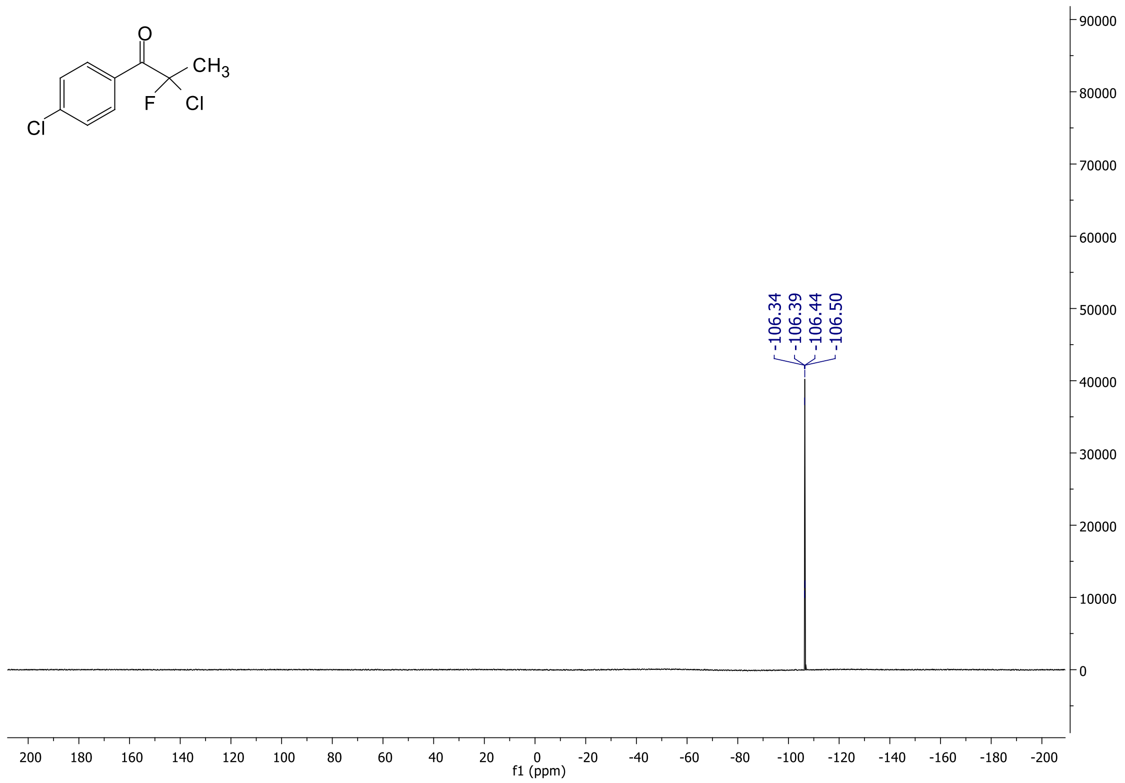

^{1}H NMR *(500 MHz, $CDCl_3$)* of Molecule **3f**:

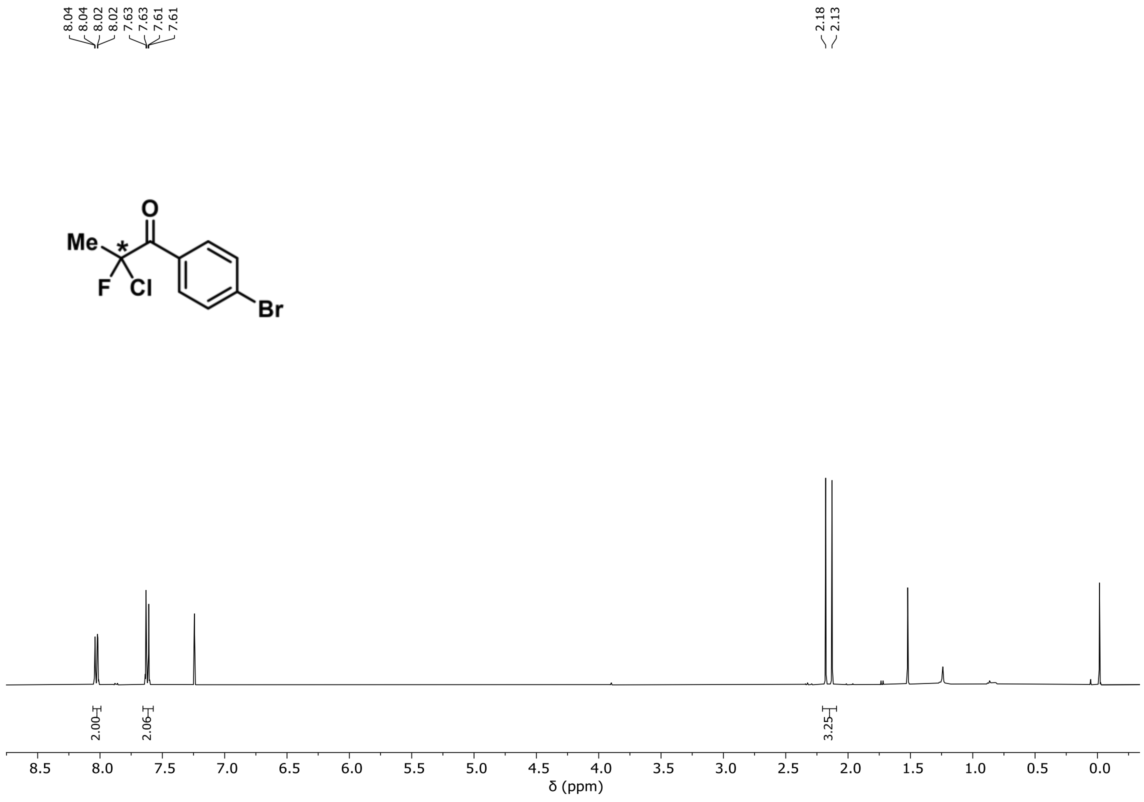

$^{13}C\{H\}$ NMR *(125 MHz, $CDCl_3$)* of Molecule **3f**:

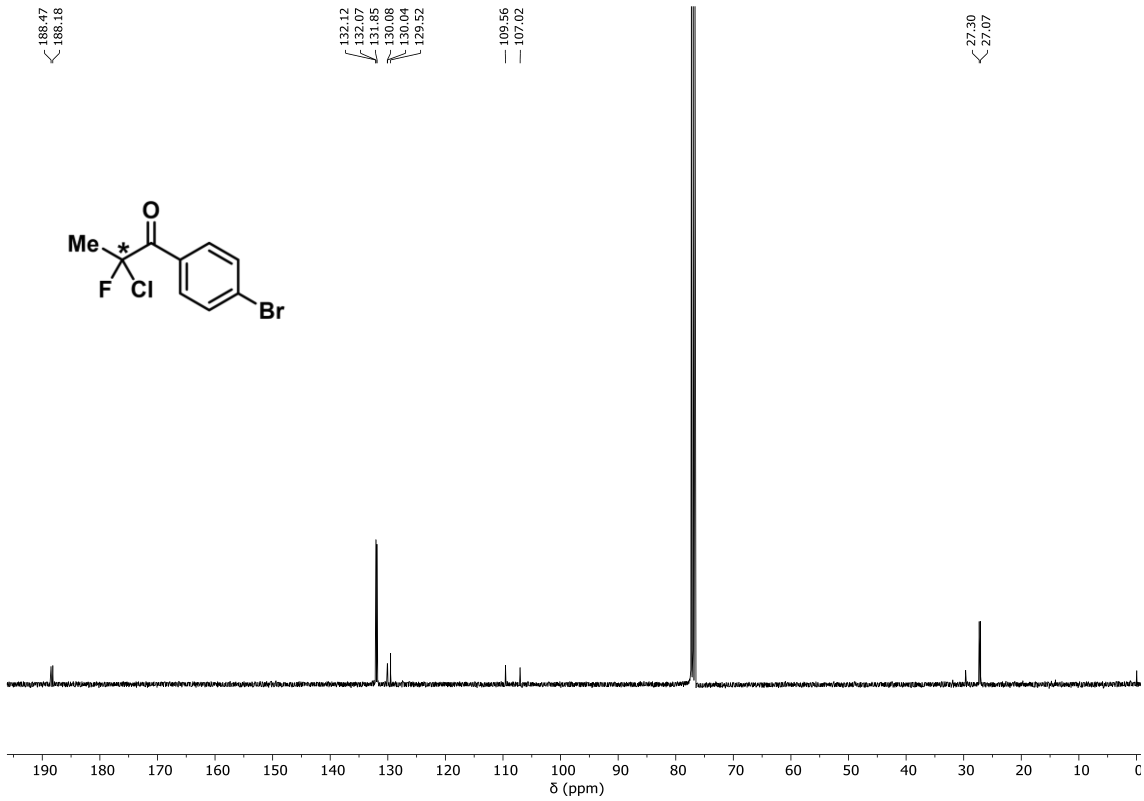

^{19}F NMR (376 MHz, $CDCl_3$) of Molecule **3f**:

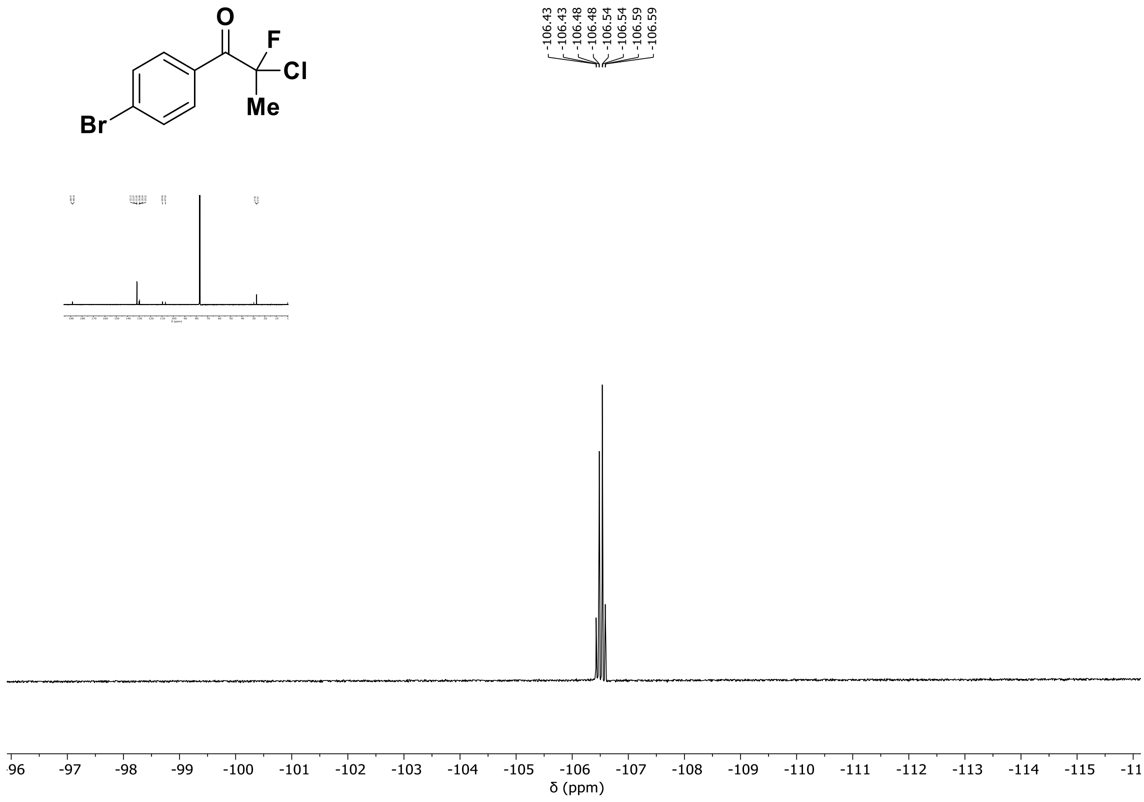

^{1}H NMR *(500 MHz, $CDCl_3$)* of Molecule **3g**:

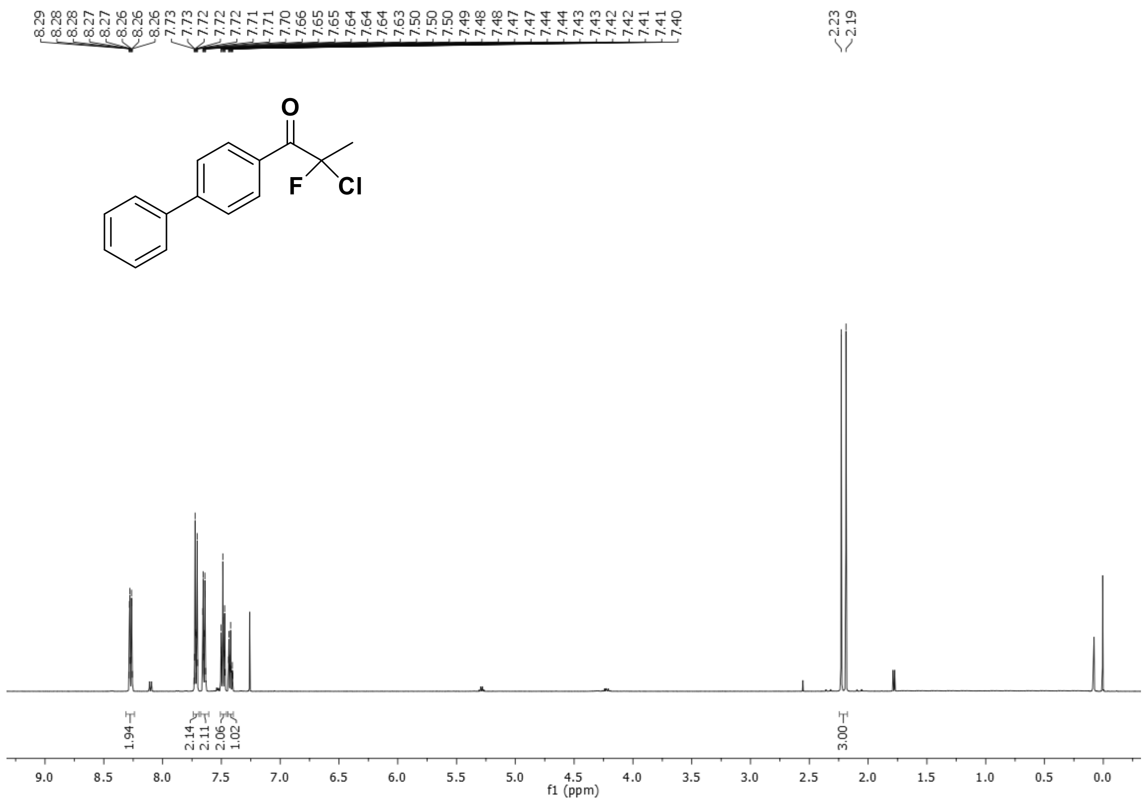

^{13}C{H} NMR *(125 MHz, $CDCl_3$)* of Molecule **3g**:

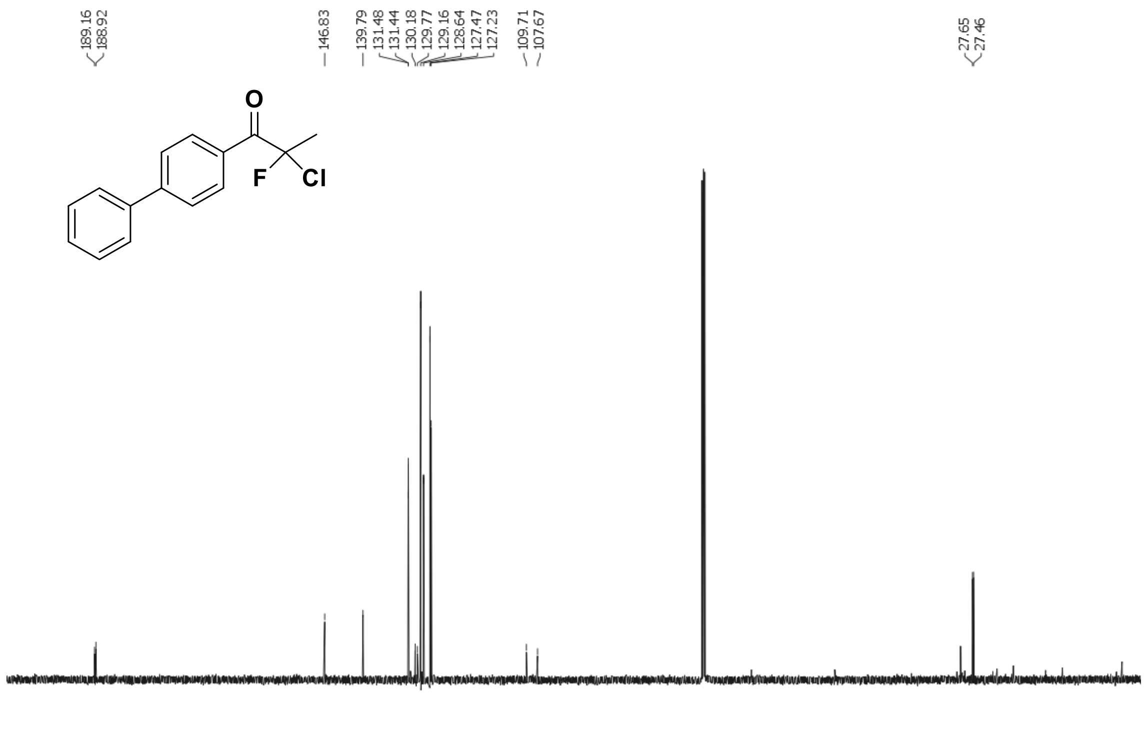

^{19}F NMR (376 MHz, $CDCl_3$) of Molecule **3g**:

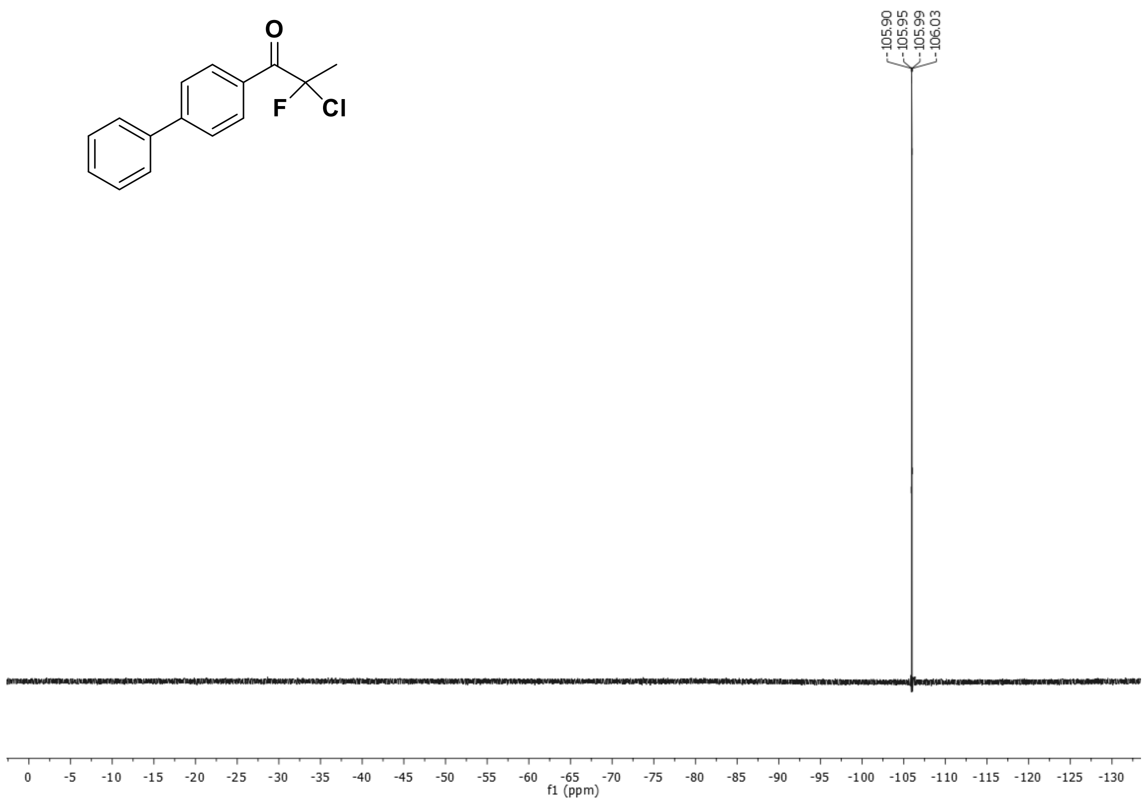

^{1}H NMR *(500 MHz, $CDCl_3$)* of Molecule **3h**:

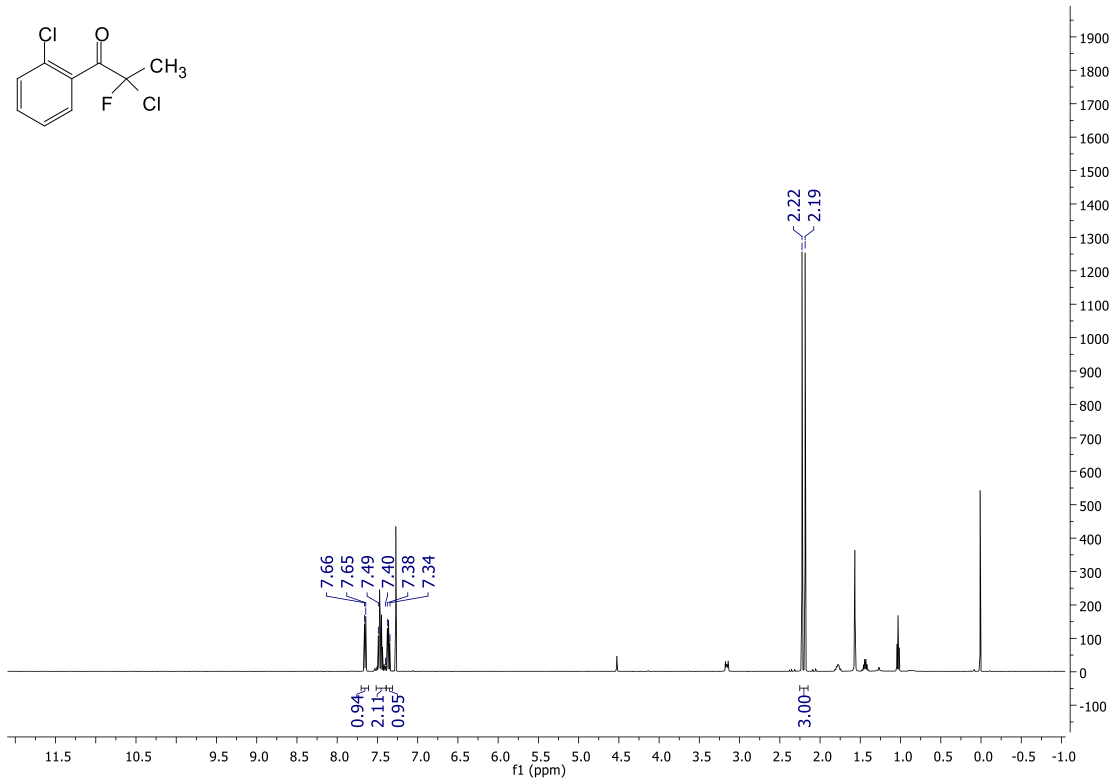

$^{13}C\{H\}$ NMR *(125 MHz, $CDCl_3$)* of Molecule **3h**:

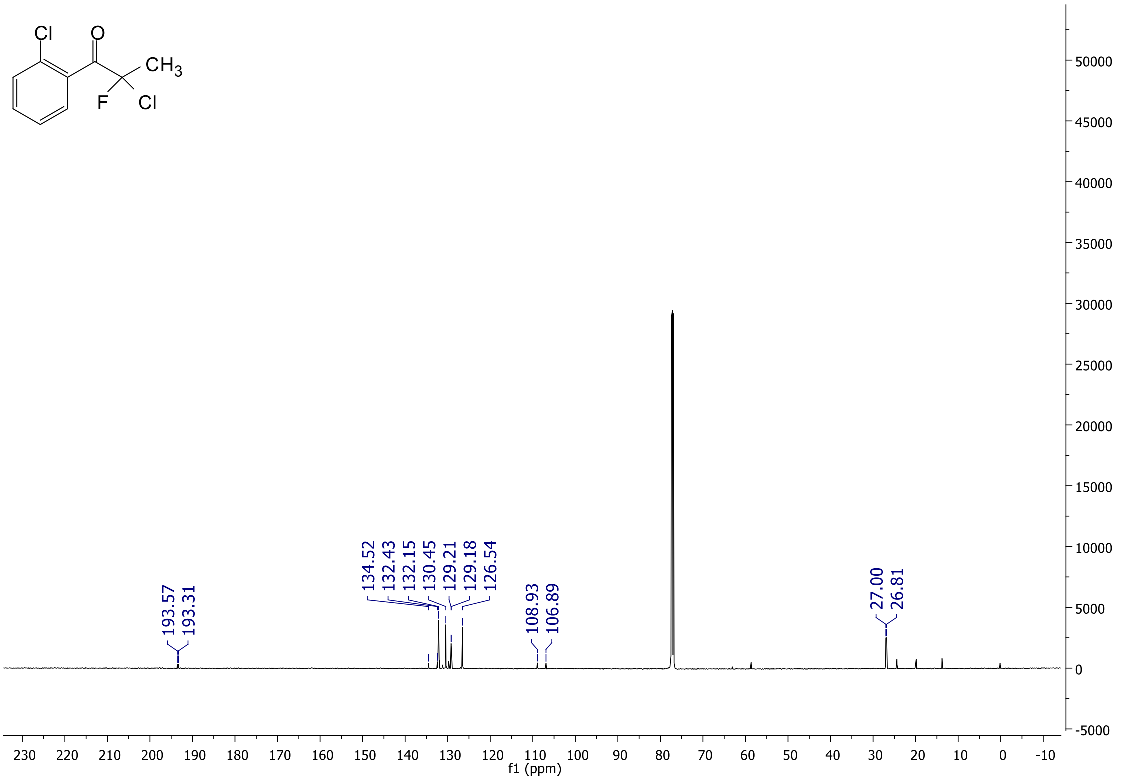

^{19}F NMR (*376 MHz, $CDCl_3$*) of Molecule **3h**:

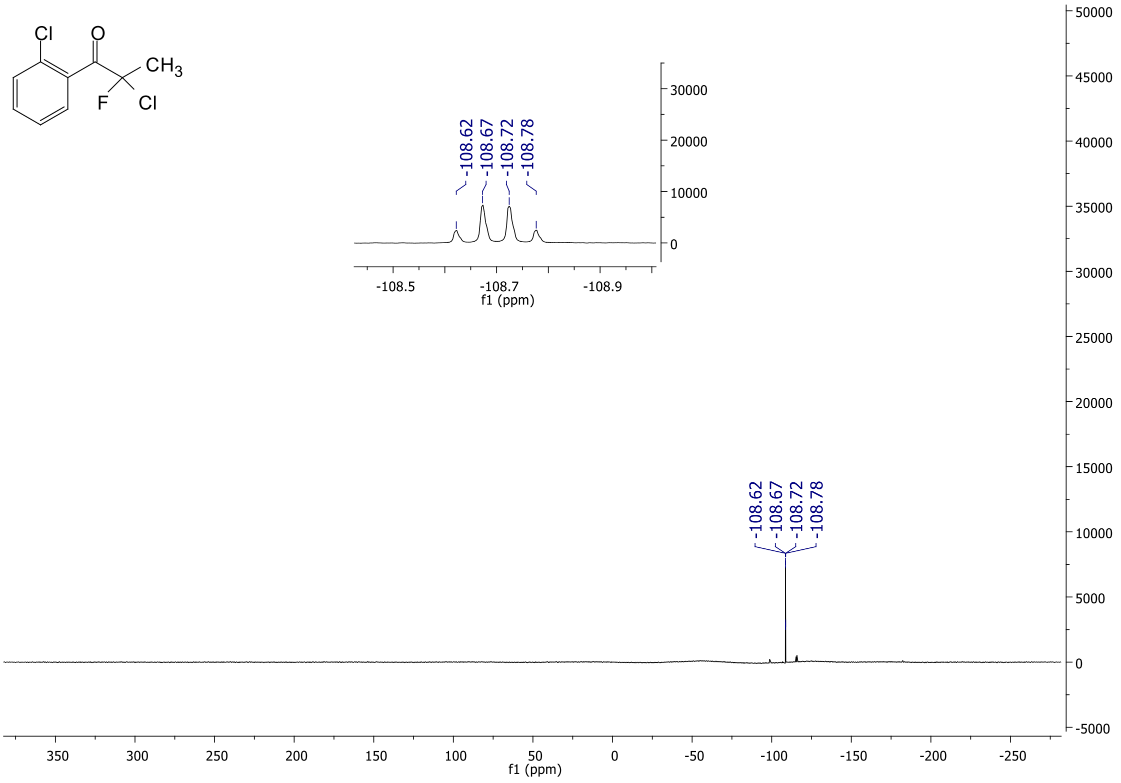

^{1}H NMR *(500 MHz, $CDCl_3$)* of Molecule **3i**:

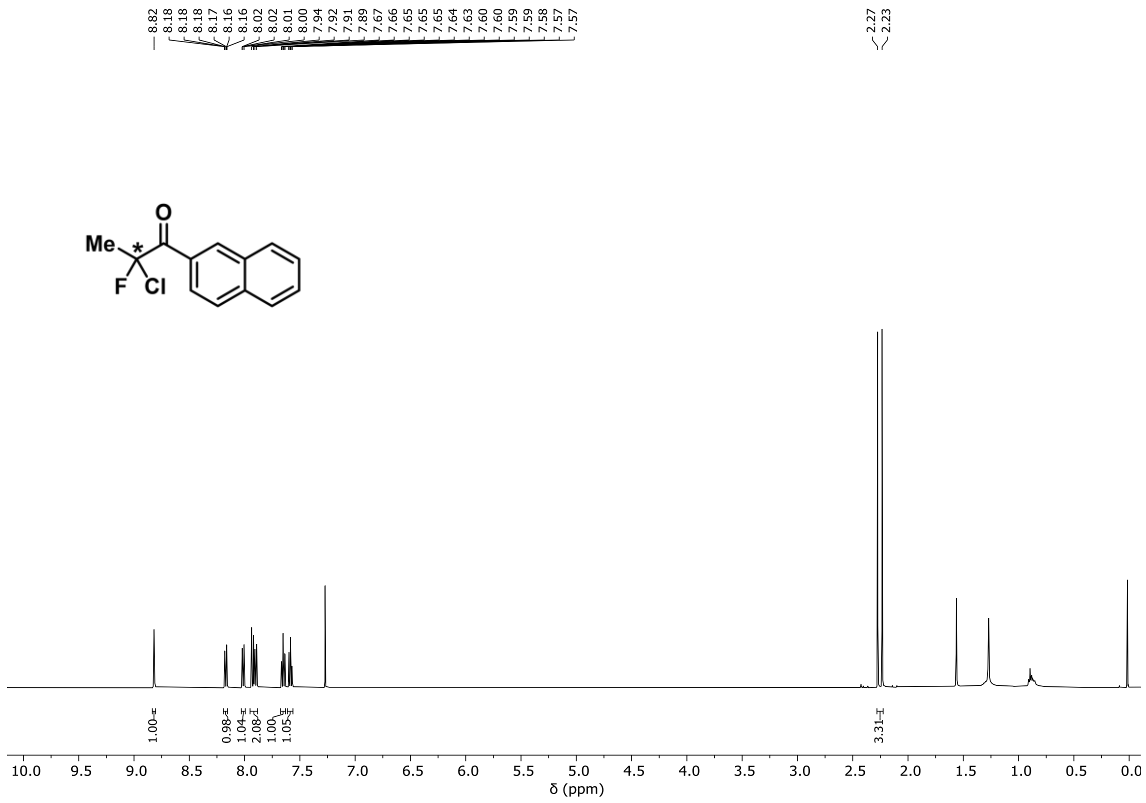

^{13}C{H} NMR *(125 MHz, $CDCl_3$)* of Molecule **3i**:

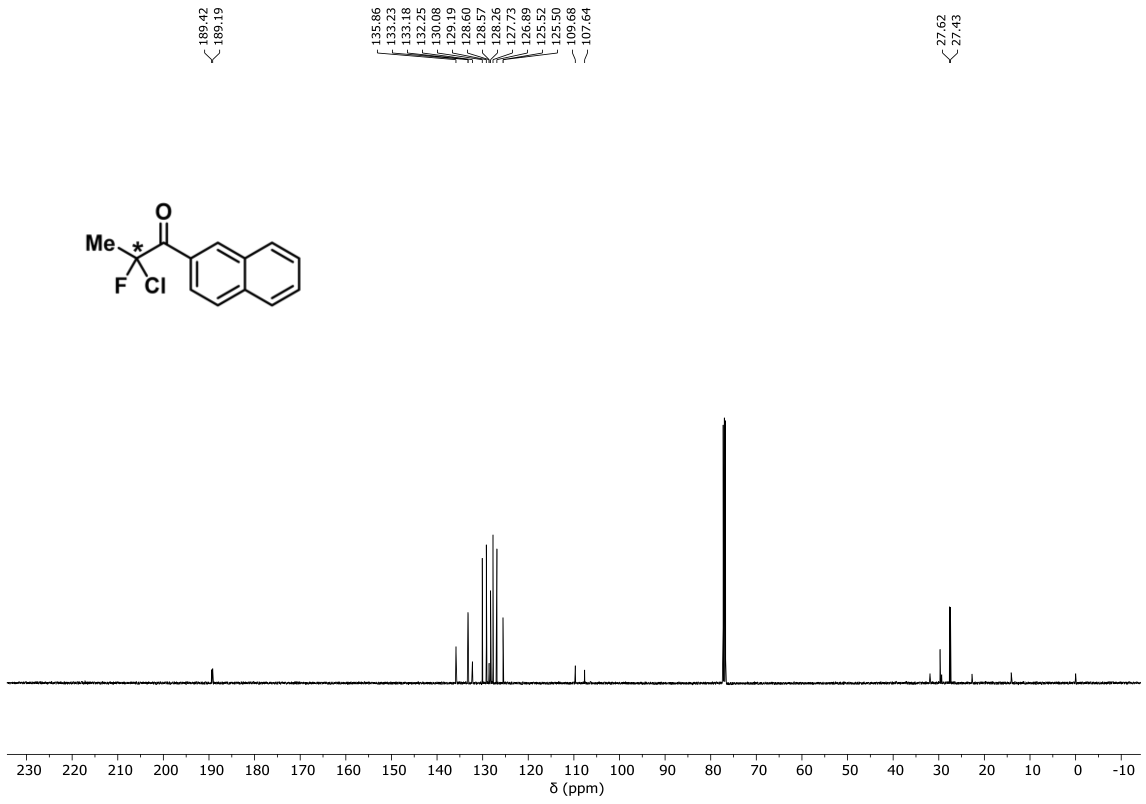

^{19}F NMR (376 MHz, $CDCl_3$) of Molecule **3i**:

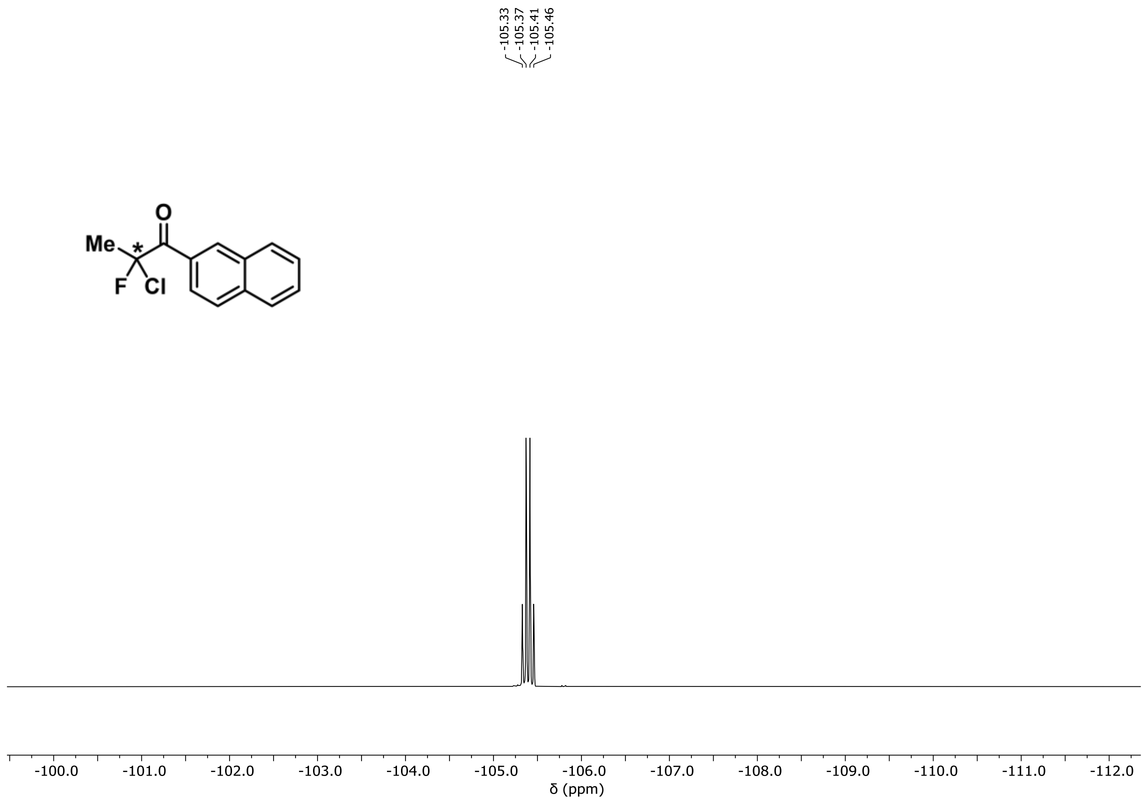

^{1}H NMR *(500 MHz, $CDCl_3$)* of Molecule **3j**:

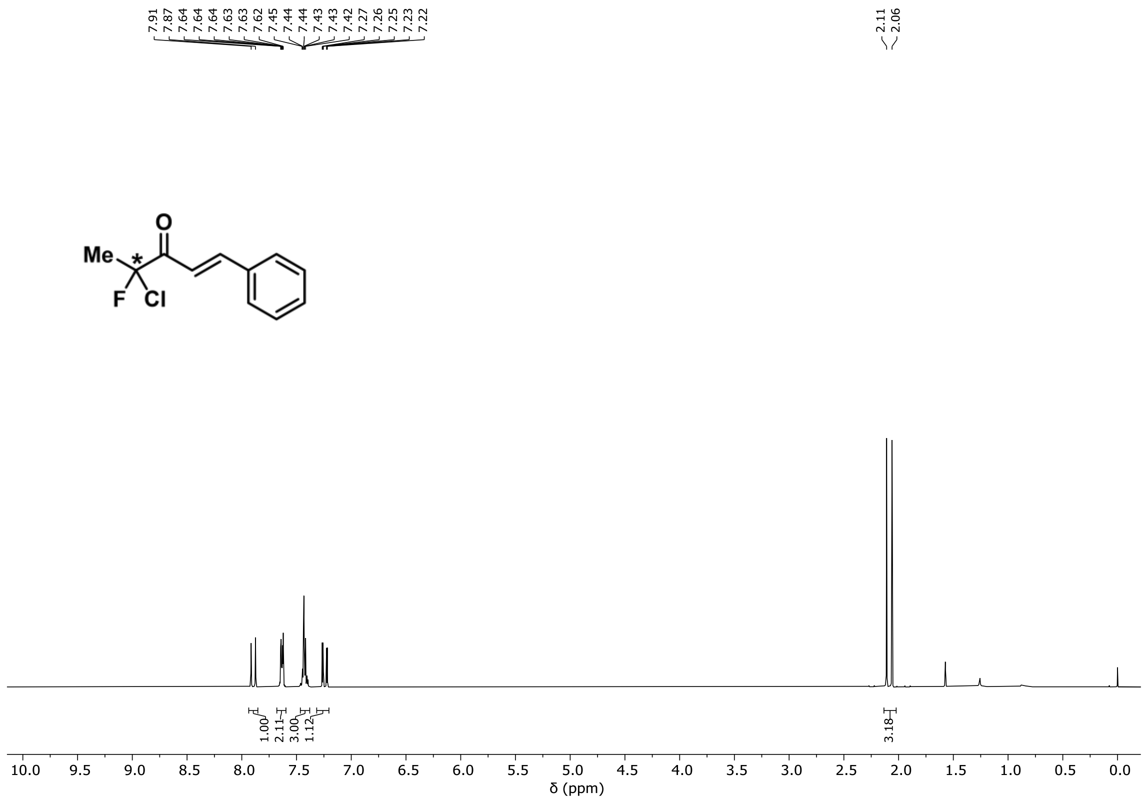

^{13}C{H} NMR *(125 MHz, $CDCl_3$)* of Molecule **3j**:

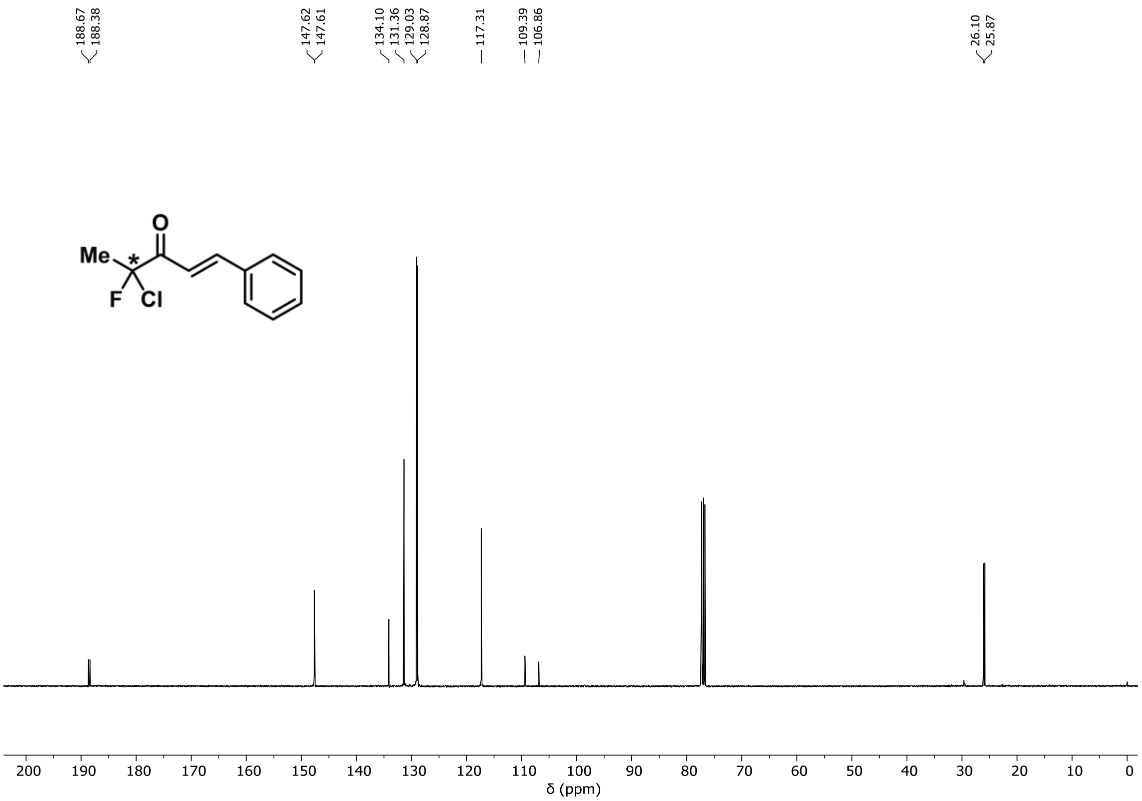

^{19}F NMR (376 MHz, $CDCl_3$) of Molecule **3j**:

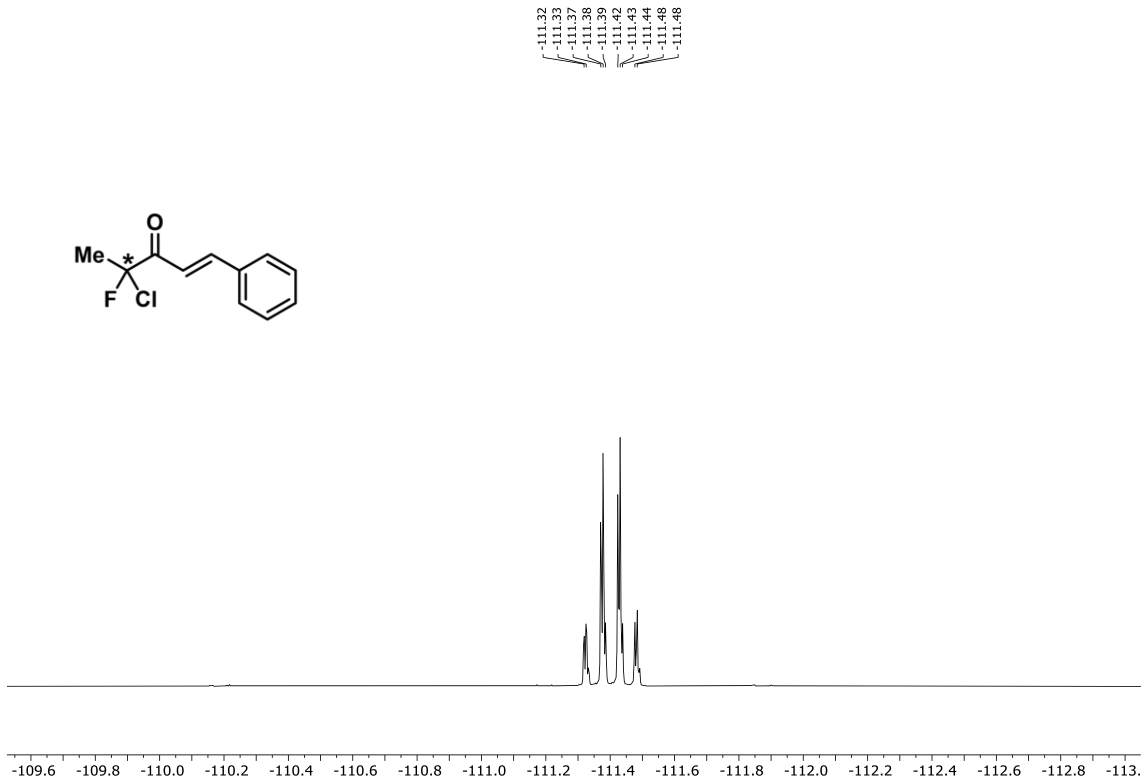

^{1}H NMR *(500 MHz, $CDCl_3$)* of Molecule **3k**:

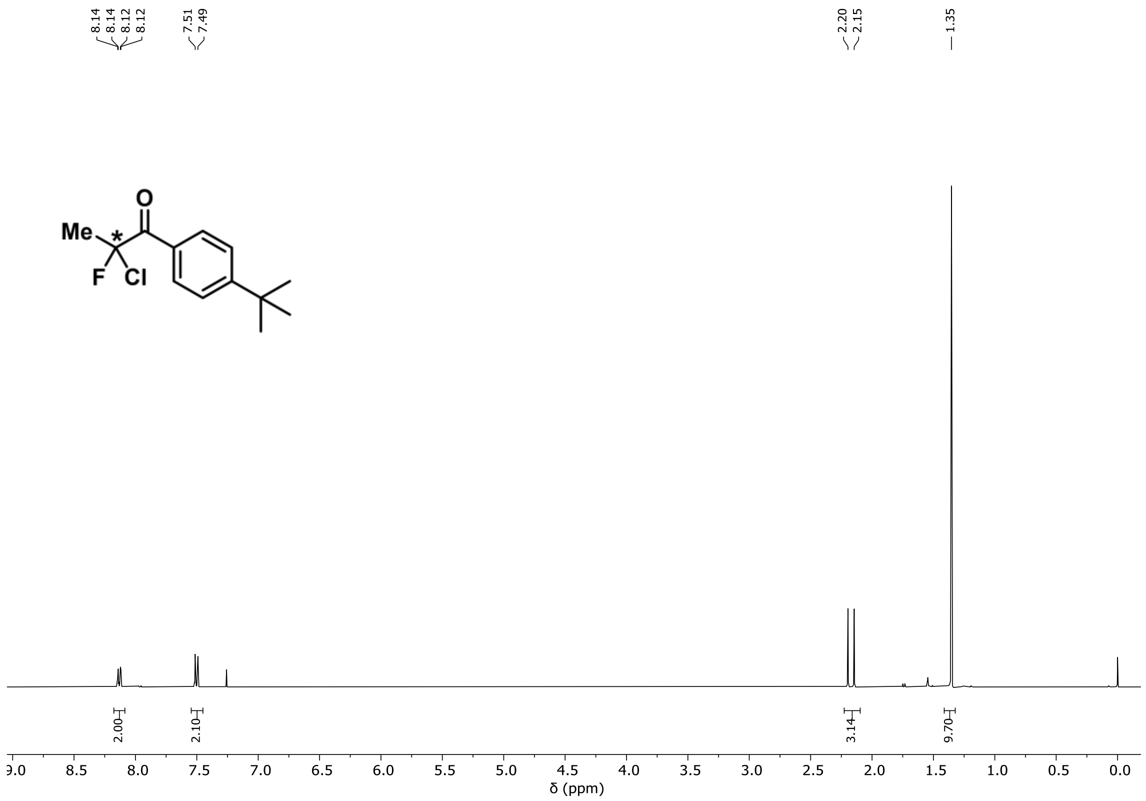

^{13}C{H} NMR *(125 MHz, $CDCl_3$)* of Molecule **3k**:

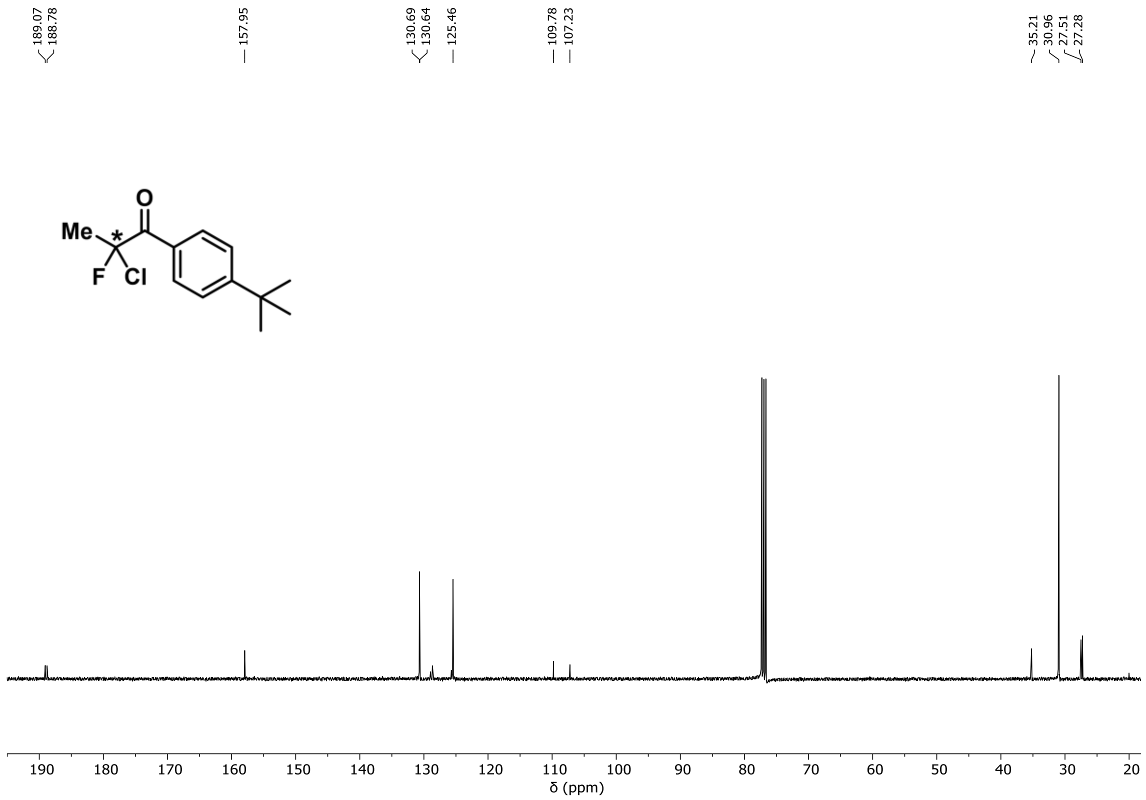

^{19}F NMR (376 MHz, $CDCl_3$) of Molecule **3k**:

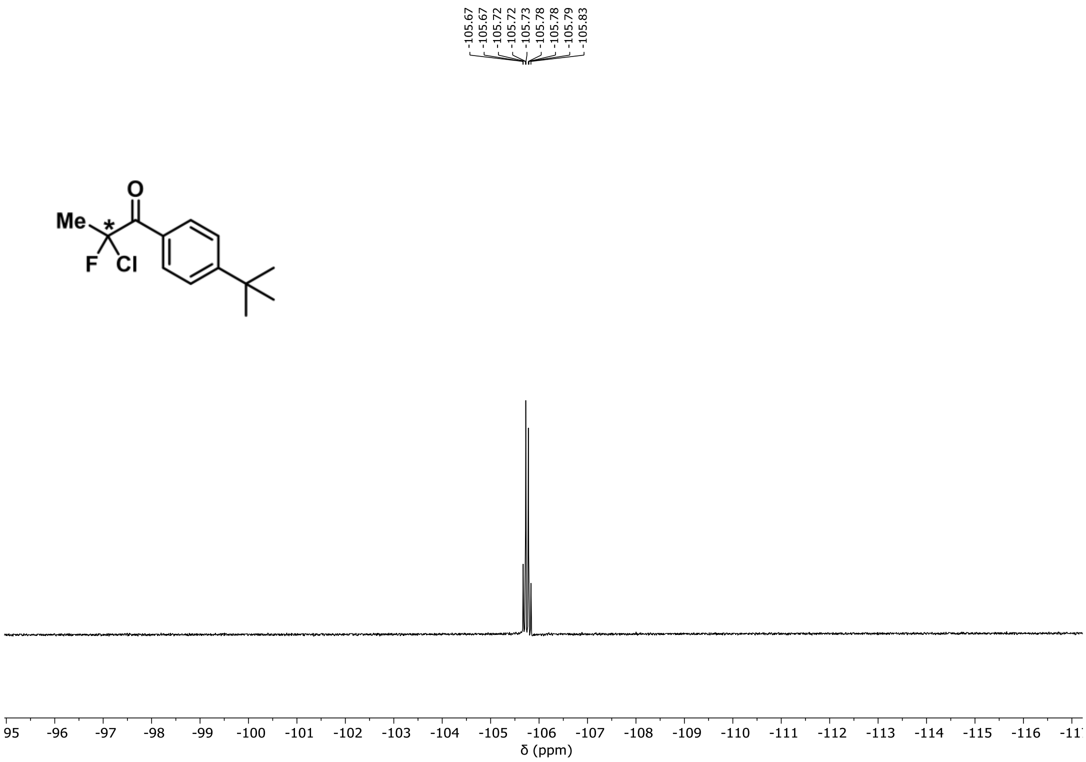

^{1}H NMR *(400 MHz, $CDCl_3$)* of Molecule **3l**:

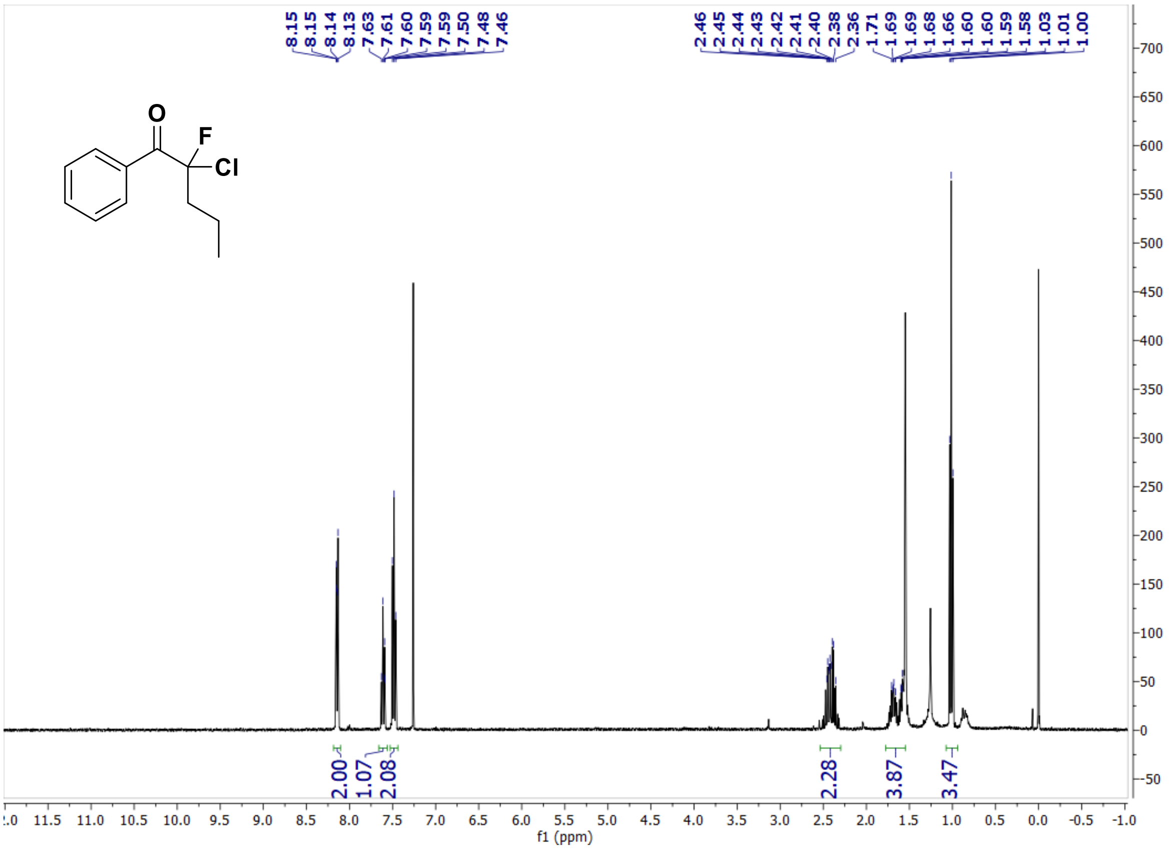

$^{13}C\{H\}$ NMR *(100 MHz, $CDCl_3$)* of Molecule **3l**:

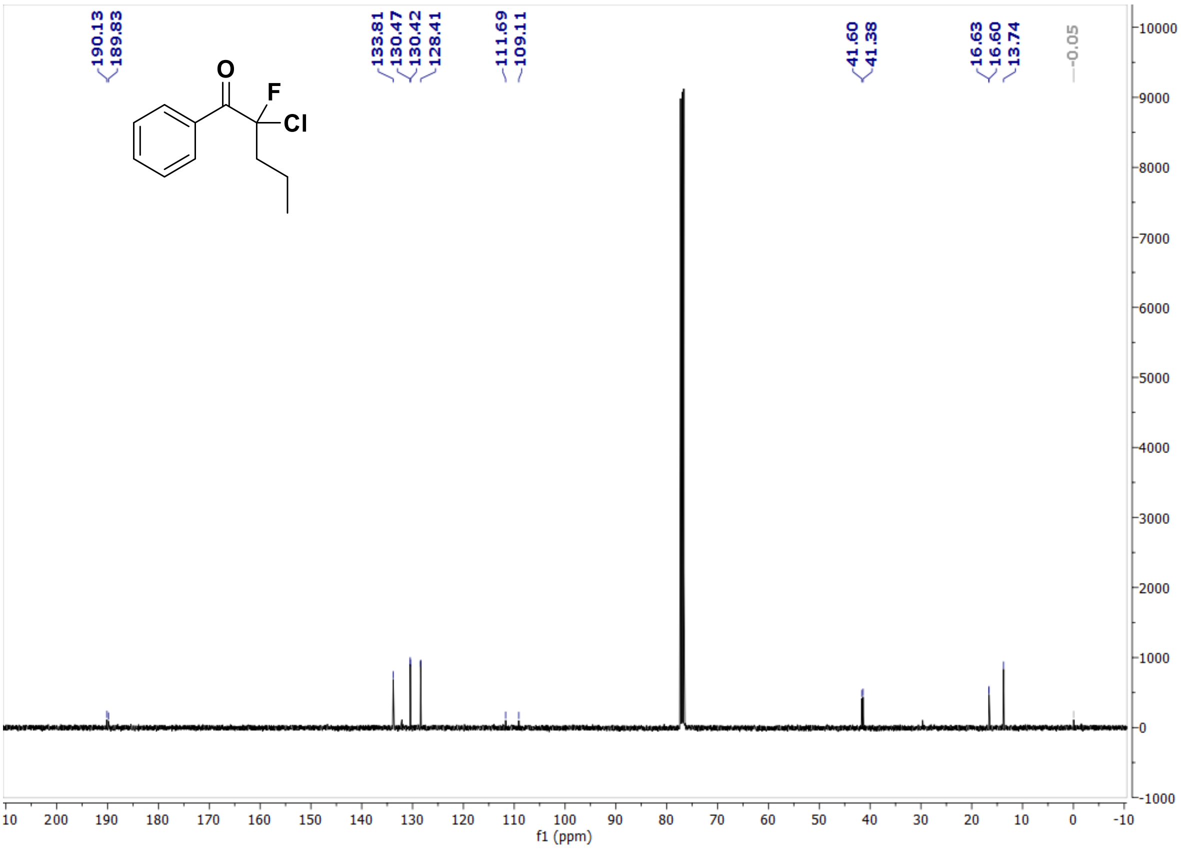

^{19}F NMR (376 MHz, $CDCl_3$) of Molecule **3l**:

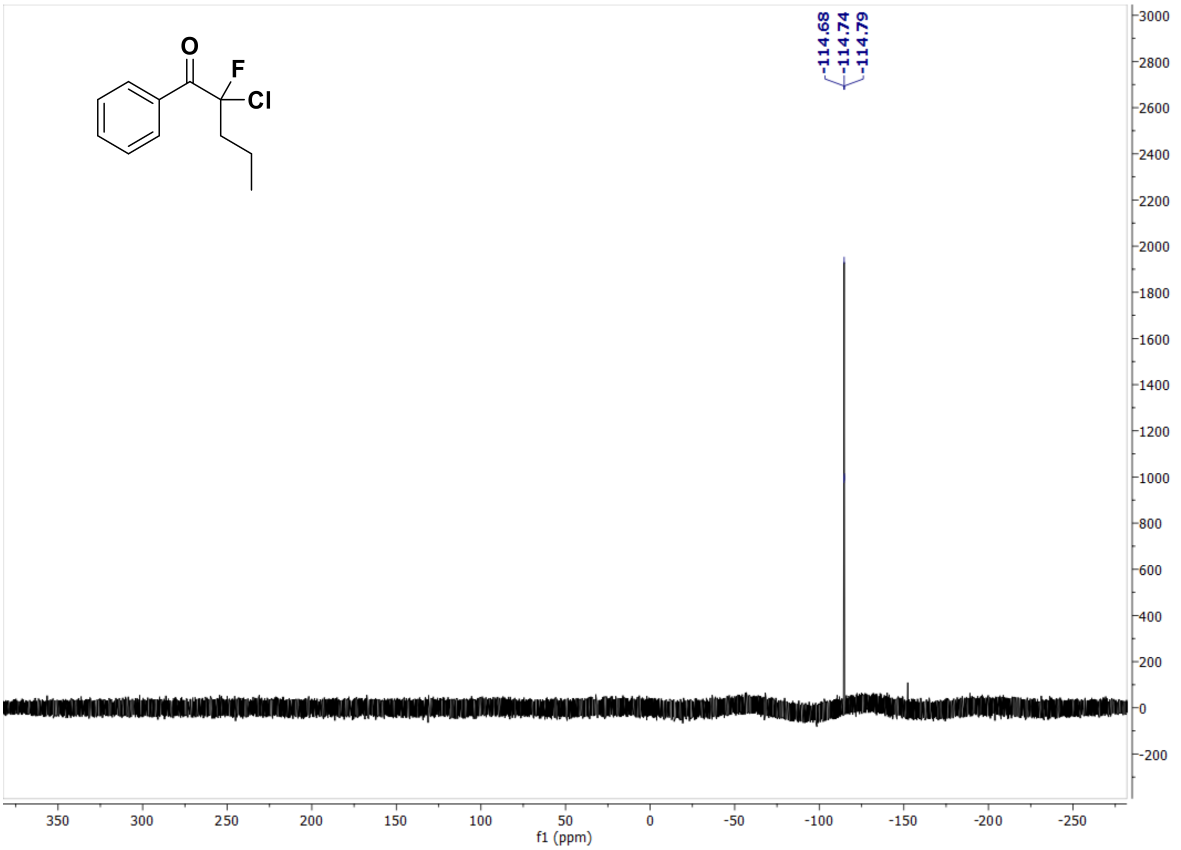

^{1}H NMR *(400 MHz, $CDCl_3$)* of Molecule **3m**:

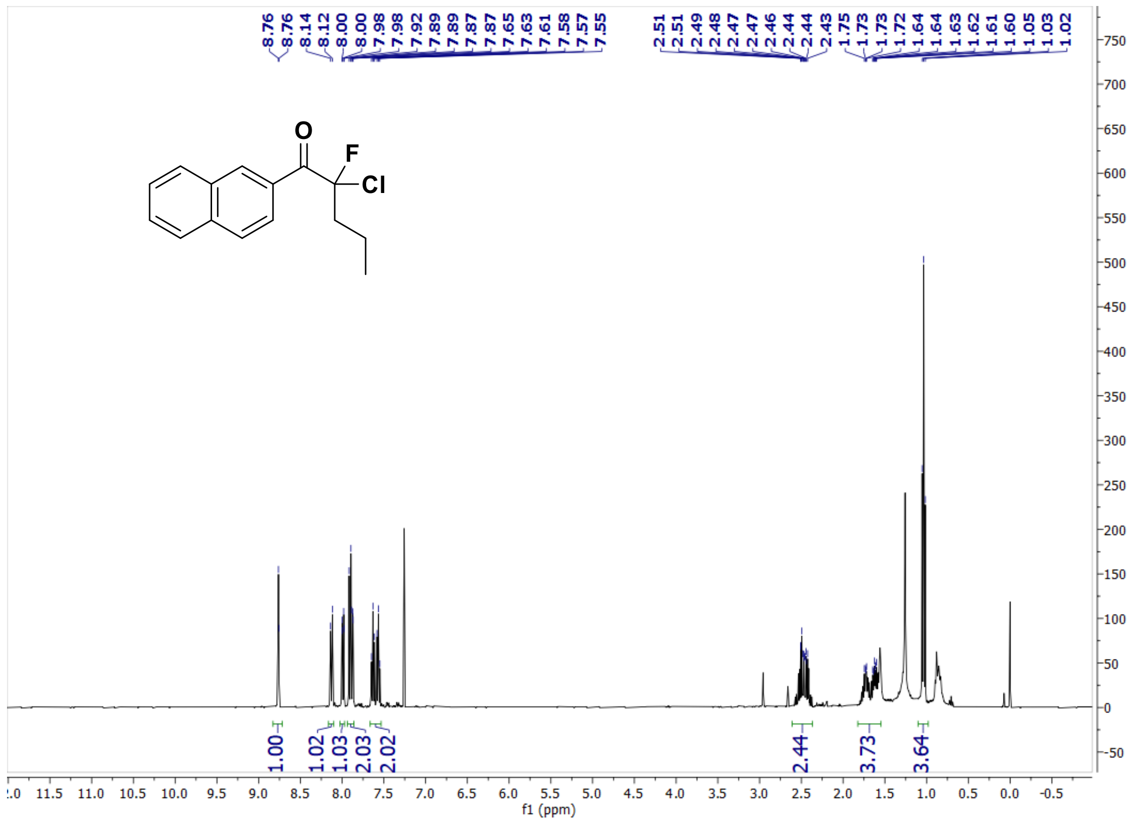

^{13}C{H} NMR *(100 MHz, $CDCl_3$)* of Molecule **3m**:

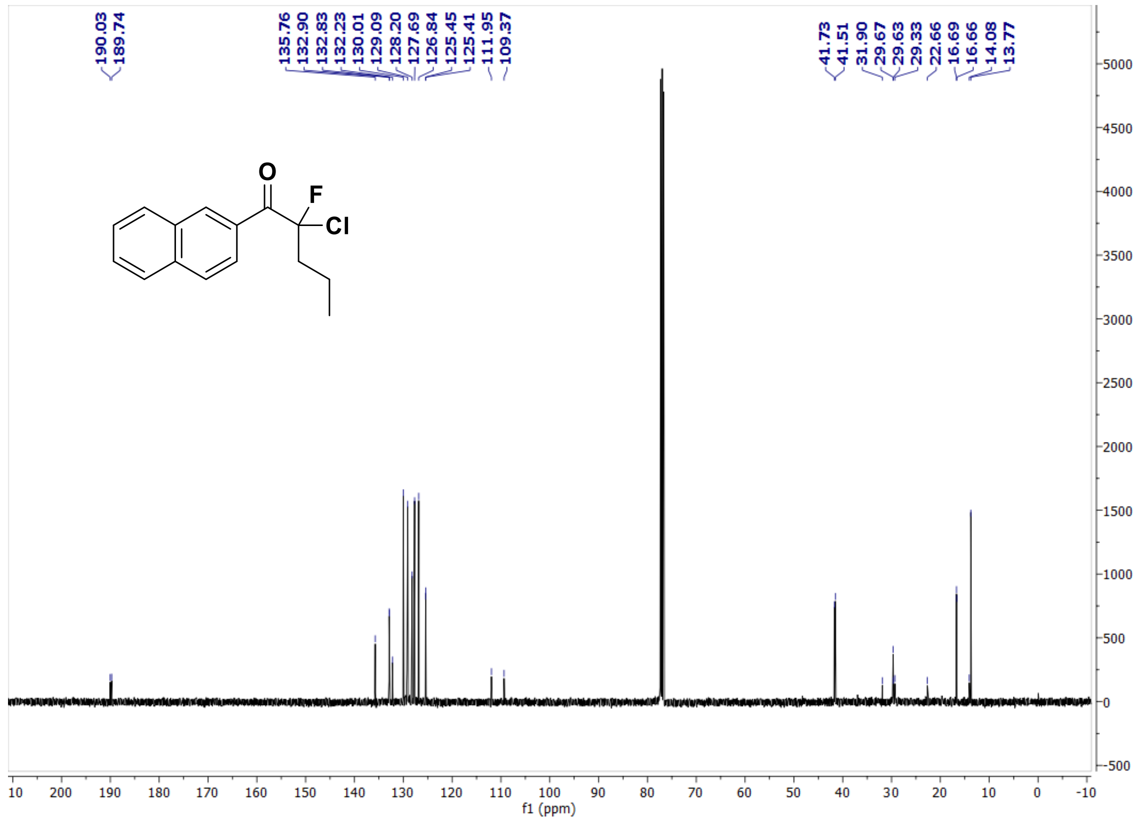

^{19}F NMR (376 MHz, $CDCl_3$) of Molecule **3m**:

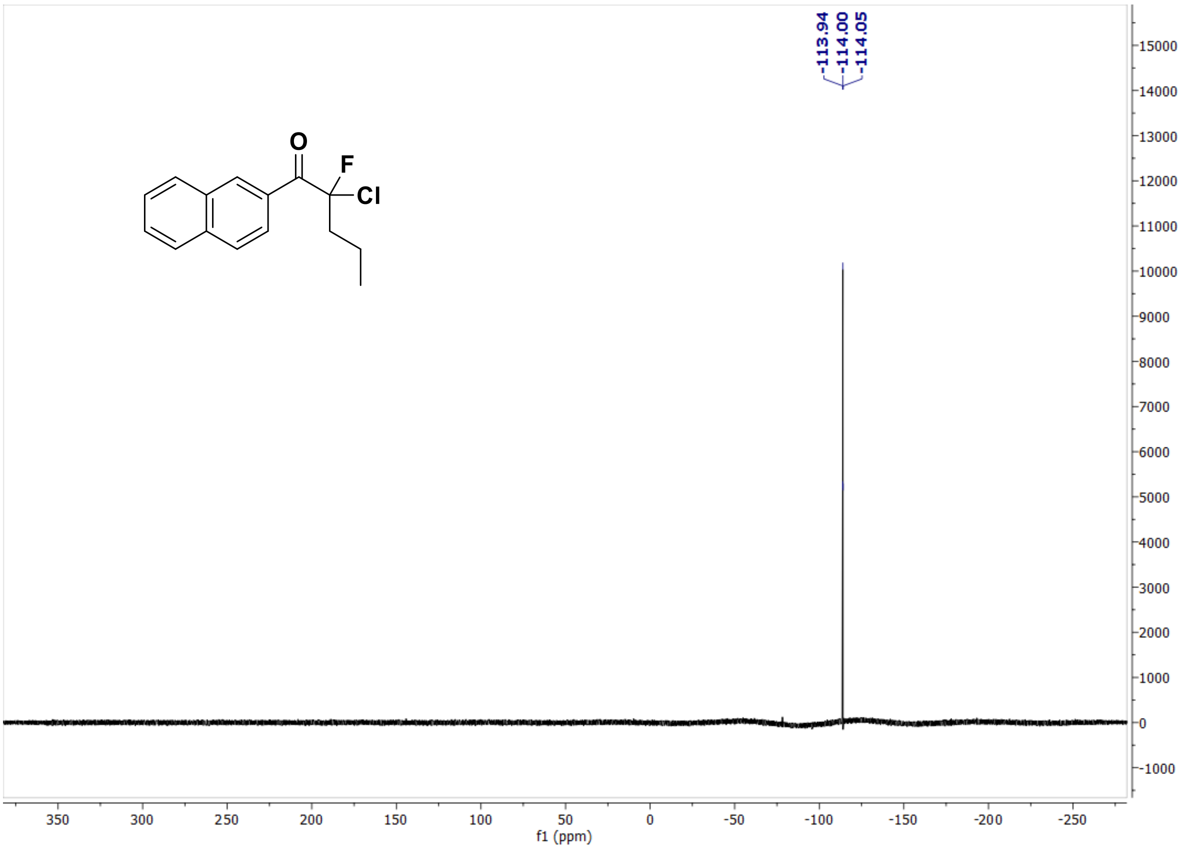

^{1}H NMR *(500 MHz, $CDCl_3$)* of Molecule **3n**:

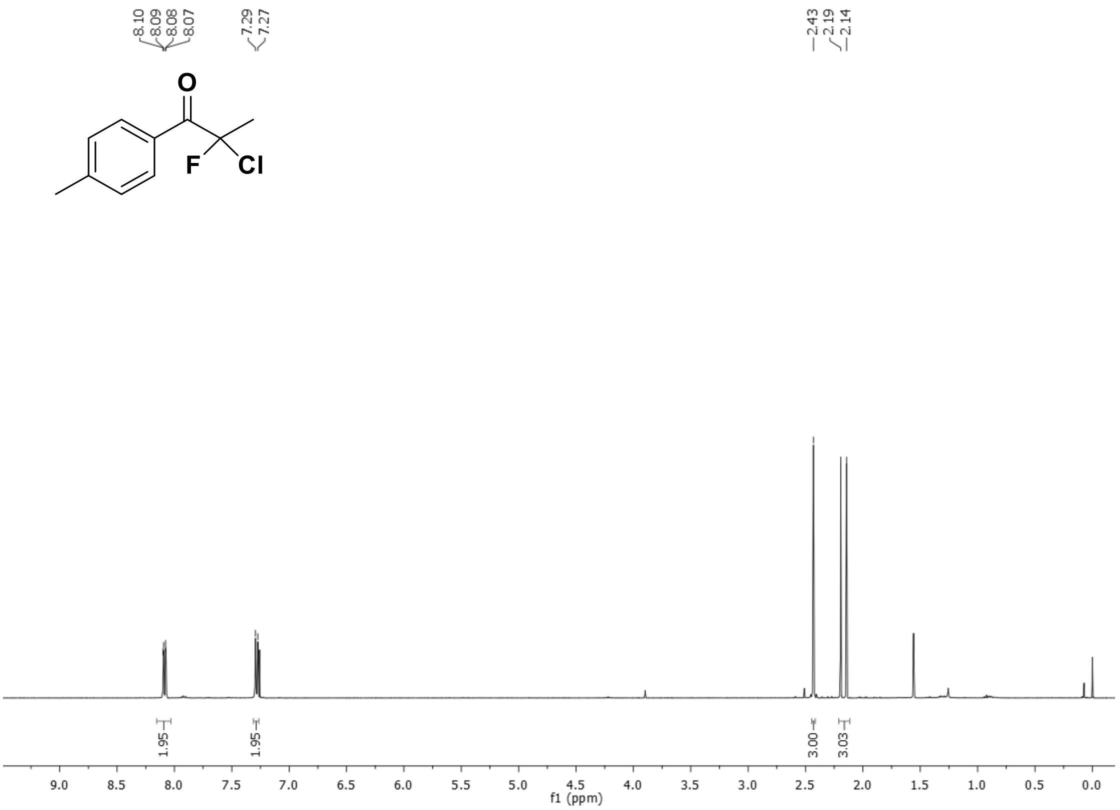

$^{13}C\{H\}$ NMR *(125 MHz, $CDCl_3$)* of Molecule **3n**:

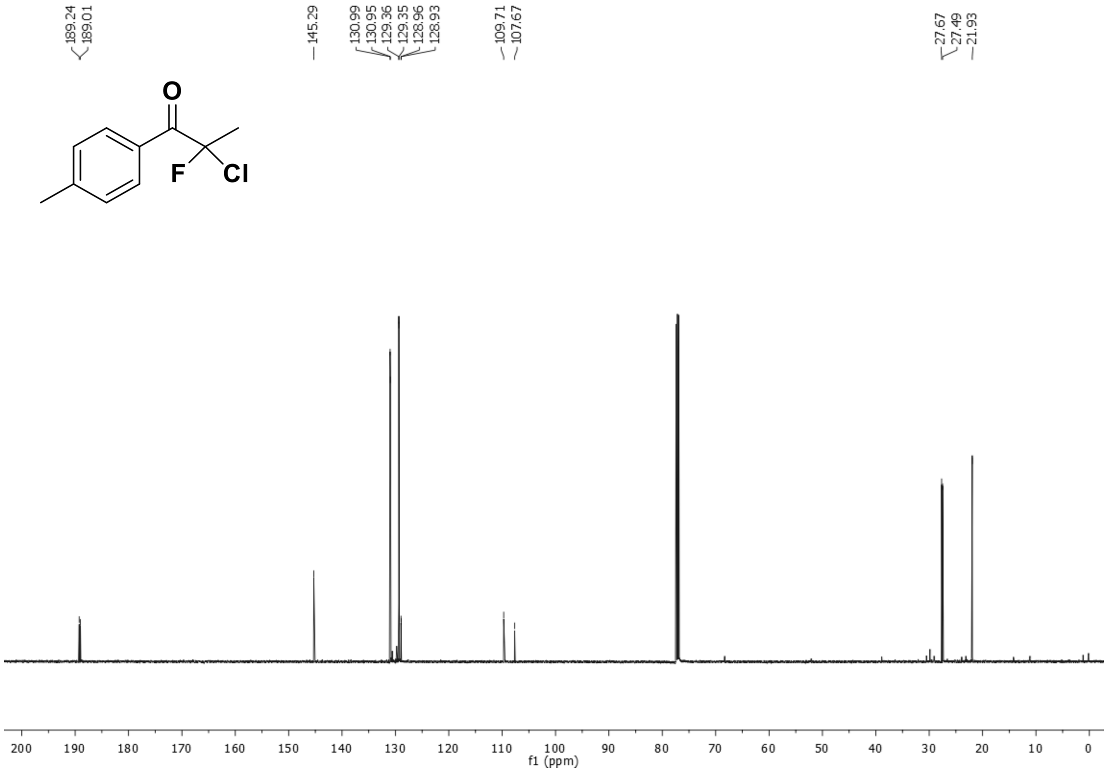

^{19}F NMR (376 MHz, $CDCl_3$) of Molecule **3n**:

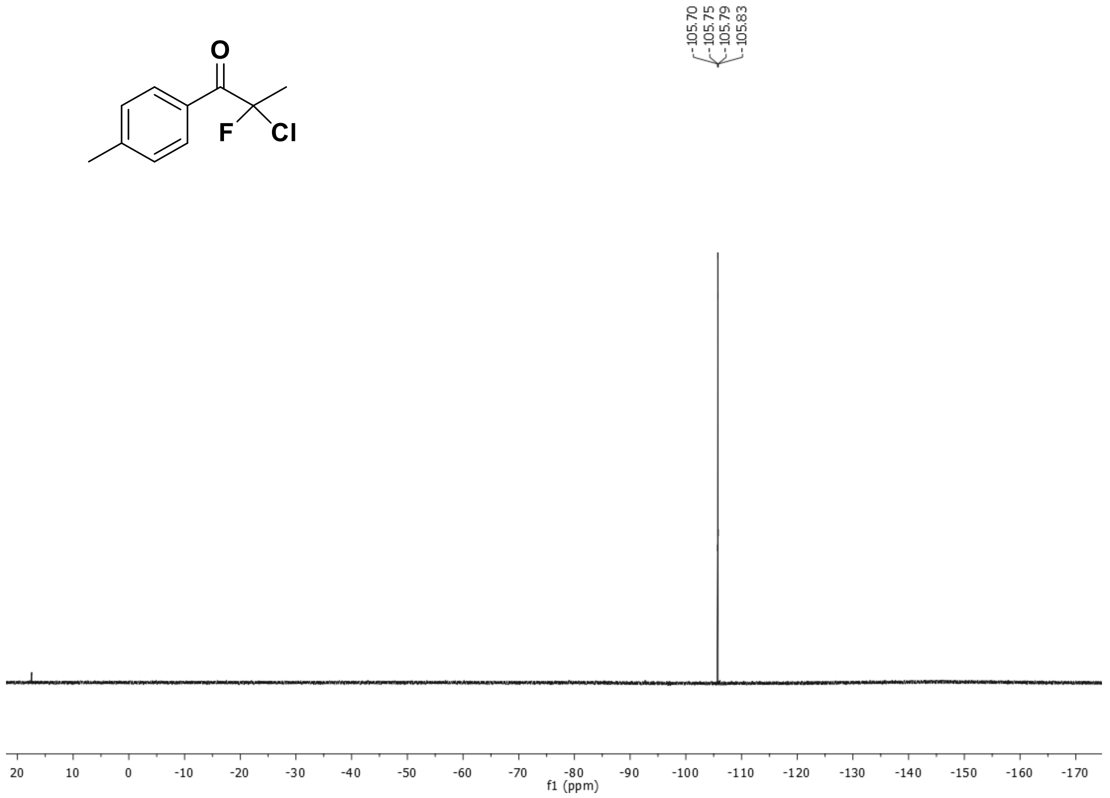

^{1}H NMR *(500 MHz, $CDCl_3$)* of Molecule **4a**:

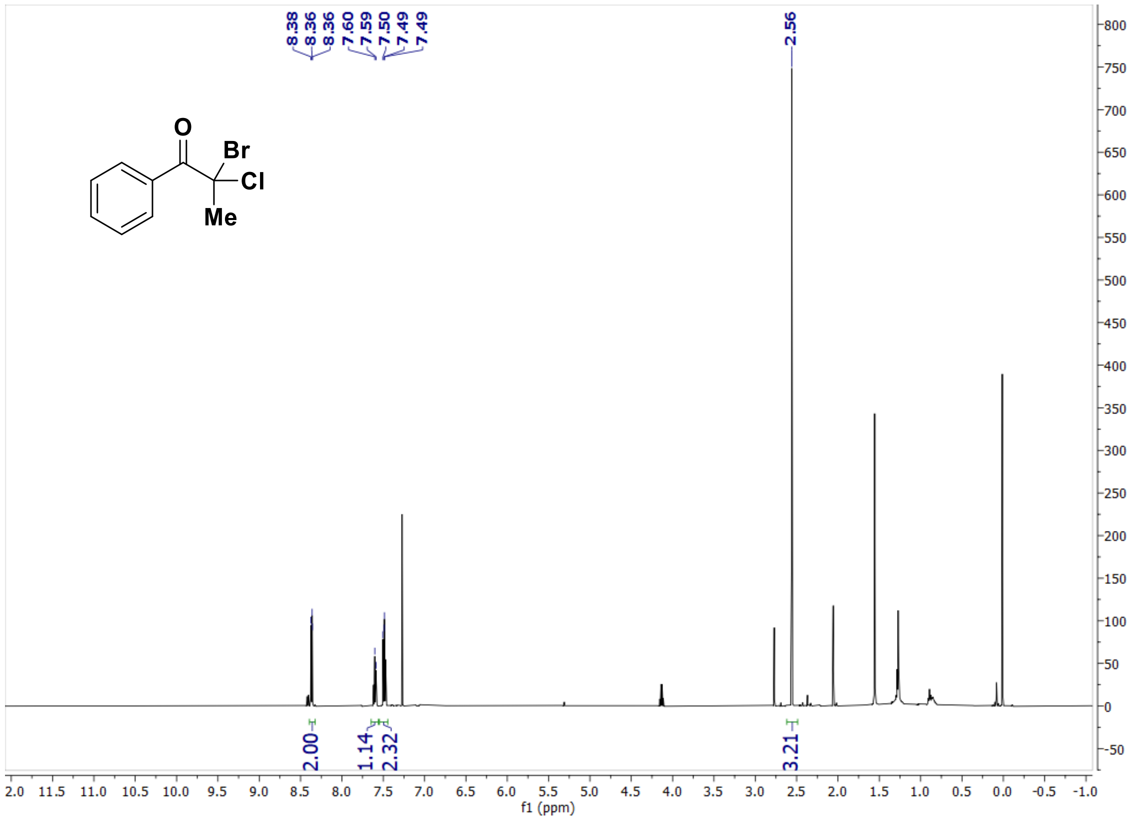

^{13}C{H} NMR *(125 MHz, $CDCl_3$)* of Molecule **4a**:

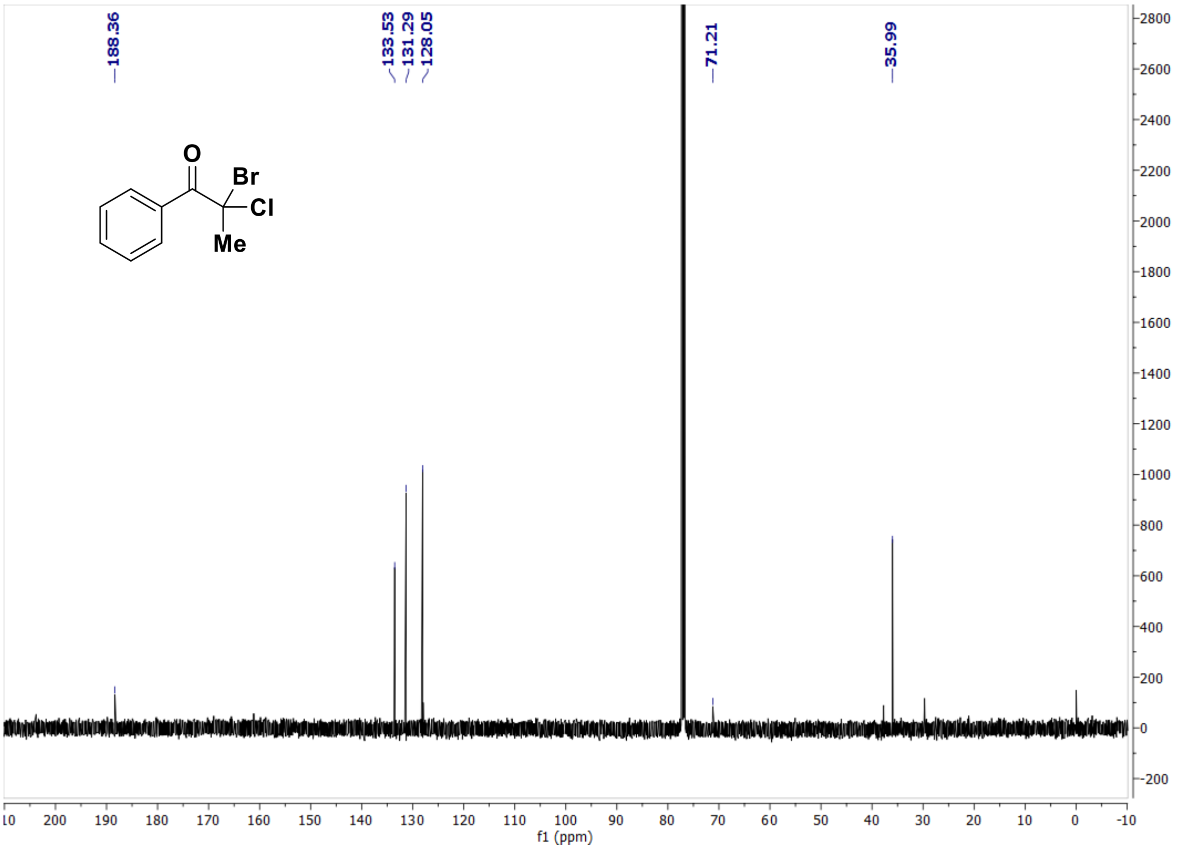

H NMR *(500 MHz, $CDCl_3$)* of Molecule **4b**:

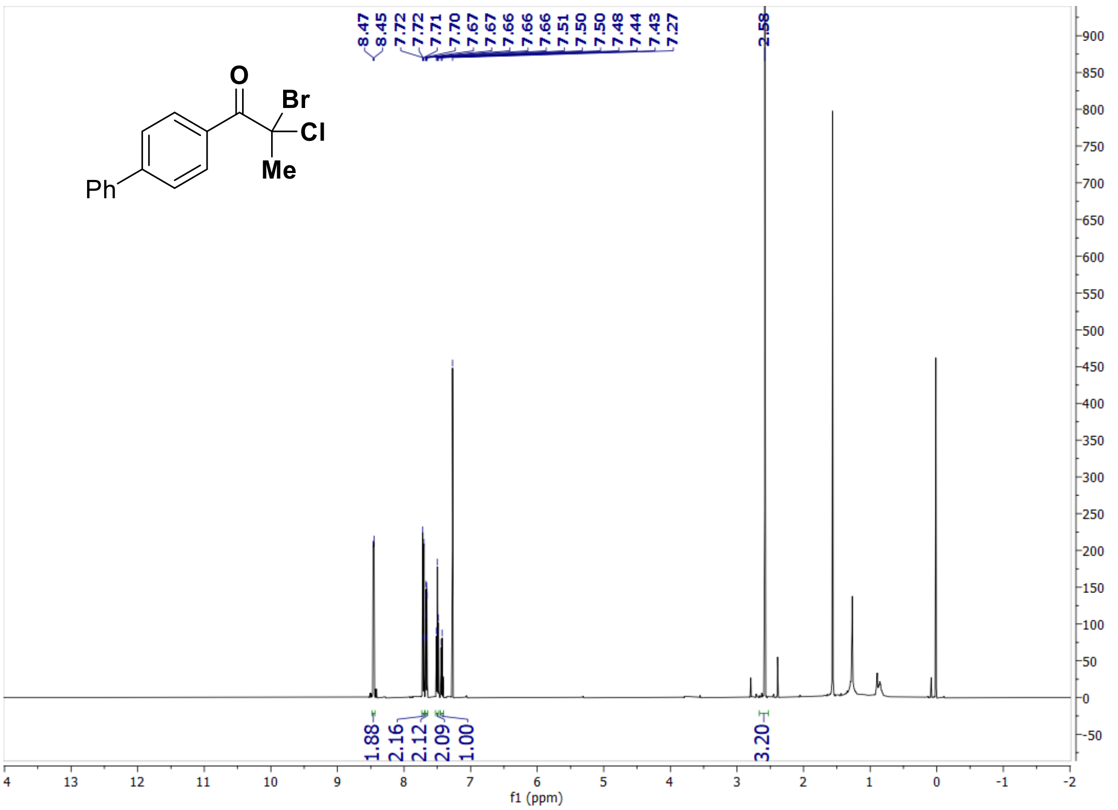

$^{13}C\{H\}$ NMR *(125 MHz, $CDCl_3$)* of Molecule **4b**:

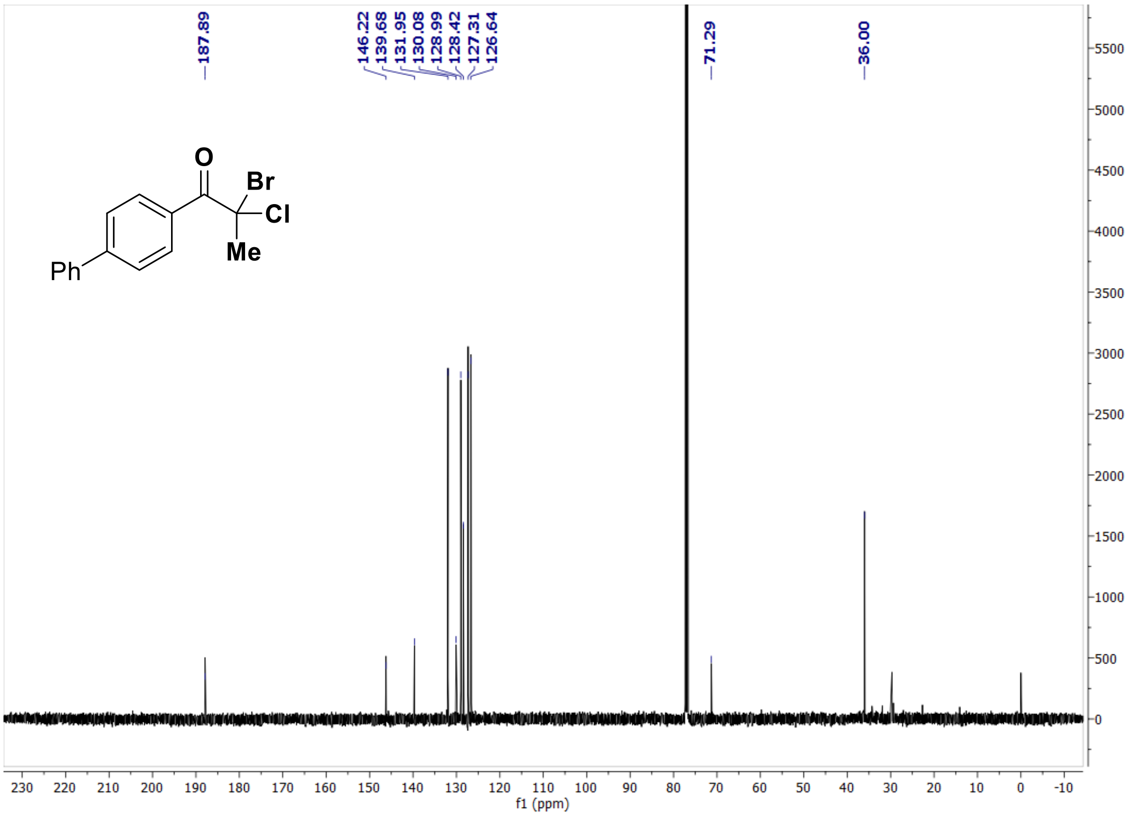

^{1}H NMR *(500 MHz, $CDCl_3$)* of Molecule **3’a**:

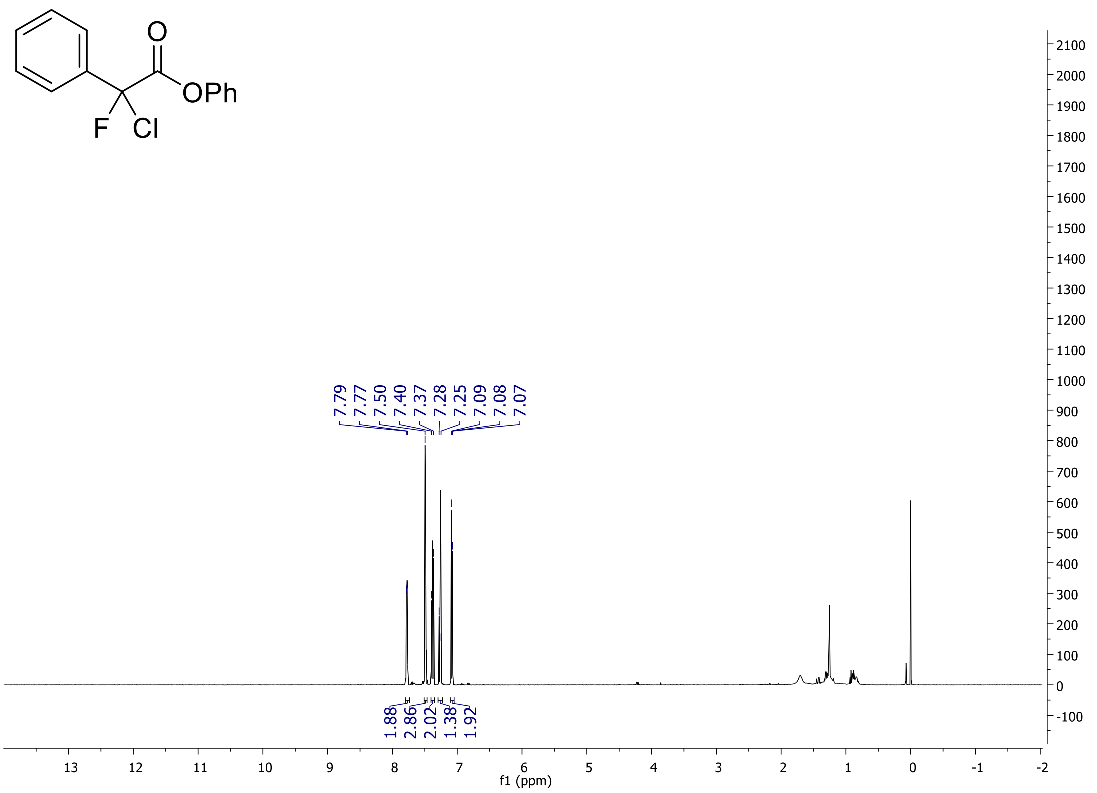

^{13}C{H} NMR *(125 MHz, $CDCl_3$)* of Molecule **3’a**:

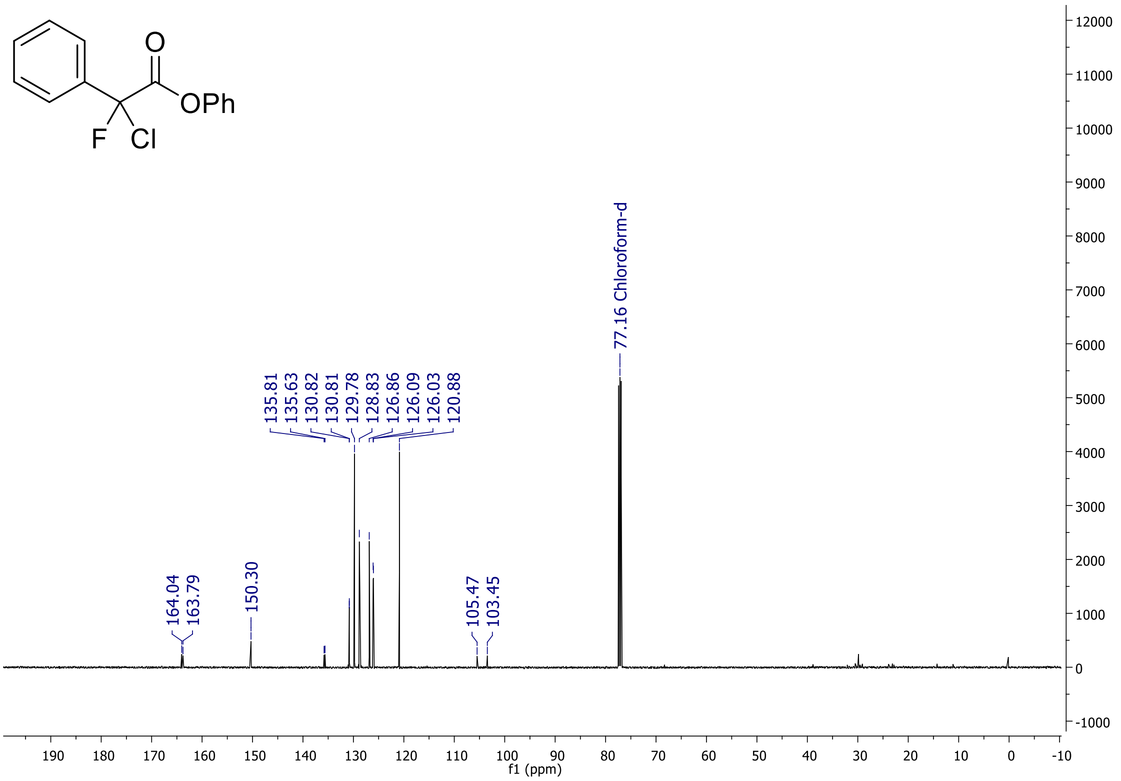

^{19}F NMR (376 MHz, $CDCl_3$) of Molecule **3’a**:

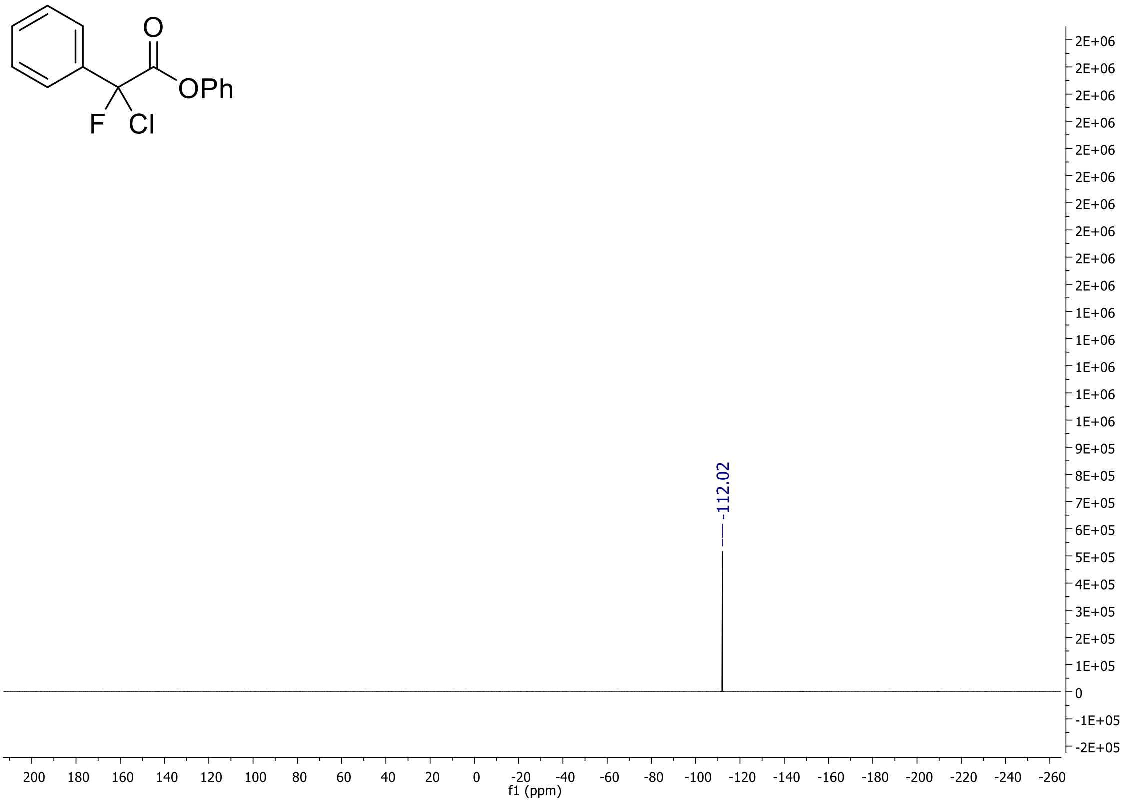

^1H NMR *(400 MHz, $CDCl_3$)* of Molecule **3’b**:

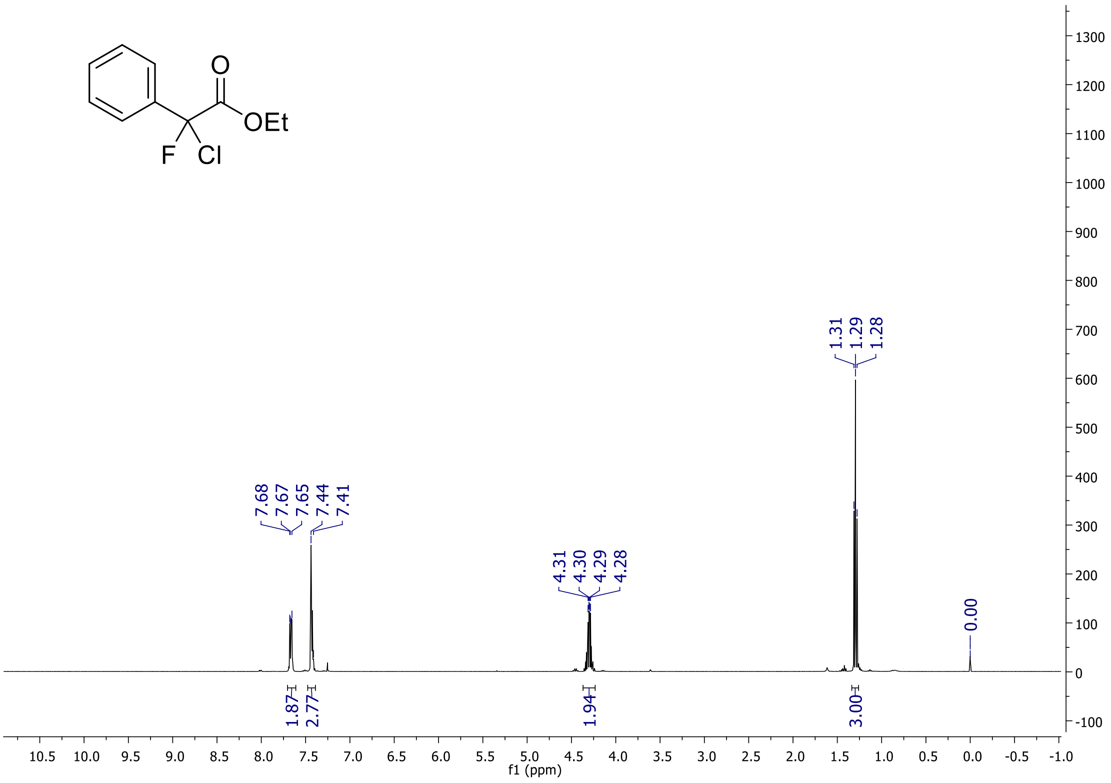

$^{13}C\{H\}$ NMR *(100 MHz, CDCl$_3$)* of Molecule **3'b**:

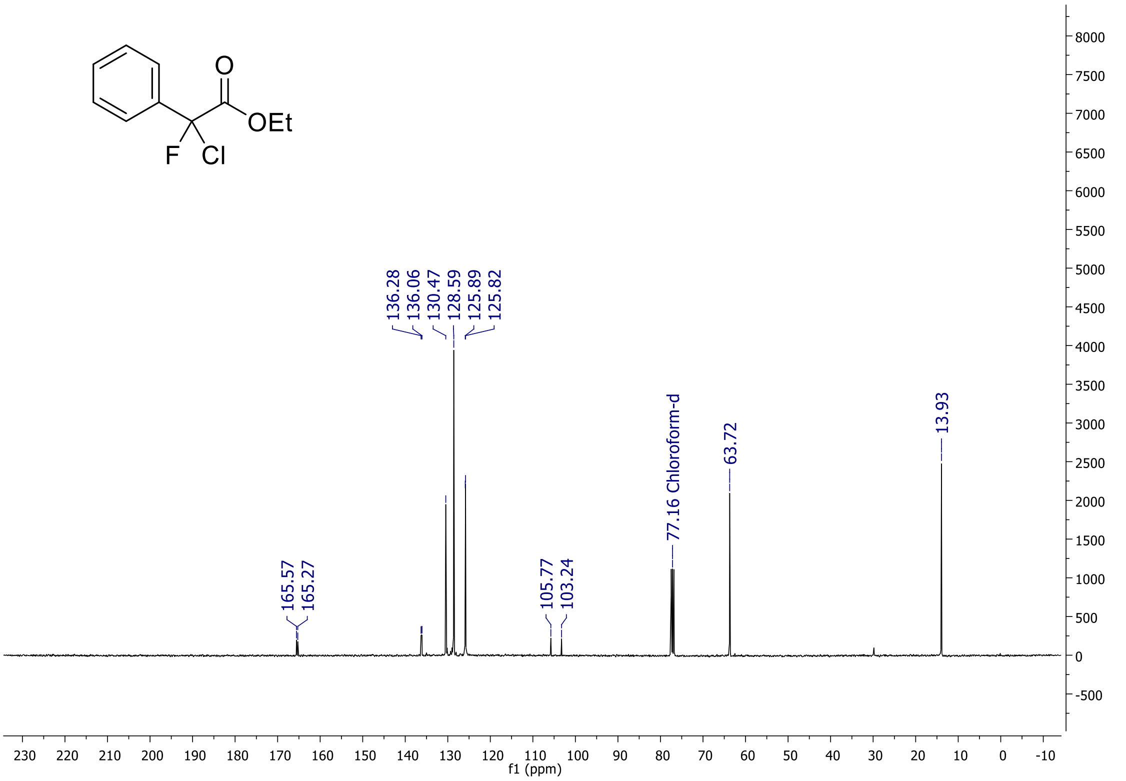

^{19}F NMR (*470 MHz, CDCl$_3$*) of Molecule **3'b**:

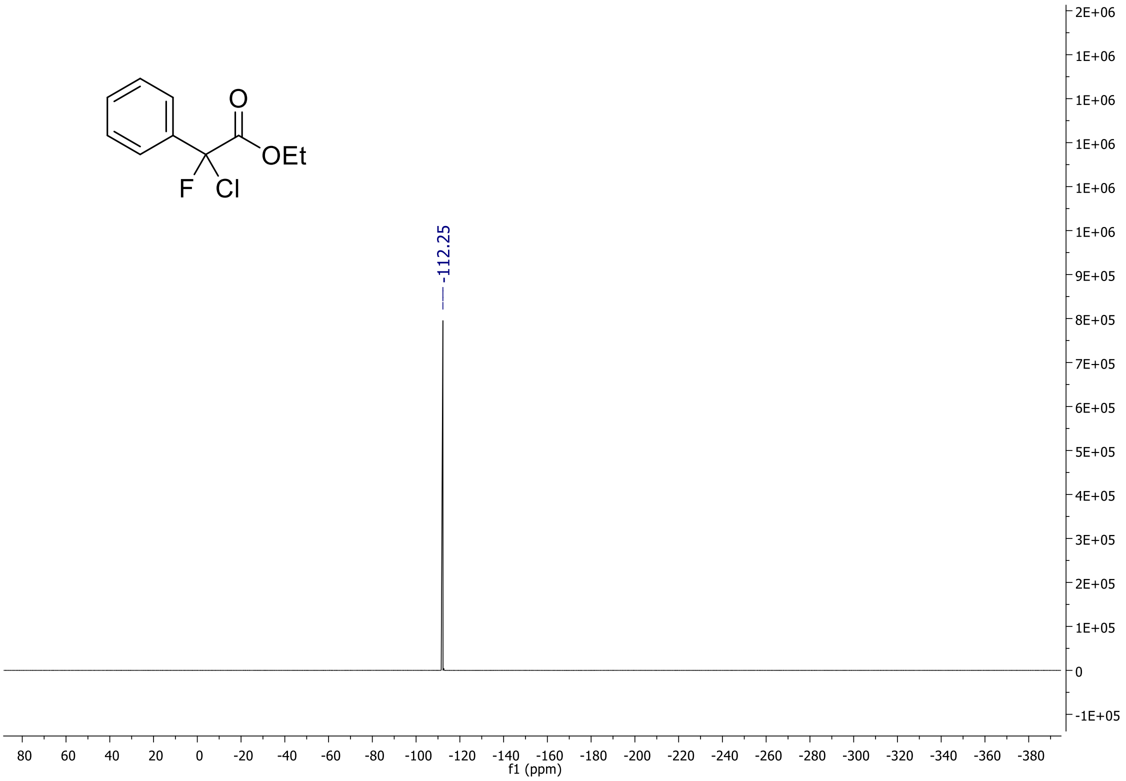

^{1}H NMR *(500 MHz, CDCl$_3$)* of Molecule **3’c**:

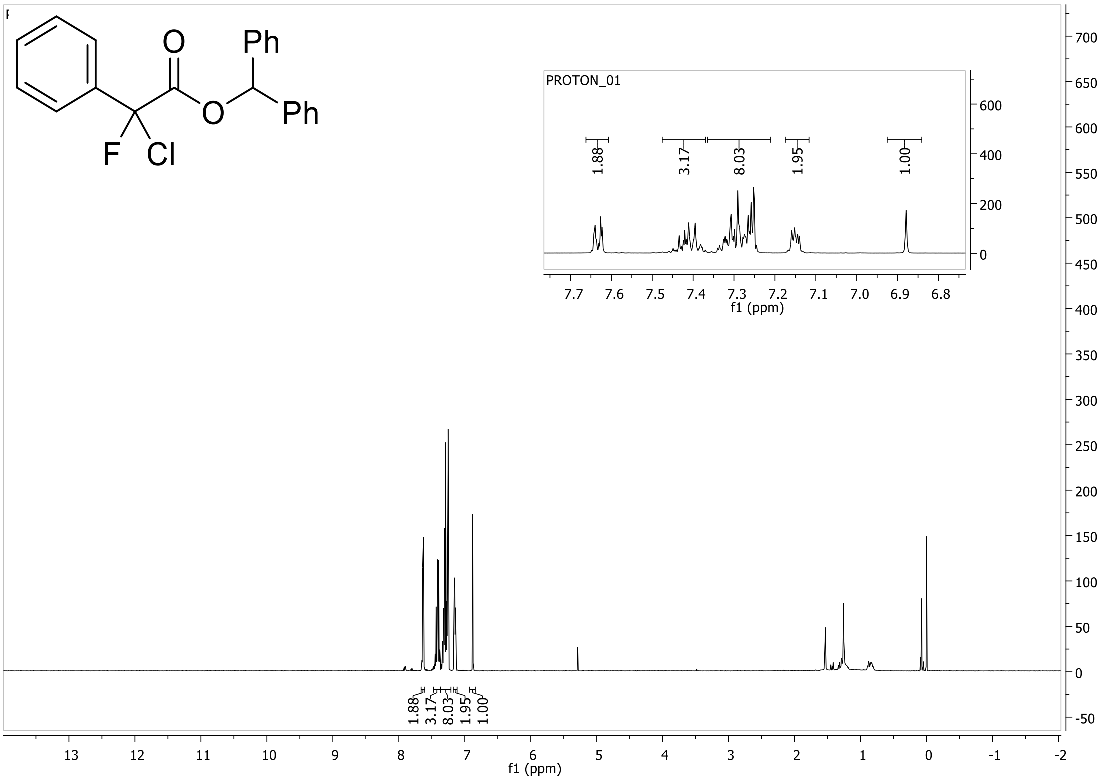

^{13}C{H} NMR *(125 MHz, CDCl$_3$)* of Molecule **3’c**:

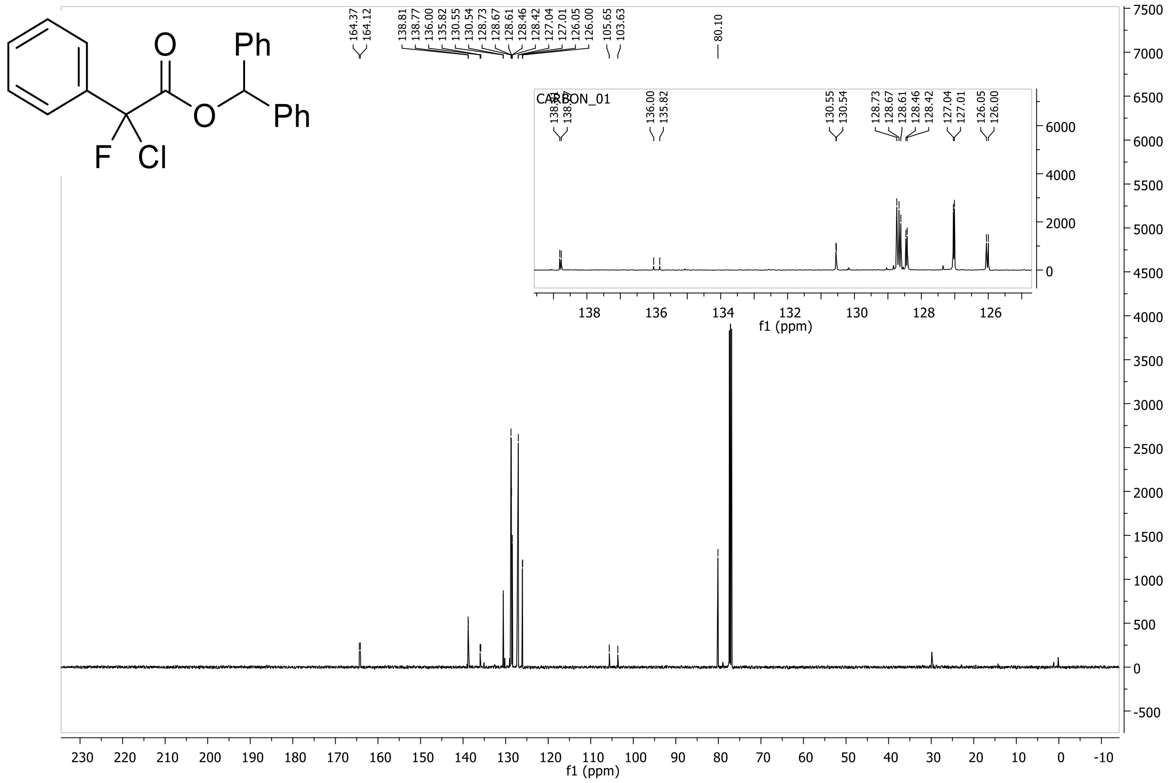

^{19}F NMR (376 MHz, $CDCl_3$) of Molecule **3’c**:

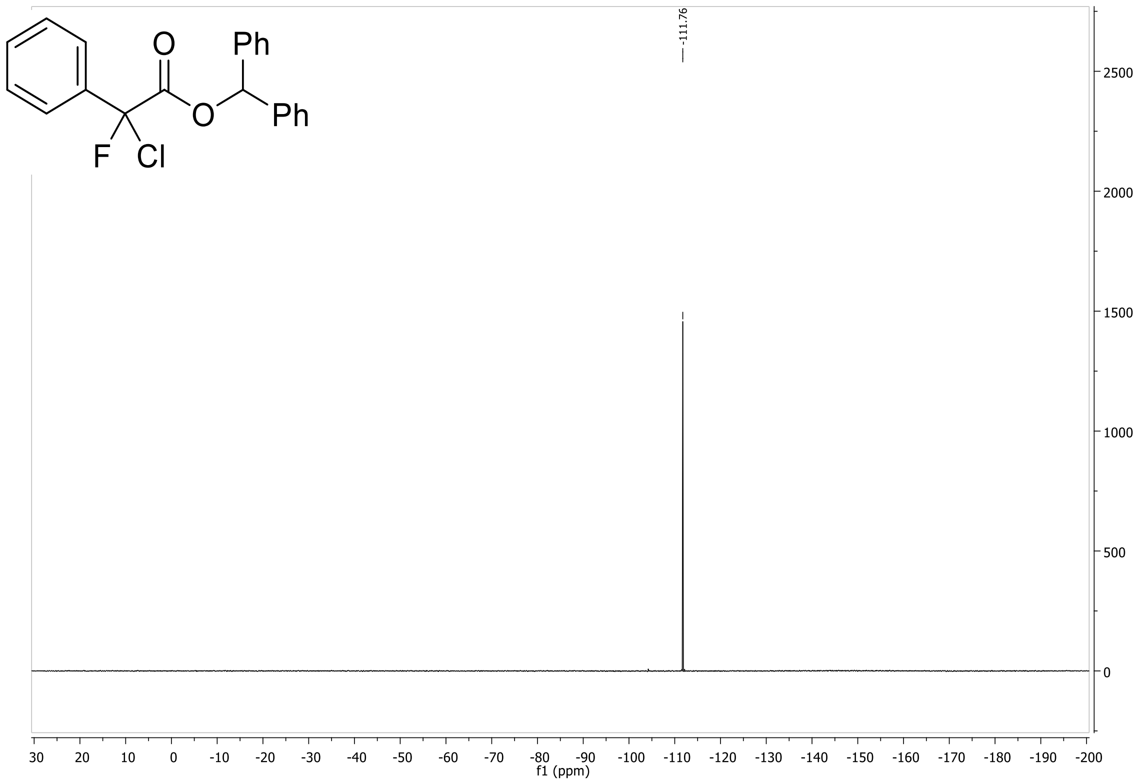

^{1}H NMR *(500 MHz, $CDCl_3$)* of Molecule **3’d**:

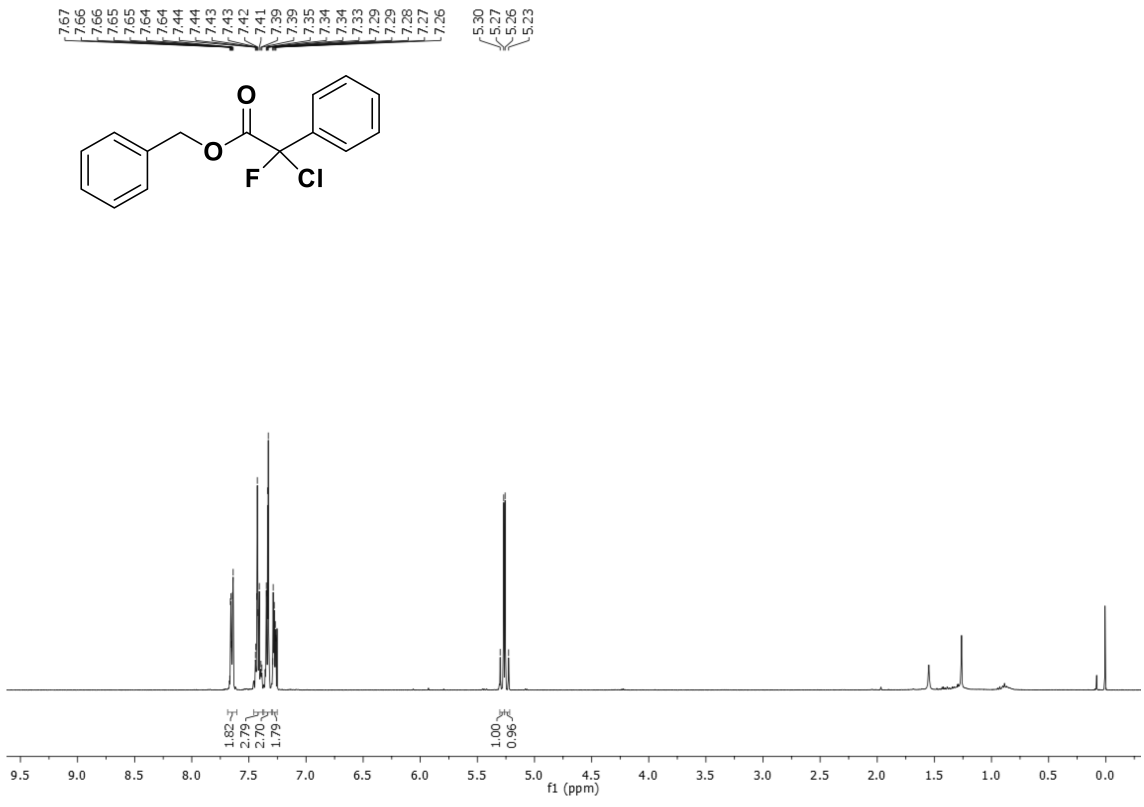

$^{13}C\{H\}$ NMR *(125 MHz, $CDCl_3$)* of Molecule **3’d**:

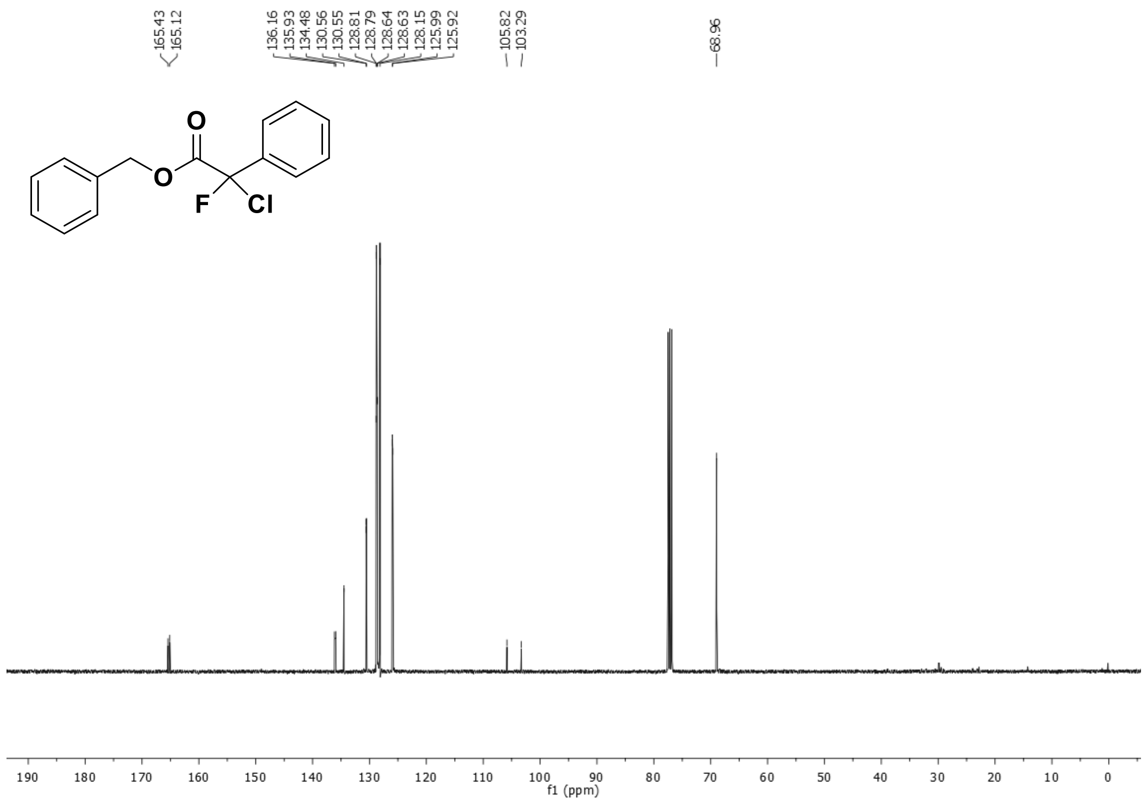

^{19}F NMR (376 MHz, $CDCl_3$) of Molecule **3’d**:

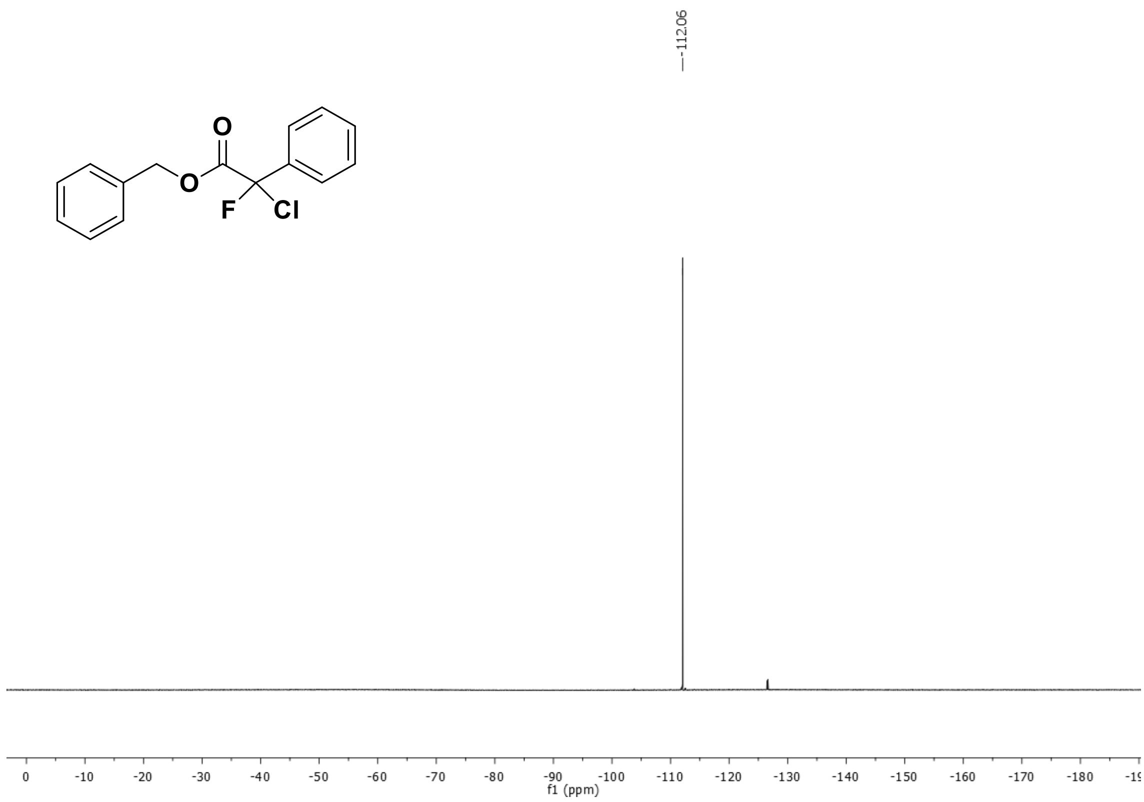

^{1}H NMR *(500 MHz, $CDCl_3$)* of Molecule **3’e**:

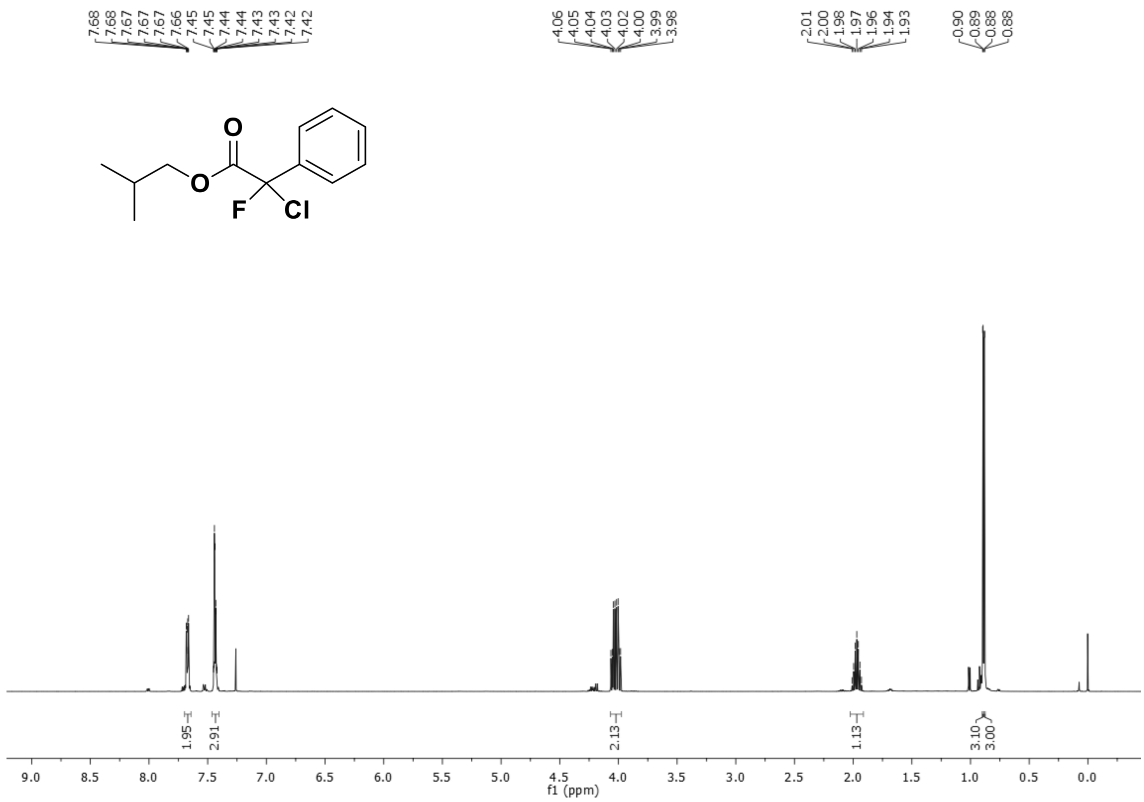

^{13}C{H} NMR *(125 MHz, $CDCl_3$)* of Molecule **3’e**:

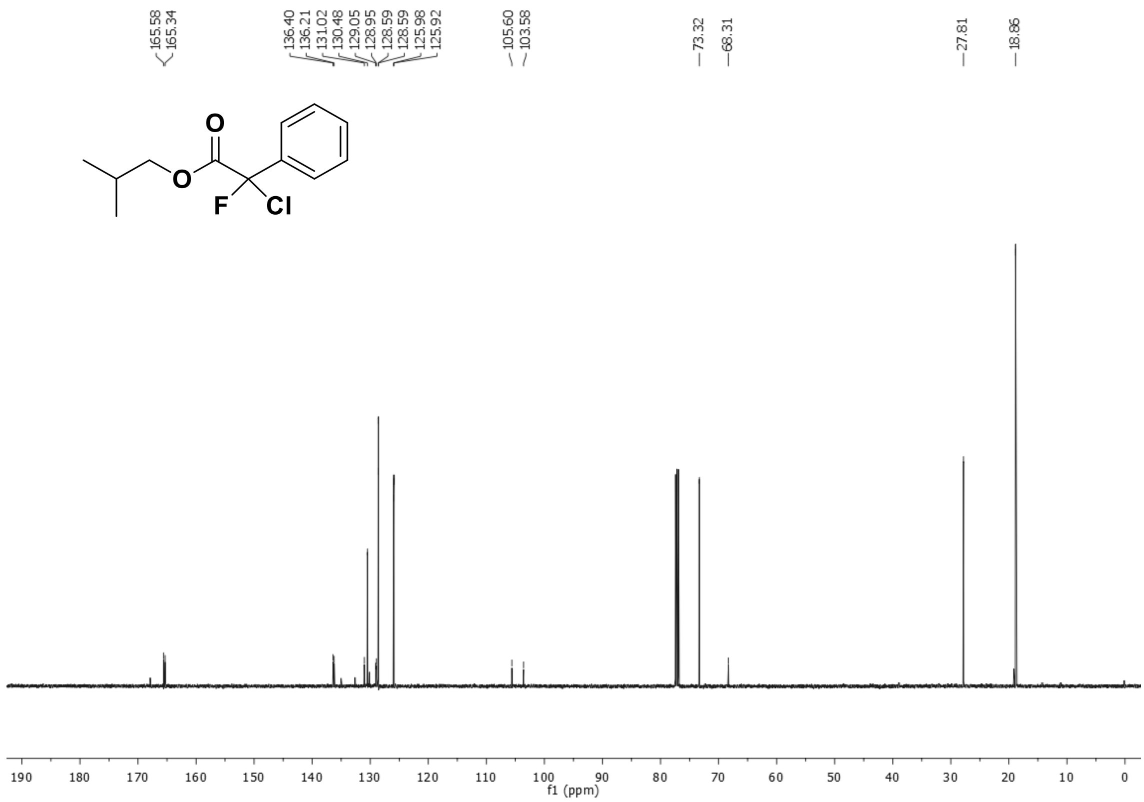

^{19}F NMR (376 MHz, $CDCl_3$) of Molecule **3'e**:

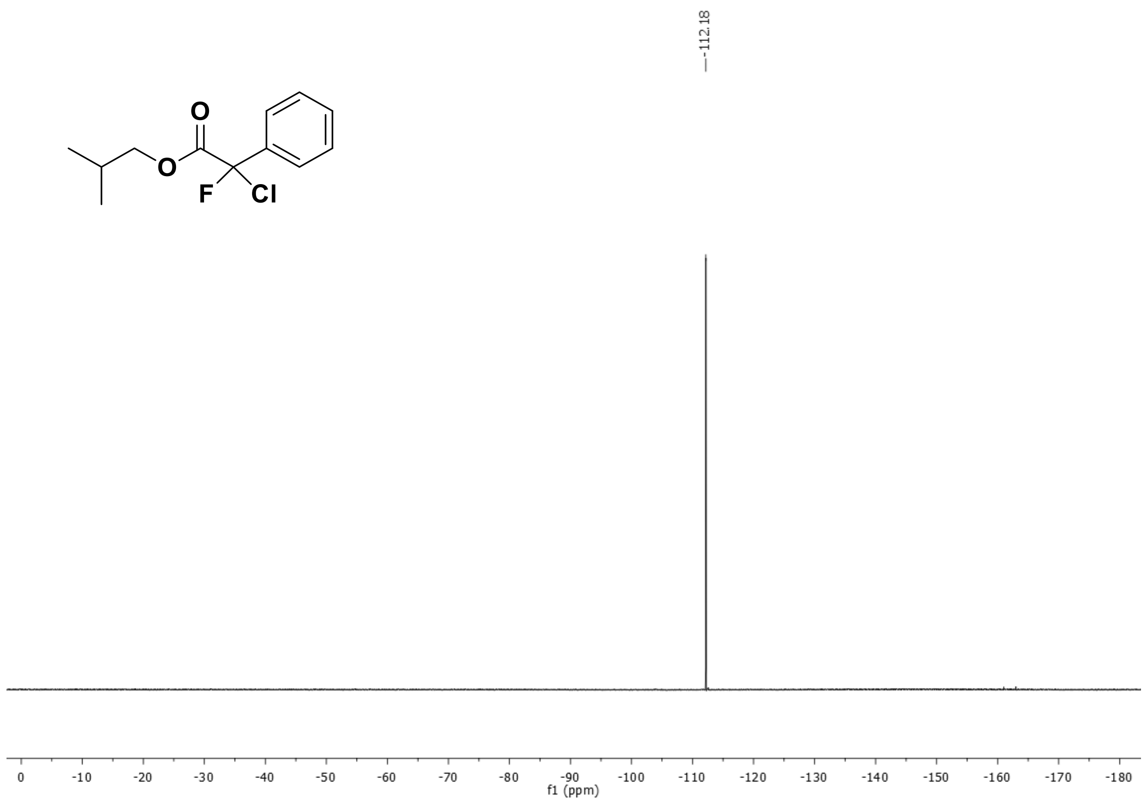

^{1}H NMR *(500 MHz, $CDCl_3$)* of Molecule **3'f**:

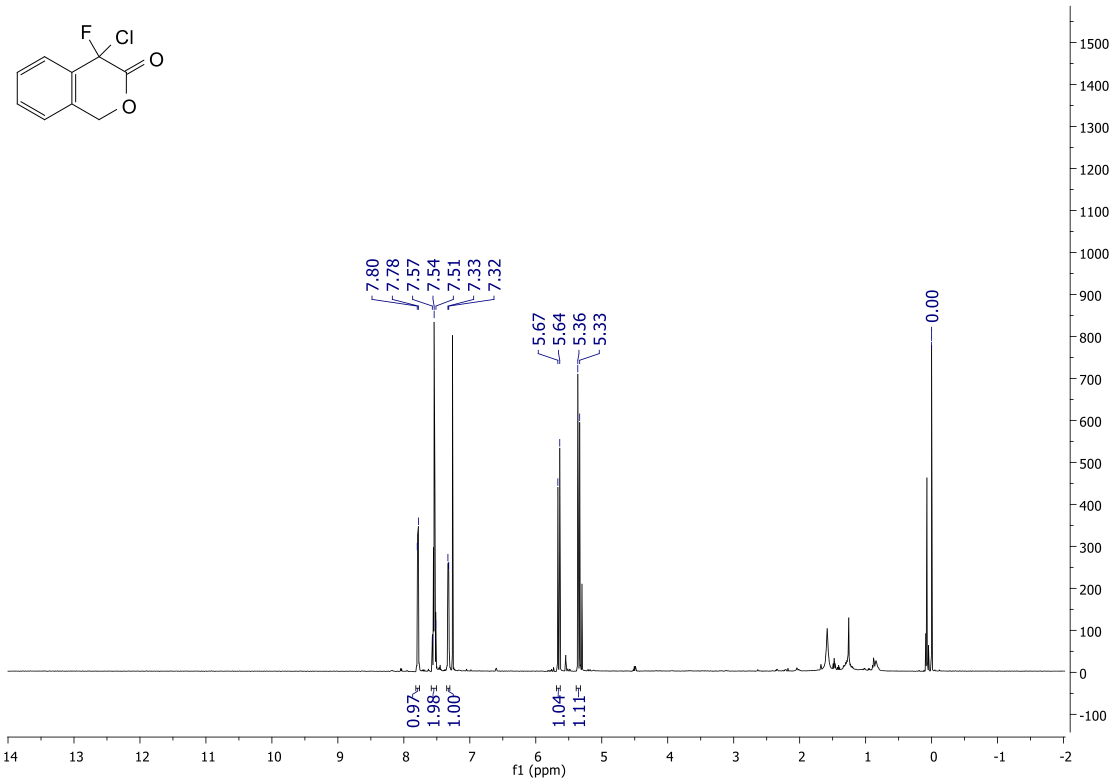

$^{13}C\{H\}$ NMR *(125 MHz, $CDCl_3$)* of Molecule **3’f**:

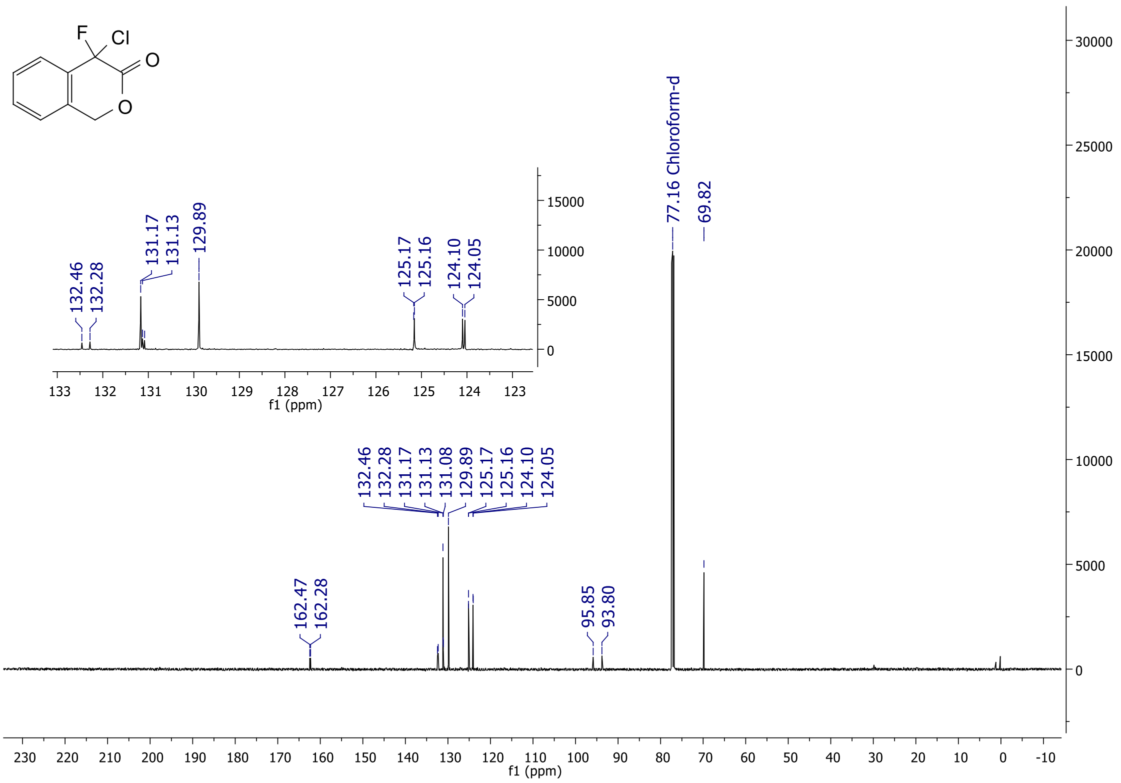

^{19}F NMR (470 MHz, $CDCl_3$) of Molecule **3’f**:

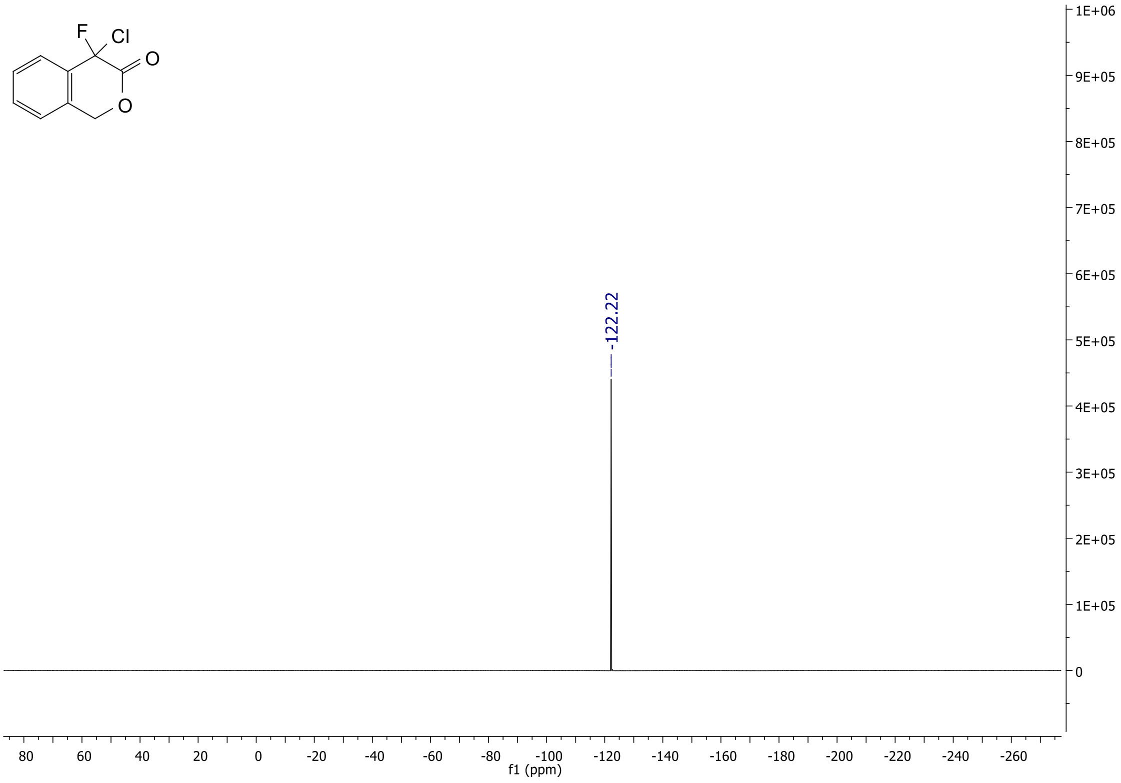

^{1}H NMR *(500 MHz, $CDCl_3$)* of Molecule **3'g**:

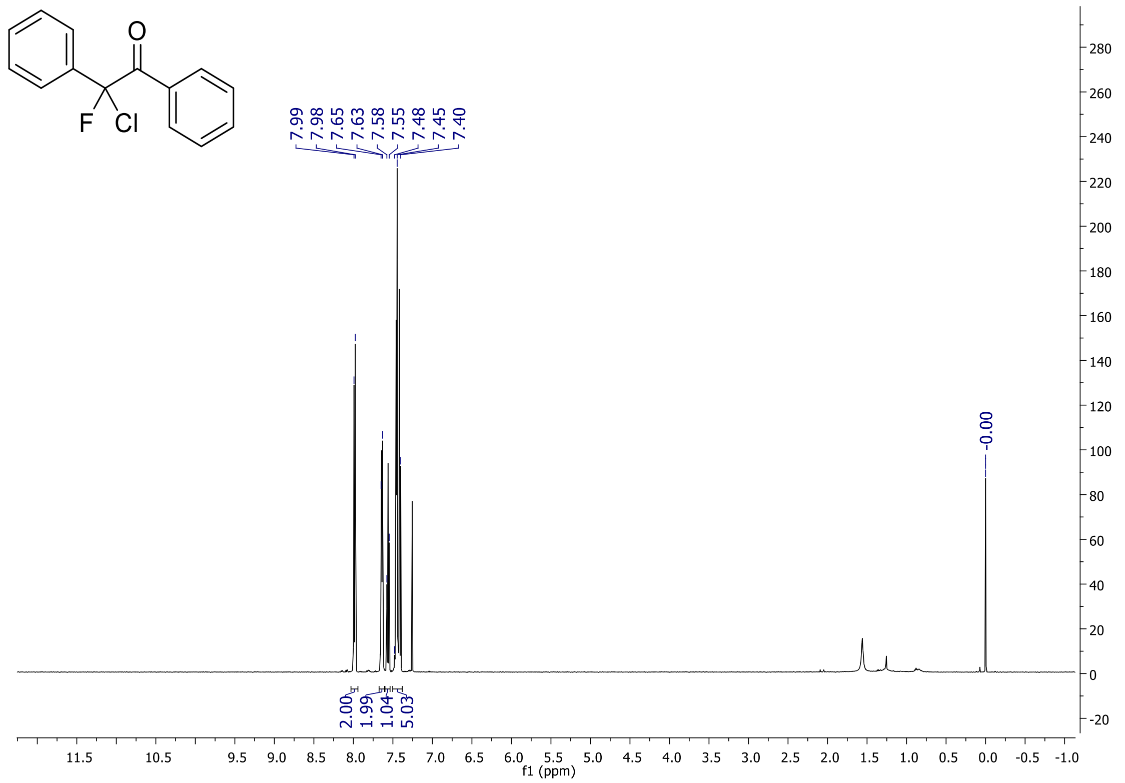

^{13}C{H} NMR *(125 MHz, $CDCl_3$)* of Molecule **3'g**:

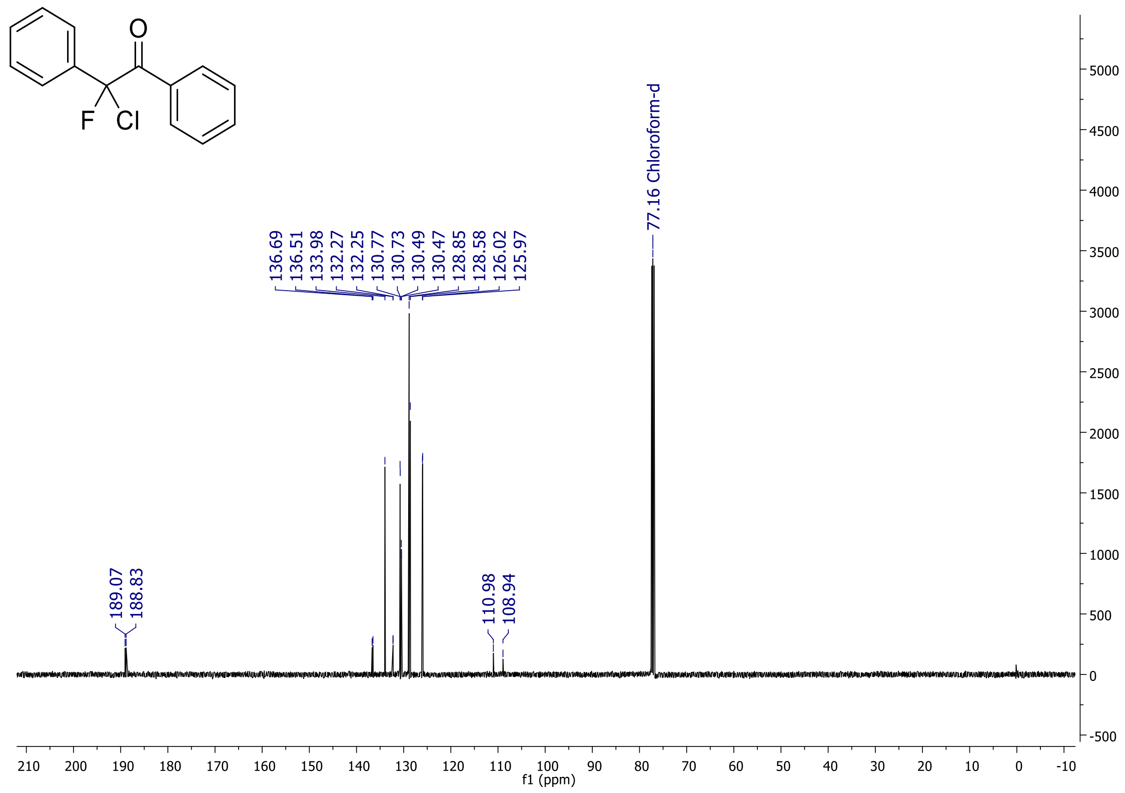

^{19}F NMR (376 MHz, $CDCl_3$) of Molecule **3’g**:

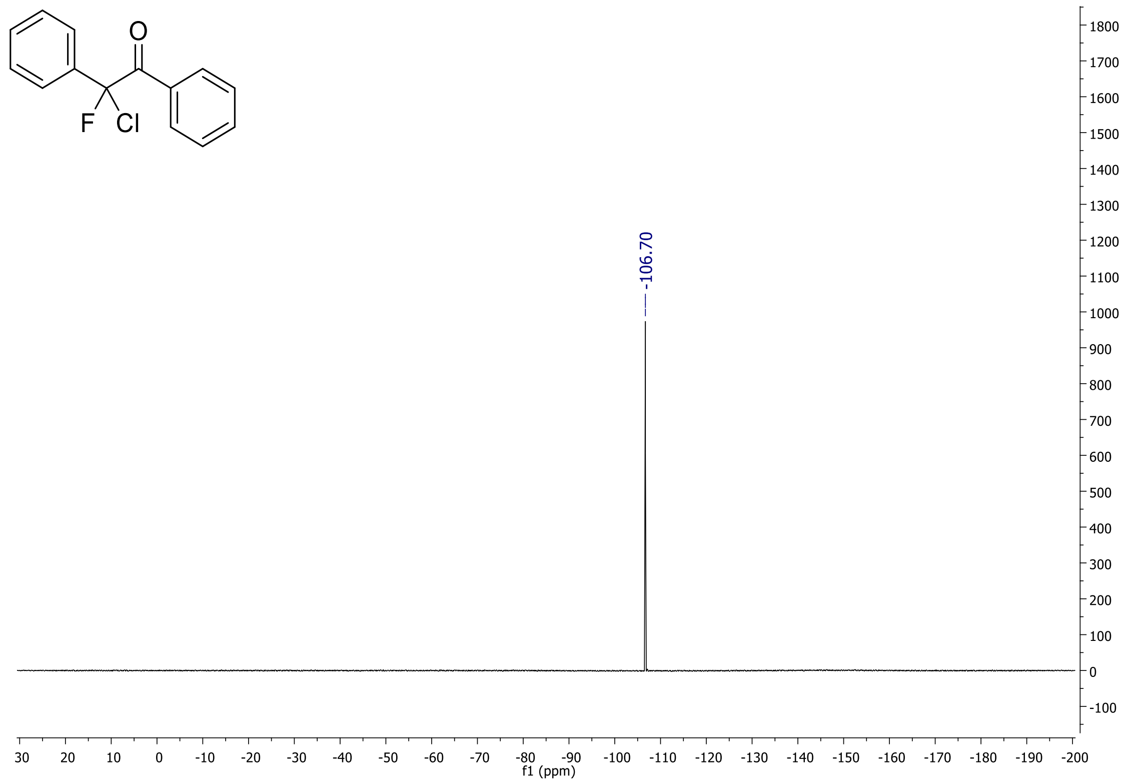

^{1}H NMR *(500 MHz, $CDCl_3$)* of Molecule **3’h**:

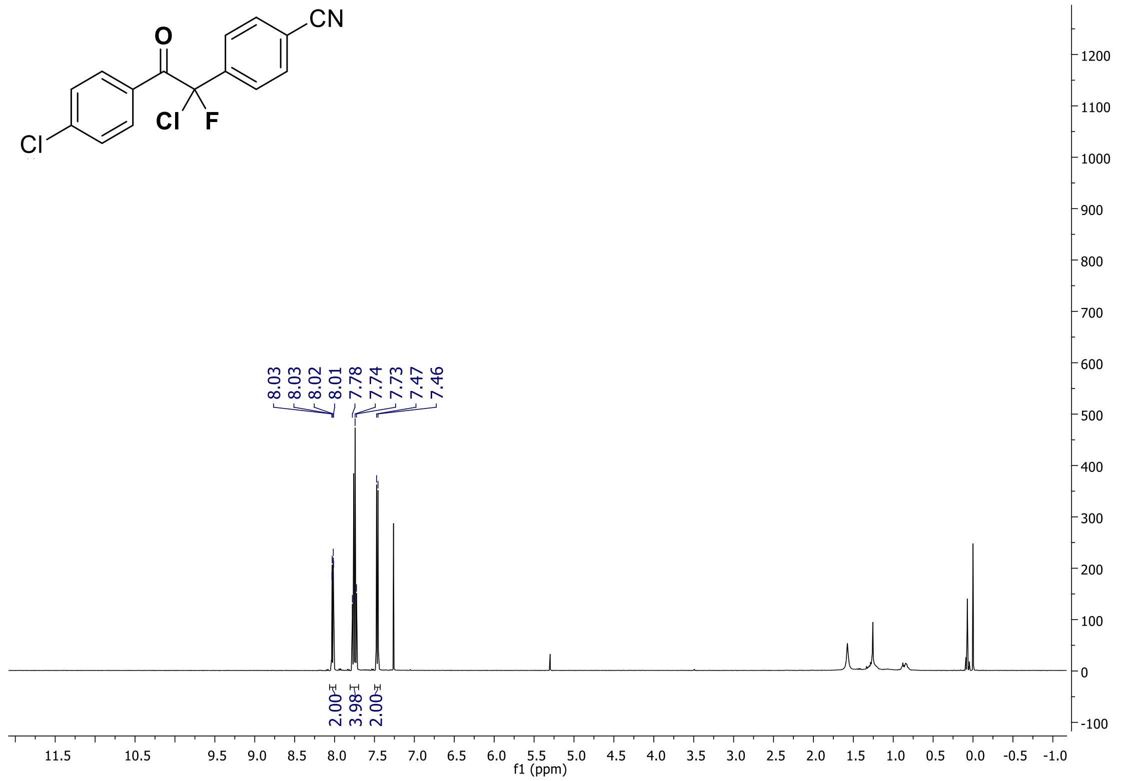

$^{13}C\{H\}$ NMR *(125 MHz, $CDCl_3$)* of Molecule **3'h**:

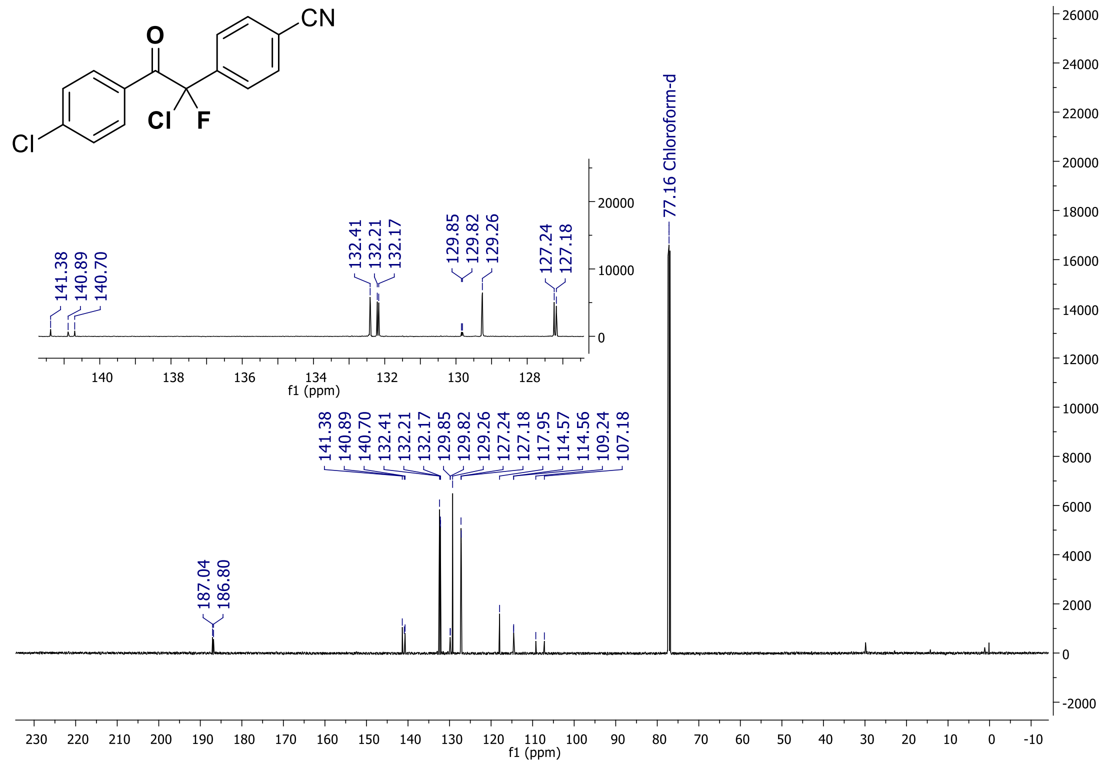

^{19}F NMR (470 MHz, $CDCl_3$) of Molecule **3'h**:

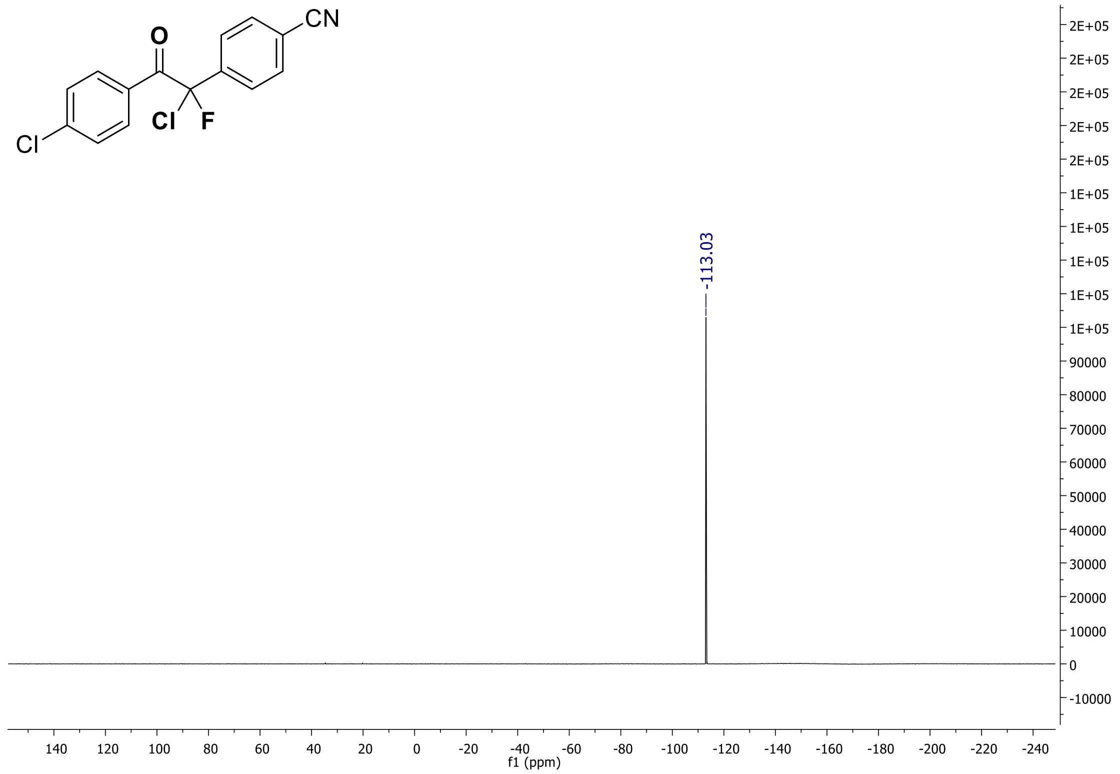

^{1}H NMR *(500 MHz, $CDCl_3$)* of Molecule **3'i**:

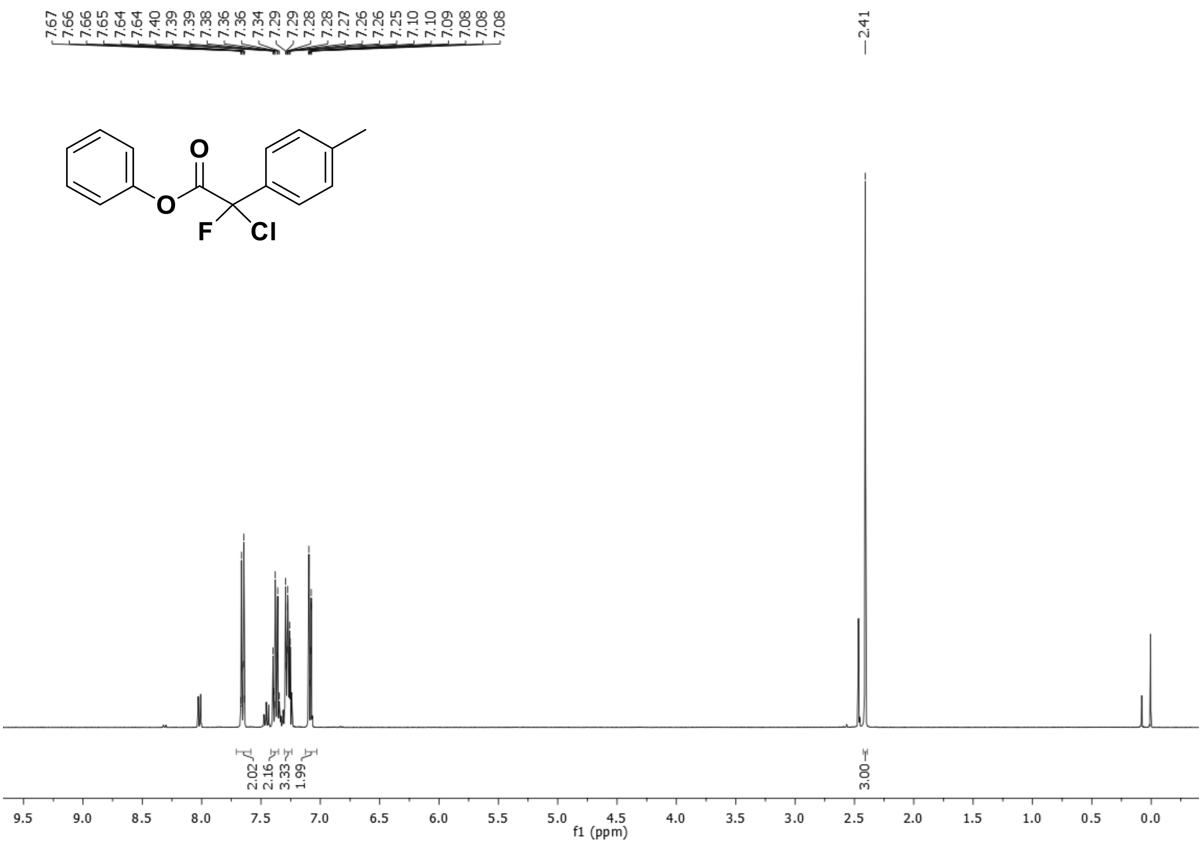

^{13}C{H} NMR *(125 MHz, $CDCl_3$)* of Molecule **3'i**:

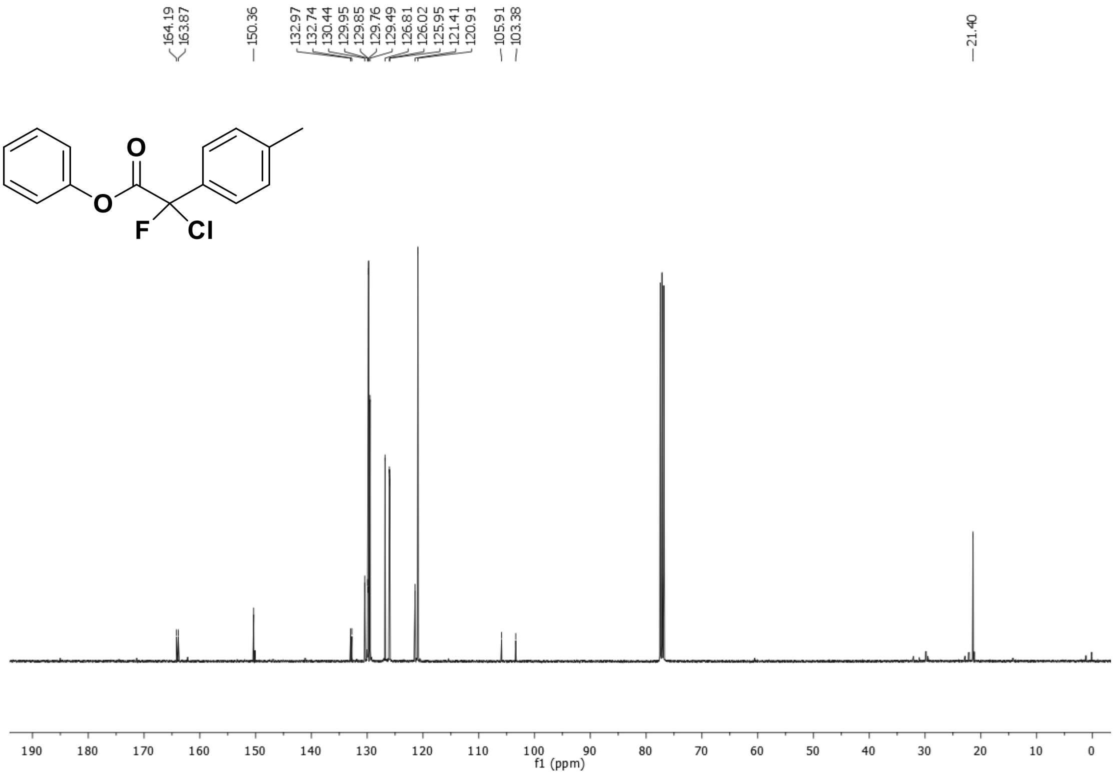

^{19}F NMR (376 MHz, $CDCl_3$) of Molecule **3’i**:

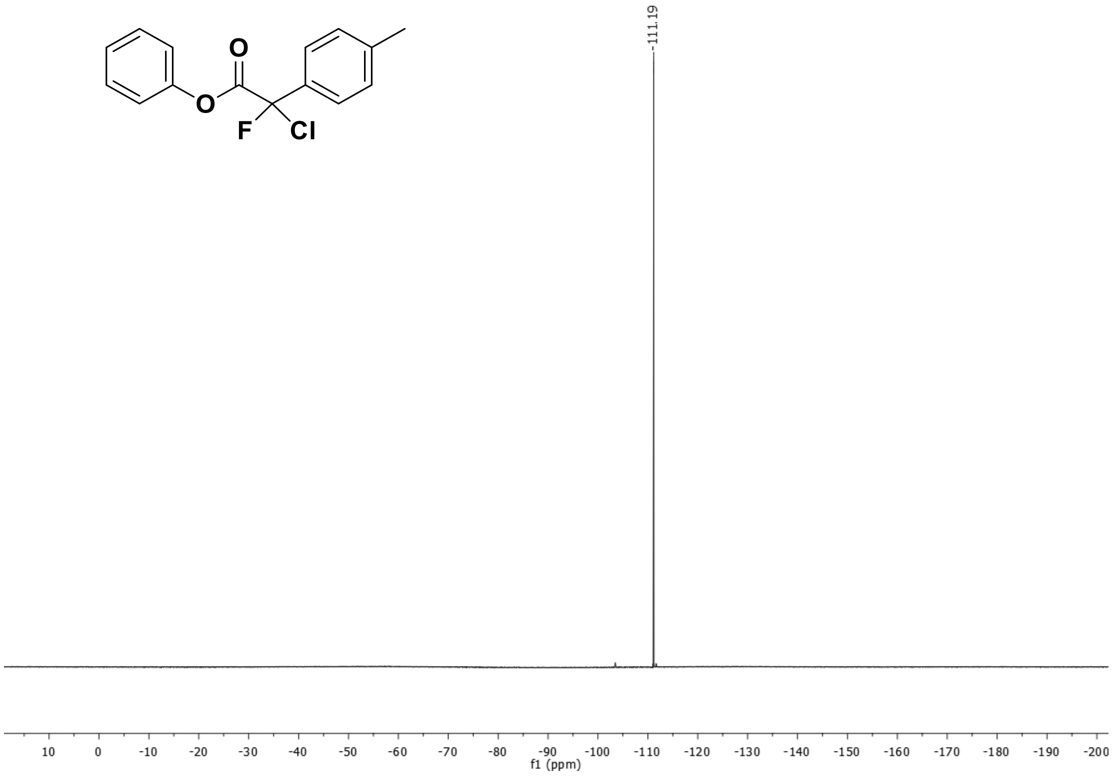

^{1}H NMR *(500 MHz, $CDCl_3$)* of Molecule **3’j**:

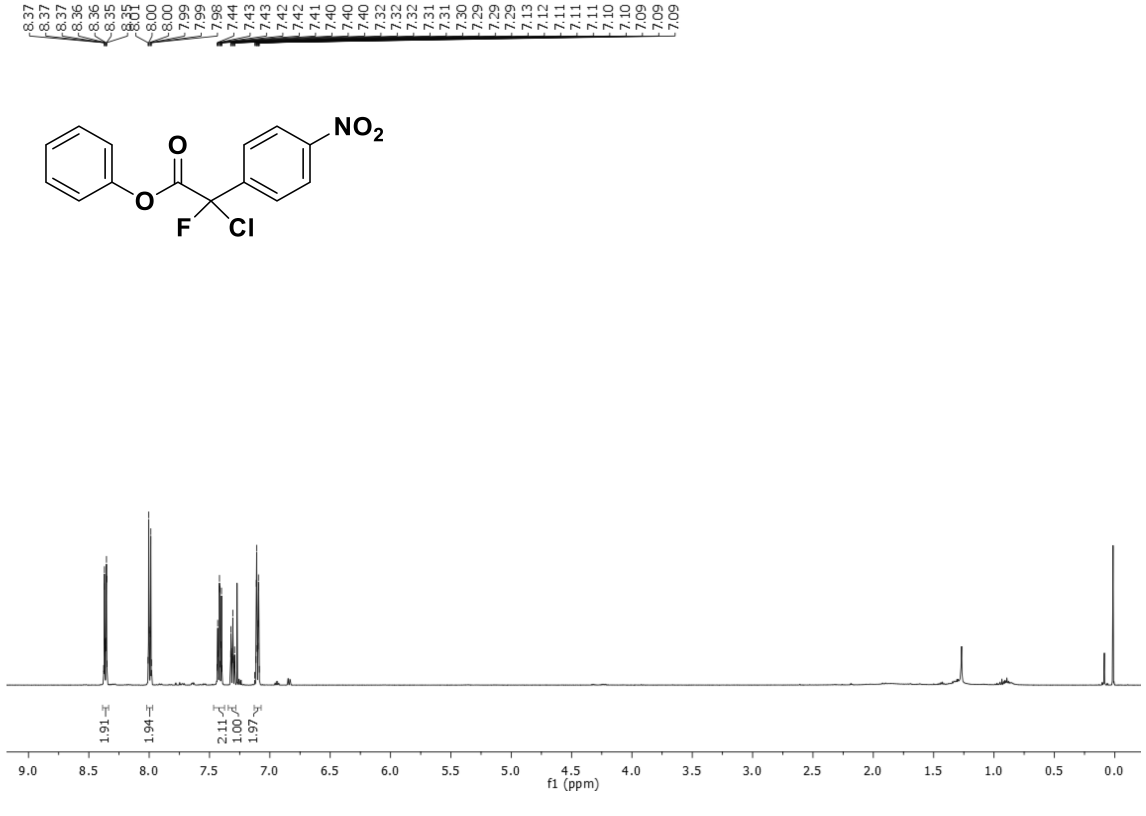

$^{13}C\{H\}$ NMR *(125 MHz, $CDCl_3$)* of Molecule **3'j**:

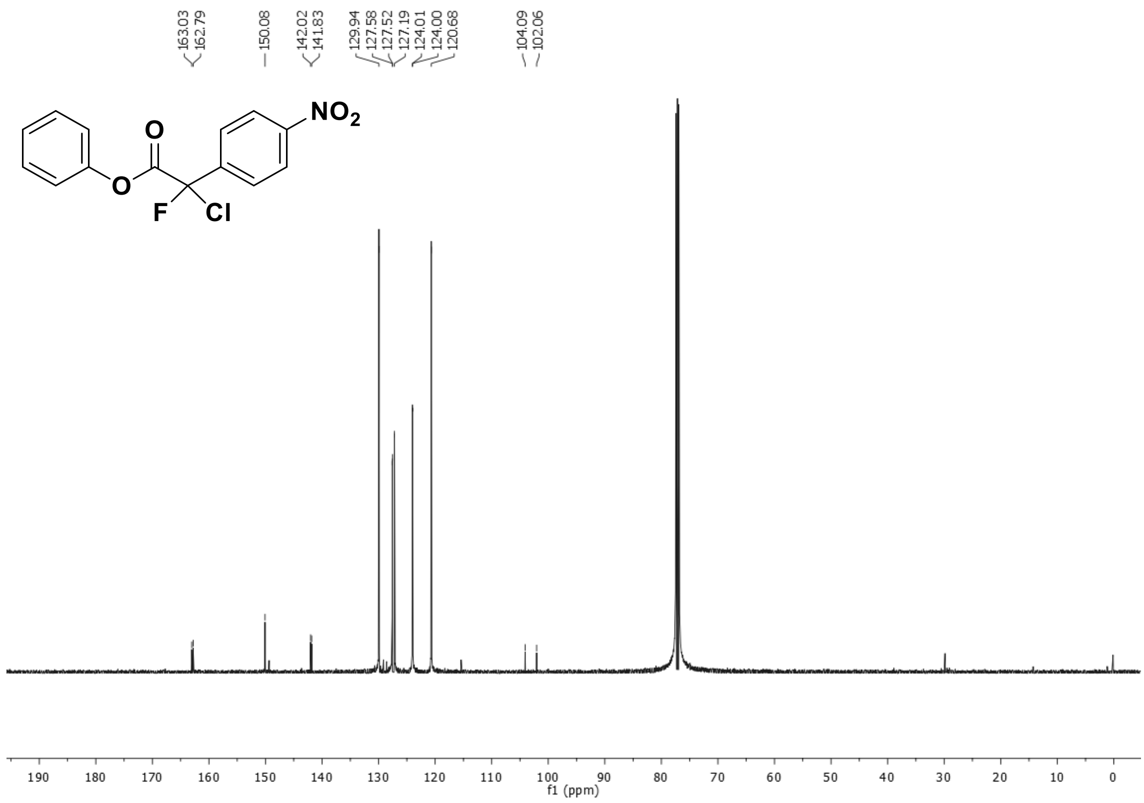

^{19}F NMR (376 MHz, $CDCl_3$) of Molecule **3'j**:

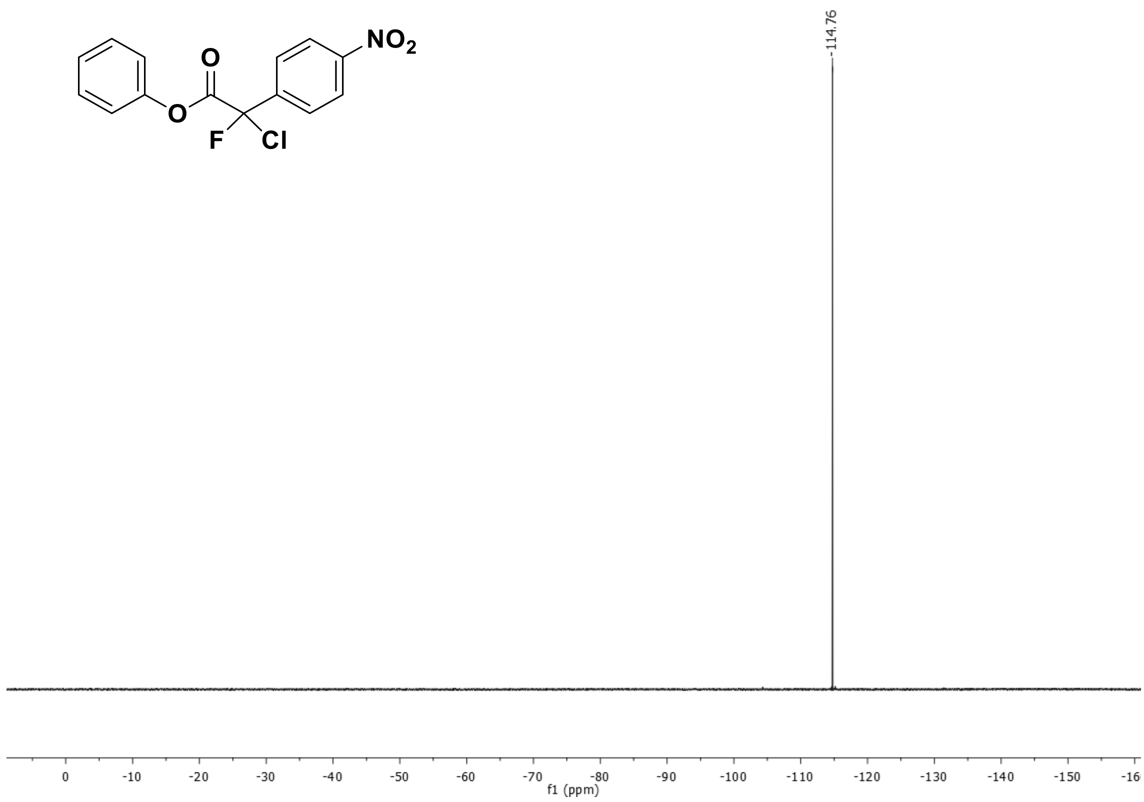

9. REFERENCES

1. Blakemore, P. R.; Burge, M. S. Iterative Stereospecific Reagent-Controlled Homologation of Pinacol Boronates by Enantioenriched α-Chloroalkyllithium Reagents. *J. Am. Chem. Soc.* **2007**, *129* (11), 3068–3069. https://doi.org/10.1021/ja068808s.

2. Zhao, X.; Zheng, X.; Yang, B.; Sheng, J.; Lu, K. Deoxygenation of Sulphoxides to Sulphides with Trichlorophosphane. *Org. Biomol. Chem.* **2018**, *16* (7), 1200–1204. https://doi.org/10.1039/C7OB02834B.

3. Zhu, Y.-C.; Li, Y.; Zhang, B.-C.; Zhang, F.-X.; Yang, Y.-N.; Wang, X.-S. Palladium-Catalyzed Enantioselective C−H Olefination of Diaryl Sulfoxides through Parallel Kinetic Resolution and Desymmetrization. *Angewandte Chemie International Edition* **2018**, *57* (18), 5129–5133. https://doi.org/10.1002/anie.201801146.

4. Carreño, M. C.; Des Mazery, R.; Urbano, A.; Colobert, F.; Solladié, G. Short Asymmetric Synthesis of (−)- and (+)-Cis-Lauthisan. *Org. Lett.* **2005**, *7* (10), 2039–2042. https://doi.org/10.1021/ol050620a.

5. Zenzola, M.; Doran, R.; Degennaro, L.; Luisi, R.; Bull, J. A. Transfer of Electrophilic NH Using Convenient Sources of Ammonia: Direct Synthesis of NH Sulfoximines from Sulfoxides. *Angewandte Chemie International Edition* **2016**, *55* (25), 7203–7207. https://doi.org/10.1002/anie.201602320.

6. Johnson, C. R.; Schroeck, C. W. Chemistry of Sulfoxides and Related Compounds. XLV. Asymmetric Syntheses Using Optically Active Oxosulfonium Alkylides. *J. Am. Chem. Soc.* **1973**, *95* (22), 7418–7423. https://doi.org/10.1021/ja00803a034.

7. Johnson, C. R.; Haake, M.; Schroeck, C. W. Chemistry of Sulfoxides and Related Compounds. XXVI. Preparation and Synthetic Applications of (Dimethylamino)Phenyloxosulfonium Methylide. *J. Am. Chem. Soc.* **1970**, *92* (22), 6594–6598. https://doi.org/10.1021/ja00725a035.

8. Barday, M.; Janot, C.; Halcovitch, N. R.; Muir, J.; Aïssa, C. Cross-Coupling of α-Carbonyl Sulfoxonium Ylides with C−H Bonds. *Angewandte Chemie International Edition* **2017**, *56* (42), 13117–13121. https://doi.org/10.1002/anie.201706804.

9. Janot, C.; Palamini, P.; Dobson, B. C.; Muir, J.; Aïssa, C. Palladium-Catalyzed Synthesis of Bis-Substituted Sulfoxonium Ylides. *Org. Lett.* **2019**, *21* (1), 296–299. https://doi.org/10.1021/acs.orglett.8b03744.

10. Echemendía, R.; De Jesus, M. P.; Furniel, L. G.; Day, D. P.; Burtoloso, A. C. B. Molecular Iodine Mediated Oxidation of Arylated α-Carbonyl Sulfoxonium Ylides to 1,2-Dicarbonyl-Containing Compounds. *European Journal of Organic Chemistry* 2022, *2022* (26), e202200441. https://doi.org/10.1002/ejoc.202200441.

11. He, H.; Yan, K.; Li, J.; Lai, R.; Luo, Y.; Guan, M.; Wu, Y. Metal-Free Insertion of Sulfoxonium Ylides into Arylamines in Water. *Synthesis* **2020**, *52* (20), 3065–3070. https://doi.org/10.1055/s-0040-1707186.

12. Caiuby, C. A. D.; Vidal, L.; Burtoloso, A. C. B.; Aïssa, C. Cyclic Sulfoxonium Ylides: Synthesis and Chemospecific Reactivity in the Catalytic Alkylation of Indoles. *ChemCatChem* **2023**, *15* (7), e202201643. https://doi.org/10.1002/cctc.202201643.

13. Talero, A. G.; Martins, B. S.; Burtoloso, A. C. B. Coupling of Sulfoxonium Ylides with Arynes: A Direct Synthesis of Pro-Chiral Aryl Ketosulfoxonium Ylides and Its Application in the Preparation of α-Aryl Ketones. *Org. Lett.* **2018**, *20* (22), 7206–7211. https://doi.org/10.1021/acs.orglett.8b03126.

14. Hongyu, Z.; Jing, H.; Shangdong, Y. Copper(I) Bromide-Catalyzed for the Synthesis of Sulfoxonium Ylides. *Chinese Journal of Organic Chemistry* **2015**, *35* (9), 1961. https://doi.org/10.6023/cjoc201504005

15. Kitahara, K.; Mizutani, H.; Iwasa, S.; Shibatomi, K. Asymmetric Synthesis of α-Chloro-α-Halo Ketones by Decarboxylative Chlorination of α-Halo-β-Ketocarboxylic Acids. *Synthesis* **2019**, *51* (23), 4385–4392. https://doi.org/10.1055/s-0039-1690009.

16. Hu, L.; Che, C.; Tan, Z.; Zhu, G. A Rapid and Selective Synthesis of α,α-Fluorohalo Esters via Fluorohalogenative or Difluorinative Hydration of Ynol Ethers. *Chem. Commun.* **2015**, *51* (93), 16641–16644. https://doi.org/10.1039/C5CC07471A.

17. Choi, G.; Kim, H. E.; Hwang, S.; Jang, H.; Chung, W. Phosphorus(III)-Mediated, Tandem Deoxygenative Geminal Chlorofluorination of 1,2-Diketones. *Org. Lett.* **2020**, *22* (11), 4190–4195. https://doi.org/10.1021/acs.orglett.0c01258.
18. Rekis, T.; Bērziņš, A.; Orola, L.; Holczbauer, T.; Actiņš, A.; Seidel-Morgenstern, A.; Lorenz, H. Single Enantiomer's Urge to Crystallize in Centrosymmetric Space Groups: Solid Solutions of Phenylpiracetam. *Crystal Growth & Design* **2017**, *17* (3), 1411–1418. https://doi.org/10.1021/acs.cgd.6b01867.
19. Valentín-Pérez, Á.; Rosa, P.; Hillard, E. A.; Giorgi, M. Chirality Determination in Crystals. *Chirality* 2022, *34* (2), 163–181. https://doi.org/10.1002/chir.23377.
